# Supplementary material for: Long-term exposure to particulate air pollution and black carbon in relation to natural and cause-specific mortality: a multicohort study in Sweden
Source: BMJ Open. 2021 Sep 8;11(9):e046040. doi: 10.1136/bmjopen-2020-046040 (PMC8438896; doi:10.1136/bmjopen-2020-046040)

Figure S1a-c. Crude hazard ratios of natural mortality associated with a) PM10, b) PM2.5 and c) BC within each cohort and by random effect meta-analyses estimates. Exposures were assessed by moving average residential concentrations within the exposure windows last 5 years and 6 to 10 years prior.

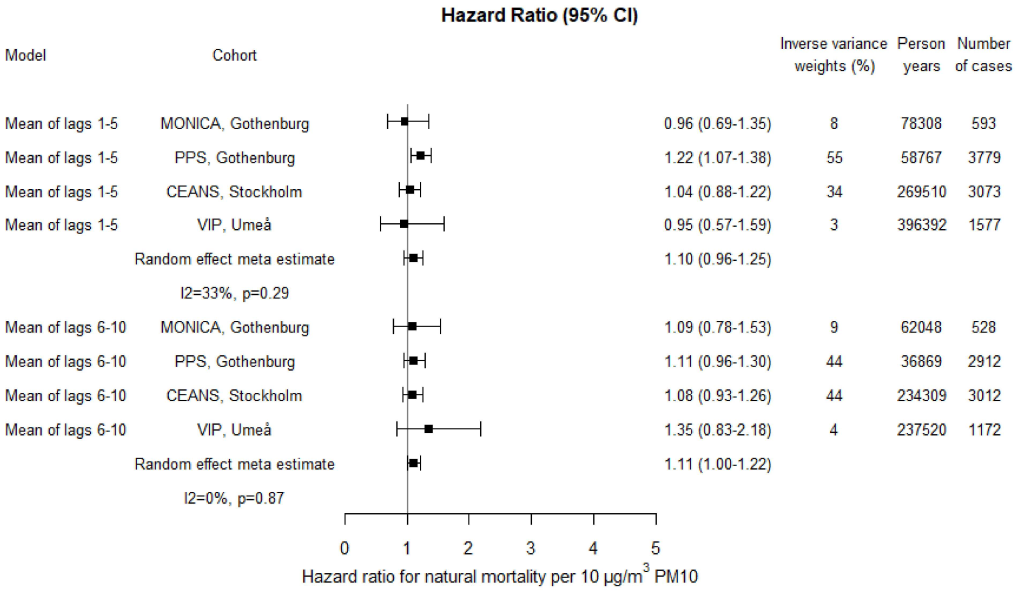

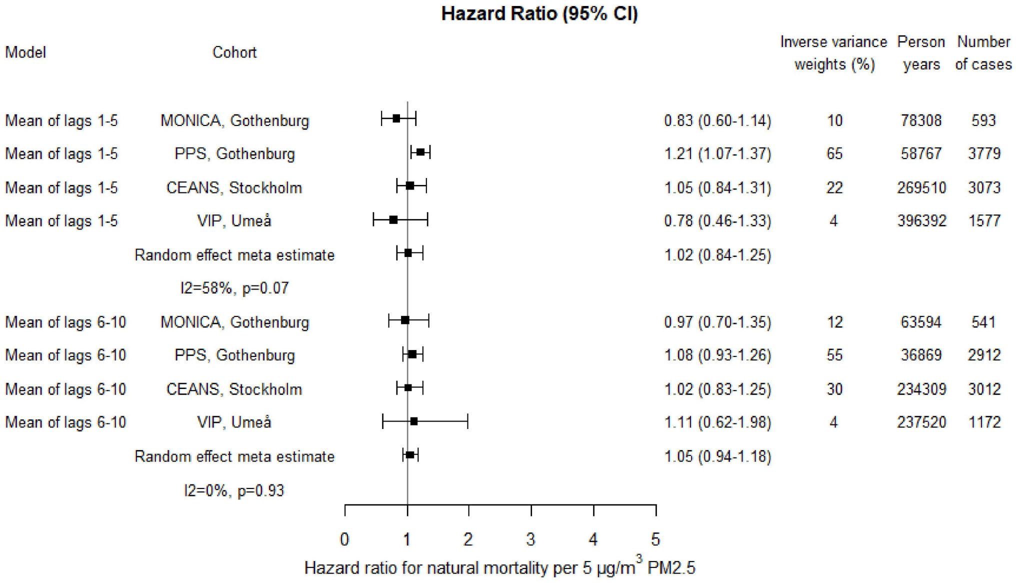

Hazard Ratio (95% CI)

Model	Cohort	Hazard Ratio (95% CI)	Inverse variance weights (%)	Person years	Number of cases
Mean of lags 1-5	MONICA, Gothenburg	1.04 (0.83-1.30)	10	78308	593
Mean of lags 1-5	PPS, Gothenburg	1.11 (1.02-1.21)	74	58767	3779
Mean of lags 1-5	CEANS, Stockholm	1.02 (0.82-1.27)	10	269510	3073
Mean of lags 1-5	VIP, Umeå	1.03 (0.77-1.37)	6	396392	1577
	Random effect meta estimate	1.09 (1.02-1.17)			
	I2=0%, p=0.82				
Mean of lags 6-10	MONICA, Gothenburg	1.04 (0.84-1.29)	12	63594	541
Mean of lags 6-10	PPS, Gothenburg	1.13 (1.03-1.24)	64	36869	2912
Mean of lags 6-10	CEANS, Stockholm	1.10 (0.92-1.32)	16	234309	3012
Mean of lags 6-10	VIP, Umeå	1.19 (0.93-1.53)	9	237520	1172
	Random effect meta estimate	1.12 (1.04-1.20)			
	I2=0%, p=0.87				

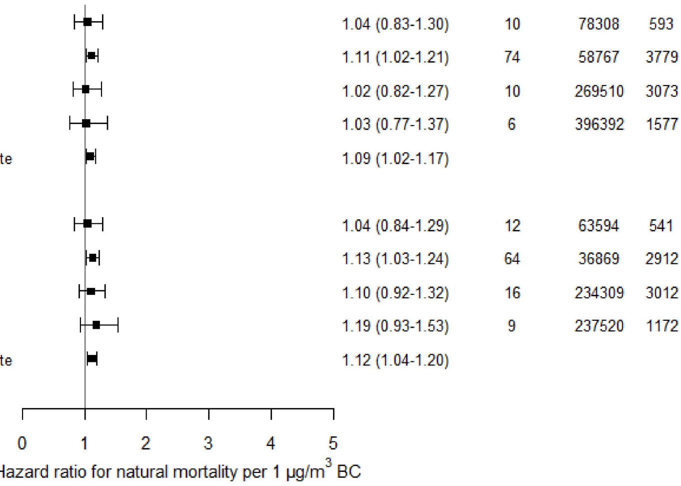

Figure S2a-c. Crude hazard ratios of CVD mortality associated with a) PM10, b) PM2.5 and c) BC within each cohort and by random effect meta-analyses estimates. Exposures were assessed by moving average residential concentrations within the exposure windows last 5 years and 6 to 10 years prior.

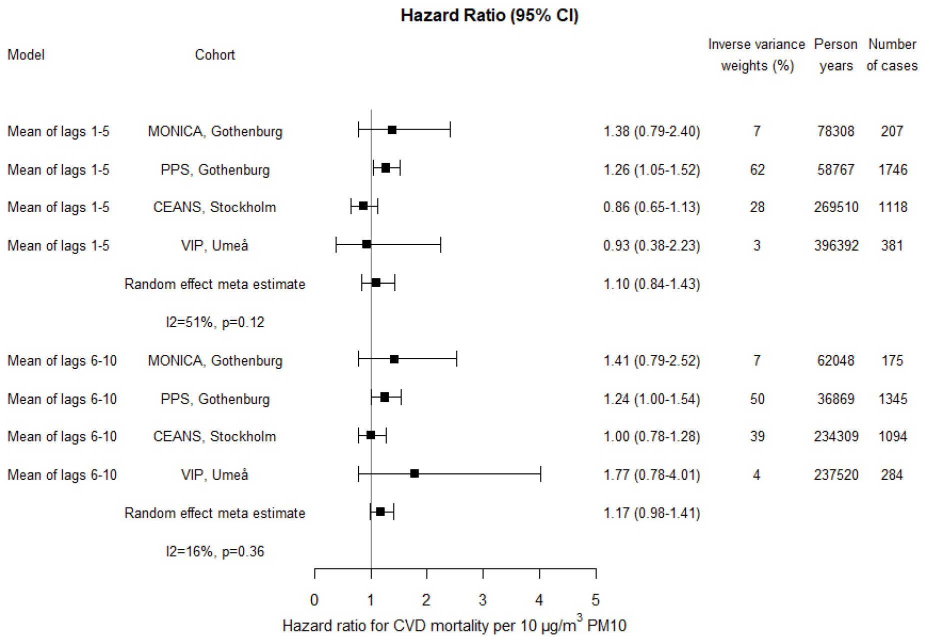

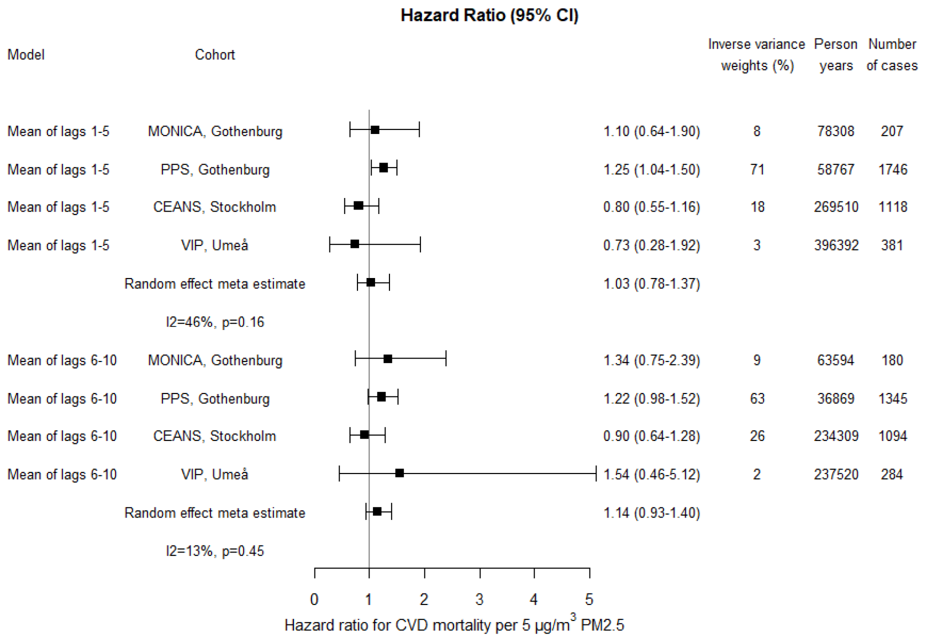

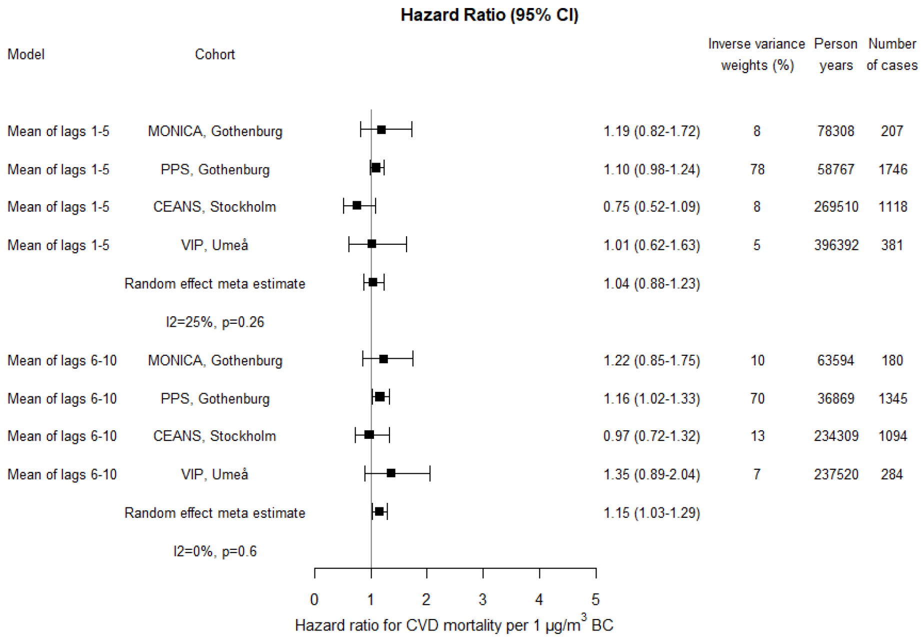
Hazard Ratio (95% CI)
Model
Cohort
Inverse variance weights (%)
Person years
Number of cases
Mean of lags 1-5 MONICA, Gothenburg 1.19 (0.82-1.72) 8 78308 207
Mean of lags 1-5 PPS, Gothenburg 1.10 (0.98-1.24) 78 58767 1746
Mean of lags 1-5 CEANS, Stockholm 0.75 (0.52-1.09) 8 269510 1118
Mean of lags 1-5 VIP, Umeå 1.01 (0.62-1.63) 5 396392 381
Random effect meta estimate 1.04 (0.88-1.23)
I2=25%, p=0.26
Mean of lags 6-10 MONICA, Gothenburg 1.22 (0.85-1.75) 10 63594 180
Mean of lags 6-10 PPS, Gothenburg 1.16 (1.02-1.33) 70 36869 1345
Mean of lags 6-10 CEANS, Stockholm 0.97 (0.72-1.32) 13 234309 1094
Mean of lags 6-10 VIP, Umeå 1.35 (0.89-2.04) 7 237520 284
Random effect meta estimate 1.15 (1.03-1.29)
I2=0%, p=0.6
0
1
2
3
4
5
Hazard ratio for CVD mortality per 1 μg/m3 BC

Figure S3a-c. Adjusted hazard ratios of lung cancer mortality associated with a) PM10, b) PM2.5 and c) BC within each cohort and by random effect meta-analyses estimates. Exposures were assessed by moving average residential concentrations within the exposure windows last 5 years and 6 to 10 years prior.

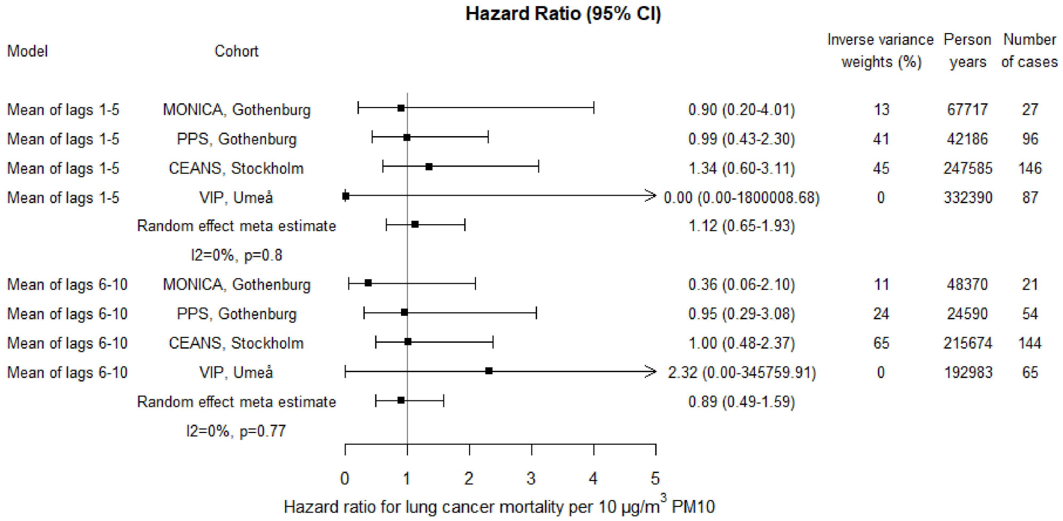

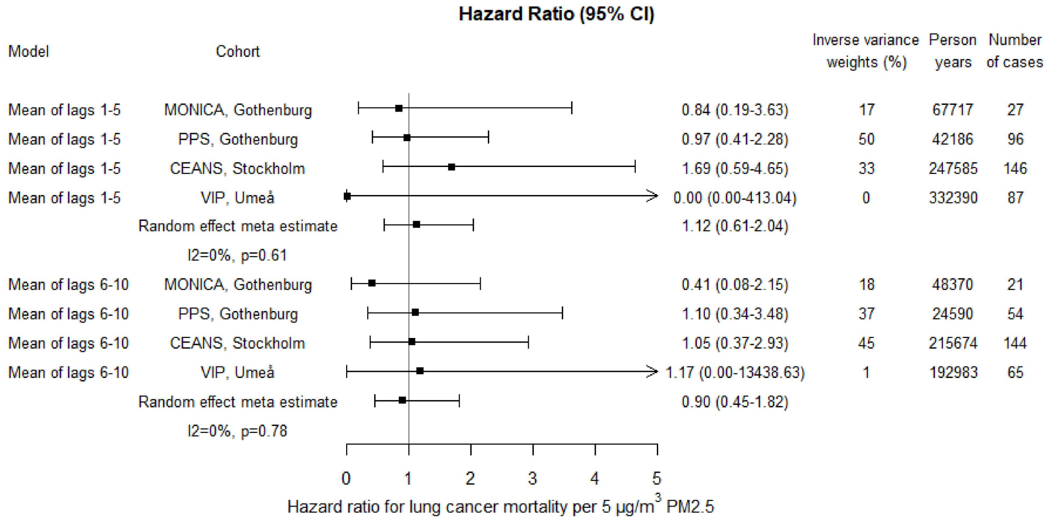

Hazard Ratio (95% CI)

Model	Cohort	Hazard Ratio (95% CI)	Inverse variance weights (%)	Person years	Number of cases
Mean of lags 1-5	MONICA, Gothenburg	0.48 (0.15-1.60)	13	67717	27
Mean of lags 1-5	PPS, Gothenburg	0.99 (0.58-1.69)	65	42186	96
Mean of lags 1-5	CEANS, Stockholm	1.16 (0.38-3.56)	15	247585	146
Mean of lags 1-5	VIP, Umeå	0.52 (0.10-2.62)	7	332390	87
	Random effect meta estimate	0.88 (0.57-1.36)			
	I2=0%, p=0.62				
Mean of lags 6-10	MONICA, Gothenburg	0.37 (0.10-1.42)	10	48370	21
Mean of lags 6-10	PPS, Gothenburg	0.84 (0.40-1.77)	31	24590	54
Mean of lags 6-10	CEANS, Stockholm	0.98 (0.36-2.71)	17	215674	144
Mean of lags 6-10	VIP, Umeå	1.03 (0.55-1.94)	43	192983	65
	Random effect meta estimate	0.87 (0.57-1.32)			
	I2=0%, p=0.59				

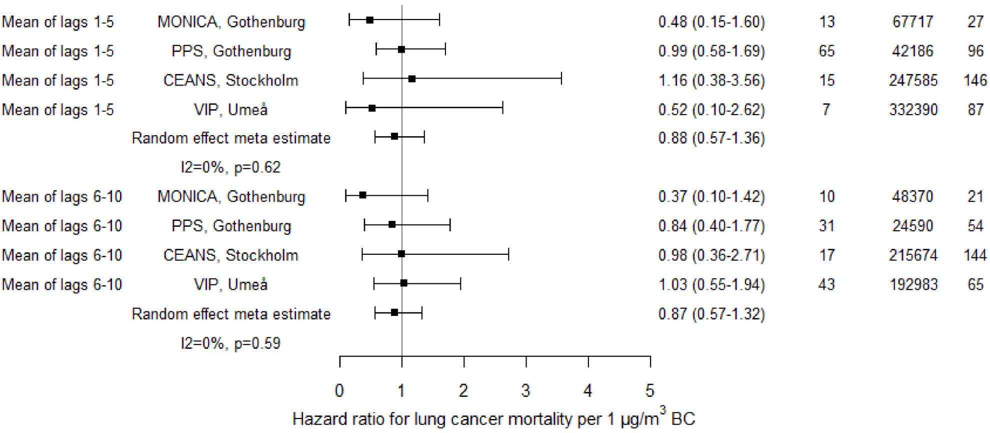

Figure S4a-c. Adjusted hazard ratios of respiratory mortality associated with a) PM10, b) PM2.5 and c) BC within each cohort and by random effect meta-analyses estimates. Exposures were assessed by moving average residential concentrations within the exposure windows last 5 years and 6 to 10 years prior.

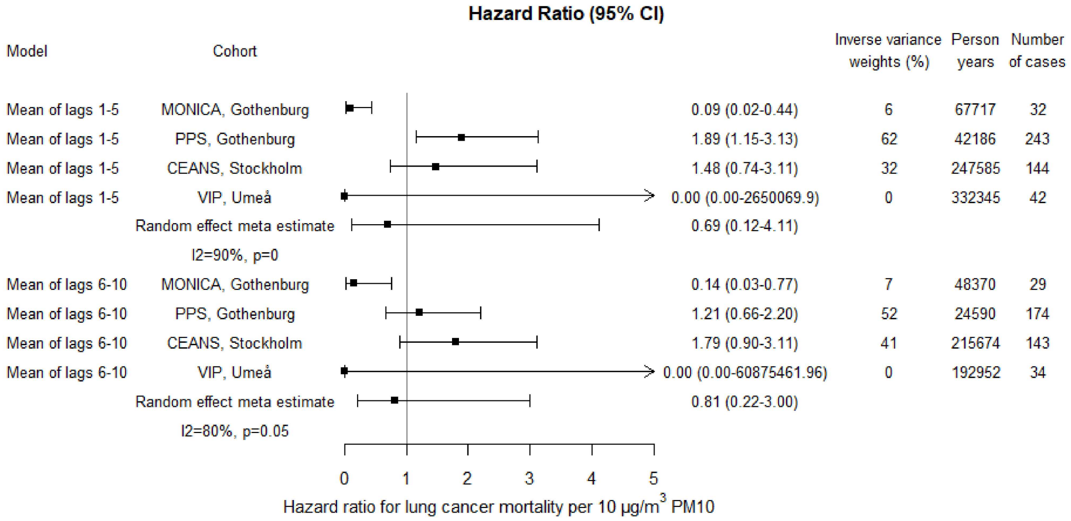

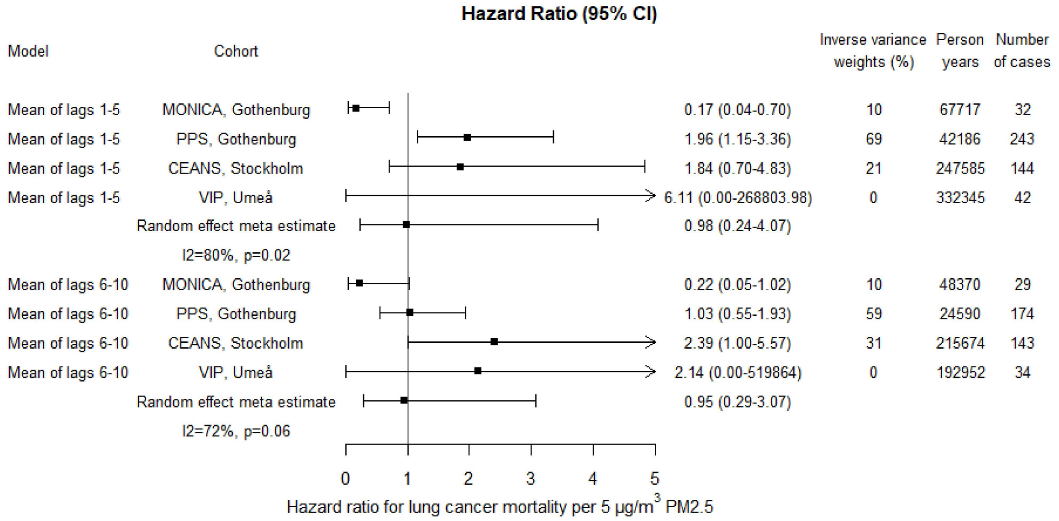

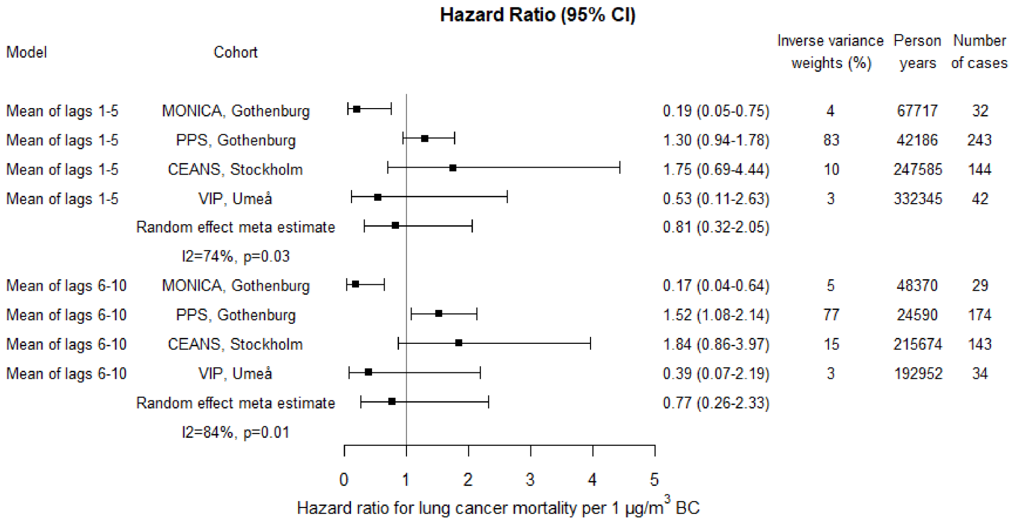

Hazard Ratio (95% CI)
Model
Cohort
Inverse variance weights (%)
Person years
Number of cases
Mean of lags 1-5 MONICA, Gothenburg 0.19 (0.05-0.75) 4 67717 32
Mean of lags 1-5 PPS, Gothenburg 1.30 (0.94-1.78) 83 42186 243
Mean of lags 1-5 CEANS, Stockholm 1.75 (0.69-4.44) 10 247585 144
Mean of lags 1-5 VIP, Umeå 0.53 (0.11-2.63) 3 332345 42
Random effect meta estimate 0.81 (0.32-2.05)
I2=74%, p=0.03
Mean of lags 6-10 MONICA, Gothenburg 0.17 (0.04-0.64) 5 48370 29
Mean of lags 6-10 PPS, Gothenburg 1.52 (1.08-2.14) 77 24590 174
Mean of lags 6-10 CEANS, Stockholm 1.84 (0.86-3.97) 15 215674 143
Mean of lags 6-10 VIP, Umeå 0.39 (0.07-2.19) 3 192952 34
Random effect meta estimate 0.77 (0.26-2.33)
I2=84%, p=0.01
0 1 2 3 4 5
Hazard ratio for lung cancer mortality per 1 µg/m3 BC

Figure S5a-c. Adjusted hazard ratios of mortality by other casues (not CVD, respiratory, lung cancer or external causes) associated with a) PM10, b) PM2.5 and c) BC within each cohort and by random effect meta-analyses estimates. Exposures were assessed by moving average residential concentrations within the exposure windows last 5 years and 6 to 10 years prior.

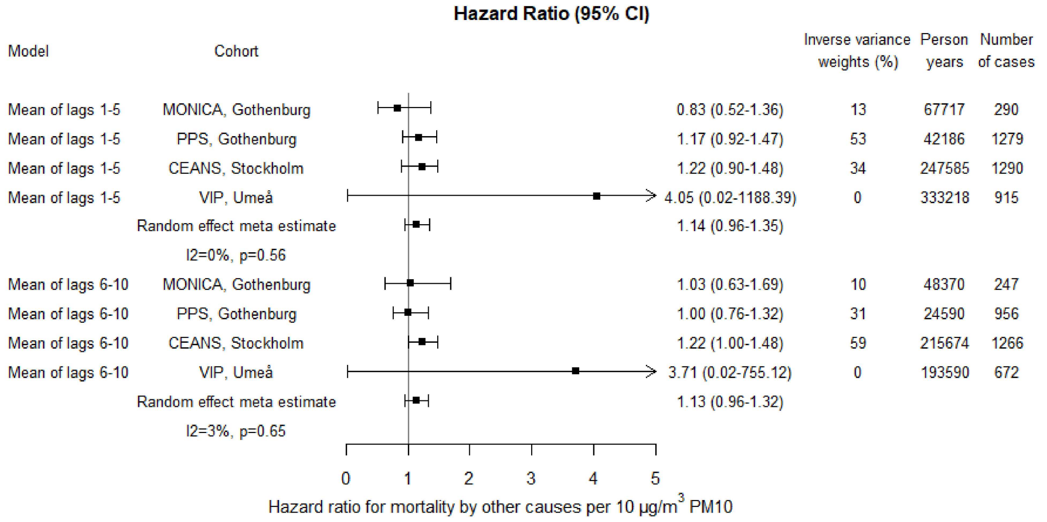

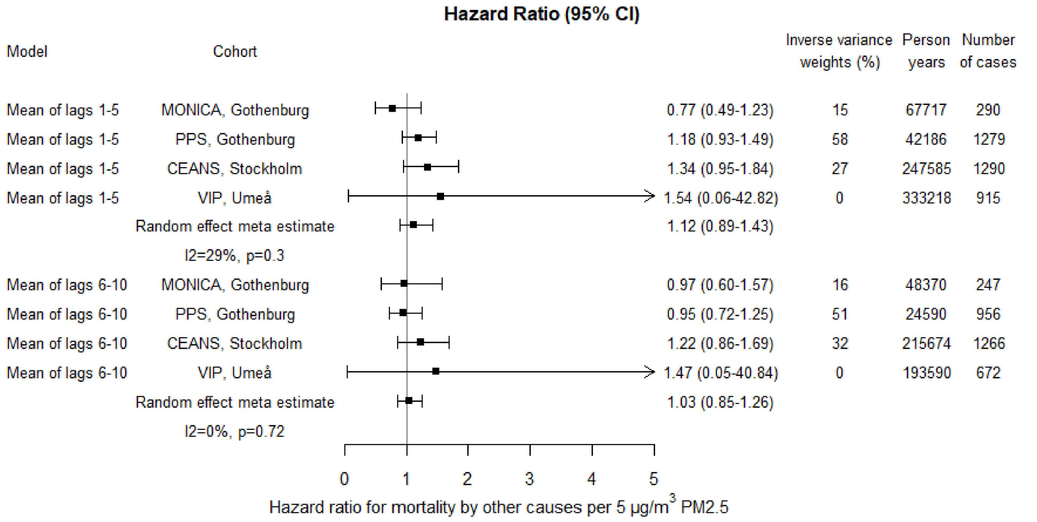

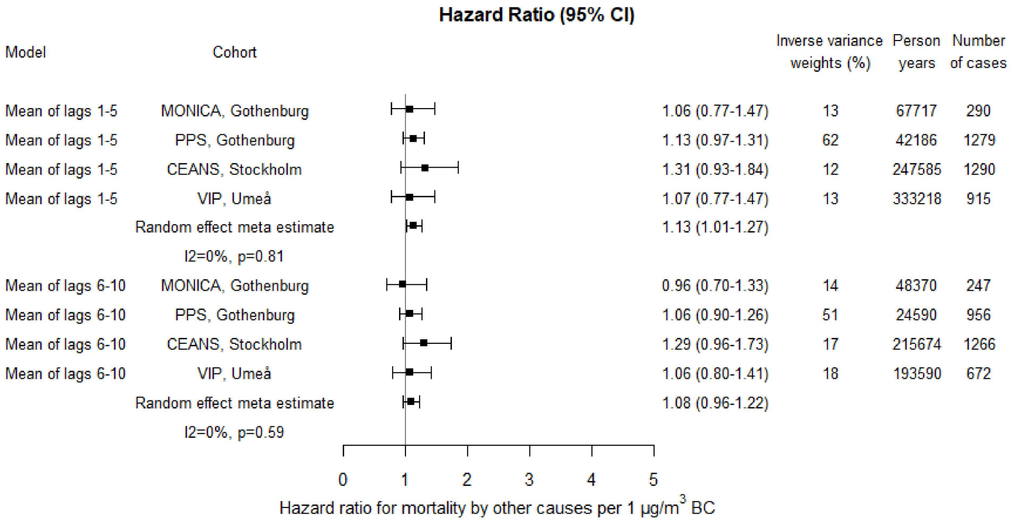
Hazard Ratio (95% CI)
Model
Cohort
Inverse variance weights (%)
Person years
Number of cases
Mean of lags 1-5 MONICA, Gothenburg 1.06 (0.77-1.47) 13 67717 290
Mean of lags 1-5 PPS, Gothenburg 1.13 (0.97-1.31) 62 42186 1279
Mean of lags 1-5 CEANS, Stockholm 1.31 (0.93-1.84) 12 247585 1290
Mean of lags 1-5 VIP, Umeå 1.07 (0.77-1.47) 13 333218 915
Random effect meta estimate 1.13 (1.01-1.27)
I2=0%, p=0.81
Mean of lags 6-10 MONICA, Gothenburg 0.96 (0.70-1.33) 14 48370 247
Mean of lags 6-10 PPS, Gothenburg 1.06 (0.90-1.26) 51 24590 956
Mean of lags 6-10 CEANS, Stockholm 1.29 (0.96-1.73) 17 215674 1266
Mean of lags 6-10 VIP, Umeå 1.06 (0.80-1.41) 18 193590 672
Random effect meta estimate 1.08 (0.96-1.22)
I2=0%, p=0.59
0
1
2
3
4
5
Hazard ratio for mortality by other causes per 1 μg/m3 BC

Figure S6a-b. Adjusted hazard ratios of a) natural mortality and b) CVD mortality associated with PM10, PM2.5 and BC, within each cohort and by random effect meta-analyses estimates. Exposures were assessed by the residential concentration at the year of recruitment.

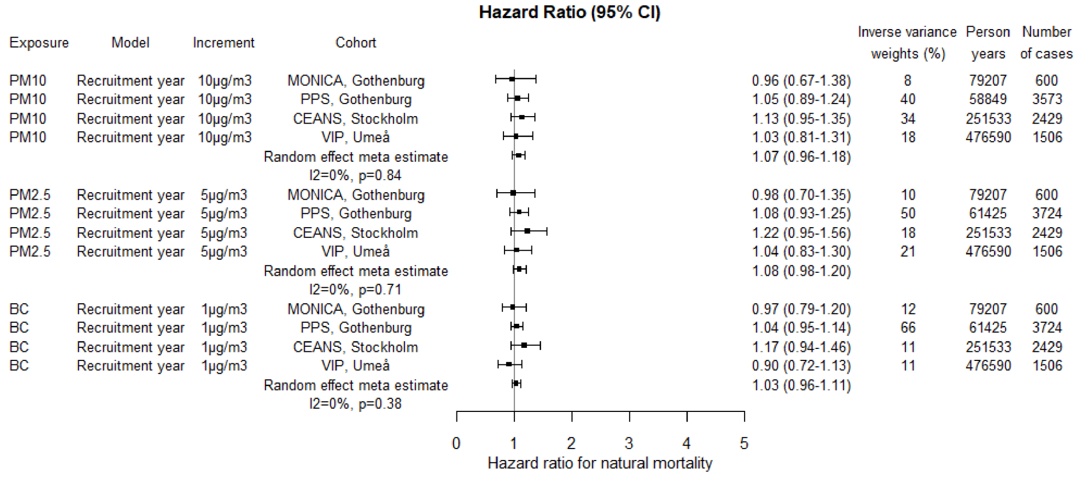

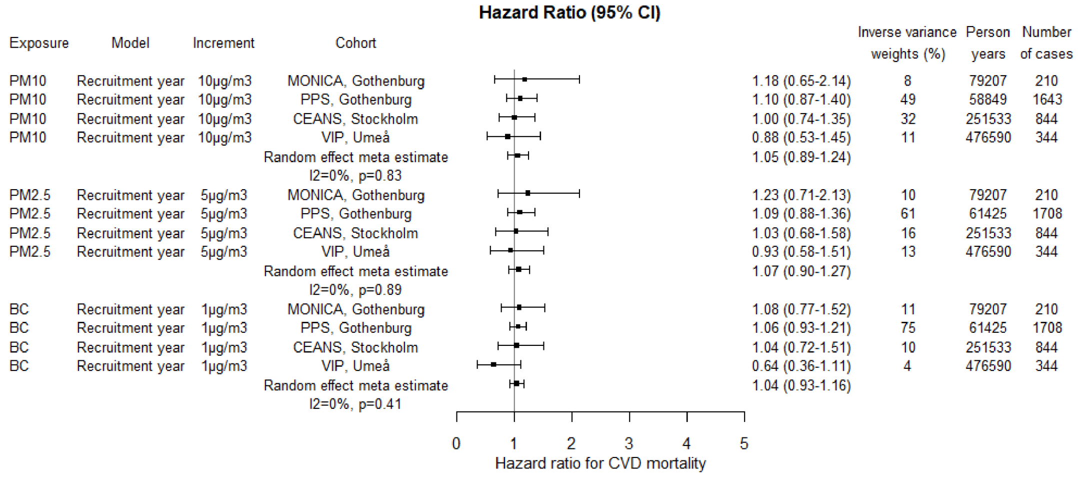

Supplement: Supplementary data [file bmjopen-2020-046040supp001.pdf]
