# Supplementary material for: Long-term exposure to particulate air pollution and black carbon in relation to natural and cause-specific mortality: a multicohort study in Sweden
Source: BMJ Open. 2021 Sep 8;11(9):e046040. doi: 10.1136/bmjopen-2020-046040 (PMC8438896; doi:10.1136/bmjopen-2020-046040)
Supplement: Author's manuscript [file bmjopen-2020-046040.draft_revisions.pdf]

BMJ Open is committed to open peer review. As part of this commitment we make the peer review history of every article we publish publicly available.

When an article is published we post the peer reviewers' comments and the authors' responses online. We also post the versions of the paper that were used during peer review. These are the versions that the peer review comments apply to.

The versions of the paper that follow are the versions that were submitted during the peer review process. They are not the versions of record or the final published versions. They should not be cited or distributed as the published version of this manuscript.

BMJ Open is an open access journal and the full, final, typeset and author-corrected version of record of the manuscript is available on our site with no access controls, subscription charges or pay-per-view fees (http://bmjopen.bmj.com).

If you have any questions on BMJ Open's open peer review process please email info.bmjopen@bmj.com

Long-term exposure to particulate air pollution and black carbon in relation to natural and cause specific mortality

Journal:	*BMJ Open*
Manuscript ID	bmjopen-2020-046040
Article Type:	Original research
Date Submitted by the Author:	19-Oct-2020

Complete List of Authors:	Sommar, Johan; Umea University Department of Public Health and Clinical Medicine, Sustainable health<br>Andersson , Eva M.; Sahlgrenska University Hospital and University of Gothenburg, Occupational and Environmental Medicine<br>Andersson, Niklas; Institute of Environmental Medicine, Karolinska Institutet<br>Sallsten, Gerd; Sahlgrenska University Hospital and University of Gothenburg, Occupational and Environmental Medicine<br>Stockfelt, Leonard; Sahlgrenska University Hospital and University of Gothenburg, Occupational and Environmental Medicine<br>Ljungman, Petter; Karolinska Institutet<br>Segersson, David; SMHI<br>Eneroth, Kristina; SLB-analys, Environment and Health Administration<br>Gidhagen, Lars; SMHI<br>Molnar, Peter; Sahlgrenska University Hospital and University of Gothenburg, Occupational and Environmental Medicine<br>Wennberg, Patrik; Umeå University, Department of Public Health and Clinical Medicine, Family Medicine<br>Rosengren, Annika ; University of Gothenburg Sahlgrenska Academy, Institute of Medicine<br>Rizzuto, Debora; Karolinska Institutet , Aging Research Center, Department of Neurobiology, Health Care Sciences and Society; Stockholm University,<br>Leander, Karin; Karolinska Institutet, Institute of Environmental Medicine<br>Lager, Anton; Karolinska Institutet, Department of Public Health Sciences<br>Magnusson, Patrik; Karolinska Institutet, Dept. Medical Epidemiology and Biostatistics<br>Johansson, Christer; Department of Environmental Science and Analytical Chemistry<br>Barregard, Lars; Sahlgrenska University Hospital and University of Gothenburg, Occupational and Environmental Medicine<br>Bellander, Tom; Karolinska Institutet, Institute of Environmental Medicine; Stockholm County Council, Centre for Occupational and Environmental Medicine<br>Pershagen, Göran; Institute of Environmental Medicine, Karolinska Institutet<br>Forsberg, Bertil; Umeå University, Public Health and Clinical Medicine

Keywords:	EPIDEMIOLOGY, PUBLIC HEALTH, Cardiac Epidemiology < CARDIOLOGY

SCHOLARONE™
Manuscripts

I, the Submitting Author has the right to grant and does grant on behalf of all authors of the Work (as defined in the below author licence), an exclusive licence and/or a non-exclusive licence for contributions from authors who are: i) UK Crown employees; ii) where BMJ has agreed a CC-BY licence shall apply, and/or iii) in accordance with the terms applicable for US Federal Government officers or employees acting as part of their official duties; on a worldwide, perpetual, irrevocable, royalty-free basis to BMJ Publishing Group Ltd ("BMJ") its licensees and where the relevant Journal is co-owned by BMJ to the co-owners of the Journal, to publish the Work in this journal and any other BMJ products and to exploit all rights, as set out in our licence.

The Submitting Author accepts and understands that any supply made under these terms is made by BMJ to the Submitting Author unless you are acting as an employee on behalf of your employer or a postgraduate student of an affiliated institution which is paying any applicable article publishing charge ("APC") for Open Access articles. Where the Submitting Author wishes to make the Work available on an Open Access basis (and intends to pay the relevant APC), the terms of reuse of such Open Access shall be governed by a Creative Commons licence – details of these licences and which Creative Commons licence will apply to this Work are set out in our licence referred to above.

Other than as permitted in any relevant BMJ Author's Self Archiving Policies, I confirm this Work has not been accepted for publication elsewhere, is not being considered for publication elsewhere and does not duplicate material already published. I confirm all authors consent to publication of this Work and authorise the granting of this licence.

For peer review only

Long-term exposure to particulate air pollution and black carbon in relation to natural and cause specific mortality

Johan Nilsson Sommar[1*], Eva M. Andersson[2], Niklas Andersson[3], Gerd Sallsten[2], Leo Stockfelt[2], Petter L. S. Ljungman[3,4], David Segersson[5], Kristina Eneroth[6], Lars Gidhagen[5], Peter Molnar[2], Patrik Wennberg[7], Annika Rosengren[8], Debora Rizzuto[9,10], Karin Leander[11], Anton Lager[12,13], Patrik KE Magnusson[14], Christer Johansson[6,15], Lars Barregard[2], Tom Bellander[3,16], Göran Pershagen[3,16] and Bertil Forsberg[1]

[1] Section of Sustainable health, Department of Public Health and Clinical Medicine, Umeå University, Umeå, Sweden
[2] Occupational and Environmental Medicine, Department of Public Health and Community Medicine, Institute of medicine, Sahlgrenska Academy, University of Gothenburg & Sahlgrenska University Hospital, Sweden.
[3] Institute of Environmental Medicine, Karolinska Institutet, Stockholm, Sweden
[4] Department of Cardiology, Danderyd Hospital, Stockholm, Sweden
[5] Swedish Meteorological and Hydrological Institute, Norrköping, Sweden
[6] SLB-analys, Environment and Health Administration, Stockholm, Sweden
[7] Public Health and Clinical Medicine, Family Medicine, Umeå University, Umeå, Sweden
[8] Department of Molecular and Clinical Medicine, Institute of Medicine, University of Gothenburg, Sahlgrenska University Hospital, Gothenburg, Sweden
[9] Ageing Research Center, Department of Neurobiology, Care Sciences and Society, Karolinska Institutet and Stockholm University, Stockholm, Sweden
[10] Stockholm Gerontology Research Center, Stockholm, Sweden
[11] Institute of Environmental Medicine, Karolinska Institutet, Stockholm, Sweden
[12] Centre for Epidemiology and Community Medicine, Stockholm County Council, Stockholm, Sweden
[13] Department of Public Health Science, Karolinska Institutet, Stockholm, Sweden
[14] Department of Medical Epidemiology and Biostatistics, Karolinska Institutet, Stockholm, Sweden
[15] Department of Environmental Science, Stockholm University, Stockholm, Sweden
[16] Centre for Occupational and Environmental Medicine, Region Stockholm, Stockholm, Sweden

* Corresponding author:

Department of Public Health and Clinical Medicine, Section of Sustainable Health, Umeå University, Umeå, Sweden
Phone: +46 907853453
E-mail: johan.sommar@umu.se

For peer review only - http://bmjopen.bmj.com/site/about/guidelines.xhtml

Abstract

Objectives: To estimate concentration-response relationships for particulate matter (PM) and black carbon (BC) in relation to mortality in cohorts from three Swedish cities with comparatively low pollutant levels.

Setting: Cohorts from Gothenburg, Stockholm and Umeå, Sweden.

Design: High-resolution dispersion models were used to estimate annual mean concentrations of PM with aerodynamic diameter ≤10 µm (PM10) and ≤2.5 µm (PM2.5), and BC, at individual addresses during each year of follow up 1990-2011. Moving averages were calculated for the time windows 1-5 years (lag 1-5) and 6-10 years (lag 6-10) preceding the outcome. Cause-specific mortality data were obtained from the national cause of death registry. Cohort-specific hazard ratios were estimated using Cox regression models and then meta-analysed including a random effect of cohort.

Participants: During the study period, 7340 cases of natural mortality, 2755 cases of cardiovascular disease (CVD) mortality and 817 cases of respiratory and lung cancer mortality were observed among in total 68679 individuals and 689813 person-years of follow-up.

Results: Both PM10 (range: 6.3–41.9 µg/m^3) and BC (range: 0.2-6.8 µg/m^3) were associated with natural mortality showing 17% (95% CI 6-31%) and 9% (95% CI 0-18%) increased risks per 10 µg/m^3 and 1 µg/m^3 of lag1-5 exposure, respectively. For PM2.5 (range: 4.0–22.4 µg/m^3) the estimated increase was 13% per 5 µg/m^3, but less precise (95% CI -9-40%). Estimates for CVD mortality appeared higher for both PM10 and PM2.5. No association was observed with respiratory mortality.

Conclusion: The results support an effect of long-term air pollution on natural mortality and mortality in CVD with high relative risks also at low exposure levels. These findings are relevant for future decisions concerning air quality policies.

Key words: air pollution, particulate matter, black carbon, natural mortality, cardiovascular mortality

For peer review only - http://bmjopen.bmj.com/site/about/guidelines.xhtml

Strengths and limitations of this study

Few previous studies have followed up individuals by home address and time varying exposures as annual mean concentrations at actual place of residence

High-resolution dispersion models of particle concentrations were used to capture local contrasts in exposure

The study did however not include information on noise exposure and green space near the home address

This study showed associations with mortality within populations with comparatively low particle concentrations, generally below EU standards

As the first (multi)-cohort study using high resolution dispersion modelled particle concentrations at mean levels below WHO guidelines, this study found high relative risks of mortality

For peer review only - http://bmjopen.bmj.com/site/about/guidelines.xhtml

Introduction

Particulate matter ambient air pollution exposure has been associated with increased risk of premature mortality in several cohort studies [1 2]. According to the Global Burden of Diseases Study 2017 [3], assessing 84 risk factors for premature mortality in 195 countries, ambient particulate matter air pollution was ranked as the tenth most important risk factor for mortality and the most important environmental risk factor. Using risk functions restricted to cohort studies of outdoor air pollution, ambient particle matter pollution was estimated to cause 2.9 million (95% confidence interval (CI): 2.5-3.4) deaths in year 2017.

Studies using NO2 and NOx as indicators of traffic related air pollution have also reported associations with increased risk of premature mortality [2 4]. Even though there could be a nitrogen dioxide associated short-term effect on mortality independent of particle matter (e.g. Mills et al., 2016 [5]), the evidence for long-term effects is weaker and exposure to particles is considered as the primary causal determinant of health effects [6].

In a review of 11 cohort studies on long-term air pollution exposure and mortality, ambient concentrations of PM2.5 were associated with increased risk of all-cause mortality and particularly cardiovascular mortality. However, the heterogeneity between study relative risk estimates was large [2]. The large European Study of Cohorts for Air Pollution Effects (ESCAPE) project including 22 cohorts, observed an increased risk of overall mortality associated with ambient PM2.5 concentrations [7] whereas no association was found with cardiovascular mortality [8]. The observed risk increase associated with PM2.5 did, nevertheless, not reach statistical significance. On the other hand, a recent study in a large Danish cohort found statistically significant increased risks for both natural cause and CVD mortality associated with PM2.5 exposure during the last 15 years [9]. Neither the Danish study nor the ESCAPE study were able to demonstrate associations with respiratory mortality [9 10].

Black carbon (BC), one component of PM, has been hypothesized to play a key role in the adverse health effects of air pollution exposure [11]. The review by Luben et al. identified three studies on the long-term effect of BC (or elemental carbon) and mortality in coronary heart disease [12] and ischemic heart disease [13 14]. However, the review did not find support for a stronger association with mortality for BC compared with PM2.5. Elemental carbon has been associated with all-cause mortality in another meta-analysis [2]. Within the ESCAPE project, PM2.5-absorbance was used as a proxy for BC. The estimated increased risk of natural mortality associated with PM2.5-absorbance did not reach statistical significance [7]. In Denmark (areas of Copenhagen and Aarhus), BC was, however, associated with both all-cause and cardiovascular mortality [9].

These previous studies varied in the method used for the exposure assessment of particle concentrations. Earlier studies assessed concentrations using centrally located monitoring stations, mostly representing the urban background [15]. Later studies use air pollution monitoring together with data on land use (land use regression, LUR) or detailed data on emissions and meteorological conditions (dispersion models). Most studies on long-term air pollution effects have assessed outdoor residential exposures at the year of inclusion into the cohort, a recent study on mortality however used annual mean concentrations on addresses during follow-up [9]. Studies using exposure assessments with a higher precision and in low-level exposure environments demonstrated higher relative risks of mortality in a recent meta-analysis [16]. Studies on low level air pollution are few, however. Adding to the knowledge from the long-term air pollution effects on mortality from the Canadian Community Health Survey-Mortality cohort [17-19] and a Medicare population in New England [20], this study is of importance for policy making and determination of limit values. Compared to these

For peer review only - http://bmjopen.bmj.com/site/about/guidelines.xhtml

previous studies that used a 1x1 km^2 resolution for the assessment of air pollutant concentrations this study will make use of high-resolution 50x50 m^2 dispersion modelled particulate matter able to capture also local exposure contrasts.

Aim

This study aims to use high-resolution dispersion modelled annual mean residential PM10, PM2.5 and BC concentrations to estimate associations with natural and cause specific mortality in a northern European environment characterized by annual averages largely compliant with EU air quality standards.

Methods and Materials

Study Cohorts

The study included four cohorts; two cohorts from Gothenburg, one from Stockholm (pooled from four cohorts) and one cohort from Umeå. Our study period ranged from January 1st 1990 to December 31st 2011 (December 31st 2013 for Umeå participants residing at the same address as in 2011). Residential address history was obtained for all cohort participants through linkage using personal identification numbers to mandatory records of residential addresses at Statistics Sweden or the Swedish Taxation Authority. These residential addresses were then geocoded by matching against the Swedish Mapping Cadastral and Land Registration Authority databases. When needed, addresses were manually checked and corrected for inconsistencies and assigned geographical coordinates.

From Gothenburg, two general population cohorts were included: The Primary Prevention Study (PPS) cohort and the GOT-MONICA cohort. The PPS cohort recruited a random third of all men in Gothenburg born between the years 1915 and 1925 who were examined in 1970-73 to study predictors of cardiovascular disease [21]. The GOT-MONICA cohort is one of the cohorts within the international MONICA project (“Multinational Monitoring of Trends and Determinants in Cardiovascular Diseases”), designed to study risk factors for cardiovascular diseases [22 23]. GOT-MONICA included residents aged 25-64 years. Recruitments were conducted in 1985, 1990 and 1995. For both cohorts, background data and information on cardiovascular risk factors were recorded by questionnaires. The recruited individuals were also examined by health care professionals.

From Stockholm County the Cardiovascular Effects of Air pollution and Noise Study (CEANS) cohort was included [24]. The cohort consists of four sub-cohorts. The Stockholm Diabetes Prevention Program [SDPP] was designed as a population-based prospective cohort, and includes 3128 men recruited between the years 1992 and 1994 and 4821 women recruited between the years 1996 and 1998. The program recruited individuals aged 35-56 years that had not previously been diagnosed with diabetes. About half of the recruited had a first or second degree relative with a history of diabetes. These were then matched with recruits of the same age and sex, who did not have a relative with a diabetes history. The second sub-cohort named SIXTY consists of a population-based random sample of 60-year old men and women (N=4232) living within Stockholm County between August 1997 and March 1999. The third cohort consists of the Screening Across the Lifespan Twin study [SALT], including twins born before year 1958. Only participants residing in Stockholm County at recruitment were included in the current analysis (N=7043). The fourth sub-cohort, the Swedish National Study on Aging and Care in Kungsholmen [SNAC-K], included a random sample of inhabitants aged at least 60 years and residing in a central area of Stockholm City (N=3363). Sub-cohort specific characteristics of included individuals in CEANS have previously been presented [25].

For peer review only - http://bmjopen.bmj.com/site/about/guidelines.xhtml

Including Umeå municipality, the Västerbotten intervention program (VIP) is a program where the population in the County is invited to a health examination the year they turn 40, 50 and 60 (and during some years also 30) years old [26]. The program was initiated in parts of the county in 1985 as a community and individual level program to reduce the morbidity and mortality from cardiovascular disease in Northern Sweden. The interview questions provide information about cardiovascular risk factors as well as social situation, education, diet and physical activity. So far more than 100 000 individuals have participated in the program.

The study was reviewed and approved by the Ethical Review Boards of Gothenburg (references T800-08 and T547- 13), Stockholm (reference 2018/2064-32), and Umeå (reference 2015/16-31Ö).

The public was not involved in the design, or conduct, or reporting, or dissemination plans of the research.

Mortality Outcomes

Cause-specific mortality was determined by linkage of national personal identification numbers to the Cause of Death Register at the Swedish National Board of Health and Welfare. We used the International Code of Diseases (ICD)9 001-779 or ICD-10 A00-R99 to define deaths by natural causes, ICD-9 400-440 or ICD-10 I10-I70 to define deaths in CVD, ICD9 162 or ICD10 C34 to define lung cancer deaths, and ICD9 460-519 or ICD10 J0-99 to define non-malignant respiratory mortality. In addition, deaths by other causes was studied (that is other than CVD, lung cancer, respiratory disease, and external causes).

Exposure Assessment

An in depth description of the dispersion model used for the exposure assessment has previously been published [27]. Both regional and local emission inventories for the years 1990, 2000 and 2011 in Gothenburg and Umeå, and years 1990, 1995, 2000, 2005 and 2011 in Stockholm, were used as input to Gaussian dispersion model simulations of annual mean concentrations of particulate matter (PM), i.e. PM10, PM2.5 and BC. To allow high resolution in vicinity of roads, a quadtree receptor grid was used, resulting in a resolution down to 35 m x 35 m. For inner-city streets with buildings on one or both sides, an additional concentration component was simulated with the Danish operational street pollution model (OSPM) [28].

Monitoring data were used in Stockholm to adjust the modelled concentrations to meteorological year-to-year variability. In Gothenburg and Umeå interpolation was used between the years and each year was also adjusted by a ventilation factor taking meteorological variability into account. Emission factors for traffic PM-exhaust for different vehicle types, speeds and driving conditions were calculated based on the Handbook on Emission Factors for Road Traffic version 3.1 [29], whereas emission factors for black carbon were based on TRANSPHORM [30]. The non-exhaust emissions of PM consist mainly of road wear particles, with a minor contribution from brake and tire wear [31-33]. For small-scale residential heating, emissions in Gothenburg and Stockholm were determined using household energy consumption data from Statistics Sweden with a resolution of 100 m x 100 m. This was based on proxy data including number of stoves or boilers in each municipality, living space of small houses per square km, population density per 100 m^2 and availability of district heating. In Umeå a detailed inventory of individual stoves and boilers was used with data from chimney sweepers and interviews about amount of wood burning [27]. Industrial and energy production facilities were included as point sources in the model. Emission from shipping were also included using a method described by Jalkanen et al. [34].

For peer review only - http://bmjopen.bmj.com/site/about/guidelines.xhtml

To model the long-range transport of PM10, PM2.5 and BC the difference between total concentrations measured at monitoring stations and modelled local particle concentrations at the same location was considered on an annual basis (Gothenburg and Umeå). In Stockholm annual concentrations from a regional background station was used to estimate the long-range transport. The long-range transport contributions were estimated differently for each city, but within each city they had a purely temporal contrast. This was assumed since the spatial variation between regional background sites has been shown to be small [35]. These yearly contributions of background concentrations were then added to the estimated local concentrations. In a previous study, validations of total concentrations against measurements showed R2 values of 0.87, 0.65 and 0.93 for PM10, PM2.5, and BC, respectively [27].

The resulting concentrations were lastly added to each study participant's home addresses and annual exposures estimated accounting for any changes in address during the study period 1990-2011.

Confounders

Cohort-specific models were adjusted for potential confounding by including sex, calendar year, sub-cohort (in Stockholm), smoking status (current, former, never smoker), alcohol consumption (in Stockholm and Umeå; daily, weekly, seldom, never), physical activity (in Gothenburg and Umeå: sedentary, moderate, intermediate or vigorous; in Stockholm: once a month or less/>1 hour per week, about once a month/1 hour per week, 3 times a week or more/>2 hour per week), marital status (single, married or living with partner, no answer), socio-economic index by occupation (in Gothenburg (PPS) and Stockholm: blue collar, low and intermediate white collar and self-employed, high level white collar and self-employed professionals with academic degrees, no answer), education level (in Gothenburg (MONICA), Stockholm and Umeå: primary school or less, up to secondary school or equivalent, university degree or more, no answer), occupation status (in Stockholm and Umeå: gainfully employed, unemployed/not gainfully employed, retired, no answer). Area level socioeconomic status was estimated for each cohort member's residence from the mean neighborhood individual income in persons of working age by Small Areas for Market Statistics (SAMS) provided by Statistics Sweden for the calendar year 1994.

Statistical methods

Cox-proportional hazard models were used to estimate hazard ratios of mortality associated with PM and BC in cohort-specific analyses. Age was used as the underlying time variable for the baseline hazard. The regression model included adjustment for calendar year, baseline information on confounders as well as area level socio-economy. Since the Stockholm CEANS cohort consisted of four sub-cohorts that differed in age and time of recruitment, separate baseline hazards were used for the analyses within this cohort. We censured individuals at death by other causes, the end of the study period or time of permanent emigration from the study areas. Associations with PM and BC were assessed using two exposure windows; a moving average over 1-5 years preceding the event (lag1-5) and 6-10 years preceding the event (lag6-10). For inclusion, annual mean concentrations were required for at least 80% of the time window. Besides these time windows, associations were also assessed with concentrations at the year of recruitment. Hazard ratios and 95% confidence intervals (CI) were expressed per 10, 5 and 1 μg/m^3 for PM10, PM2.5 and BC, respectively, as well as per interquartile ranges (IQRs) across the three study areas.

Meta-analyses of cohort-specific estimates were performed using random effects [36]. Heterogeneity between cohort estimates were assessed by the I2 statistic and statistically tested for deviations from homogeneity by a Chi-square test based on the Cochran's Q statistic.

For peer review only - http://bmjopen.bmj.com/site/about/guidelines.xhtml

Results

Participant characteristics

The total number of individuals included in our meta-analysis was 68,679, with the number of individuals included in each cohort presented in table 1. Total number of deaths by natural causes was 7344. Age at recruitment differed largely between cohorts with the highest median in the Gothenburg PPS cohort and lowest in VIP Umeå, 69 compared with 40 years. By design only males were recruited to the Gothenburg PPS cohort, whereas the other three cohorts had a slightly larger proportion of women compared with men. Baseline current smoking was highest in the Gothenburg PPS cohort and lowest in Stockholm CEANS and VIP Umeå, 39% compared with about 20%. Leisure time physical activity had a different categorization within the Stockholm CEANS cohort but it can be concluded that there was a larger proportion of individuals reporting a sedentary lifestyle at baseline in this cohort. The cohorts in Gothenburg had the largest proportion of physically active individuals, with higher frequency and intensity. Alcohol consumption was more frequent in Stockholm CEANS compared with VIP Umeå. University education was most common in Stockholm CEANS and VIP Umeå (above 30%) and less common in Gothenburg. A larger proportion of individuals were gainfully employed in the younger VIP Umeå than in Stockholm CEANS, 84% compared with 66%. Blue-collar occupation was more likely within the Gothenburg PPS cohort compared with Stockholm CEANS.

Particle concentrations

Particle concentrations of PM10, PM2.5 and BC all had the highest mean and IQR in the Gothenburg cohorts and the lowest in VIP Umeå (figure 1). In total, means of lag1-5 PM10, PM2.5 and BC were; 11.3, 6.9 and 0.6 μg/m^3, respectively. Lag1-5 concentrations were on average lower than lag6-10 concentrations due to a decreasing trend in concentrations during the study period.

Associations with mortality

Meta-estimates showed increased risks associated with PM10, PM2.5 and BC in relation to natural mortality; for lag1-5 17% (95% CI 6-31) increased risk per 10 μg/m^3 PM10, 13% (95% CI -9-40) increased risk per 5 μg/m^3 PM2.5 and 9% (95% CI 0-18) increased risk per 1 μg/m^3 BC (figure 2a-c; crude estimates in supplementary material figure S1a-c). The combined risk increase associated with PM2.5 did however not reach statistical significance. For PM10 and PM2.5 higher risk estimates were found for lag1-5 concentrations compared with lag6-10, whereas for BC, the risk estimates were the same. Increased risks were estimated for all cohorts except for Gothenburg MONICA, where the hazard ratio estimate for natural mortality was below 1.

The risk estimates for CVD mortality associated with PM10 and PM2.5 appeared higher compared with natural mortality; 19 (95% CI 1-40) and 23% (95% CI 3-48) increased risks for lag1-5, per 10 and 5 μg/m^3, respectively (figure 3a-b; crude estimates in supplementary material figure S2a-b). The increased risks associated with BC seemed lower for CVD mortality than for natural mortality for lag1-5, but similar for lag6-10 (figure 3c; crude estimates in supplementary material figure S2c).

The HRs per IQR for natural mortality were higher for PM2.5 and PM10 than for BC; 6, 5 and 2% increased risk, respectively. The same order per IQR was found for CVD mortality; 10, 5 and 1% for PM2.5, PM10 and BC, respectively.

No associations between PM and lung cancer or non-malignant respiratory mortality were observed (supplementary material figure S3-4a-c). Increased risk was, however, observed for mortality by other causes (that is other than CVD, lung cancer, respiratory disease, and external causes) with the

For peer review only - http://bmjopen.bmj.com/site/about/guidelines.xhtml

same magnitude as observed for natural and CVD mortality, but these reached statistical significance only for the association with BC (supplementary material figure S5a-c).

For comparison with previous studies, associations were also assessed with residential PM concentrations for the recruitment year. These estimates were in general lower compared with those with lag1-5 and lag6-10 concentrations (supplementary material figure S6a-b). Estimated risk increases for natural mortality were 7 (95% CI -4-18), 8 (95% CI -2-20) and 3% (95% CI -4-11) in association with PM10, PM2.5 and BC, per 10, 5 and 1 µg/m^3, respectively.

The heterogeneity between cohort hazard ratio estimates in relation to precision was in general very low. Heterogeneity was, however, observed for associations with lag1-5 PM2.5 and lag6-10 BC in relation to natural mortality, but did not reach statistical significance (figure 2-3a-c).

Discussion

We observed in four Swedish cohorts located in Gothenburg, Stockholm and Umeå increased risks associated with long-term residential outdoor levels of PM10, PM2.5 and BC in relation to natural and CVD mortality. PM10 was most consistently associated with natural and CVD mortality, whereas PM2.5 and BC demonstrated positive associations for both outcomes but reached statistical significance only for CVD and natural mortality, respectively. Risk estimates associated with PM10 and PM2.5 appeared larger for exposures during the last 5 years in comparison to 6-10 years prior. For BC the associated risks were the same for both exposure windows in relation to natural mortality but higher for the exposure window 6-10 years prior in relation to CVD mortality. Within a previous meta-analysis of these cohorts, an association was found between BC and stroke incidence [25]. No association was, however, found between PM10 or PM2.5 and incidence in ischemic heart disease.

The observed 17% increased risk per 10 µg/m^3 PM10 in relation to natural mortality is high compared with most studies (e.g. the 6% increased risk found by Hoek et al. 2013 [2]) but similar to the recent findings within a Danish cohort presenting 12% increased risk (95% CI 3-22%) associated with a 15-year moving average of residential ambient concentrations [9]. Their estimate for CVD mortality was 30% (95% CI 11-53%) which was higher than our meta-estimate of 19%. For PM2.5 our observed risk increase by 13% per 5 µg/m^3 for natural mortality is also in agreement with the Danish estimate of 13% (95 CI 5-21%). As for PM10 the risk increase for CVD mortality associated with PM2.5 was again lower in our study, 23% compared to 29% (95% CI 13-47%). The average PM10 and PM2.5 exposure levels were 2.2 and 2.6 times higher in the Danish cohort compared to our study (lag 1-10 time-weighted concentrations from the Danish cohort compared with lag 1-5 moving averages in our study).

Using less detailed assessments of outdoor residential exposures, a meta-analysis of 22 cohorts within the ESCAPE project reported a borderline significant 4% (95% CI 0-9%) increased risk of natural-cause mortality for a 10 µg/m^3 difference in annual PM10 at the year of recruitment [7]. No association with PM10 was, however, observed for deaths in ischemic heart disease or myocardial infarction, whereas a suggested association was found in relation to deaths in cerebrovascular disease; 22% (95% CI -9-63%) increased risk per 10 µg/m^3 [8]. The findings for mortality associated with PM2.5 have been heterogeneous, however, the meta-estimate among the ESCAPE cohorts showed a 7% (95% CI 2-13%) increased risk per 5 µg/m^3 of natural mortality and 21% (95% CI -13-69%) increased risk of death in cerebrovascular disease. As for PM10 no association was found in relation to death in ischemic heart disease or myocardial infarction. Within earlier studies; a review and meta-analysis by Hoek et al. (2013) found 6% (95% CI 4-8%) and 15% (95% CI 4-27) increased risks of all-cause mortality and cardiovascular mortality per 5 µg/m^3, respectively. A stronger association between PM and mortality with cardiovascular causes compared to all natural mortality has also

For peer review only - http://bmjopen.bmj.com/site/about/guidelines.xhtml

been reported by others [1], and is in agreement with our findings on CVD mortality; and most apparent in relation to exposures during the last 5 years.

In accordance with our meta-estimate for BC the Danish cohort reported a risk increase by 9% (95% CI 4-15%) per 1 µg/m3 for all-cause mortality [9]. An increased risk by 16% (95% CI 5-27%) was also observed for CVD mortality, higher than the 5% increased risk found in our study. In the review by Hoek et al. (2013) elemental carbon was found associated with a 6% (95% CI 5-7%) increased risk of all-cause mortality per 1 µg/m^3.

The lack of association between PM and non-malignant respiratory mortality in our study is in agreement with results from both the Danish study [9], the ESCAPE study [10] and a large cohort study in Rome [37]. Previous studies have, however, found an increased risk of lung cancer incidence associated with both PM10 and PM2.5 [38 39]. The statistical power for these outcomes was low in our meta-analyses due to the limited number of deaths in lung cancer and other respiratory causes.

The observed heterogeneity of PM associated risk for premature mortality between studies is possibly related to differences in particle composition, housing with differences in particle infiltration, population characteristics, and the minor differences in confounding adjustments. The air pollution dispersion model used for the exposure assessment in our study has a high spatial resolution, and for the follow up period it was also updated with new regional and local emission inventories as well as PM measurements to provide annual mean temporal PM concentrations. A higher precision in the exposure estimate have been found to produce higher relative health risk estimates [16]. The mortality risk associated with PM2.5 has recently been reported to be higher for local sources compared with distant sources, but with less precision in relative risk estimates, indicating the importance of high spatial resolution exposure data to successfully model differences in mortality [40]. The meta-analysis results in this study and the results presented by Hvidtfeldt et al. (2019) both generally provided higher hazard ratio estimates compared with previous mortality studies. Both studies also used moving averages based on annual mean concentrations at the place of residence. Besides exposure data with a high precision such a moving average over relevant time windows may be advantageous compared with residential concentrations at the year of recruitment or a time-weighted average exposure during the follow-up. These two studies also adjusted both for individual data on confounders and neighbourhood level data on socioeconomics.

Strengths and limitations

The strength of this study includes the state-of-the-art air pollution modelling used for exposure assessment. Several factors that affect the local dispersion of air pollution particles were considered including meteorological conditions, amount of traffic including vehicle types and speed, which affect exhaust and wear emissions, the width of the street, and the height of neighbouring building. This facilitates models with a high spatial resolution providing the ability to estimate an individual's exposure at the residential address. Temporal differences for each address were also accounted for by repeatedly updating the exposure model during the period of follow up. Our modelled concentrations have showed a good agreement when validated against measured concentrations by monitoring stations in city centres [27]. Due to uncertainties in data input to the dispersion model, and since modelled PM levels at the home address do not equal true personal exposure, exposure misclassifications at the individual level will nevertheless arise. True personal exposure would for instance include indoor particle concentrations at the place of residence, at the workplace, and during commuting. During the years of follow up there has been a decreasing trend in both total concentrations of PM and age specific mortality. It was therefore important to adjust for calendar

For peer review only - http://bmjopen.bmj.com/site/about/guidelines.xhtml

year in the Cox models. Limitations also include the lack of information on noise exposure and green space near the home address which could be confounders or effect-modifiers of air pollution effects.

Another strength of this study is the large number of cohort participants and ability to adjust for a large set of confounders at both individual and area level, however information on confounders from questionnaires were only available at baseline. Even though we expect the cohort participants with age ranging from 40 years to have fairly stable lifestyles and habits, such baseline variables may change, for example due to disease. No clear heterogeneity in associations between cohorts was observed, however, with only four cohorts the test had low statistical power to reject the null hypothesis of homogeneity.

This study additionally contributes with hazard ratio estimates between PM and mortality in the lower range of exposure with PM2.5 ranging between 4.0 to 20.4 µg/m^3 and PM10 ranging between 6.3 to 41.9 µg/m^3. Even though this is lower than the current European Union standards of 25 µg/m^3 for PM2.5 and (with a few exceptions) lower than 40 µg/m^3 for PM10, the study found high relative risks for premature mortality.

Conclusions

This study of four cohorts with relatively low exposures showed increased risks of mortality in association with long-term exposure to PM10, PM2.5 and BC, and thus strongly support long-term air pollution associated effects. Since high risk increases were observed even at relatively low exposures, the findings are relevant for future decisions concerning air quality policies.

For peer review only - http://bmjopen.bmj.com/site/about/guidelines.xhtml

Tables

Table 1. Descriptive statistics of the study participants in each cohort

		Gothenburg, MONICA	Gothenburg, PPS	Stockholm, CEANS	Umeå, VIP
Participants (n)		4,500	5,850	22,314	42,580
Baseline data collection (calendar years)		1985,1990,1995	1970-74	1992-2004	1992-2014
Age at enrolment (years; median (range))		46 (25–66)	51 (47–56)	56 (35-104)	40 (40-50)
Women (%)		52	0	58	52
Smoking status (%)	Current smoker	29	39	22	19
	Former smoker	N/A	33	36	30
	Never smoker	65	27	40	49
	Missing data	5	0	1.8	1
Physical activity (%)	Once a month or less/<1h/week	N/A	N/A	61	N/A
	About once a month/~1h/week	N/A	N/A	26	N/A
	3 times a week or more/>2h/week	N/A	N/A	7.6	N/A
	Missing data	N/A	N/A	4.6	N/A
Leisure time physical activity (%)	Sedentary	18	24	N/A	36
	Moderate	62	59	N/A	42
	Intermediate and vigorous	18	17	N/A	22
	Missing data	2	1	N/A	2
Alcohol consumption (%)	Daily	N/A	N/A	6.7	2
	Weekly	N/A	N/A	55	16
	Seldom	N/A	N/A	31	42
	Never	N/A	N/A	5.6	1
	Missing data	N/A	N/A	1.8	39
Married/living with partner (%)	No	21	14	29	23
	Yes	47	86	70	77
	Missing data	32	<0.1	1.3	1
Education level (%)	Primary school or less	13	N/A	30	30
	Up to secondary school or equivalent	32[a]	N/A	36	30
	University degree & more	20	N/A	31	40
	Missing data	35	N/A	2.8	1

For peer review only

Occupation (%)	Gainfully employed	N/A	N/A	66	85
	Unemployed/not gainfully empolyed	N/A	N/A	5.8	6
	Retired	N/A	N/A	27	4
	Missing data	N/A	N/A	1.3	4
Socioeconomic index by occupation (%)	Blue collar	N/A	48	27	N/A
	Low-and intermediate white collar and self-employed	N/A	23	51	N/A
	High level white collar and self-employed professional with academic degrees	N/A	30	18	N/A
	Missing data	N/A	0	4.4	N/A
Mean income by SAMS 1994 (SEK)		154,780	148,602	N/A	130,000
Mean income by SAMS 2009 (SEK)		N/A	N/A	303,910	N/A

CEANS, Cardiovascular Effects of Air Pollution and Noise Study; MONICA, Multinational Monitoring of Trends and Determinants in Cardiovascular Diseases; N/A, not available; PPS, Primary Prevention Study; SAMS, Small Areas for Market Statistics; SEK, Swedish Krona; VIP, Västerbotten Intervention Programme.

a. Includes technical training

For peer review only

Legends to figures

Figure 1a-c. Boxplot and density function of a) PM10, b) PM2.5 and c) BC concentrations, in total and separate for each cohort. Tabulated values are quartile limits together with means and standard deviations.

Figure 2a-c. Adjusted hazard ratios of natural mortality associated with a) PM10, b) PM2.5 and c) BC within each cohort and by random effect meta-analyses estimates. Exposures were assessed by moving average residential concentrations within the exposure windows last 5 years and 6 to 10 years prior.

Figure 3a-c. Adjusted hazard ratios of CVD mortality associated with a) PM10, b) PM2.5 and c) BC within each cohort and by random effect meta-analyses estimates. Exposures were assessed by moving average residential concentrations within the exposure windows last 5 years and 6 to 10 years prior.

For peer review only

References

1. Brook RD, Rajagopalan S, Pope CA, 3rd, et al. Particulate matter air pollution and cardiovascular disease: An update to the scientific statement from the American Heart Association. *Circulation* 2010;121(21):2331-78. doi: 10.1161/CIR.0b013e3181dbece1
2. Hoek G, Krishnan RM, Beelen R, et al. Long-term air pollution exposure and cardio- respiratory mortality: a review. *Environ Health-Glob* 2013;12 doi: Artn 43 10.1186/1476-069x-12-43
3. Stanaway JD, Afshin A, Gakidou E, et al. Global, regional, and national comparative risk assessment of 84 behavioural, environmental and occupational, and metabolic risks or clusters of risks for 195 countries and territories, 1990–2017: a systematic analysis for the Global Burden of Disease Study 2017. *The Lancet* 2018;392(10159):1923-94. doi: 10.1016/S0140-6736(18)32225-6
4. Atkinson RW, Butland BK, Anderson HR, et al. Long-term Concentrations of Nitrogen Dioxide and Mortality: A Meta-analysis of Cohort Studies. *Epidemiology* 2018;29(4):460-72. doi: 10.1097/ede.0000000000000847 [published Online First: 2018/05/11]
5. Mills IC, Atkinson RW, Anderson HR, et al. Distinguishing the associations between daily mortality and hospital admissions and nitrogen dioxide from those of particulate matter: a systematic review and meta-analysis. *BMJ Open* 2016;6(7):e010751. doi: 10.1136/bmjopen-2015-010751
6. WHO R. Review of evidence on health aspects of air pollution - REVIHAAP Project: Technical Report. Copenhagen2013.
7. Beelen R, Raaschou-Nielsen O, Stafoggia M, et al. Effects of long-term exposure to air pollution on natural-cause mortality: an analysis of 22 European cohorts within the multicentre ESCAPE project. *Lancet* 2014;383(9919):785-95. doi: 10.1016/S0140-6736(13)62158-3
8. Beelen R, Stafoggia M, Raaschou-Nielsen O, et al. Long-term exposure to air pollution and cardiovascular mortality: an analysis of 22 European cohorts. *Epidemiology* 2014;25(3):368-78. doi: 10.1097/EDE.0000000000000076
9. Hvidtfeldt UA, Sorensen M, Geels C, et al. Long-term residential exposure to PM2.5, PM10, black carbon, NO2, and ozone and mortality in a Danish cohort. *Environment international* 2019;123:265-72. doi: 10.1016/j.envint.2018.12.010
10. Dimakopoulou K, Samoli E, Beelen R, et al. Air Pollution and Nonmalignant Respiratory Mortality in 16 Cohorts within the ESCAPE Project. *American journal of respiratory and critical care medicine* 2014;189(6):684-96. doi: 10.1164/rccm.201310-1777OC
11. Luben TJ, Nichols JL, Dutton SJ, et al. A systematic review of cardiovascular emergency department visits, hospital admissions and mortality associated with ambient black carbon. *Environment international* 2017;107:154-62. doi: 10.1016/j.envint.2017.07.005
12. Gan WQ, Koehoorn M, Davies HW, et al. Long-term exposure to traffic-related air pollution and the risk of coronary heart disease hospitalization and mortality. *Environmental health perspectives* 2011;119(4):501-7. doi: 10.1289/ehp.1002511
13. Ostro B, Hu JL, Goldberg D, et al. Associations of Mortality with Long-Term Exposures to Fine and Ultrafine Particles, Species and Sources: Results from the California Teachers Study Cohort. *Environmental health perspectives* 2015;123(6):549-56. doi: 10.1289/ehp.1408565
14. Ostro B, Lipsett M, Reynolds P, et al. Long-Term Exposure to Constituents of Fine Particulate Air Pollution and Mortality: Results from the California Teachers Study. *Environmental health perspectives* 2010;118(3):363-69. doi: 10.1289/ehp.0901181
15. Baxter LK, Dionisio KL, Burke J, et al. Exposure prediction approaches used in air pollution epidemiology studies: Key findings and future recommendations. *J Expo Sci Env Epid* 2013;23(6):654-59. doi: 10.1038/jes.2013.62
16. Vodonos A, Awad YA, Schwartz J. The concentration-response between long-term PM2.5 exposure and mortality; A meta-regression approach. *Environmental research* 2018;166:677-89. doi: https://doi.org/10.1016/j.envres.2018.06.021

For peer review only - http://bmjopen.bmj.com/site/about/guidelines.xhtml

17. Christidis T, Erickson AC, Pappin AJ, et al. Low concentrations of fine particle air pollution and mortality in the Canadian Community Health Survey cohort. *Environmental health : a global access science source* 2019;18(1):84-84. doi: 10.1186/s12940-019-0518-y
18. Pinault LL, Weichenthal S, Crouse DL, et al. Associations between fine particulate matter and mortality in the 2001 Canadian Census Health and Environment Cohort. *Environmental research* 2017;159:406-15. doi: 10.1016/j.envres.2017.08.037 [published Online First: 2017/08/30]
19. Pinault L, Tjepkema M, Crouse DL, et al. Risk estimates of mortality attributed to low concentrations of ambient fine particulate matter in the Canadian community health survey cohort. *Environmental health : a global access science source* 2016;15:18-18. doi: 10.1186/s12940-016-0111-6
20. Shi L, Zanobetti A, Kloog I, et al. Low-Concentration PM2.5 and Mortality: Estimating Acute and Chronic Effects in a Population-Based Study. *Environmental health perspectives* 2016;124(1):46-52. doi: 10.1289/ehp.1409111 [published Online First: 2015/06/03]
21. WILHELMSEN L, BERGLUND G, ELMFELDT D, et al. The multifactor primary prevention trial in Göteborg, Sweden. *European heart journal* 1986;7(4):279-88. doi: 10.1093/oxfordjournals.eurheartj.a062065
22. WILHELMSEN L, JOHANSSON S, ROSENGREN A, et al. Risk factors for cardiovascular disease during the period 1985–1995 in Göteborg, Sweden. The GOT-MONICA Project. *Journal of Internal Medicine* 1997;242(3):199-211. doi: 10.1046/j.1365-2796.1997.00163.x
23. WHO Monica Project Principal Investigators. The world health organization monica project (monitoring trends and determinants in cardiovascular disease): A major international collaboration. *Journal of Clinical Epidemiology* 1988;41(2):105-14. doi: https://doi.org/10.1016/0895-4356(88)90084-4
24. Korek MJ, Bellander TD, Lind T, et al. Traffic-related air pollution exposure and incidence of stroke in four cohorts from Stockholm. *Journal of exposure science & environmental epidemiology* 2015;25(5):517-23. doi: 10.1038/jes.2015.22
25. Ljungman PLS, Andersson N, Stockfelt L, et al. Long-Term Exposure to Particulate Air Pollution, Black Carbon, and Their Source Components in Relation to Ischemic Heart Disease and Stroke. *Environmental health perspectives* 2019;127(10):107012. doi: 10.1289/EHP4757
26. Norberg M, Wall S, Boman K, et al. The Västerbotten Intervention Programme: background, design and implications. *Global health action* 2010;3 doi: 10.3402/gha.v3i0.4643 [published Online First: 2010/03/27]
27. Segersson D, Eneroth K, Gidhagen L, et al. Health Impact of PM10, PM2.5 and Black Carbon Exposure Due to Different Source Sectors in Stockholm, Gothenburg and Umea, Sweden. *International Journal of Environmental Research and Public Health* 2017;14(7) doi: Artn 742 10.3390/Ijerph14070742
28. Berkowicz R. OSPM - A Parameterised Street Pollution Model. *Environmental monitoring and assessment* 2000;65(1):323-31. doi: 10.1023/A:1006448321977
29. Hausberger S, Rexeis M, Zallinger M. Emission Factors from the Model PHEM for the HBEFA Version 3., Institute for internal combustion engines and thermodynamics, Graz University of Technology. 2009
30. Ilias V, Leonidas N, Zissis S. Methodology for the Quantification of Road Transport PM-Emissions, Using Emission Factors or Profiles. Deliverable D1.1.2, Updated February 2013. TRANSPHORM, http://www.transphorm.eu/Portals/51/Documents/Deliverables/New%20Deliverables/D1.1.2_updated.pdf [accessed 26 October 2018]. 2013
31. Denby BR, Sundvor I, Johansson C, et al. A coupled road dust and surface moisture model to predict non-exhaust road traffic induced particle emissions (NORTRIP). Part 2: Surface moisture and salt impact modelling. *Atmos Environ* 2013;81:485-503. doi: https://doi.org/10.1016/j.atmosenv.2013.09.003

For peer review only - http://bmjopen.bmj.com/site/about/guidelines.xhtml

32. Denby BR, Sundvor I, Johansson C, et al. A coupled road dust and surface moisture model to predict non-exhaust road traffic induced particle emissions (NORTRIP). Part 1: Road dust loading and suspension modelling. *Atmos Environ* 2013;77:283-300. doi: https://doi.org/10.1016/j.atmosenv.2013.04.069
33. Omstedt G, Bringfelt B, Johansson C. A model for vehicle-induced non-tailpipe emissions of particles along Swedish roads. *Atmos Environ* 2005;39(33):6088-97. doi: https://doi.org/10.1016/j.atmosenv.2005.06.037
34. Jalkanen JP, Johansson L, Kukkonen J, et al. Extension of an assessment model of ship traffic exhaust emissions for particulate matter and carbon monoxide. *Atmos Chem Phys* 2012;12(5):2641-59. doi: 10.5194/acp-12-2641-2012
35. Forsberg B, Hansson HC, Johansson C, et al. Comparative health impact assessment of local and regional particulate air pollutants in Scandinavia. *Ambio* 2005;34(1):11-9. [published Online First: 2005/03/26]
36. Dersimonian R, Laird N. Metaanalysis in Clinical-Trials. *Control Clin Trials* 1986;7(3):177-88. doi: Doi 10.1016/0197-2456(86)90046-2
37. Cesaroni G, Badaloni C, Gariazzo C, et al. Long-term exposure to urban air pollution and mortality in a cohort of more than a million adults in Rome. *Environmental health perspectives* 2013;121(3):324-31. doi: 10.1289/ehp.1205862
38. Hamra GB, Guha N, Cohen A, et al. Outdoor Particulate Matter Exposure and Lung Cancer: A Systematic Review and Meta-Analysis. *Environmental health perspectives* 2014;122(9):906-11. doi: doi:10.1289/ehp/1408092
39. Raaschou-Nielsen O, Andersen ZJ, Beelen R, et al. Air pollution and lung cancer incidence in 17 European cohorts: prospective analyses from the European Study of Cohorts for Air Pollution Effects (ESCAPE). *The Lancet Oncology* 2013;14(9):813-22. doi: https://doi.org/10.1016/S1470-2045(13)70279-1
40. Lefler JS, Higbee JD, Burnett RT, et al. Air pollution and mortality in a large, representative U.S. cohort: multiple-pollutant analyses, and spatial and temporal decompositions. *Environmental health : a global access science source* 2019;18(1):101. doi: 10.1186/s12940-019-0544-9

For peer review only

For peer review only - http://bmjopen.bmj.com/site/about/guidelines.xhtml

Acknowledgement

This study was funded by the Swedish Environmental Protection Agency as part of the Swedish Clean Air and Climate Research Program (grant NV-06576-13). BF and JNS was also supported by NordForsk grant no. 75007. The GOT-MONICA and PPS cohorts were supported by the Swedish Research Council, 2013-5187 (SIMSAM). The SDPP cohort was funded by the Stockholm County Council, the Swedish Research Council, the Swedish Diabetes Association, the Novo Nordisk Scandinavia, and GlaxoSmithKline. The 60YO cohort was funded by the Stockholm County Council and the Swedish Research Council. The Swedish Twin Registry is managed by Karolinska Institutet and receives funding through the Swedish Research Council under the grant no. 2017-00641. Data collection in SALT was supported by a grant from the National Institutes of Health (NIH), grant no. 1R01 AG08724. The VIP-Umeå cohort was funded by the Västerbotten County Council. P.L.L. was supported by funding from the Swedish Research Council for Health, Working Life and Welfare (FORTE) 2015-00917 and Karolinska Institute's Strategic Research Area in Epidemiology (SFO-EPI). L.B. was financed by grants from the Swedish state under the agreement between the Swedish government and the county councils: the ALF-agreement (74580).

Role of the funding source

The paper reflects only the authors views, and the funders had no role in the design or conduct of the study.

Competing interests

All authors have completed the ICMJE uniform disclosure form at www.icmje.org/coi_disclosure.pdf and declare: no support from any organization other than those listed above for the submitted work; no financial relationships with any organizations that might have an interest in the submitted work in the previous three years; no other relationships or activities that could appear to have influenced the submitted work.

Author contributions

JNS performed the meta-analysis and wrote with support from BF the first draft manuscript. JNS, EA, NA and GS conducted the cohort analyses. DS, KE, LG, CJ were responsible for dispersion modelling of particles. PW, AR, DR, KL, AL and PM and were responsible for collection of cohort data. GP, BF, LB, TB, JNS, GS, LS, PL, EA and NA designed the epidemiological study. All authors contributed to the interpretation of the results, finalization of the manuscript, and approved the final manuscript.

Transparency declaration

The lead author affirms that the manuscript is an honest, accurate, and transparent account of the study being reported; that no important aspects of the study have been omitted; and that any discrepancies from the study as planned have been explained.

Data sharing statement

No additional data available

Dissemination declaration

For peer review only

Dissemination of the results to study participants and/or patient organisations is not applicable.

For peer review only

Figure 1a-c. Boxplot and density function of a) PM10, b) PM2.5 and c) BC concentrations, in total and separate for each cohort. Tabulated values are quartile limits (Q1-Q3) and interquartile ranges (IQR:s) together with means and standard deviations.

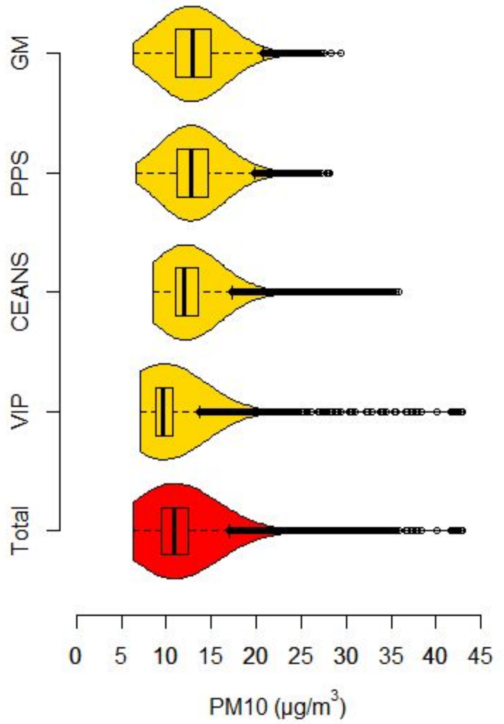

	Median (Q1-Q3)	IQR	Mean (sd)
GM	12.9 (11.1-14.9)	3.9	13.1 (2.8)
PPS	12.8 (11.3-14.7)	3.5	13.1 (2.6)
CEANS	12.0 (11.1-13.6)	2.5	12.5 (2.2)
VIP	9.6 (8.9-10.8)	1.9	9.9 (1.5)
Total	10.9 (9.5-12.5)	3.0	11.3 (2.4)

ew only

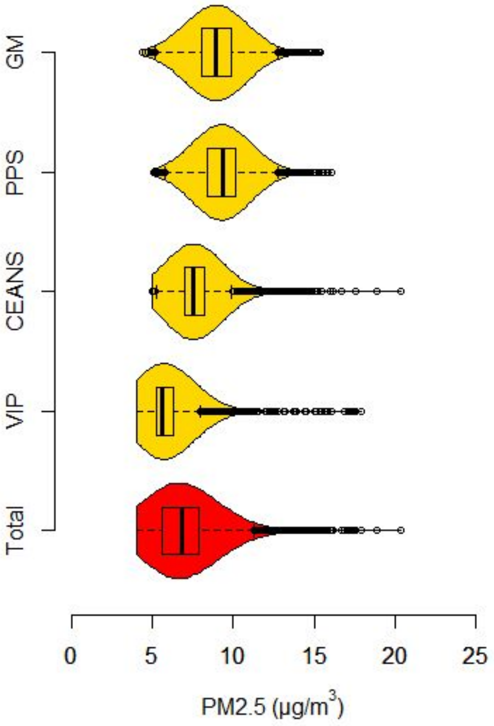

	Median (Q1-Q3)	IQR	Mean (sd)
GM	9.0 (8.0-9.9)	1.9	9.0 (1.4)
PPS	9.3 (8.4-10.2)	1.7	9.3 (1.3)
CEANS	7.5 (7.0-8.2)	1.2	7.6 (0.9)
VIP	5.7 (5.3-6.3)	1.1	5.8 (0.8)
Total	6.8 (5.6-7.9)	2.3	6.9 (1.5)

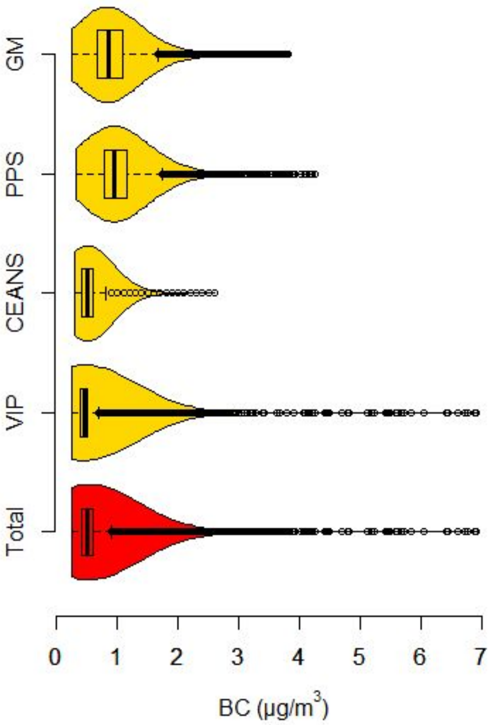

	Median (Q1-Q3)	IQR	Mean (sd)
GM	0.8 (0.7-1.1)	0.4	0.9 (0.4)
PPS	0.9 (0.8-1.2)	0.4	1.0 (0.4)
CEANS	0.5 (0.4-0.6)	0.2	0.5 (0.2)
VIP	0.4 (0.4-0.5)	0.1	0.5 (0.1)
Total	0.5 (0.4-0.6)	0.2	0.6 (0.3)

CEANS, Cardiovascular Effects of Air Pollution and Noise Study; MONICA, Multinational Monitoring of

Trends and Determinants in Cardiovascular Diseases; PPS, Primary Prevention Study; VIP, Västerbotten Intervention Programme

For peer review only

Figure 2a-c. Natural mortality

Figure 2a-c. Adjusted hazard ratios of natural mortality associated with a) PM10, b) PM2.5 and c) BC within each cohort and by random effect meta-analyses estimates. Exposures were assessed by moving average residential concentrations within the exposure windows last 5 years and 6 to 10 years prior.

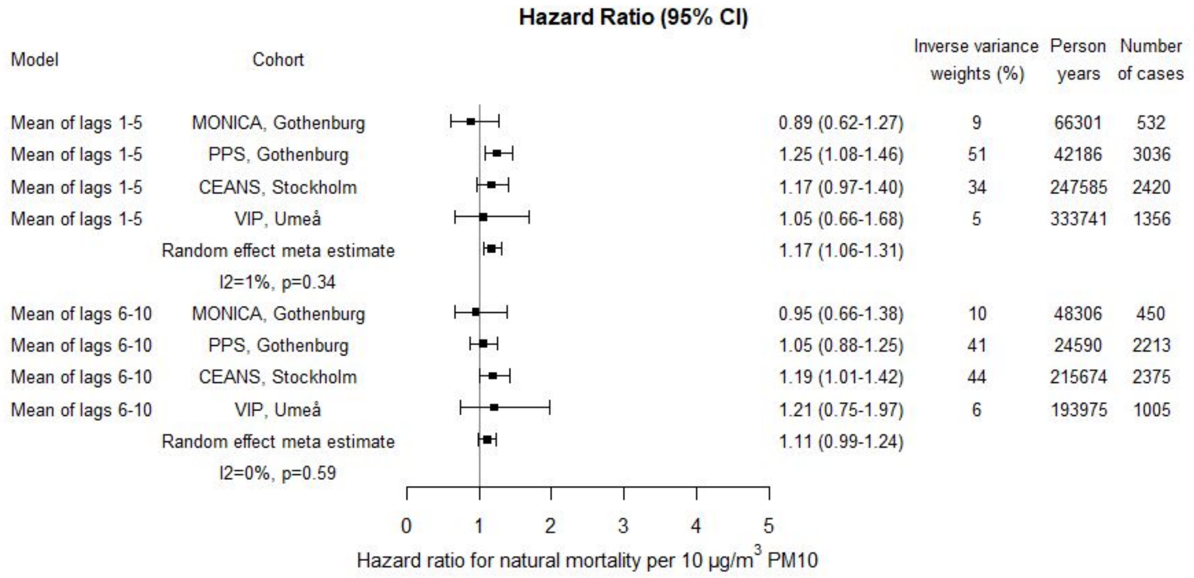

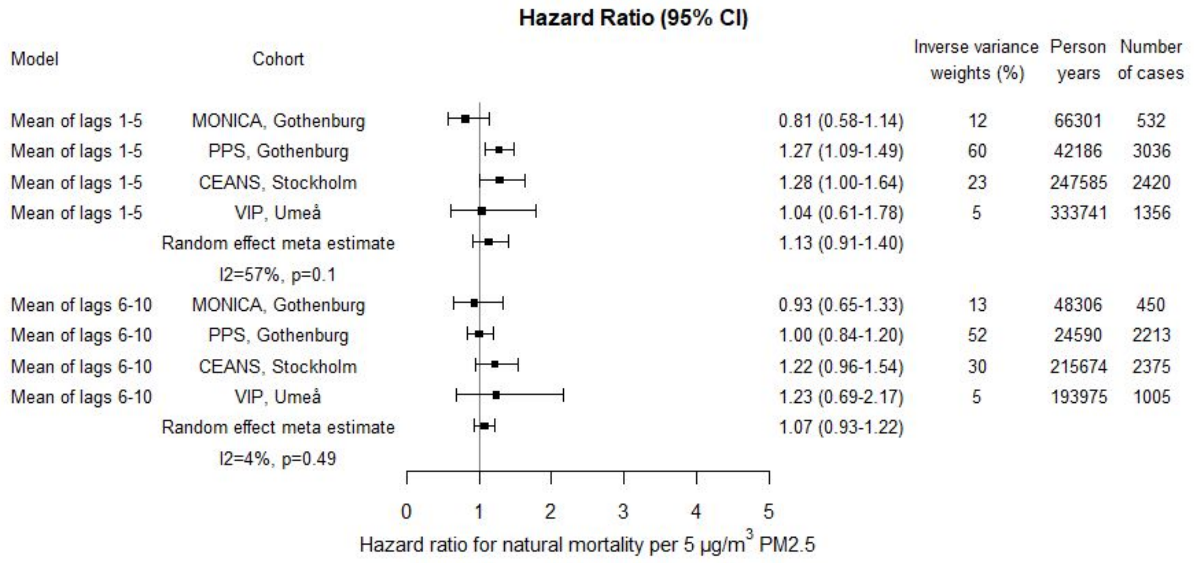

Figure 2a-c. Natural mortality

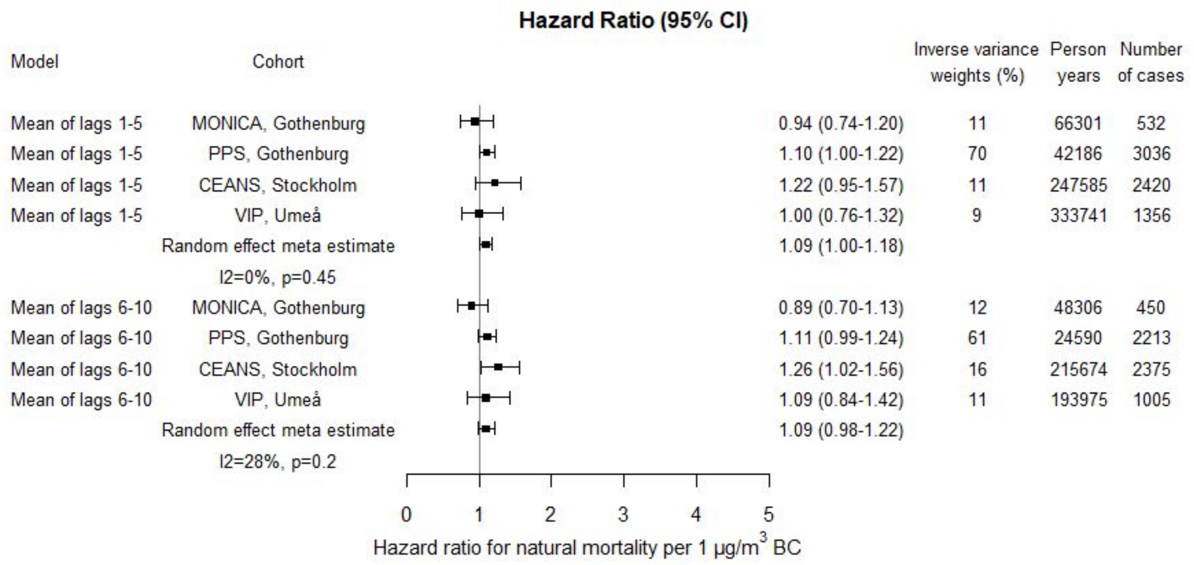

For peer review only

Figure 3a-c. CVD mortality

Figure 3a-c. Adjusted hazard ratios of CVD mortality associated with a) PM10, b) PM2.5 and c) BC within each cohort and by random effect meta-analyses estimates. Exposures were assessed by moving average residential concentrations within the exposure windows last 5 years and 6 to 10 years prior.

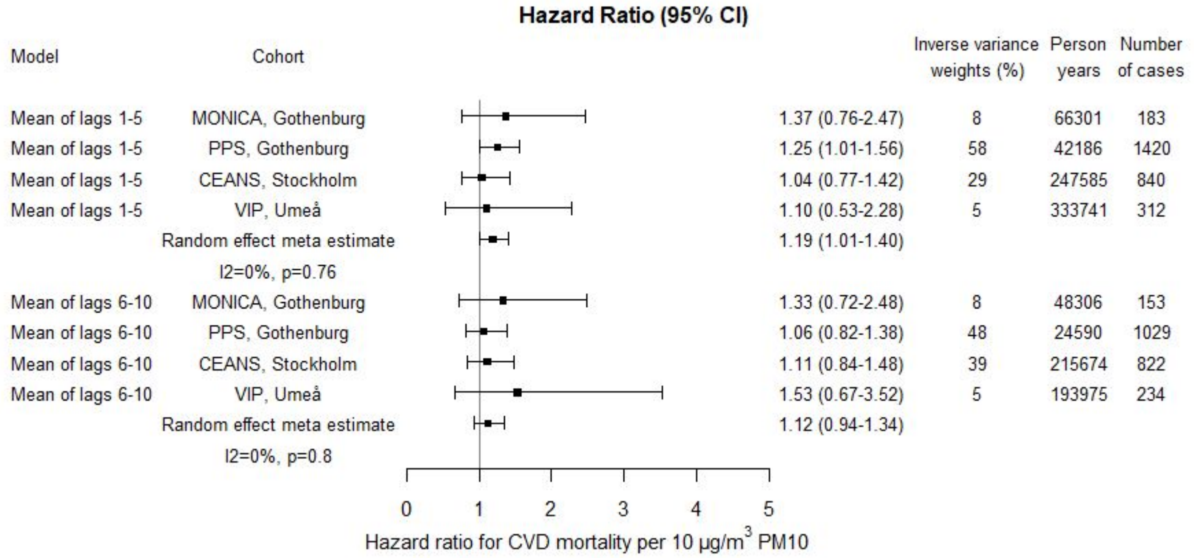

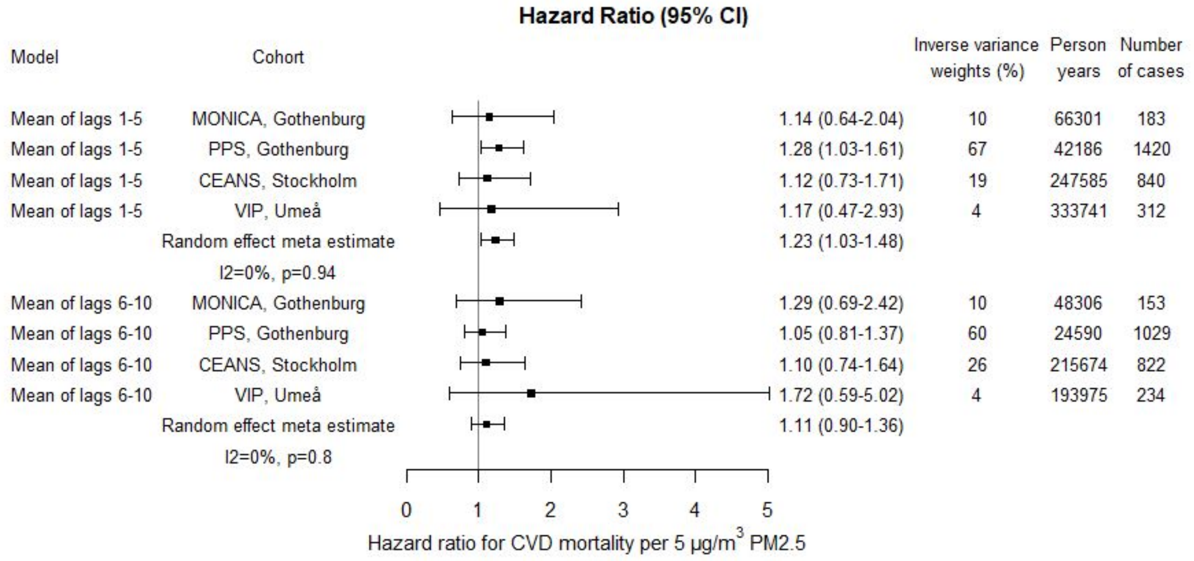

Figure 3a-c. CVD mortality

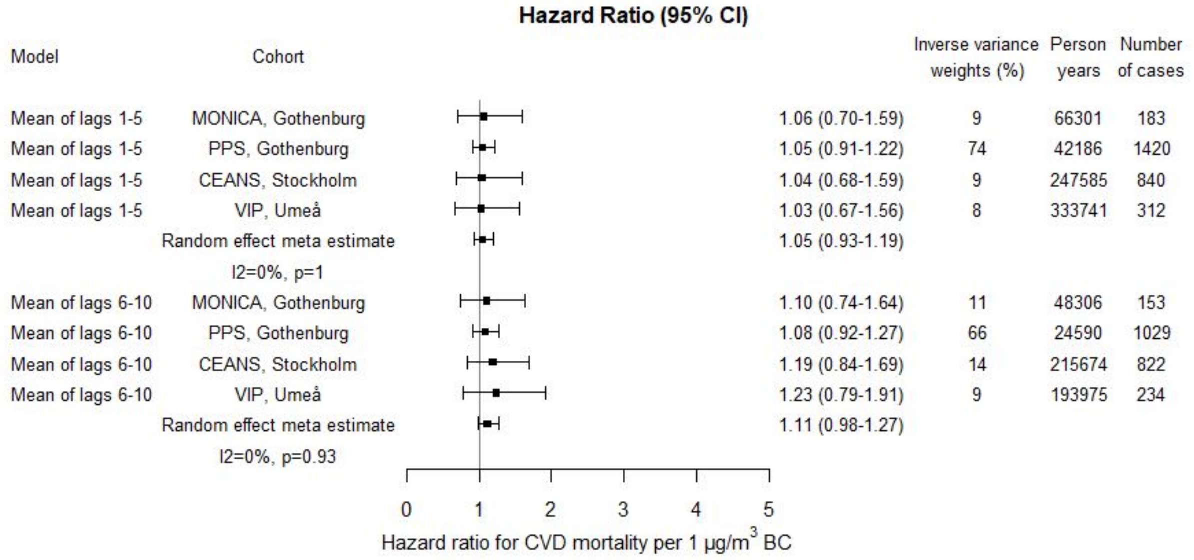

For peer review only

Figure S1a-c. Crude hazard ratios of natural mortality associated with a) PM10, b) PM2.5 and c) BC within each cohort and by random effect meta-analyses estimates. Exposures were assessed by moving average residential concentrations within the exposure windows last 5 years and 6 to 10 years prior.

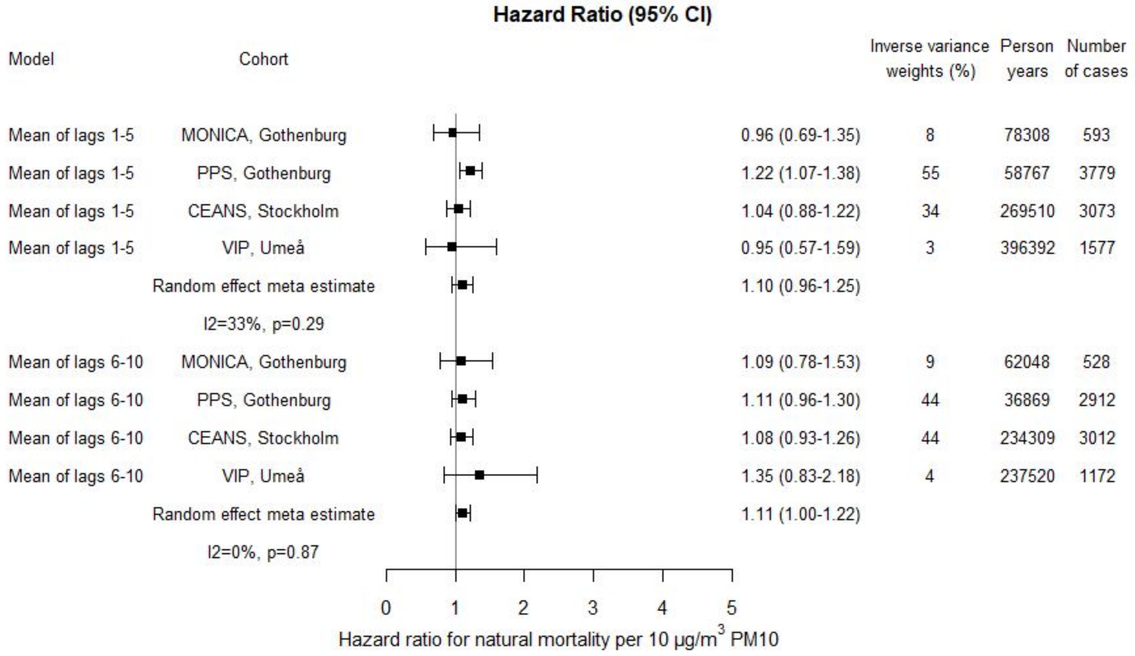

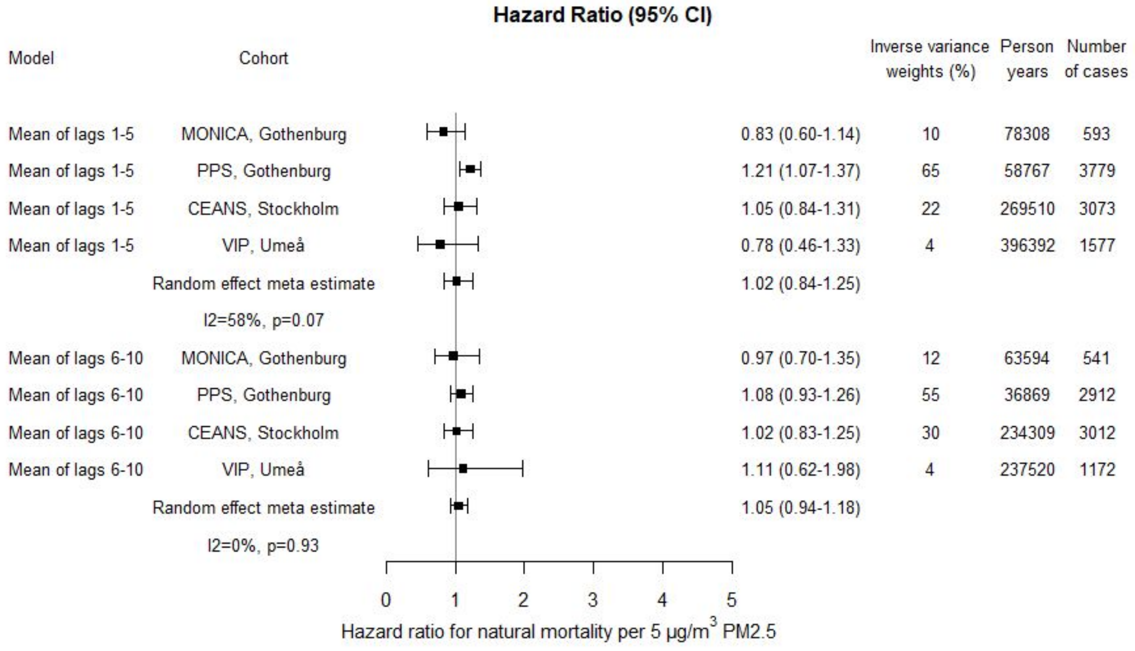

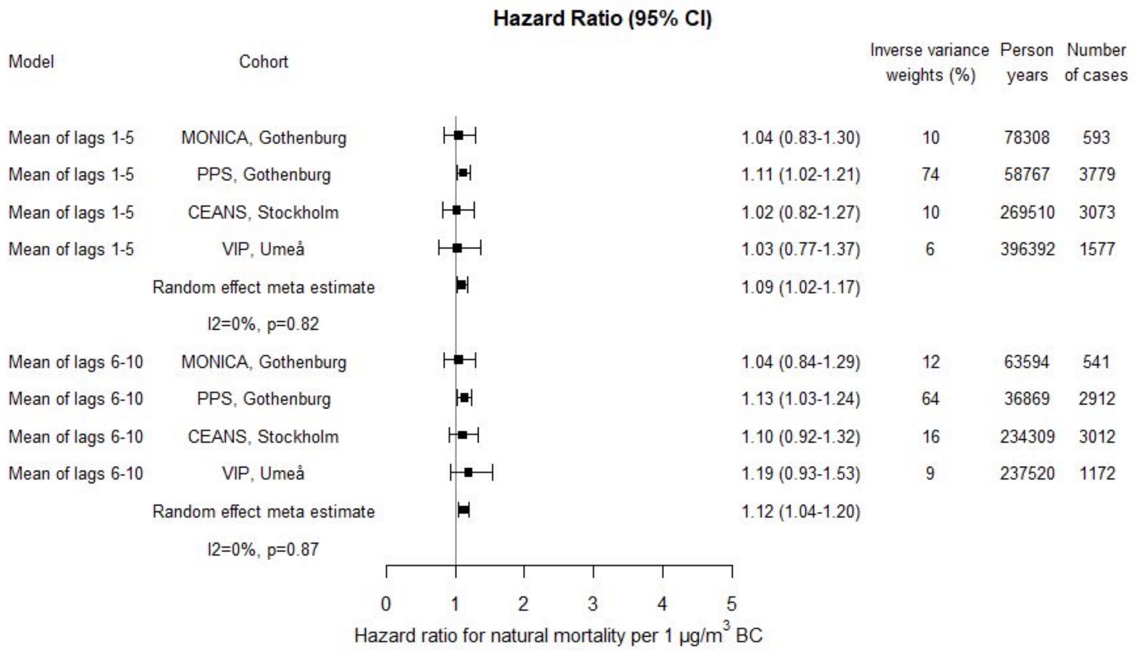

For peer review only

Figure S2a-c. Crude hazard ratios of CVD mortality associated with a) PM10, b) PM2.5 and c) BC within each cohort and by random effect meta-analyses estimates. Exposures were assessed by moving average residential concentrations within the exposure windows last 5 years and 6 to 10 years prior.

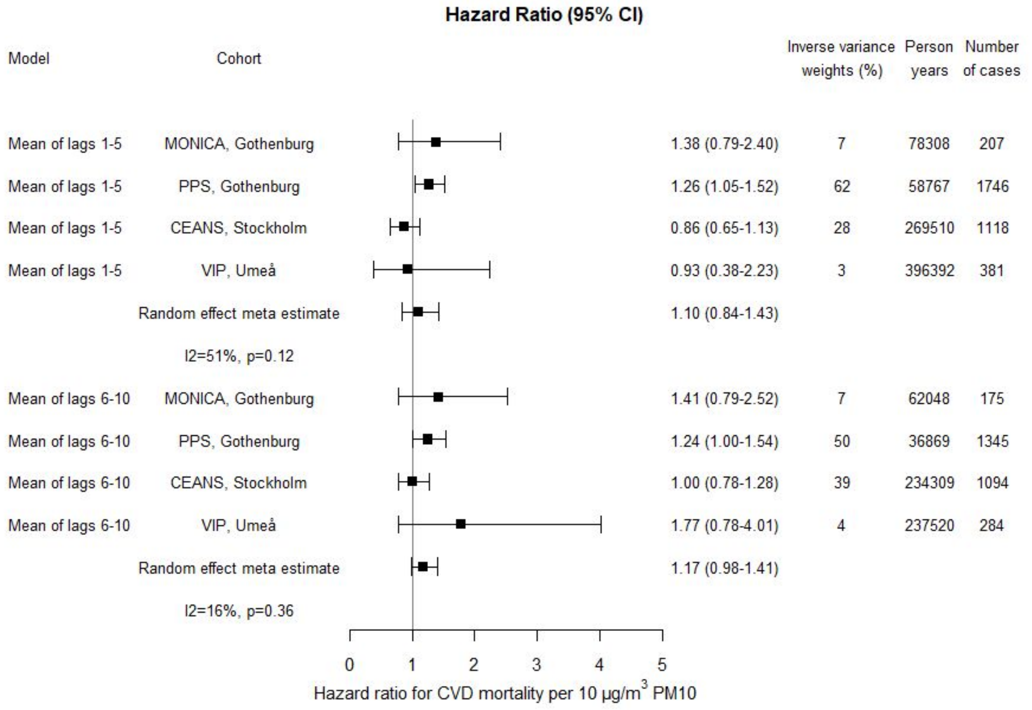

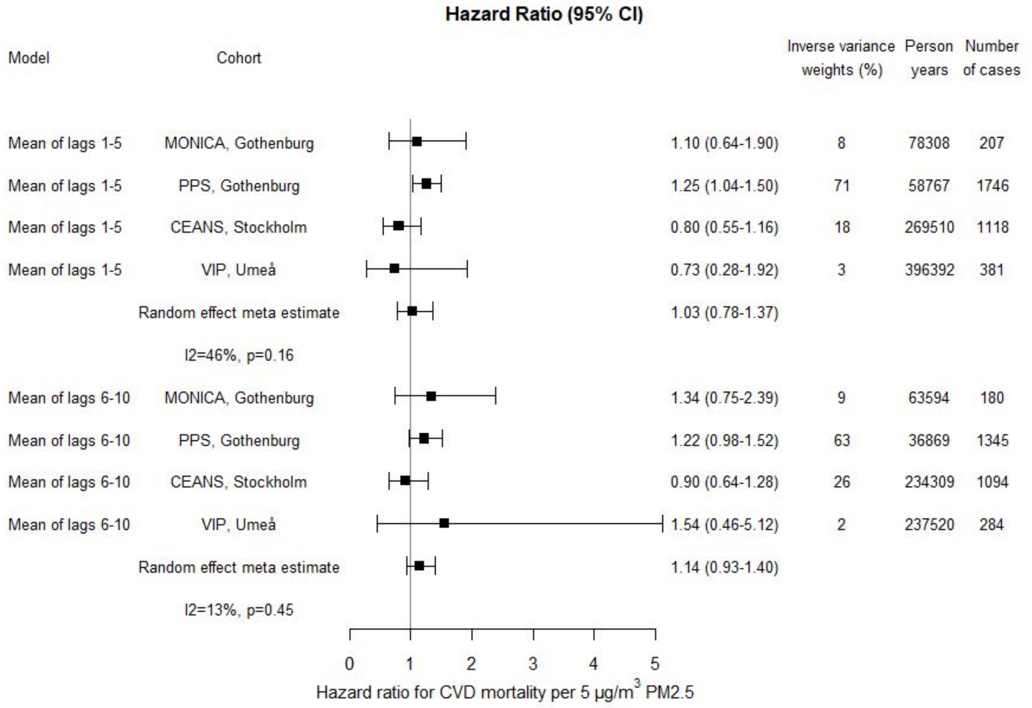

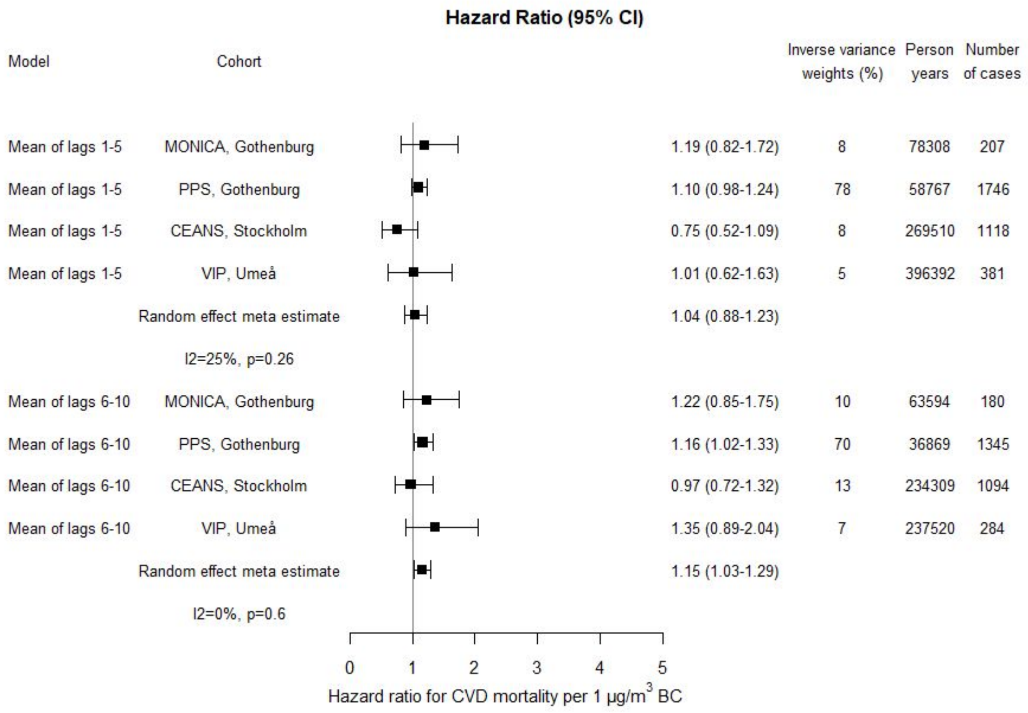
Hazard Ratio (95% CI)
Model
Cohort
Inverse variance weights (%)
Person years
Number of cases
Mean of lags 1-5 | MONICA, Gothenburg | 1.19 (0.82-1.72) | 8 | 78308 | 207
Mean of lags 1-5 | PPS, Gothenburg | 1.10 (0.98-1.24) | 78 | 58767 | 1746
Mean of lags 1-5 | CEANS, Stockholm | 0.75 (0.52-1.09) | 8 | 269510 | 1118
Mean of lags 1-5 | VIP, Umeå | 1.01 (0.62-1.63) | 5 | 396392 | 381
Random effect meta estimate | 1.04 (0.88-1.23)
I2=25%, p=0.26
Mean of lags 6-10 | MONICA, Gothenburg | 1.22 (0.85-1.75) | 10 | 63594 | 180
Mean of lags 6-10 | PPS, Gothenburg | 1.16 (1.02-1.33) | 70 | 36869 | 1345
Mean of lags 6-10 | CEANS, Stockholm | 0.97 (0.72-1.32) | 13 | 234309 | 1094
Mean of lags 6-10 | VIP, Umeå | 1.35 (0.89-2.04) | 7 | 237520 | 284
Random effect meta estimate | 1.15 (1.03-1.29)
I2=0%, p=0.6
0
1
2
3
4
5
Hazard ratio for CVD mortality per 1 μg/m3 BC

Figure S3a-c. Adjusted hazard ratios of lung cancer mortality associated with a) PM10, b) PM2.5 and c) BC within each cohort and by random effect meta-analyses estimates. Exposures were assessed by moving average residential concentrations within the exposure windows last 5 years and 6 to 10 years prior.

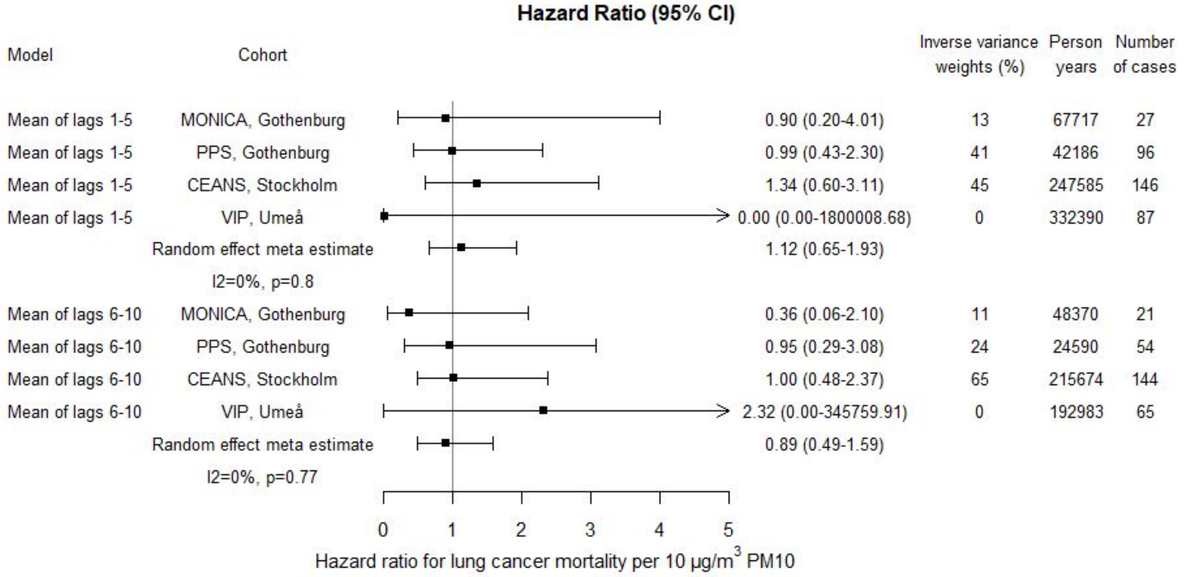

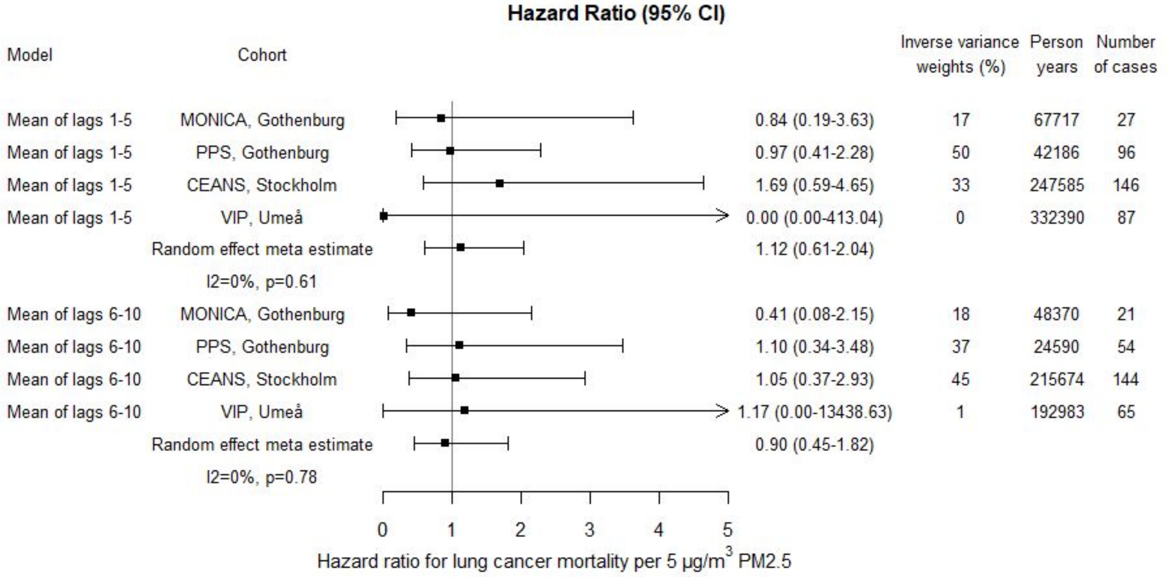

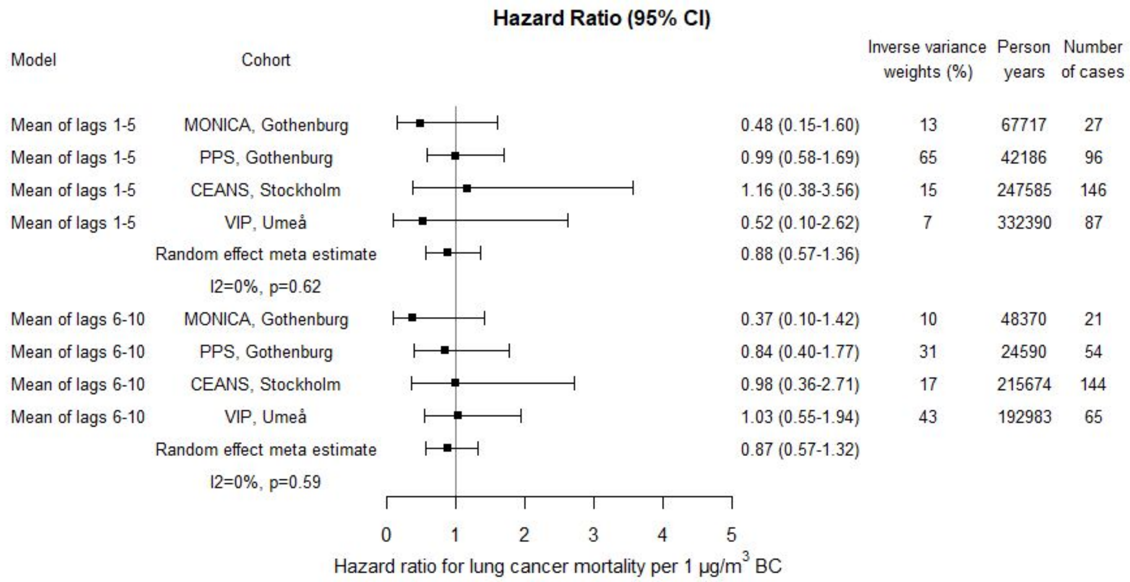

peer review only

Figure S4a-c. Adjusted hazard ratios of respiratory mortality associated with a) PM10, b) PM2.5 and c) BC within each cohort and by random effect meta-analyses estimates. Exposures were assessed by moving average residential concentrations within the exposure windows last 5 years and 6 to 10 years prior.

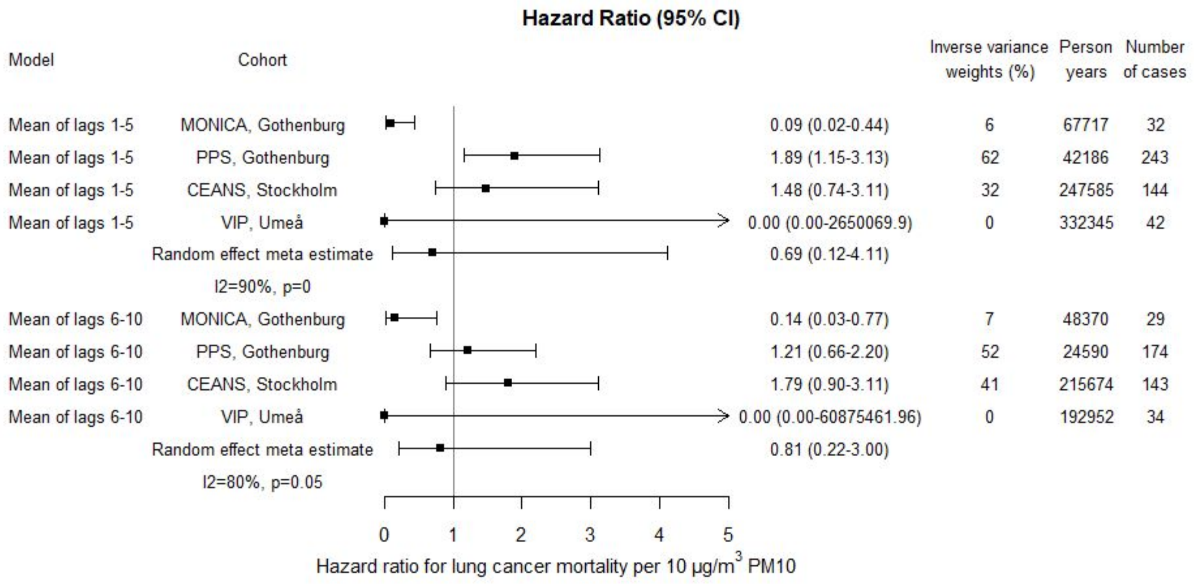

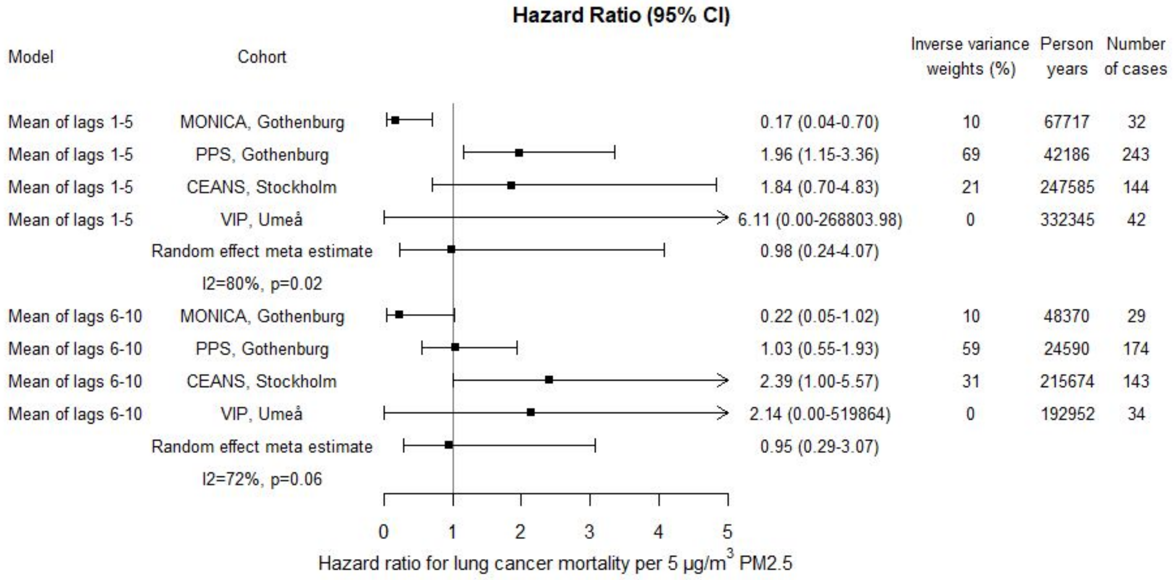

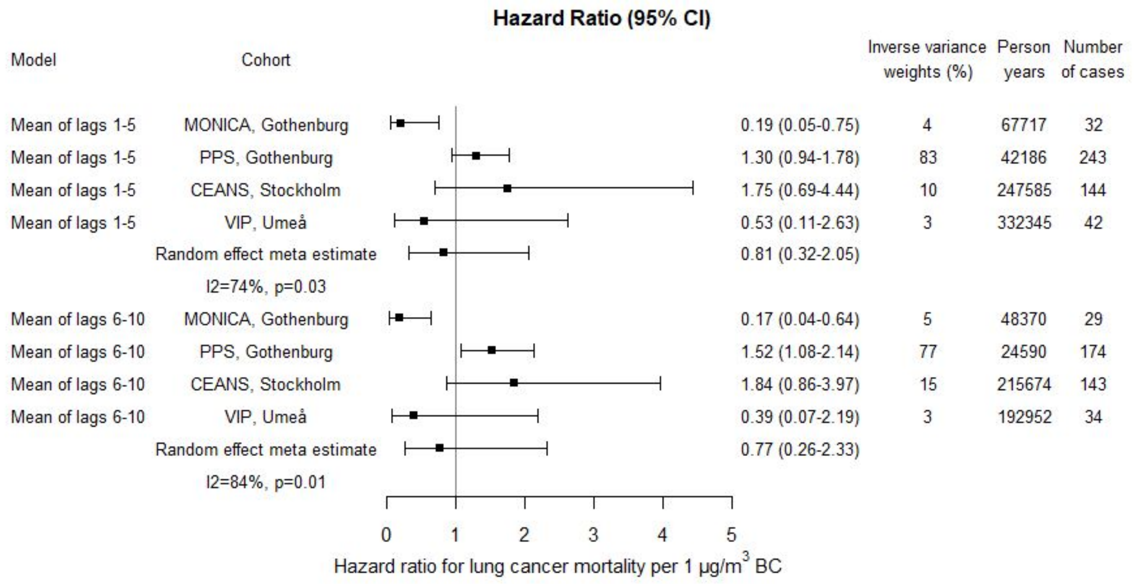

peer review only

Figure S5a-c. Adjusted hazard ratios of mortality by other casues (not CVD, respiratory, lung cancer or external causes) associated with a) PM10, b) PM2.5 and c) BC within each cohort and by random effect meta-analyses estimates. Exposures were assessed by moving average residential concentrations within the exposure windows last 5 years and 6 to 10 years prior.

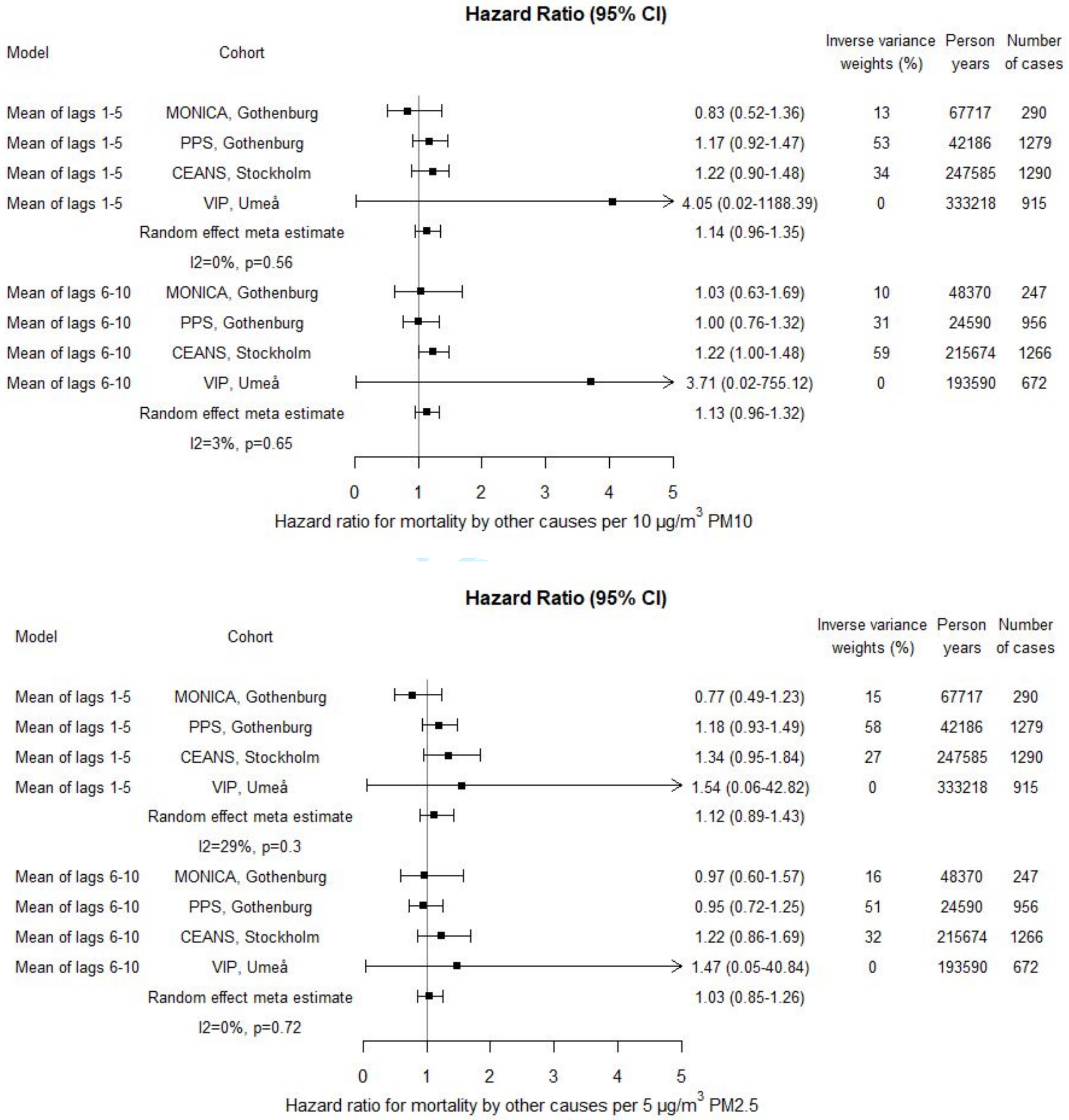

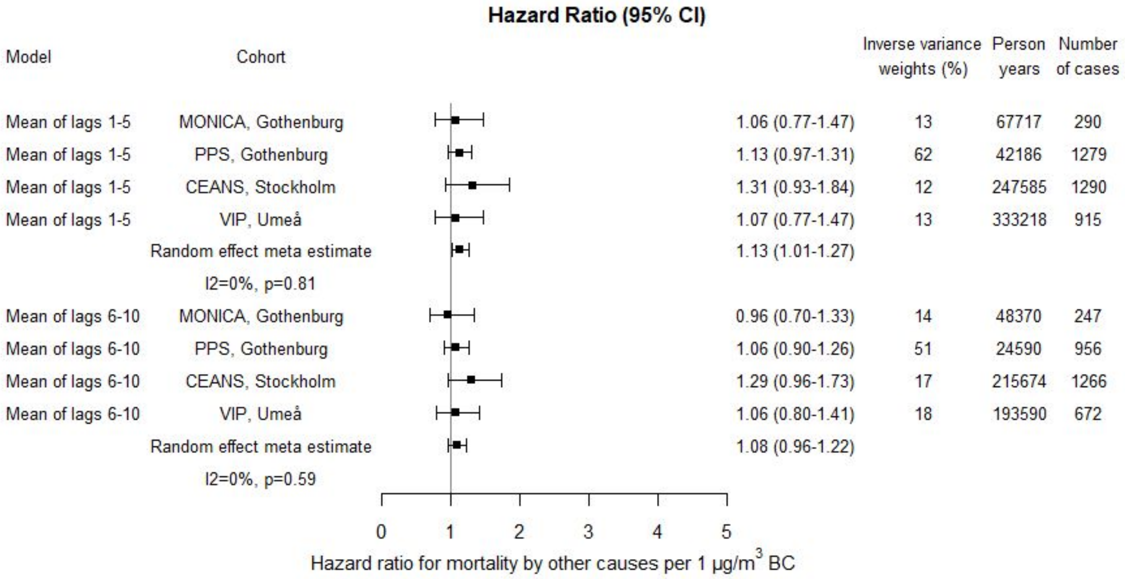

peer review only

Figure S6a-b. Adjusted hazard ratios of a) natural mortality and b) CVD mortality associated with PM10, PM2.5 and BC, within each cohort and by random effect meta-analyses estimates. Exposures were assessed by the residential concentration at the year of recruitment.

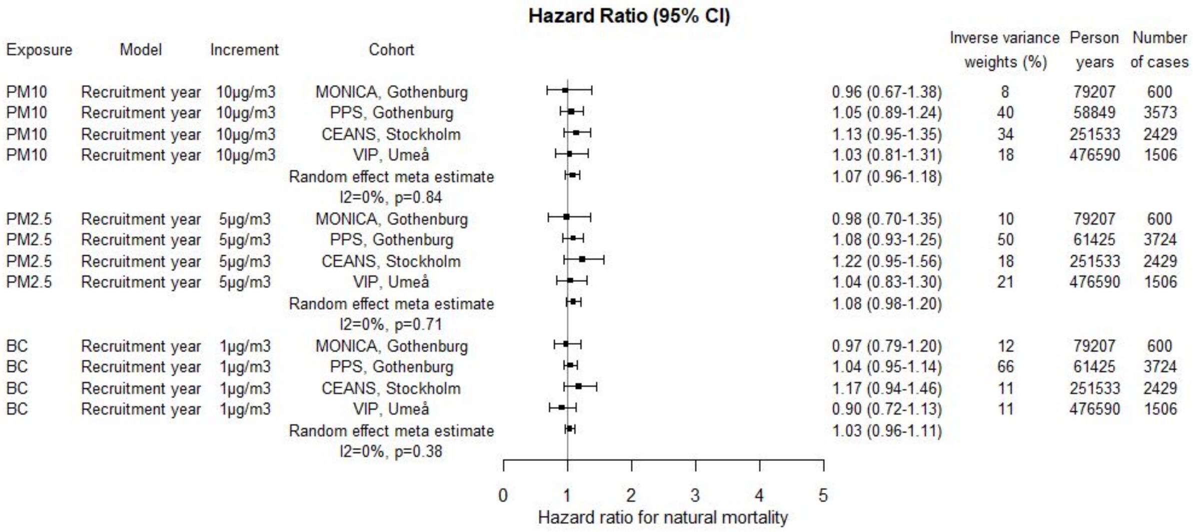

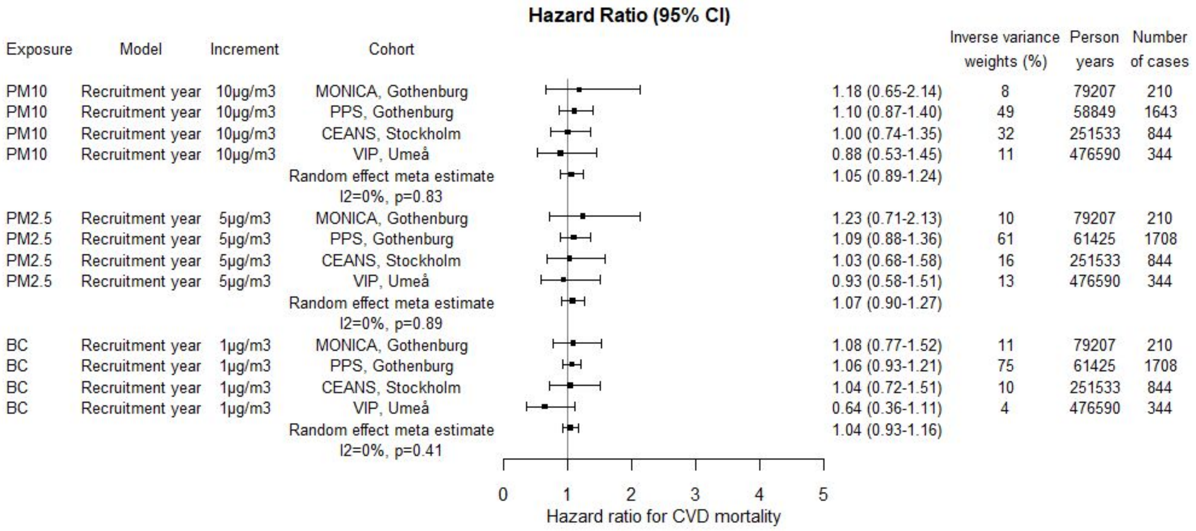

STROBE 2007 (v4) Statement—Checklist of items that should be included in reports of *cohort studies*

Section/Topic	Item #	Recommendation	Reported on page #
**Title and abstract**	1	(*a*) Indicate the study's design with a commonly used term in the title or the abstract	2
		(*b*) Provide in the abstract an informative and balanced summary of what was done and what was found	2
**Introduction**			
Background/rationale	2	Explain the scientific background and rationale for the investigation being reported	3
Objectives	3	State specific objectives, including any prespecified hypotheses	3-4
**Methods**			
Study design	4	Present key elements of study design early in the paper	4
Setting	5	Describe the setting, locations, and relevant dates, including periods of recruitment, exposure, follow-up, and data collection	4-6
Participants	6	(*a*) Give the eligibility criteria, and the sources and methods of selection of participants. Describe methods of follow-up	4-5
		(*b*) For matched studies, give matching criteria and number of exposed and unexposed	
Variables	7	Clearly define all outcomes, exposures, predictors, potential confounders, and effect modifiers. Give diagnostic criteria, if applicable	5-6
Data sources/ measurement	8*	For each variable of interest, give sources of data and details of methods of assessment (measurement). Describe comparability of assessment methods if there is more than one group	*5*
Bias	9	Describe any efforts to address potential sources of bias	10
Study size	10	Explain how the study size was arrived at	4-7
Quantitative variables	11	Explain how quantitative variables were handled in the analyses. If applicable, describe which groupings were chosen and why	5-6
Statistical methods	12	(*a*) Describe all statistical methods, including those used to control for confounding	6
		(*b*) Describe any methods used to examine subgroups and interactions	
		(*c*) Explain how missing data were addressed	6
		(*d*) If applicable, explain how loss to follow-up was addressed	6
		(*e*) Describe any sensitivity analyses	
**Results**			

For peer review only

Participants	13*	(a) Report numbers of individuals at each stage of study—eg numbers potentially eligible, examined for eligibility, confirmed eligible, included in the study, completing follow-up, and analysed	7
		(b) Give reasons for non-participation at each stage	
		(c) Consider use of a flow diagram	
Descriptive data	14*	(a) Give characteristics of study participants (eg demographic, clinical, social) and information on exposures and potential confounders	7
		(b) Indicate number of participants with missing data for each variable of interest	7
		(c) Summarise follow-up time (eg, average and total amount)	7
Outcome data	15*	Report numbers of outcome events or summary measures over time	7
Main results	16	(*a*) Give unadjusted estimates and, if applicable, confounder-adjusted estimates and their precision (eg, 95% confidence interval). Make clear which confounders were adjusted for and why they were included	7
		(*b*) Report category boundaries when continuous variables were categorized	
		(*c*) If relevant, consider translating estimates of relative risk into absolute risk for a meaningful time period	
Other analyses	17	Report other analyses done—eg analyses of subgroups and interactions, and sensitivity analyses	
**Discussion**			
Key results	18	Summarise key results with reference to study objectives	9
**Limitations**			
Interpretation	20	Give a cautious overall interpretation of results considering objectives, limitations, multiplicity of analyses, results from similar studies, and other relevant evidence	10
Generalisability	21	Discuss the generalisability (external validity) of the study results	10-11
**Other information**			
Funding	22	Give the source of funding and the role of the funders for the present study and, if applicable, for the original study on which the present article is based	12

*Give information separately for cases and controls in case-control studies and, if applicable, for exposed and unexposed groups in cohort and cross-sectional studies.

Note: An Explanation and Elaboration article discusses each checklist item and gives methodological background and published examples of transparent reporting. The STROBE checklist is best used in conjunction with this article (freely available on the Web sites of PLoS Medicine at http://www.plosmedicine.org/, Annals of Internal Medicine at http://www.annals.org/, and Epidemiology at http://www.epidem.com/). Information on the STROBE Initiative is available at www.strobe-statement.org.

For peer review only

BMJ Open

Long-term exposure to particulate air pollution and black carbon in relation to natural and cause specific mortality: a multi-cohort study in Sweden

Journal:	*BMJ Open*
Manuscript ID	bmjopen-2020-046040.R1
Article Type:	Original research
Date Submitted by the Author:	16-Feb-2021
Complete List of Authors:	Sommar, Johan; Umea University Department of Public Health and Clinical Medicine, Sustainable health<br>Andersson , Eva M.; Sahlgrenska University Hospital and University of Gothenburg, Occupational and Environmental Medicine<br>Andersson, Niklas; Institute of Environmental Medicine, Karolinska Institutet<br>Sallsten, Gerd; Sahlgrenska University Hospital and University of Gothenburg, Occupational and Environmental Medicine<br>Stockfelt, Leonard; Sahlgrenska University Hospital and University of Gothenburg, Occupational and Environmental Medicine<br>Ljungman, Petter; Karolinska Institutet<br>Segersson, David; SMHI<br>Eneroth, Kristina; SLB-analys, Environment and Health Administration<br>Gidhagen, Lars; SMHI<br>Molnar, Peter; Sahlgrenska University Hospital and University of Gothenburg, Occupational and Environmental Medicine<br>Wennberg, Patrik; Umeå University, Department of Public Health and Clinical Medicine, Family Medicine<br>Rosengren, Annika ; University of Gothenburg Sahlgrenska Academy, Institute of Medicine<br>Rizzuto, Debora; Karolinska Institutet , Aging Research Center, Department of Neurobiology, Health Care Sciences and Society; Stockholm University,<br>Leander, Karin; Karolinska Institutet, Institute of Environmental Medicine<br>Lager, Anton; Karolinska Institutet, Department of Public Health Sciences<br>Magnusson, Patrik; Karolinska Institutet, Dept. Medical Epidemiology and Biostatistics<br>Johansson, Christer; Department of Environmental Science and Analytical Chemistry<br>Barregard, Lars; Sahlgrenska University Hospital and University of Gothenburg, Occupational and Environmental Medicine<br>Bellander, Tom; Karolinska Institutet, Institute of Environmental Medicine; Stockholm County Council, Centre for Occupational and Environmental Medicine<br>Pershagen, Göran; Institute of Environmental Medicine, Karolinska Institutet<br>Forsberg, Bertil; Umeå University, Public Health and Clinical Medicine

**Primary Subject Heading**:	Epidemiology
Secondary Subject Heading:	Public health
Keywords:	EPIDEMIOLOGY, PUBLIC HEALTH, Cardiac Epidemiology < CARDIOLOGY

SCHOLARONE™
Manuscripts

I, the Submitting Author has the right to grant and does grant on behalf of all authors of the Work (as defined in the below author licence), an exclusive licence and/or a non-exclusive licence for contributions from authors who are: i) UK Crown employees; ii) where BMJ has agreed a CC-BY licence shall apply, and/or iii) in accordance with the terms applicable for US Federal Government officers or employees acting as part of their official duties; on a worldwide, perpetual, irrevocable, royalty-free basis to BMJ Publishing Group Ltd ("BMJ") its licensees and where the relevant Journal is co-owned by BMJ to the co-owners of the Journal, to publish the Work in this journal and any other BMJ products and to exploit all rights, as set out in our licence.

The Submitting Author accepts and understands that any supply made under these terms is made by BMJ to the Submitting Author unless you are acting as an employee on behalf of your employer or a postgraduate student of an affiliated institution which is paying any applicable article publishing charge ("APC") for Open Access articles. Where the Submitting Author wishes to make the Work available on an Open Access basis (and intends to pay the relevant APC), the terms of reuse of such Open Access shall be governed by a Creative Commons licence – details of these licences and which Creative Commons licence will apply to this Work are set out in our licence referred to above.

Other than as permitted in any relevant BMJ Author's Self Archiving Policies, I confirm this Work has not been accepted for publication elsewhere, is not being considered for publication elsewhere and does not duplicate material already published. I confirm all authors consent to publication of this Work and authorise the granting of this licence.

For peer review only

Long-term exposure to particulate air pollution and black carbon in relation to natural and cause specific mortality: a multi-cohort study in Sweden

Johan Nilsson Sommar[1*], Eva M. Andersson[2], Niklas Andersson[3], Gerd Sallsten[2], Leo Stockfelt[2], Petter L. S. Ljungman[3,4], David Segersson[5], Kristina Eneroth[6], Lars Gidhagen[5], Peter Molnar[2], Patrik Wennberg[7], Annika Rosengren[8], Debora Rizzuto[9,10], Karin Leander[11], Anton Lager[12,13], Patrik KE Magnusson[14], Christer Johansson[6,15], Lars Barregard[2], Tom Bellander[3,16], Göran Pershagen[3,16] and Bertil Forsberg[1]

[1] Section of Sustainable health, Department of Public Health and Clinical Medicine, Umeå University, Umeå, Sweden
[2] Occupational and Environmental Medicine, Department of Public Health and Community Medicine, Institute of medicine, Sahlgrenska Academy, University of Gothenburg & Sahlgrenska University Hospital, Sweden.
[3] Institute of Environmental Medicine, Karolinska Institutet, Stockholm, Sweden
[4] Department of Cardiology, Danderyd Hospital, Stockholm, Sweden
[5] Swedish Meteorological and Hydrological Institute, Norrköping, Sweden
[6] SLB-analys, Environment and Health Administration, Stockholm, Sweden
[7] Public Health and Clinical Medicine, Family Medicine, Umeå University, Umeå, Sweden
[8] Department of Molecular and Clinical Medicine, Institute of Medicine, University of Gothenburg, Sahlgrenska University Hospital, Gothenburg, Sweden
[9] Ageing Research Center, Department of Neurobiology, Care Sciences and Society, Karolinska Institutet and Stockholm University, Stockholm, Sweden
[10] Stockholm Gerontology Research Center, Stockholm, Sweden
[11] Institute of Environmental Medicine, Karolinska Institutet, Stockholm, Sweden
[12] Centre for Epidemiology and Community Medicine, Stockholm County Council, Stockholm, Sweden
[13] Department of Public Health Science, Karolinska Institutet, Stockholm, Sweden
[14] Department of Medical Epidemiology and Biostatistics, Karolinska Institutet, Stockholm, Sweden
[15] Department of Environmental Science, Stockholm University, Stockholm, Sweden
[16] Centre for Occupational and Environmental Medicine, Region Stockholm, Stockholm, Sweden

* Corresponding author:

Department of Public Health and Clinical Medicine, Section of Sustainable Health, Umeå University, Umeå, Sweden
Phone: +46 907853453
E-mail: johan.sommar@umu.se

For peer review only - http://bmjopen.bmj.com/site/about/guidelines.xhtml

Abstract

Objectives: To estimate concentration-response relationships for particulate matter (PM) and black carbon (BC) in relation to mortality in cohorts from three Swedish cities with comparatively low pollutant levels.

Setting: Cohorts from Gothenburg, Stockholm and Umeå, Sweden.

Design: High-resolution dispersion models were used to estimate annual mean concentrations of PM with aerodynamic diameter ≤10 µm (PM10) and ≤2.5 µm (PM2.5), and BC, at individual addresses during each year of follow up 1990-2011. Moving averages were calculated for the time windows 1-5 years (lag 1-5) and 6-10 years (lag 6-10) preceding the outcome. Cause-specific mortality data were obtained from the national cause of death registry. Cohort-specific hazard ratios were estimated using Cox regression models and then meta-analysed including a random effect of cohort.

Participants: During the study period, 7340 cases of natural mortality, 2755 cases of cardiovascular disease (CVD) mortality and 817 cases of respiratory and lung cancer mortality were observed among in total 68679 individuals and 689813 person-years of follow-up.

Results: Both PM10 (range: 6.3–41.9 µg/m^3) and BC (range: 0.2-6.8 µg/m^3) were associated with natural mortality showing 17% (95% CI 6-31%) and 9% (95% CI 0-18%) increased risks per 10 µg/m^3 and 1 µg/m^3 of lag1-5 exposure, respectively. For PM2.5 (range: 4.0–22.4 µg/m^3) the estimated increase was 13% per 5 µg/m^3, but less precise (95% CI -9-40%). Estimates for CVD mortality appeared higher for both PM10 and PM2.5. No association was observed with respiratory mortality.

Conclusion: The results support an effect of long-term air pollution on natural mortality and mortality in CVD with high relative risks also at low exposure levels. These findings are relevant for future decisions concerning air quality policies.

Key words: air pollution, particulate matter, black carbon, natural mortality, cardiovascular mortality

For peer review only

Strengths and limitations of this study

Few previous studies have followed up individuals by home address and time varying exposures as annual mean concentrations at actual place of residence

High-resolution dispersion models of particle concentrations were used to capture local contrasts in exposure

The study did however not include information on noise exposure and green space near the home address

This study showed associations with mortality within populations with comparatively low particle concentrations, generally below EU standards

As the first (multi)-cohort study using high resolution dispersion modelled particle concentrations at mean levels below WHO guidelines, this study found high relative risks of mortality

For peer review only - http://bmjopen.bmj.com/site/about/guidelines.xhtml

Introduction

Particulate matter ambient air pollution exposure has been associated with increased risk of premature mortality in several cohort studies [1 2]. According to the Global Burden of Diseases Study 2017 [3], assessing 84 risk factors for premature mortality in 195 countries, ambient particulate matter air pollution was ranked as the tenth most important risk factor for mortality and the most important environmental risk factor. Using risk functions restricted to cohort studies of outdoor air pollution, ambient particle matter pollution was estimated to cause 2.9 million (95% confidence interval (CI): 2.5-3.4) deaths in year 2017.

In a review of 11 cohort studies on long-term air pollution exposure and mortality, ambient concentrations of PM2.5 were associated with increased risk of all-cause mortality and particularly cardiovascular mortality. However, the heterogeneity between study relative risk estimates was large [2]. The large European Study of Cohorts for Air Pollution Effects (ESCAPE) project including 22 cohorts, observed an increased risk of overall mortality associated with ambient PM2.5 concentrations [4] whereas no association was found with cardiovascular mortality [5]. In a recent study within a large Danish cohort found increased risks for both natural cause and CVD mortality associated with PM2.5 exposure during the last 15 years [6]. Neither the Danish study nor the ESCAPE study were able to demonstrate associations with respiratory mortality [6 7].

Black carbon (BC), one component of PM, has been hypothesized to play a key role in the adverse health effects of air pollution exposure [8]. The review by Luben et al. identified three studies on the long-term effect of BC (or elemental carbon) and mortality in coronary heart disease [9] and ischemic heart disease [10 11]. However, the review did not find support for a stronger association with mortality for BC compared with PM2.5. Elemental carbon has been associated with all-cause mortality in another meta-analysis [2]. Within the ESCAPE project, PM2.5-absorbance was used as a proxy for BC. The estimated increased risk of natural mortality associated with PM2.5-absorbance did not reach statistical significance [4]. In Denmark (areas of Copenhagen and Aarhus), BC was, however, associated with both all-cause and cardiovascular mortality [6].

These previous studies varied in the method used for the exposure assessment of particle concentrations. Earlier studies assessed concentrations using centrally located monitoring stations, mostly representing the urban background [12]. Later studies use air pollution monitoring together with data on land use (land use regression, LUR) or detailed data on emissions and meteorological conditions (dispersion models). Most studies on long-term air pollution effects have assessed outdoor residential exposures at the year of inclusion into the cohort, a recent study on mortality however used annual mean concentrations on addresses during follow-up [6]. Studies using exposure assessments with a higher precision and in low-level exposure environments demonstrated higher relative risks of mortality in a recent meta-analysis [13]. Studies on low level air pollution are few, however. Adding to the knowledge from the long-term air pollution effects on mortality from the Canadian Community Health Survey-Mortality cohort [14-16] and a Medicare population in New England [17], this study is of importance for policy making and determination of limit values. Compared to these previous studies that used a 1x1 km^2 resolution for the assessment of air pollutant concentrations this study will make use of high-resolution 50x50 m^2 dispersion modelled particulate matter able to capture also local exposure contrasts.

Aim

This study aims to use high-resolution dispersion modelled annual mean residential PM10, PM2.5 and BC concentrations to estimate associations with natural and cause specific mortality in a

For peer review only - http://bmjopen.bmj.com/site/about/guidelines.xhtml

northern European environment characterized by annual averages largely compliant with EU air quality standards.

Methods and Materials

Study Cohorts

The study included four cohorts; two cohorts from Gothenburg, one from Stockholm (pooled from four cohorts) and one cohort from Umeå. Our study period ranged from January 1st 1990 to December 31st 2011 (December 31st 2013 for Umeå participants residing at the same address as in 2011). Residential address history was obtained for all cohort participants through linkage using personal identification numbers to mandatory records of residential addresses at Statistics Sweden or the Swedish Taxation Authority. These residential addresses were then geocoded by matching against the Swedish Mapping Cadastral and Land Registration Authority databases. When needed, addresses were manually checked and corrected for inconsistencies and assigned geographical coordinates.

From Gothenburg, two general population cohorts were included: The Primary Prevention Study (PPS) cohort and the GOT-MONICA cohort. The PPS cohort recruited a random third of all men in Gothenburg born between the years 1915 and 1925 who were examined in 1970-73 to study predictors of cardiovascular disease [18]. The GOT-MONICA cohort is one of the cohorts within the international MONICA project ("Multinational Monitoring of Trends and Determinants in Cardiovascular Diseases"), designed to study risk factors for cardiovascular diseases [19 20]. GOT-MONICA included residents aged 25-64 years. Recruitments were conducted in 1985, 1990 and 1995. For both cohorts, background data and information on cardiovascular risk factors were recorded by questionnaires. The recruited individuals were also examined by health care professionals.

Recruiting individuals from Stockholm County the Cardiovascular Effects of Air pollution and Noise Study (CEANS) cohort was included [21]. The cohort consists of four sub-cohorts. The Stockholm Diabetes Prevention Program [SDPP] was designed as a population-based prospective cohort, and includes 3128 men recruited between the years 1992 and 1994 and 4821 women recruited between the years 1996 and 1998. The program recruited individuals aged 35-56 years that had not previously been diagnosed with diabetes. About half of the recruited had a first or second degree relative with a history of diabetes. These were then matched with recruits of the same age and sex, who did not have a relative with a diabetes history. The second sub-cohort named SIXTY consists of a population-based random sample of 60-year old men and women (N=4232) living within Stockholm County between August 1997 and March 1999. The third cohort consists of the Screening Across the Lifespan Twin study [SALT], including twins born before year 1958. Only participants residing in Stockholm County at recruitment were included in the current analysis (N=7043). The fourth sub-cohort, the Swedish National Study on Aging and Care in Kungsholmen [SNAC-K], included a random sample of inhabitants aged at least 60 years and residing in a central area of Stockholm City (N=3363). Sub-cohort specific characteristics of included individuals in CEANS have previously been presented [22].

Including Umeå municipality, the Västerbotten intervention program (VIP) is a program where the population in the County is invited to a health examination the year they turn 40, 50 and 60 (and during some years also 30) years old [23]. The program was initiated in parts of the county in 1985 as a community and individual level program to reduce the morbidity and mortality from cardiovascular disease in Northern Sweden. The interview questions provide information about cardiovascular risk factors as well as social situation, education, diet and physical activity. So far more than 100 000 individuals have participated in the program.

For peer review only - http://bmjopen.bmj.com/site/about/guidelines.xhtml

The study was reviewed and approved by the Ethical Review Boards of Gothenburg (references T800-08 and T547- 13), Stockholm (reference 2018/2064-32), and Umeå (reference 2015/16-31Ö).

The public was not involved in the design, or conduct, or reporting, or dissemination plans of the research.

Patient and public involvement

No patient involved.

Mortality Outcomes

Cause-specific mortality was determined by linkage of national personal identification numbers to the Cause of Death Register at the Swedish National Board of Health and Welfare. We used the International Code of Diseases (ICD)9 001-779 or ICD-10 A00-R99 to define deaths by natural causes, ICD-9 400-440 or ICD-10 I10-I70 to define deaths in CVD, ICD9 162 or ICD10 C34 to define lung cancer deaths, and ICD9 460-519 or ICD10 J0-99 to define non-malignant respiratory mortality. In addition, deaths by other causes was studied (that is other than CVD, lung cancer, respiratory disease, and external causes).

Exposure Assessment

An in depth description of the dispersion model used for the exposure assessment has previously been published [24]. Both regional and local emission inventories for the years 1990, 2000 and 2011 in Gothenburg and Umeå, and years 1990, 1995, 2000, 2005 and 2011 in Stockholm, were used as input to Gaussian dispersion model simulations of annual mean concentrations of particulate matter (PM), i.e. PM10, PM2.5 and BC. To allow high resolution in vicinity of roads, a quadtree receptor grid was used, resulting in a resolution down to 35 m x 35 m. For inner-city streets with buildings on one or both sides, an additional concentration component was simulated with the Danish operational street pollution model (OSPM) [25].

Monitoring data were used in Stockholm to adjust the modelled concentrations to meteorological year-to-year variability. In Gothenburg and Umeå interpolation was used between the years and each year was also adjusted by a ventilation factor taking meteorological variability into account. Emission factors for traffic PM-exhaust for different vehicle types, speeds and driving conditions were calculated based on the Handbook on Emission Factors for Road Traffic version 3.1 [26], whereas emission factors for black carbon were based on TRANSPHORM [27]. The non-exhaust emissions of PM consist mainly of road wear particles, with a minor contribution from brake and tire wear [28-30]. For small-scale residential heating, emissions in Gothenburg and Stockholm were determined using household energy consumption data from Statistics Sweden with a resolution of 100 m x 100 m. This was based on proxy data including number of stoves or boilers in each municipality, living space of small houses per square km, population density per 100 m^2 and availability of district heating. In Umeå a detailed inventory of individual stoves and boilers was used with data from chimney sweepers and interviews about amount of wood burning [24]. Industrial and energy production facilities were included as point sources in the model. Emission from shipping were also included using a method described by Jalkanen et al. [31].

To model the long-range transport of PM10, PM2.5 and BC the difference between total concentrations measured at monitoring stations and modelled local particle concentrations at the same location was considered on an annual basis (Gothenburg and Umeå). In Stockholm annual concentrations from a regional background station was used to estimate the long-range transport. The long-range transport contributions were estimated differently for each city, but within each city

For peer review only - http://bmjopen.bmj.com/site/about/guidelines.xhtml

they had a purely temporal contrast. This was assumed since the spatial variation between regional background sites has been shown to be small [32]. These yearly contributions of background concentrations were then added to the estimated local concentrations. In a previous study, validations of total concentrations against measurements showed R2 values of 0.87, 0.65 and 0.93 for PM10, PM2.5, and BC, respectively [24].

The resulting concentrations were lastly added to each study participant's home addresses and annual exposures estimated accounting for any changes in address during the study period 1990-2011.

Confounders

Cohort-specific models were adjusted for potential confounding by including sex, calendar year, sub-cohort (in Stockholm), smoking status (current, former, never smoker), alcohol consumption (in Stockholm and Umeå; daily, weekly, seldom, never), physical activity (in Gothenburg and Umeå: sedentary, moderate, intermediate or vigorous; in Stockholm: once a month or less/>1 hour per week, about once a month/1 hour per week, 3 times a week or more/>2 hour per week), marital status (single, married or living with partner, no answer), socio-economic index by occupation (in Gothenburg (PPS) and Stockholm: blue collar, low and intermediate white collar and self-employed, high level white collar and self-employed professionals with academic degrees, no answer), education level (in Gothenburg (MONICA), Stockholm and Umeå: primary school or less, up to secondary school or equivalent, university degree or more, no answer), occupation status (in Stockholm and Umeå: gainfully employed, unemployed/not gainfully employed, retired, no answer). Area level socioeconomic status was estimated for each cohort member's residence from the mean neighborhood individual income in persons of working age by Small Areas for Market Statistics (SAMS) provided by Statistics Sweden for the calendar year 1994.

Statistical methods

Cox-proportional hazard models were used to estimate hazard ratios of mortality associated with PM and BC in cohort-specific analyses. Age was used as the underlying time variable for the baseline hazard. The regression model included adjustment for calendar year, baseline information on confounders as well as area level socio-economy. Since the Stockholm CEANS cohort consisted of four sub-cohorts that differed in age and time of recruitment, separate baseline hazards were used for the analyses within this cohort. We censured individuals at death by other causes, the end of the study period or time of permanent emigration from the study areas. Associations with PM and BC were assessed using two exposure windows; a moving average over 1-5 years preceding the event (lag1-5) and 6-10 years preceding the event (lag6-10). For inclusion, annual mean concentrations were required for at least 80% of the time window. These moving averages were used as time-varying exposures and therefore considers both between and within individual contrast during the study period. Besides these time windows, associations were also assessed with concentrations at the year of recruitment. Hazard ratios and 95% confidence intervals (CI) were expressed per 10, 5 and 1 μg/m^3 for PM10, PM2.5 and BC, respectively, as well as per interquartile ranges (IQRs) across the three study areas.

Meta-analyses of cohort-specific estimates were performed using random effects [33]. Heterogeneity between cohort estimates were assessed by the I2 statistic and statistically tested for deviations from homogeneity by a Chi-square test based on the Cochran's Q statistic.

Results

For peer review only - http://bmjopen.bmj.com/site/about/guidelines.xhtml

Participant characteristics

The total number of individuals included in our meta-analysis was 68,679, with the number of individuals included in each cohort presented in table 1. Total number of deaths by natural causes was 7344. Age at recruitment differed largely between cohorts with the highest median in the Gothenburg PPS cohort and lowest in VIP Umeå, 69 compared with 40 years. By design only males were recruited to the Gothenburg PPS cohort, whereas the other three cohorts had a slightly larger proportion of women compared with men. Baseline current smoking was highest in the Gothenburg PPS cohort and lowest in Stockholm CEANS and VIP Umeå, 39% compared with about 20%. Leisure time physical activity had a different categorization within the Stockholm CEANS cohort but it can be concluded that there was a larger proportion of individuals reporting a sedentary lifestyle at baseline in this cohort. The cohorts in Gothenburg had the largest proportion of physically active individuals, with higher frequency and intensity. Alcohol consumption was more frequent in Stockholm CEANS compared with VIP Umeå. University education was most common in Stockholm CEANS and VIP Umeå (above 30%) and less common in Gothenburg. A larger proportion of individuals were gainfully employed in the younger VIP Umeå than in Stockholm CEANS, 84% compared with 66%. Blue-collar occupation was more likely within the Gothenburg PPS cohort compared with Stockholm CEANS.

Particle concentrations

Particle concentrations of PM10, PM2.5 and BC all had the highest mean and IQR in the Gothenburg cohorts and the lowest in VIP Umeå (figure 1-3). In total, means of lag1-5 PM10, PM2.5 and BC were; 11.3, 6.9 and 0.6 μg/m^3, respectively. Lag1-5 concentrations were on average lower than lag6-10 concentrations due to a decreasing trend in concentrations during the study period. The Pearson correlation coefficient between lag 1-5 concentrations of PM10 and PM2.5 was 0.88, PM10 and BC 0.71, and between PM2.5 and BC 0.75.

Associations with mortality

Meta-estimates showed increased risks associated with PM10, PM2.5 and BC in relation to natural mortality; for lag1-5 17% (95% CI 6-31) increased risk per 10 μg/m^3 PM10, 13% (95% CI -9-40) increased risk per 5 μg/m^3 PM2.5 and 9% (95% CI 0-18) increased risk per 1 μg/m^3 BC (figure 4-6; crude estimates in supplementary material figure S1a-c). The statistical uncertainty was however considerable for the combined risk increase associated with PM2.5 and the confidence interval included 1. For PM10 and PM2.5 higher risk estimates were found for lag1-5 concentrations compared with lag6-10, whereas for BC, the risk estimates were the same. Increased risks were estimated for all cohorts except for Gothenburg MONICA, where the hazard ratio estimate for natural mortality was below 1.

The risk estimates for CVD mortality associated with PM10 and PM2.5 appeared higher compared with natural mortality; 19 (95% CI 1-40) and 23% (95% CI 3-48) increased risks for lag1-5, per 10 and 5 μg/m^3, respectively (figure 7-9; crude estimates in supplementary material figure S2a-b). The increased risks associated with BC seemed lower for CVD mortality than for natural mortality for lag1-5, but similar for lag6-10 (figure 9; crude estimates in supplementary material figure S2c).

The HRs per IQR for natural mortality were higher for PM2.5 and PM10 than for BC; 6, 5 and 2% increased risk, respectively. The same order per IQR was found for CVD mortality; 10, 5 and 1% for PM2.5, PM10 and BC, respectively.

No associations between PM and lung cancer or non-malignant respiratory mortality were observed (supplementary material figure S3-4a-c). Increased risk was, however, observed for mortality by

For peer review only - http://bmjopen.bmj.com/site/about/guidelines.xhtml

other causes (that is other than CVD, lung cancer, respiratory disease, and external causes) with the same magnitude as observed for natural and CVD mortality (supplementary material figure S5a-c).

For comparison with previous studies, associations were also assessed with residential PM concentrations for the recruitment year. These estimates were in general lower compared with those with lag1-5 and lag6-10 concentrations (supplementary material figure S6a-b). Estimated risk increases for natural mortality were 7 (95% CI -4-18), 8 (95% CI -2-20) and 3% (95% CI -4-11) in association with PM10, PM2.5 and BC, per 10, 5 and 1 μg/m^3, respectively.

The heterogeneity between cohort hazard ratio estimates in relation to precision was in general very low. Heterogeneity was, however, observed for associations with lag1-5 PM2.5 and lag6-10 BC in relation to natural mortality, although non-of these were statistically significant (figure 4-9).

Discussion

We observed in four Swedish cohorts located in Gothenburg, Stockholm and Umeå increased risks associated with long-term residential outdoor levels of PM10, PM2.5 and BC in relation to natural and CVD mortality. PM10 was most consistently associated with natural and CVD mortality, whereas larger uncertainties were found for the associations between PM2.5 and CVD mortality and BC and natural mortality. Risk estimates associated with PM10 and PM2.5 appeared larger for exposures during the last 5 years in comparison to 6-10 years prior. For BC the associated risks were the same for both exposure windows in relation to natural mortality but higher for the exposure window 6-10 years prior in relation to CVD mortality. Within a previous meta-analysis of these cohorts, an association was found between BC and stroke incidence [22]. No association was, however, found between PM10 or PM2.5 and incidence in ischemic heart disease.

The observed 17% increased risk per 10 μg/m^3 PM10 in relation to natural mortality is high compared with most studies (e.g. the 6% increased risk found by Hoek et al. 2013 [2]) but similar to the recent findings within a Danish cohort presenting 12% increased risk (95% CI 3-22%) associated with a 15-year moving average of residential ambient concentrations [6]. Their estimate for CVD mortality was 30% (95% CI 11-53%) which was higher than our meta-estimate of 19%. For PM2.5 our observed risk increase by 13% per 5 μg/m^3 for natural mortality is also in agreement with the Danish estimate of 13% (95 CI 5-21%). As for PM10 the risk increase for CVD mortality associated with PM2.5 was again lower in our study, 23% compared to 29% (95% CI 13-47%). The average PM10 and PM2.5 exposure levels were 2.2 and 2.6 times higher in the Danish cohort compared to our study (lag 1-10 time-weighted concentrations from the Danish cohort compared with lag 1-5 moving averages in our study).

Using less detailed assessments of outdoor residential exposures, a meta-analysis of 22 cohorts within the ESCAPE project reported a borderline significant 4% (95% CI 0-9%) increased risk of natural-cause mortality for a 10 μg/m^3 difference in annual PM10 at the year of recruitment [4]. No association with PM10 was, however, observed for deaths in ischemic heart disease or myocardial infarction, whereas a suggested association was found in relation to deaths in cerebrovascular disease; 22% (95% CI -9-63%) increased risk per 10 μg/m^3 [5]. The findings for mortality associated with PM2.5 have been heterogeneous, however, the meta-estimate among the ESCAPE cohorts showed a 7% (95% CI 2-13%) increased risk per 5 μg/m^3 of natural mortality and 21% (95% CI -13-69%) increased risk of death in cerebrovascular disease. As for PM10 no association was found in relation to death in ischemic heart disease or myocardial infarction. Within earlier studies; a review and meta-analysis by Hoek et al. (2013) found 6% (95% CI 4-8%) and 15% (95% CI 4-27) increased risks of all-cause mortality and cardiovascular mortality per 5 μg/m^3, respectively. A stronger association between PM and mortality with cardiovascular causes compared to all natural mortality has also

For peer review only - http://bmjopen.bmj.com/site/about/guidelines.xhtml

been reported by others [1], and is in agreement with our findings on CVD mortality; and most apparent in relation to exposures during the last 5 years.

In accordance with our meta-estimate for BC the Danish cohort reported a risk increase by 9% (95% CI 4-15%) per 1 µg/m3 for all-cause mortality [6]. An increased risk by 16% (95% CI 5-27%) was also observed for CVD mortality, higher than the 5% increased risk found in our study. In the review by Hoek et al. (2013) elemental carbon was found associated with a 6% (95% CI 5-7%) increased risk of all-cause mortality per 1 µg/m^3.

The lack of association between PM and non-malignant respiratory mortality in our study is in agreement with results from both the Danish study [6], the ESCAPE study [7] and a large cohort study in Rome [34]. Previous studies have, however, found an increased risk of lung cancer incidence associated with both PM10 and PM2.5 [35 36]. The statistical power for these outcomes was low in our meta-analyses due to the limited number of deaths in lung cancer and other respiratory causes.

The observed heterogeneity of PM associated risk for premature mortality between studies is possibly related to differences in particle composition, housing with differences in particle infiltration, population characteristics, and the minor differences in confounding adjustments. The air pollution dispersion model used for the exposure assessment in our study has a high spatial resolution, and for the follow up period it was also updated with new regional and local emission inventories as well as PM measurements to provide annual mean temporal PM concentrations. A higher precision in the exposure estimate have been found to produce higher relative health risk estimates [13]. The mortality risk associated with PM2.5 has recently been reported to be higher for local sources compared with distant sources, but with less precision in relative risk estimates, indicating the importance of high spatial resolution exposure data to successfully model differences in mortality [37]. The meta-analysis results in this study and the results presented by Hvidtfeldt et al. (2019) both generally provided higher hazard ratio estimates compared with previous mortality studies. Both studies also used moving averages based on annual mean concentrations at the place of residence. Besides exposure data with a high precision such a moving average over relevant time windows may be advantageous compared with residential concentrations at the year of recruitment or a time-weighted average exposure during the follow-up. These two studies also adjusted both for individual data on confounders and neighbourhood level data on socioeconomics.

Strengths and limitations

The strength of this study includes the state-of-the-art air pollution modelling used for exposure assessment. Several factors that affect the local dispersion of air pollution particles were considered including meteorological conditions, amount of traffic including vehicle types and speed, which affect exhaust and wear emissions, the width of the street, and the height of neighbouring building. This facilitates models with a high spatial resolution providing the ability to estimate an individual's exposure at the residential address. Temporal differences for each address were also accounted for by repeatedly updating the exposure model during the period of follow up. Our modelled concentrations have showed a good agreement when validated against measured concentrations by monitoring stations in city centres [24]. Due to uncertainties in data input to the dispersion model, and since modelled PM levels at the home address do not equal true personal exposure, exposure misclassifications at the individual level will nevertheless arise. True personal exposure would for instance include indoor particle concentrations at the place of residence, at the workplace, and during commuting. During the years of follow up there has been a decreasing trend in both total concentrations of PM and age specific mortality. It was therefore important to adjust for calendar

For peer review only - http://bmjopen.bmj.com/site/about/guidelines.xhtml

year in the Cox models. Limitations also include the lack of information on noise exposure and green space near the home address which could be confounders or effect-modifiers of air pollution effects.

Another strength of this study is the large number of cohort participants and ability to adjust for a large set of confounders at both individual and area level, however information on confounders from questionnaires were only available at baseline. Even though we expect the cohort participants with age ranging from 40 years to have fairly stable lifestyles and habits, such baseline variables may change, for example due to disease. No clear heterogeneity in associations between cohorts was observed, however, with only four cohorts the test had low statistical power to reject the null hypothesis of homogeneity.

This study additionally contributes with hazard ratio estimates between PM and mortality in the lower range of exposure with PM2.5 ranging between 4.0 to 20.4 μg/m^3 and PM10 ranging between 6.3 to 41.9 μg/m^3. Even though this is lower than the current European Union standards of 25 μg/m^3 for PM2.5 and (with a few exceptions) lower than 40 μg/m^3 for PM10, the study found higher relative risks for premature mortality compared with most previous studies even though mean exposures in those studies have been higher.

Conclusions

This study of four cohorts with relatively low exposures showed increased risks of mortality in association with long-term exposure to PM10, PM2.5 and BC, and thus strongly support long-term air pollution associated effects. Since high risk increases were observed even at relatively low exposures, the findings are relevant for future decisions concerning air quality policies.

For peer review only - http://bmjopen.bmj.com/site/about/guidelines.xhtml

Tables

Table 1. Descriptive statistics of the study participants in each cohort

		Gothenburg, MONICA	Gothenburg, PPS	Stockholm, CEANS	Umeå, VIP
Participants (n)		4,500	5,850	22,314	42,580
Baseline data collection (calendar years)		1985,1990,1995	1970-74	1992-2004	1992-2014
Age at enrolment (years; median (range))		46 (25–66)	51 (47–56)	56 (35-104)	40 (40-50)
Women (%)		52	0	58	52
Smoking status (%)	Current smoker	29	39	22	19
	Former smoker	N/A	33	36	30
	Never smoker	65	27	40	49
	Missing data	5	0	1.8	1
Physical activity (%)	Once a month or less/<1h/week	N/A	N/A	61	N/A
	About once a month/~1h/week	N/A	N/A	26	N/A
	3 times a week or more/>2h/week	N/A	N/A	7.6	N/A
	Missing data	N/A	N/A	4.6	N/A
Leisure time physical activity (%)	Sedentary	18	24	N/A	36
	Moderate	62	59	N/A	42
	Intermediate and vigorous	18	17	N/A	22
	Missing data	2	1	N/A	2
Alcohol consumption (%)	Daily	N/A	N/A	6.7	2
	Weekly	N/A	N/A	55	16
	Seldom	N/A	N/A	31	42
	Never	N/A	N/A	5.6	1
	Missing data	N/A	N/A	1.8	39
Married/living with partner (%)	No	21	14	29	23
	Yes	47	86	70	77
	Missing data	32	<0.1	1.3	1
Education level (%)	Primary school or less	13	N/A	30	30
	Up to secondary school or equivalent	32[a]	N/A	36	30
	University degree & more	20	N/A	31	40
	Missing data	35	N/A	2.8	1

For peer review only

For peer review only - http://bmjopen.bmj.com/site/about/guidelines.xhtml

Occupation (%)	Gainfully employed	N/A	N/A	66	85
	Unemployed/not gainfully empolyed	N/A	N/A	5.8	6
	Retired	N/A	N/A	27	4
	Missing data	N/A	N/A	1.3	4
Socioeconomic index by occupation (%)	Blue collar	N/A	48	27	N/A
	Low-and intermediate white collar and self-employed	N/A	23	51	N/A
	High level white collar and self-employed professional with academic degrees	N/A	30	18	N/A
	Missing data	N/A	0	4.4	N/A
Mean income by SAMS 1994 (SEK)		154,780	148,602	N/A	130,000
Mean income by SAMS 2009 (SEK)		N/A	N/A	303,910	N/A

CEANS, Cardiovascular Effects of Air Pollution and Noise Study; MONICA, Multinational Monitoring of Trends and Determinants in Cardiovascular Diseases; N/A, not available; PPS, Primary Prevention Study; SAMS, Small Areas for Market Statistics; SEK, Swedish Krona; VIP, Västerbotten Intervention Programme.

a. Includes technical training

For peer review only - http://bmjopen.bmj.com/site/about/guidelines.xhtml

Legends to figures

Figure 1. Boxplot and density function of PM10 concentrations, in total and separate for each cohort. Tabulated values are quartile limits together with means and standard deviations.

Figure 2. Boxplot and density function of PM2.5 concentrations, in total and separate for each cohort. Tabulated values are quartile limits together with means and standard deviations.

Figure 3. Boxplot and density function of BC concentrations, in total and separate for each cohort. Tabulated values are quartile limits together with means and standard deviations.

Figure 4. Adjusted hazard ratios of natural mortality associated with PM10 within each cohort and by random effect meta-analyses estimates. Exposures were assessed by moving average residential concentrations within the exposure windows last 5 years and 6 to 10 years prior.

Figure 5. Adjusted hazard ratios of natural mortality associated with PM2.5 within each cohort and by random effect meta-analyses estimates. Exposures were assessed by moving average residential concentrations within the exposure windows last 5 years and 6 to 10 years prior.

Figure 6. Adjusted hazard ratios of natural mortality associated with BC within each cohort and by random effect meta-analyses estimates. Exposures were assessed by moving average residential concentrations within the exposure windows last 5 years and 6 to 10 years prior.

Figure 7. Adjusted hazard ratios of CVD mortality associated with PM10 within each cohort and by random effect meta-analyses estimates. Exposures were assessed by moving average residential concentrations within the exposure windows last 5 years and 6 to 10 years prior.

Figure 8. Adjusted hazard ratios of CVD mortality associated with PM2.5 within each cohort and by random effect meta-analyses estimates. Exposures were assessed by moving average residential concentrations within the exposure windows last 5 years and 6 to 10 years prior.

Figure 9. Adjusted hazard ratios of CVD mortality associated with BC within each cohort and by random effect meta-analyses estimates. Exposures were assessed by moving average residential concentrations within the exposure windows last 5 years and 6 to 10 years prior.

For peer review only

Author contributions

JNS performed the meta-analysis and wrote with support from BF the first draft manuscript. JNS, EA, NA and GS conducted the cohort analyses. DS, KE, LG, CJ were responsible for dispersion modelling of particles. PW, AR, DR, KL, AL, PM and PKM and were responsible for collection of cohort data. GP, BF, LB, TB, JNS, GS, LS, PL, EA and NA designed the epidemiological study. All authors contributed to the interpretation of the results, finalization of the manuscript, and approved the final manuscript.

Competing interests

All authors have completed the ICMJE uniform disclosure form at www.icmje.org/coi_disclosure.pdf and declare: no support from any organization other than those listed above for the submitted work; no financial relationships with any organizations that might have an interest in the submitted work in the previous three years; no other relationships or activities that could appear to have influenced the submitted work.

Funding

This study was funded by the Swedish Environmental Protection Agency as part of the Swedish Clean Air and Climate Research Program (grant NV-06576-13). BF and JNS was also supported by NordForsk grant no. 75007. The GOT-MONICA and PPS cohorts were supported by the Swedish Research Council, 2013-5187 (SIMSAM). The SDPP cohort was funded by the Stockholm County Council, the Swedish Research Council, the Swedish Diabetes Association, the Novo Nordisk Scandinavia, and GlaxoSmithKline. The 60YO cohort was funded by the Stockholm County Council and the Swedish Research Council. The Swedish Twin Registry is managed by Karolinska Institutet and receives funding through the Swedish Research Council under the grant no. 2017-00641. Data collection in SALT was supported by a grant from the National Institutes of Health (NIH), grant no. 1R01 AG08724. The VIP-Umeå cohort was funded by the Västerbotten County Council. PLL was supported by funding from the Swedish Research Council for Health, Working Life and Welfare (FORTE) 2015-00917 and Karolinska Institute's Strategic Research Area in Epidemiology (SFO-EPI). LB was financed by grants from the Swedish state under the agreement between the Swedish government and the county councils: the ALF-agreement (74580).

Data sharing statement

No additional data available

For peer review only

References

1. Brook RD, Rajagopalan S, Pope CA, 3rd, et al. Particulate matter air pollution and cardiovascular disease: An update to the scientific statement from the American Heart Association. *Circulation* 2010;121(21):2331-78. doi: 10.1161/CIR.0b013e3181dbece1
2. Hoek G, Krishnan RM, Beelen R, et al. Long-term air pollution exposure and cardio- respiratory mortality: a review. *Environ Health-Glob* 2013;12 doi: Artn 43 10.1186/1476-069x-12-43
3. Stanaway JD, Afshin A, Gakidou E, et al. Global, regional, and national comparative risk assessment of 84 behavioural, environmental and occupational, and metabolic risks or clusters of risks for 195 countries and territories, 1990–2017: a systematic analysis for the Global Burden of Disease Study 2017. *The Lancet* 2018;392(10159):1923-94. doi: 10.1016/S0140-6736(18)32225-6
4. Beelen R, Raaschou-Nielsen O, Stafoggia M, et al. Effects of long-term exposure to air pollution on natural-cause mortality: an analysis of 22 European cohorts within the multicentre ESCAPE project. *Lancet* 2014;383(9919):785-95. doi: 10.1016/S0140-6736(13)62158-3
5. Beelen R, Stafoggia M, Raaschou-Nielsen O, et al. Long-term exposure to air pollution and cardiovascular mortality: an analysis of 22 European cohorts. *Epidemiology* 2014;25(3):368-78. doi: 10.1097/EDE.0000000000000076
6. Hvidtfeldt UA, Sorensen M, Geels C, et al. Long-term residential exposure to PM2.5, PM10, black carbon, NO2, and ozone and mortality in a Danish cohort. *Environment international* 2019;123:265-72. doi: 10.1016/j.envint.2018.12.010
7. Dimakopoulou K, Samoli E, Beelen R, et al. Air Pollution and Nonmalignant Respiratory Mortality in 16 Cohorts within the ESCAPE Project. *American journal of respiratory and critical care medicine* 2014;189(6):684-96. doi: 10.1164/rccm.201310-1777OC
8. Luben TJ, Nichols JL, Dutton SJ, et al. A systematic review of cardiovascular emergency department visits, hospital admissions and mortality associated with ambient black carbon. *Environment international* 2017;107:154-62. doi: 10.1016/j.envint.2017.07.005
9. Gan WQ, Koehoorn M, Davies HW, et al. Long-term exposure to traffic-related air pollution and the risk of coronary heart disease hospitalization and mortality. *Environmental health perspectives* 2011;119(4):501-7. doi: 10.1289/ehp.1002511
10. Ostro B, Hu JL, Goldberg D, et al. Associations of Mortality with Long-Term Exposures to Fine and Ultrafine Particles, Species and Sources: Results from the California Teachers Study Cohort. *Environmental health perspectives* 2015;123(6):549-56. doi: 10.1289/ehp.1408565
11. Ostro B, Lipsett M, Reynolds P, et al. Long-Term Exposure to Constituents of Fine Particulate Air Pollution and Mortality: Results from the California Teachers Study. *Environmental health perspectives* 2010;118(3):363-69. doi: 10.1289/ehp.0901181
12. Baxter LK, Dionisio KL, Burke J, et al. Exposure prediction approaches used in air pollution epidemiology studies: Key findings and future recommendations. *J Expo Sci Env Epid* 2013;23(6):654-59. doi: 10.1038/jes.2013.62
13. Vodonos A, Awad YA, Schwartz J. The concentration-response between long-term PM2.5 exposure and mortality; A meta-regression approach. *Environmental research* 2018;166:677-89. doi: https://doi.org/10.1016/j.envres.2018.06.021
14. Christidis T, Erickson AC, Pappin AJ, et al. Low concentrations of fine particle air pollution and mortality in the Canadian Community Health Survey cohort. *Environmental health : a global access science source* 2019;18(1):84-84. doi: 10.1186/s12940-019-0518-y
15. Pinault LL, Weichenthal S, Crouse DL, et al. Associations between fine particulate matter and mortality in the 2001 Canadian Census Health and Environment Cohort. *Environmental research* 2017;159:406-15. doi: 10.1016/j.envres.2017.08.037 [published Online First: 2017/08/30]
16. Pinault L, Tjepkema M, Crouse DL, et al. Risk estimates of mortality attributed to low concentrations of ambient fine particulate matter in the Canadian community health survey

For peer review only - http://bmjopen.bmj.com/site/about/guidelines.xhtml

cohort. *Environmental health : a global access science source* 2016;15:18-18. doi: 10.1186/s12940-016-0111-6

17. Shi L, Zanobetti A, Kloog I, et al. Low-Concentration PM2.5 and Mortality: Estimating Acute and Chronic Effects in a Population-Based Study. *Environmental health perspectives* 2016;124(1):46-52. doi: 10.1289/ehp.1409111 [published Online First: 2015/06/03]
18. WILHELMSEN L, BERGLUND G, ELMFELDT D, et al. The multifactor primary prevention trial in Göteborg, Sweden. *European heart journal* 1986;7(4):279-88. doi: 10.1093/oxfordjournals.eurheartj.a062065
19. WILHELMSEN L, JOHANSSON S, ROSENGREN A, et al. Risk factors for cardiovascular disease during the period 1985–1995 in Göteborg, Sweden. The GOT-MONICA Project. *Journal of Internal Medicine* 1997;242(3):199-211. doi: 10.1046/j.1365-2796.1997.00163.x
20. WHO Monica Project Principal Investigators. The world health organization monica project (monitoring trends and determinants in cardiovascular disease): A major international collaboration. *Journal of Clinical Epidemiology* 1988;41(2):105-14. doi: https://doi.org/10.1016/0895-4356(88)90084-4
21. Korek MJ, Bellander TD, Lind T, et al. Traffic-related air pollution exposure and incidence of stroke in four cohorts from Stockholm. *Journal of exposure science & environmental epidemiology* 2015;25(5):517-23. doi: 10.1038/jes.2015.22
22. Ljungman PLS, Andersson N, Stockfelt L, et al. Long-Term Exposure to Particulate Air Pollution, Black Carbon, and Their Source Components in Relation to Ischemic Heart Disease and Stroke. *Environmental health perspectives* 2019;127(10):107012. doi: 10.1289/EHP4757
23. Norberg M, Wall S, Boman K, et al. The Västerbotten Intervention Programme: background, design and implications. *Global health action* 2010;3 doi: 10.3402/gha.v3i0.4643 [published Online First: 2010/03/27]
24. Segersson D, Eneroth K, Gidhagen L, et al. Health Impact of PM10, PM2.5 and Black Carbon Exposure Due to Different Source Sectors in Stockholm, Gothenburg and Umea, Sweden. *International Journal of Environmental Research and Public Health* 2017;14(7) doi: Artn 742 10.3390/Ijerph14070742
25. Berkowicz R. OSPM - A Parameterised Street Pollution Model. *Environmental monitoring and assessment* 2000;65(1):323-31. doi: 10.1023/A:1006448321977
26. Hausberger S, Rexeis M, Zallinger M. Emission Factors from the Model PHEM for the HBEFA Version 3., Institute for internal combustion engines and thermodynamics, Graz University of Technology. 2009
27. Ilias V, Leonidas N, Zissis S. Methodology for the Quantification of Road Transport PM-Emissions, Using Emission Factors or Profiles. Deliverable D1.1.2, Updated February 2013. TRANSPHORM, http://www.transphorm.eu/Portals/51/Documents/Deliverables/New%20Deliverables/D1.1.2_updated.pdf [accessed 26 October 2018]. 2013
28. Denby BR, Sundvor I, Johansson C, et al. A coupled road dust and surface moisture model to predict non-exhaust road traffic induced particle emissions (NORTRIP). Part 2: Surface moisture and salt impact modelling. *Atmos Environ* 2013;81:485-503. doi: https://doi.org/10.1016/j.atmosenv.2013.09.003
29. Denby BR, Sundvor I, Johansson C, et al. A coupled road dust and surface moisture model to predict non-exhaust road traffic induced particle emissions (NORTRIP). Part 1: Road dust loading and suspension modelling. *Atmos Environ* 2013;77:283-300. doi: https://doi.org/10.1016/j.atmosenv.2013.04.069
30. Omstedt G, Bringfelt B, Johansson C. A model for vehicle-induced non-tailpipe emissions of particles along Swedish roads. *Atmos Environ* 2005;39(33):6088-97. doi: https://doi.org/10.1016/j.atmosenv.2005.06.037
31. Jalkanen JP, Johansson L, Kukkonen J, et al. Extension of an assessment model of ship traffic exhaust emissions for particulate matter and carbon monoxide. *Atmos Chem Phys* 2012;12(5):2641-59. doi: 10.5194/acp-12-2641-2012

For peer review only - http://bmjopen.bmj.com/site/about/guidelines.xhtml

32. Forsberg B, Hansson HC, Johansson C, et al. Comparative health impact assessment of local and regional particulate air pollutants in Scandinavia. *Ambio* 2005;34(1):11-9. [published Online First: 2005/03/26]
33. Dersimonian R, Laird N. Metaanalysis in Clinical-Trials. *Control Clin Trials* 1986;7(3):177-88. doi: Doi 10.1016/0197-2456(86)90046-2
34. Cesaroni G, Badaloni C, Gariazzo C, et al. Long-term exposure to urban air pollution and mortality in a cohort of more than a million adults in Rome. *Environmental health perspectives* 2013;121(3):324-31. doi: 10.1289/ehp.1205862
35. Hamra GB, Guha N, Cohen A, et al. Outdoor Particulate Matter Exposure and Lung Cancer: A Systematic Review and Meta-Analysis. *Environmental health perspectives* 2014;122(9):906-11. doi: doi:10.1289/ehp/1408092
36. Raaschou-Nielsen O, Andersen ZJ, Beelen R, et al. Air pollution and lung cancer incidence in 17 European cohorts: prospective analyses from the European Study of Cohorts for Air Pollution Effects (ESCAPE). *The Lancet Oncology* 2013;14(9):813-22. doi: https://doi.org/10.1016/S1470-2045(13)70279-1
37. Lefler JS, Higbee JD, Burnett RT, et al. Air pollution and mortality in a large, representative U.S. cohort: multiple-pollutant analyses, and spatial and temporal decompositions. *Environmental health : a global access science source* 2019;18(1):101. doi: 10.1186/s12940-019-0544-9

For peer review only - http://bmjopen.bmj.com/site/about/guidelines.xhtml

Figure 1. Boxplot and density function of PM10 concentrations, in total and separate for each cohort. Tabulated values are quartile limits (Q1-Q3) and interquartile ranges (IQR:s) together with means and standard deviations.

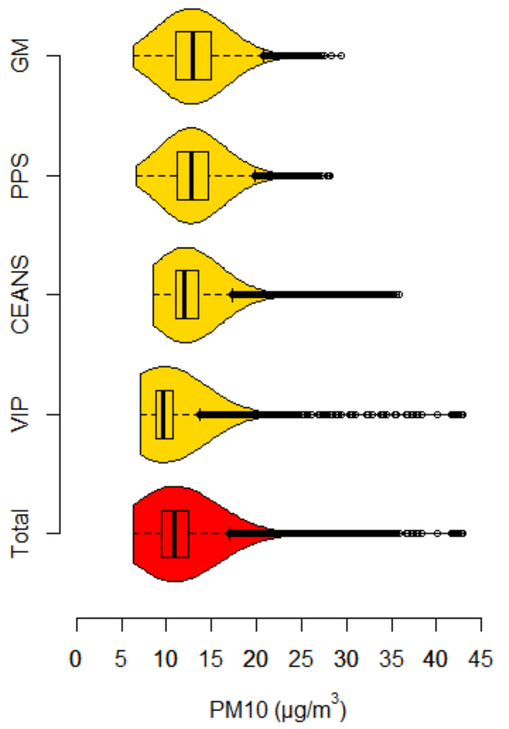

	Median (Q1-Q3)	IQR	Mean (sd)
GM	12.9 (11.1-14.9)	3.9	13.1 (2.8)
PPS	12.8 (11.3-14.7)	3.5	13.1 (2.6)
CEANS	12.0 (11.1-13.6)	2.5	12.5 (2.2)
VIP	9.6 (8.9-10.8)	1.9	9.9 (1.5)
Total	10.9 (9.5-12.5)	3.0	11.3 (2.4)

CEANS, Cardiovascular Effects of Air Pollution and Noise Study; MONICA, Multinational Monitoring of Trends and Determinants in Cardiovascular Diseases; PPS, Primary Prevention Study; VIP, Västerbotten Intervention Programme

For peer review only - http://bmjopen.bmj.com/site/about/guidelines.xhtml

Figure 2. Boxplot and density function of PM2.5 concentrations, in total and separate for each cohort. Tabulated values are quartile limits (Q1-Q3) and interquartile ranges (IQR:s) together with means and standard deviations.

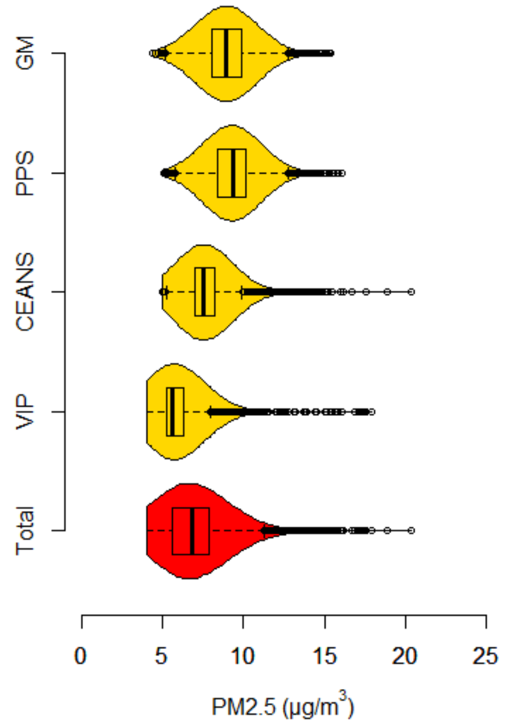

	Median (Q1-Q3)	IQR	Mean (sd)
GM	9.0 (8.0-9.9)	1.9	9.0 (1.4)
PPS	9.3 (8.4-10.2)	1.7	9.3 (1.3)
CEANS	7.5 (7.0-8.2)	1.2	7.6 (0.9)
VIP	5.7 (5.3-6.3)	1.1	5.8 (0.8)
Total	6.8 (5.6-7.9)	2.3	6.9 (1.5)

CEANS, Cardiovascular Effects of Air Pollution and Noise Study; MONICA, Multinational Monitoring of Trends and Determinants in Cardiovascular Diseases; PPS, Primary Prevention Study; VIP, Västerbotten Intervention Programme

Figure 3. Boxplot and density function of BC concentrations, in total and separate for each cohort. Tabulated values are quartile limits (Q1-Q3) and interquartile ranges (IQR:s) together with means and standard deviations.

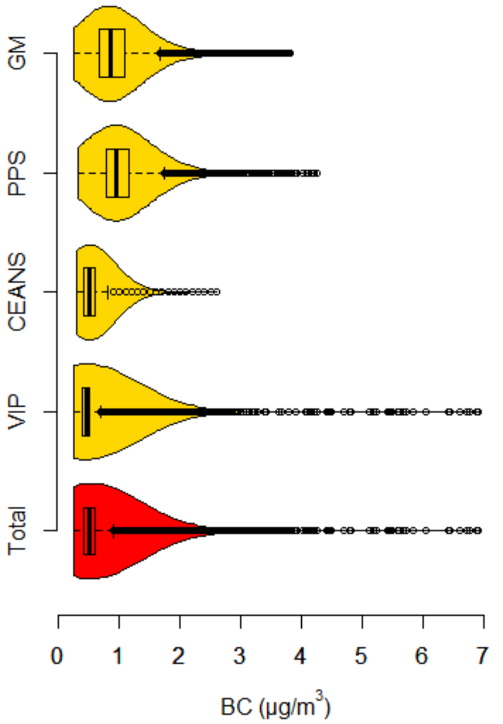

Median (Q1-Q3)	IQR	Mean (sd)
0.8 (0.7-1.1)	0.4	0.9 (0.4)
0.9 (0.8-1.2)	0.4	1.0 (0.4)
0.5 (0.4-0.6)	0.2	0.5 (0.2)
0.4 (0.4-0.5)	0.1	0.5 (0.1)
0.5 (0.4-0.6)	0.2	0.6 (0.3)

CEANS, Cardiovascular Effects of Air Pollution and Noise Study; MONICA, Multinational Monitoring of Trends and Determinants in Cardiovascular Diseases; PPS, Primary Prevention Study; VIP, Västerbotten Intervention Programme

ew only

Figure 4. Adjusted hazard ratios of natural mortality associated with PM10 within each cohort and by random effect meta-analyses estimates. Exposures were assessed by moving average residential concentrations within the exposure windows last 5 years and 6 to 10 years prior.

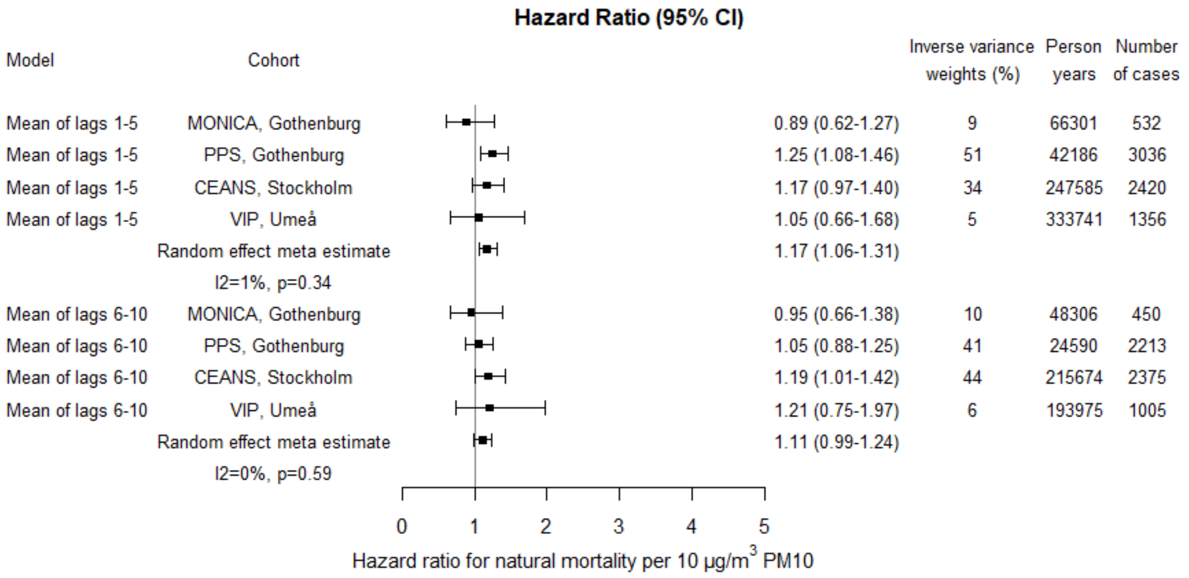

er review only

For peer review only - http://bmjopen.bmj.com/site/about/guidelines.xhtml

Figure 5. Adjusted hazard ratios of natural mortality associated with PM2.5 within each cohort and by random effect meta-analyses estimates. Exposures were assessed by moving average residential concentrations within the exposure windows last 5 years and 6 to 10 years prior.

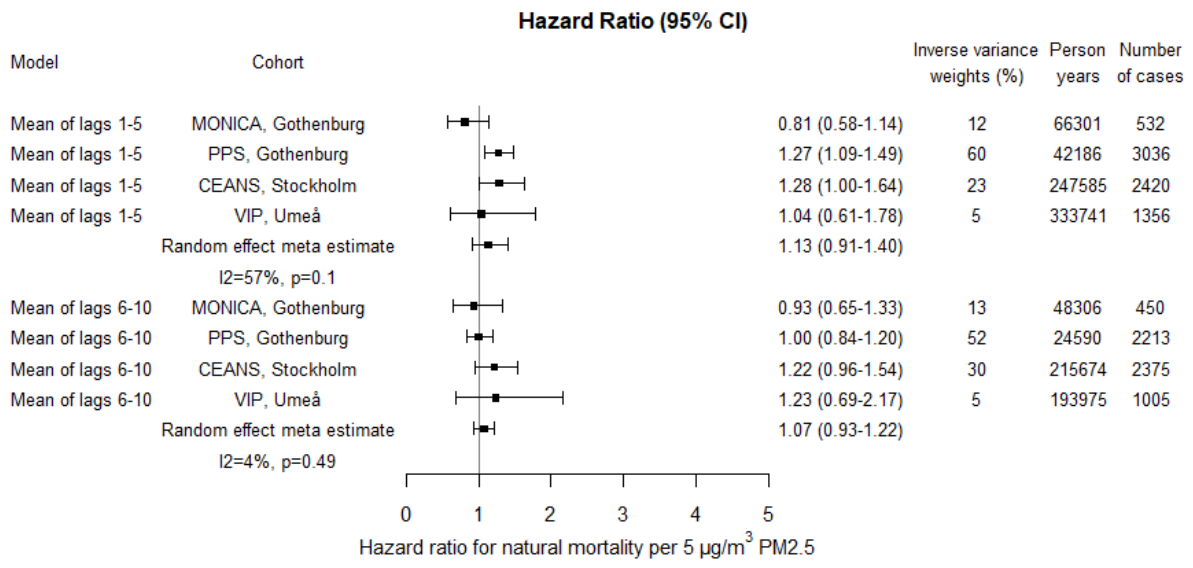

For peer review only

Figure 6. Adjusted hazard ratios of natural mortality associated with BC within each cohort and by random effect meta-analyses estimates. Exposures were assessed by moving average residential concentrations within the exposure windows last 5 years and 6 to 10 years prior.

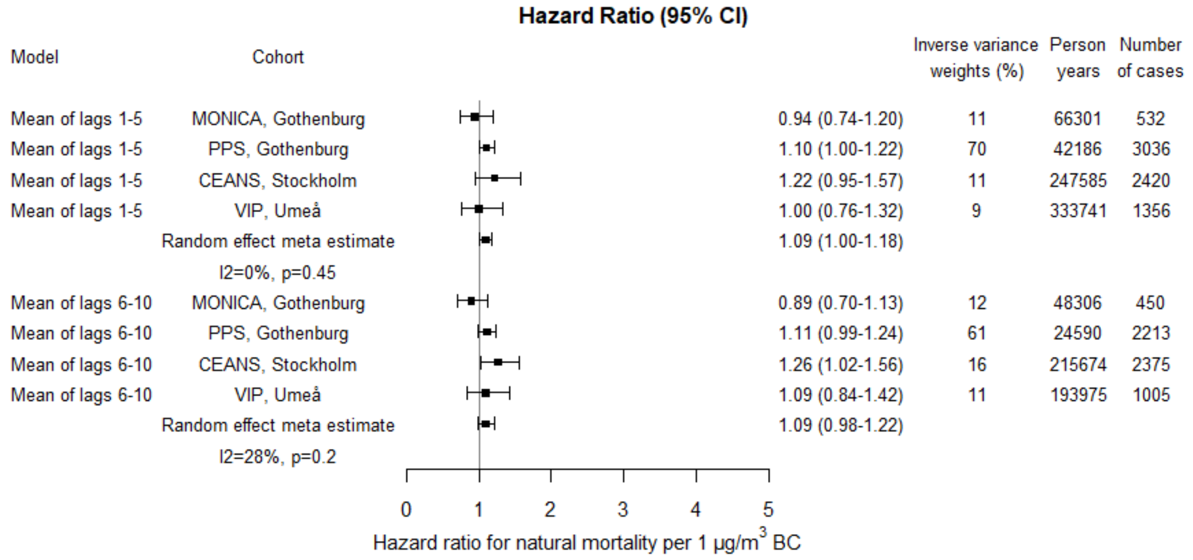

For peer review only

Figure 7. Adjusted hazard ratios of CVD mortality associated with PM10 within each cohort and by random effect meta-analyses estimates. Exposures were assessed by moving average residential concentrations within the exposure windows last 5 years and 6 to 10 years prior.

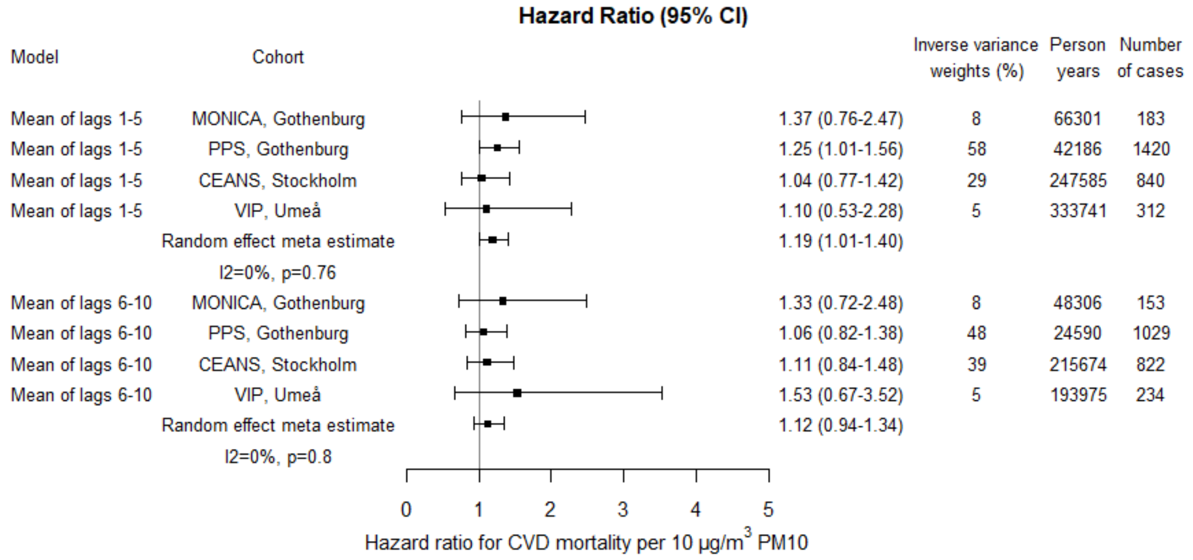

For peer review only

Figure 8. Adjusted hazard ratios of CVD mortality associated with PM2.5 within each cohort and by random effect meta-analyses estimates. Exposures were assessed by moving average residential concentrations within the exposure windows last 5 years and 6 to 10 years prior.

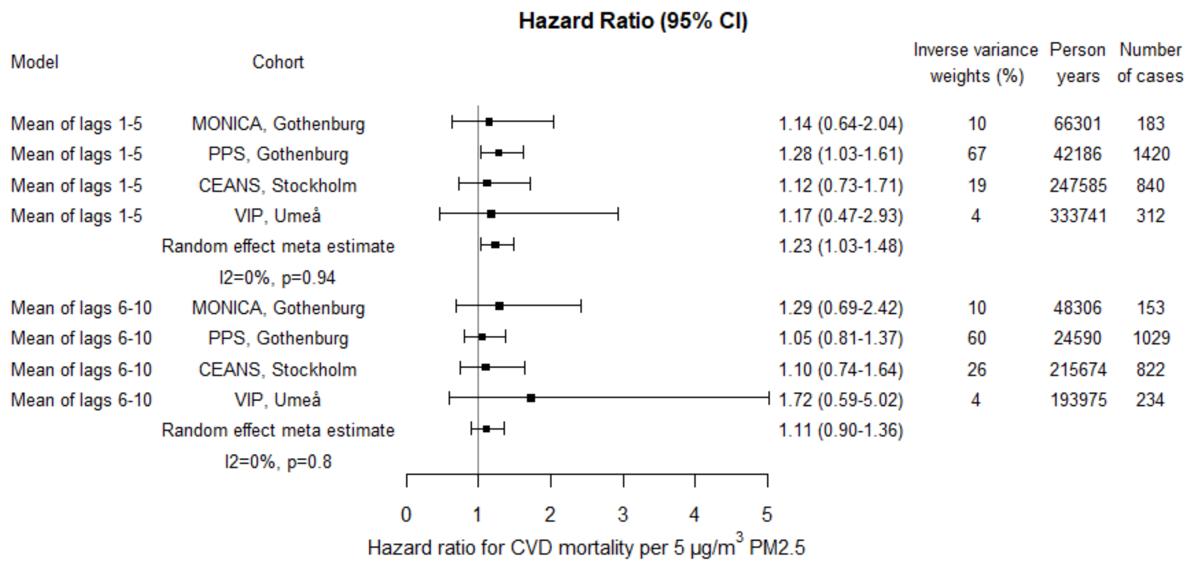

For peer review only

Figure 9. Adjusted hazard ratios of CVD mortality associated with BC within each cohort and by random effect meta-analyses estimates. Exposures were assessed by moving average residential concentrations within the exposure windows last 5 years and 6 to 10 years prior.

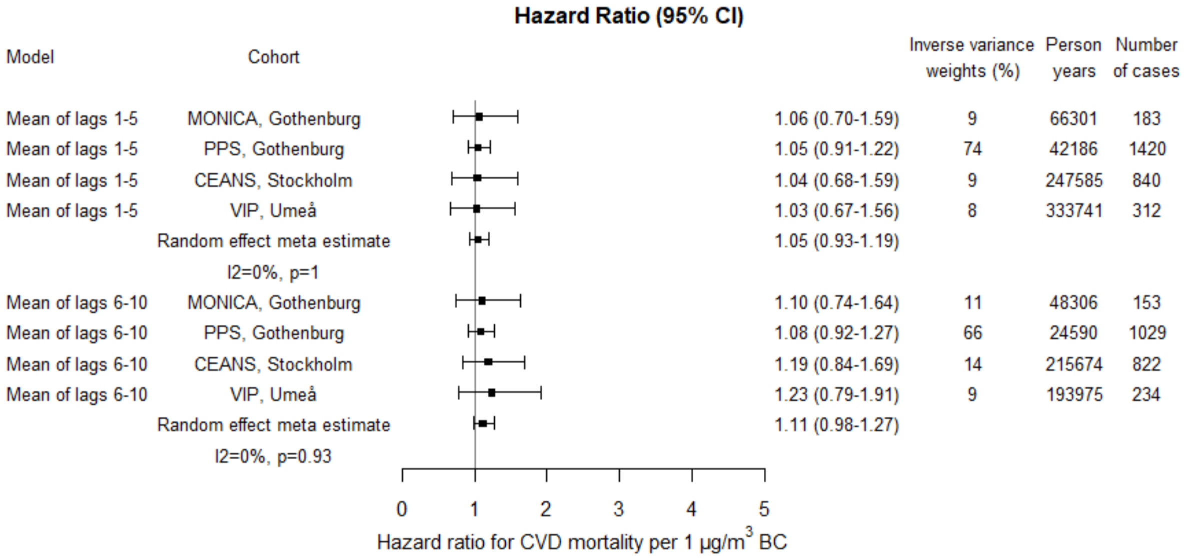

For peer review only - http://bmjopen.bmj.com/site/about/guidelines.xhtml

Figure S1a-c. Crude hazard ratios of natural mortality associated with a) PM10, b) PM2.5 and c) BC within each cohort and by random effect meta-analyses estimates. Exposures were assessed by moving average residential concentrations within the exposure windows last 5 years and 6 to 10 years prior.

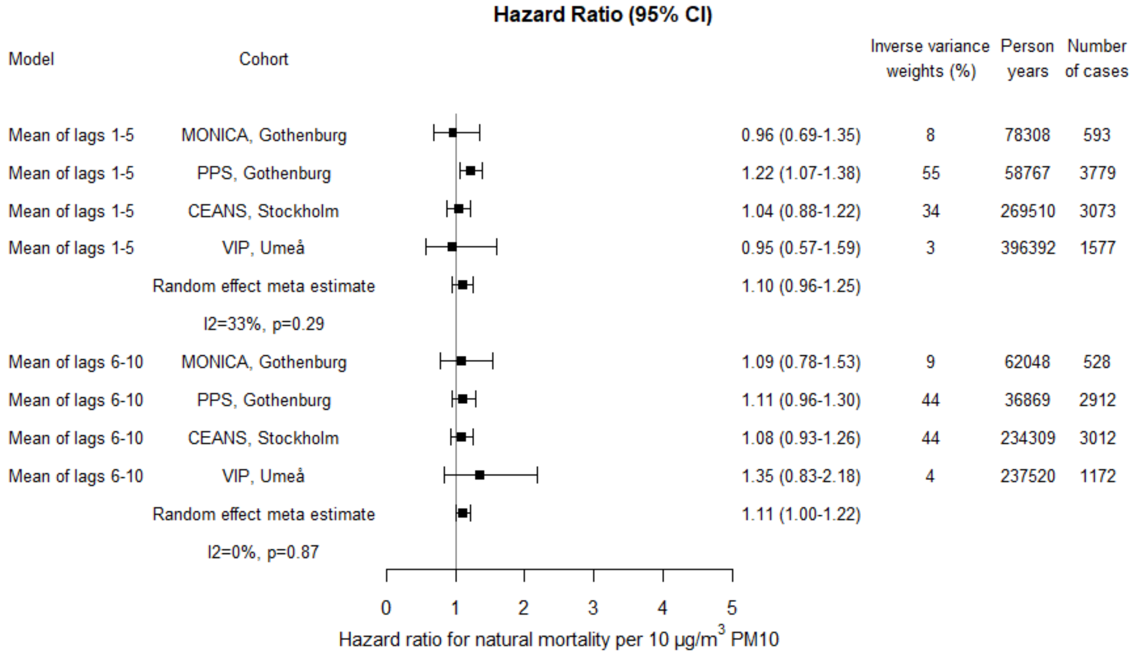

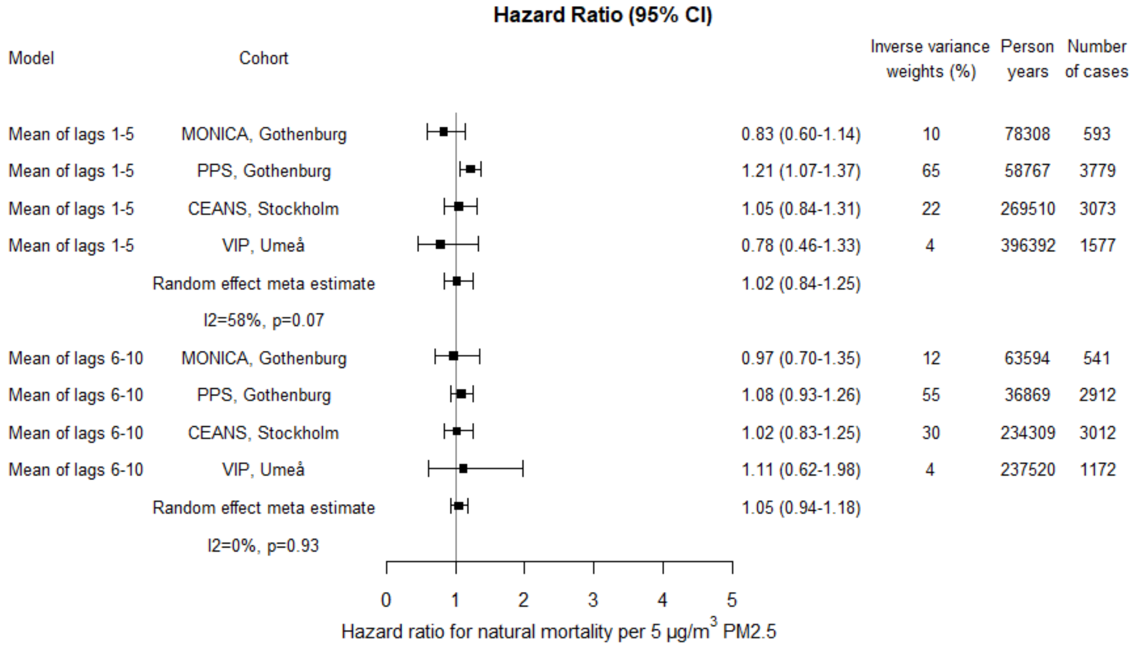

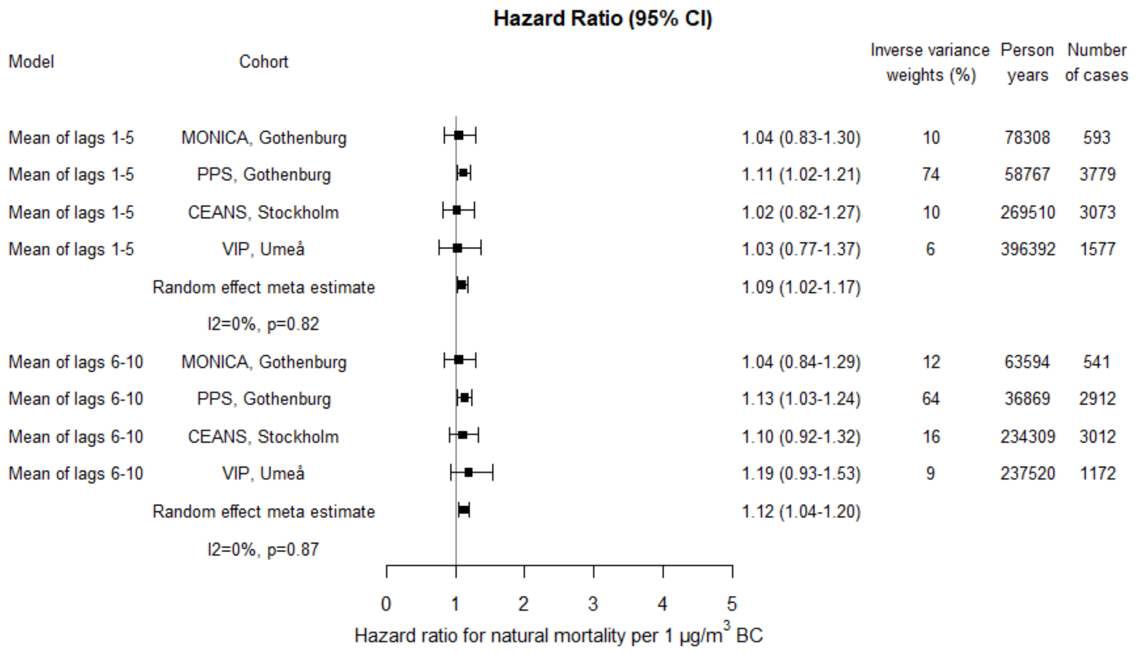

For peer review only

Figure S2a-c. Crude hazard ratios of CVD mortality associated with a) PM10, b) PM2.5 and c) BC within each cohort and by random effect meta-analyses estimates. Exposures were assessed by moving average residential concentrations within the exposure windows last 5 years and 6 to 10 years prior.

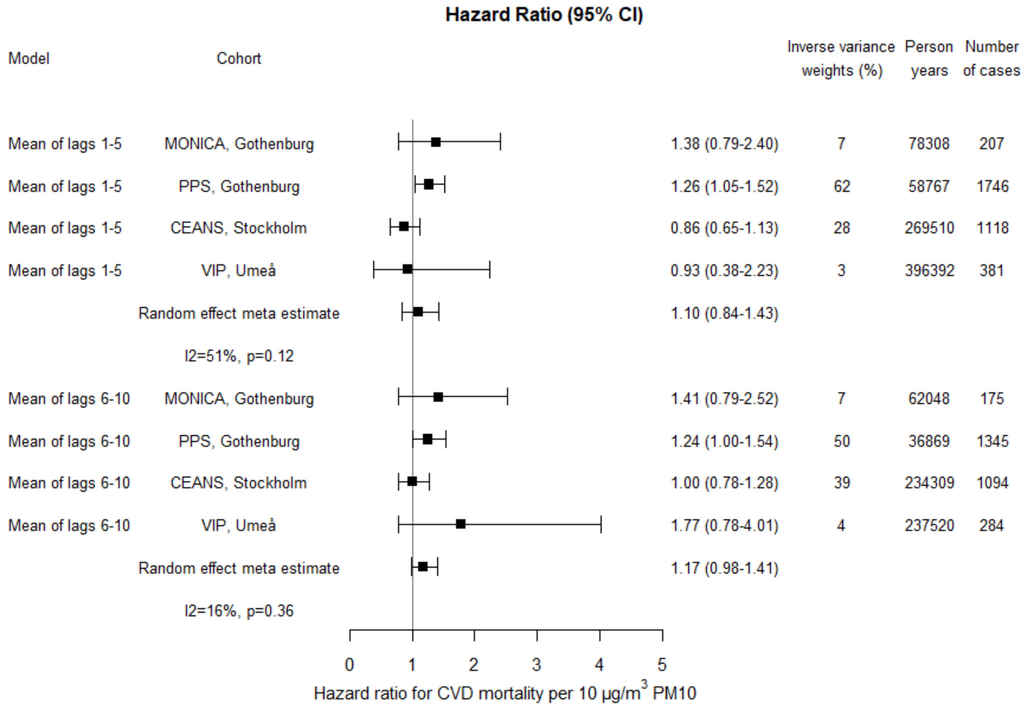

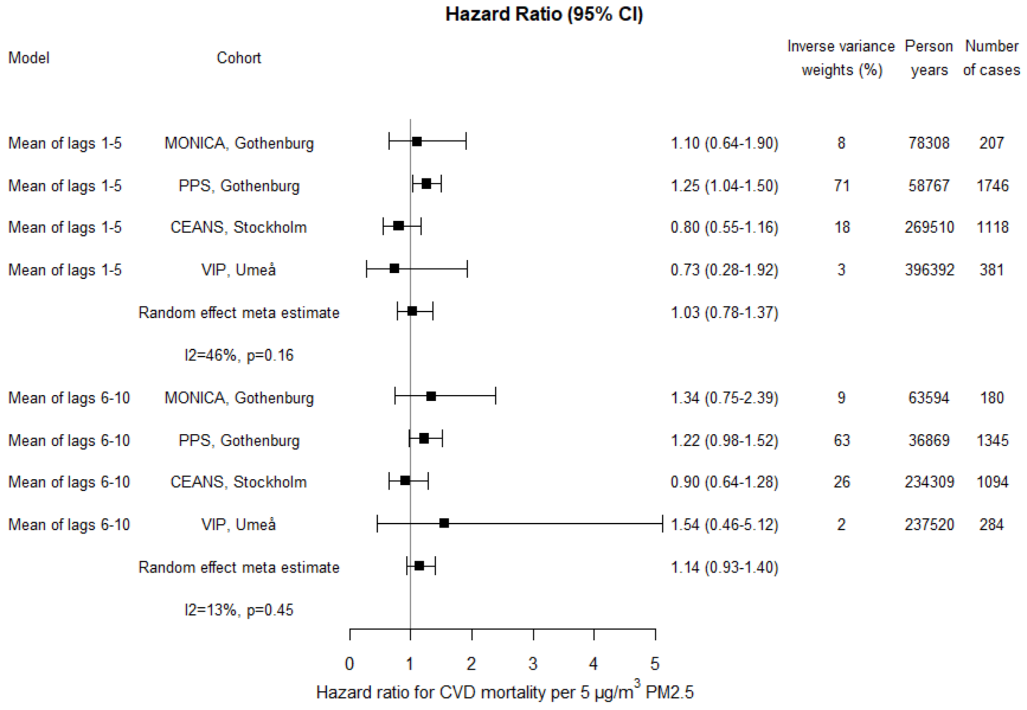

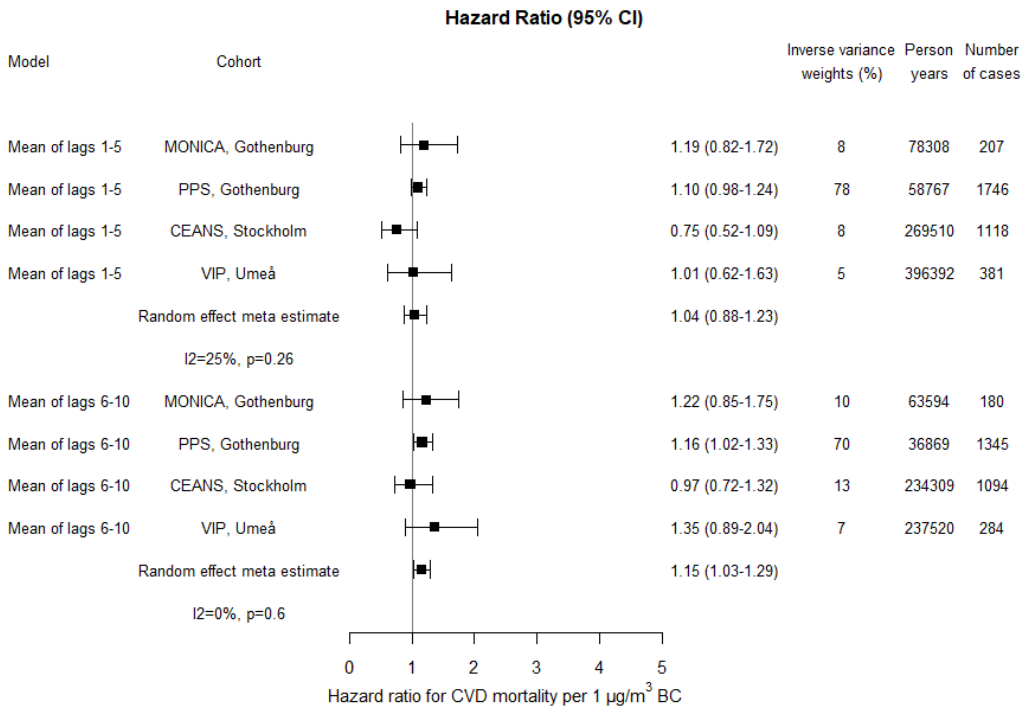

For peer review only

Figure S3a-c. Adjusted hazard ratios of lung cancer mortality associated with a) PM10, b) PM2.5 and c) BC within each cohort and by random effect meta-analyses estimates. Exposures were assessed by moving average residential concentrations within the exposure windows last 5 years and 6 to 10 years prior.

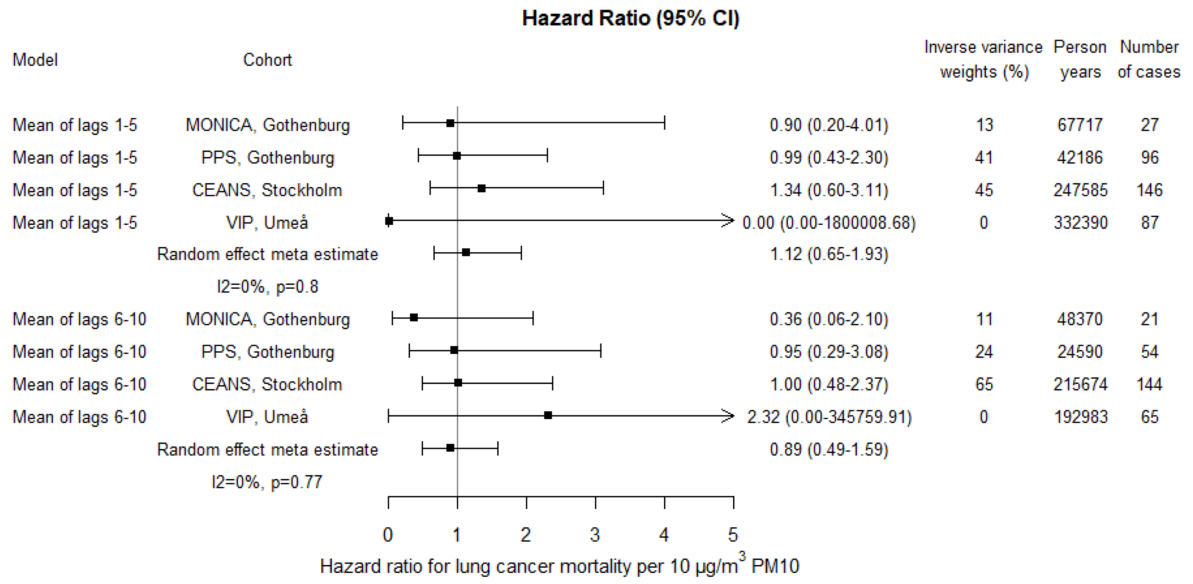

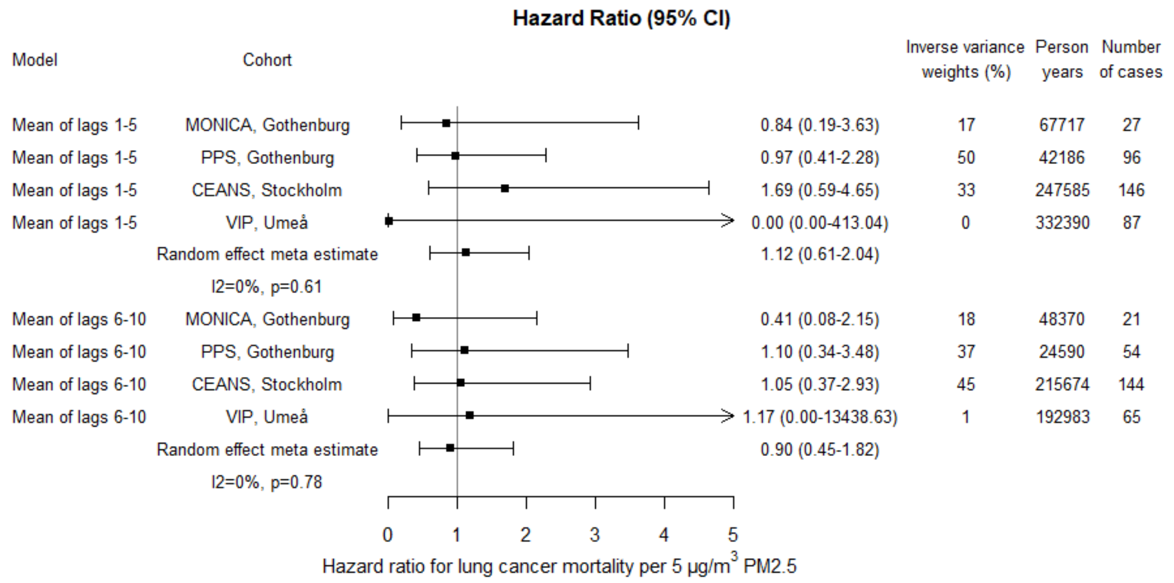

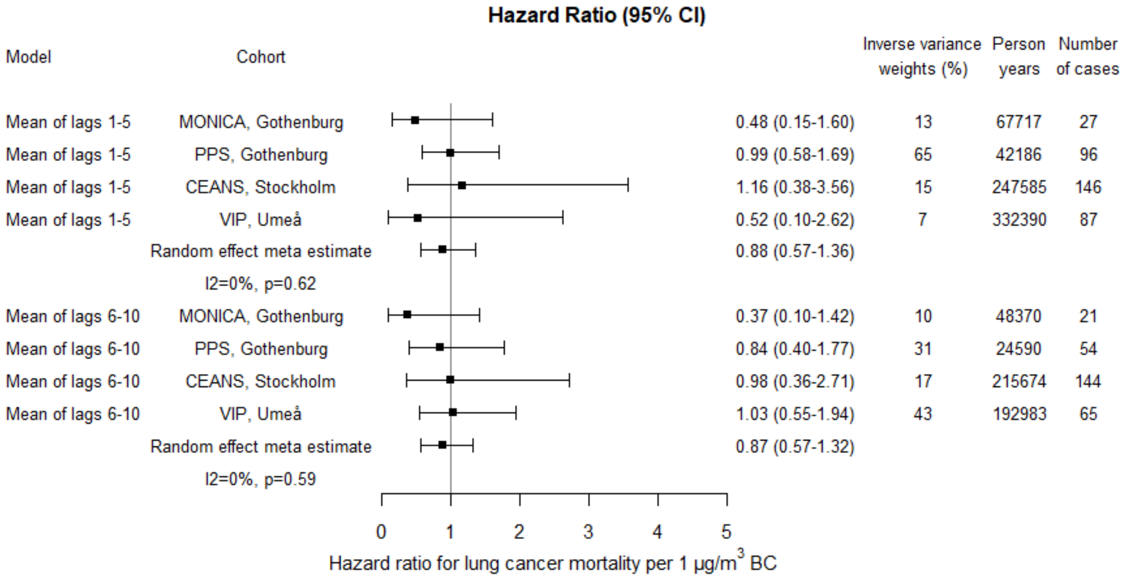

peer review only

Figure S4a-c. Adjusted hazard ratios of respiratory mortality associated with a) PM10, b) PM2.5 and c) BC within each cohort and by random effect meta-analyses estimates. Exposures were assessed by moving average residential concentrations within the exposure windows last 5 years and 6 to 10 years prior.

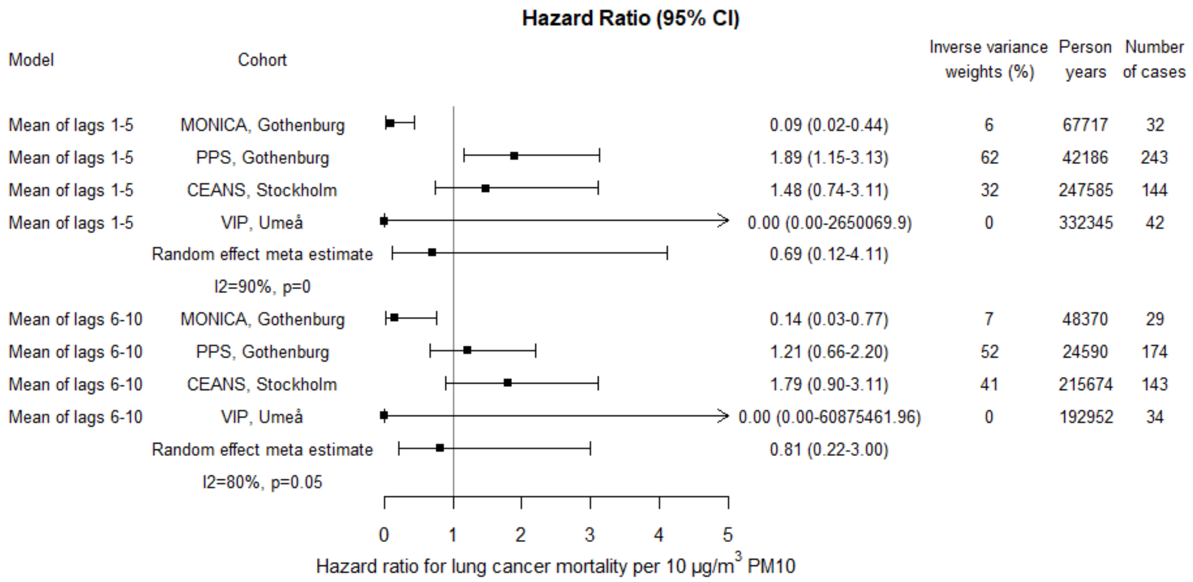

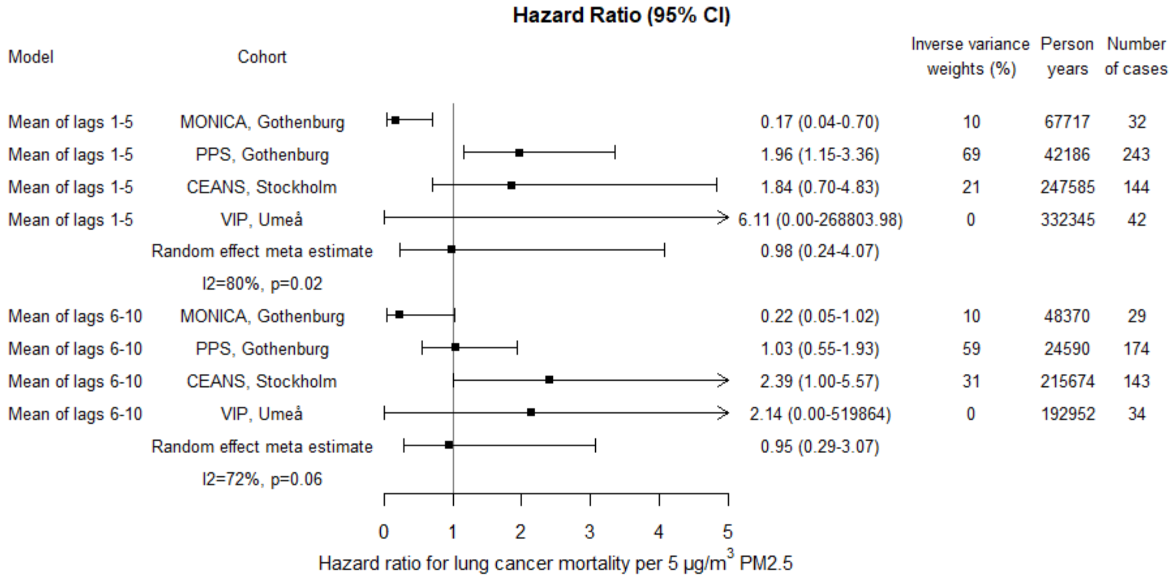

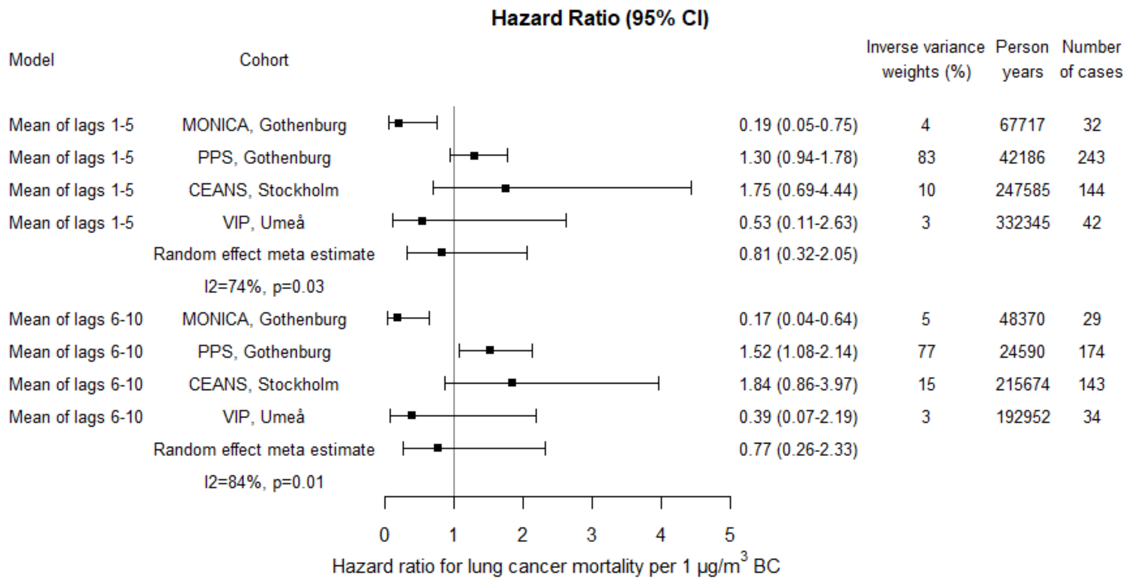

peer review only

Figure S5a-c. Adjusted hazard ratios of mortality by other casues (not CVD, respiratory, lung cancer or external causes) associated with a) PM10, b) PM2.5 and c) BC within each cohort and by random effect meta-analyses estimates. Exposures were assessed by moving average residential concentrations within the exposure windows last 5 years and 6 to 10 years prior.

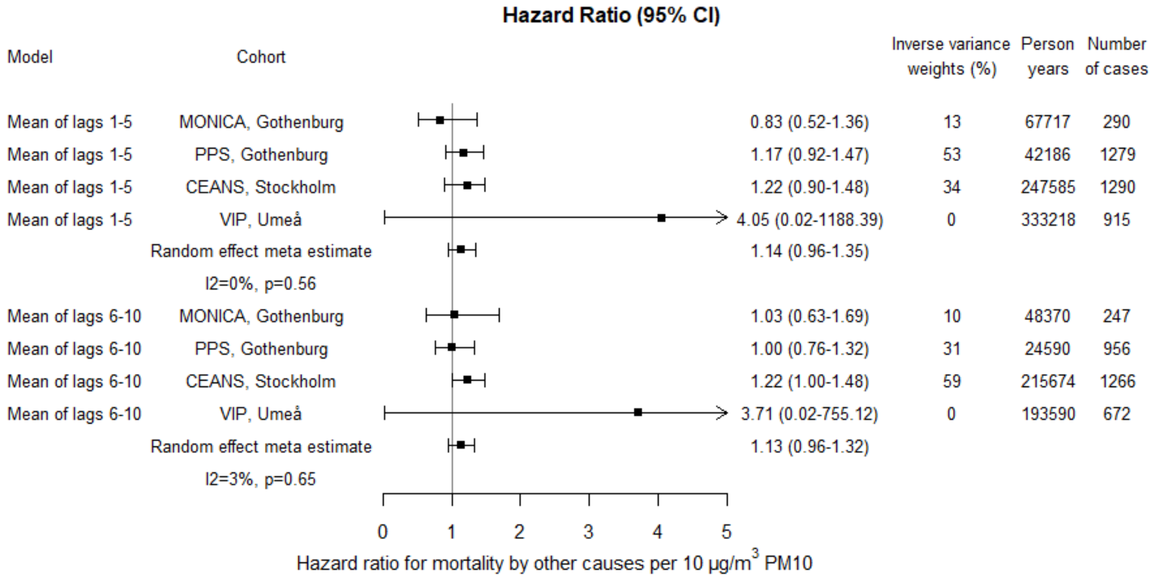

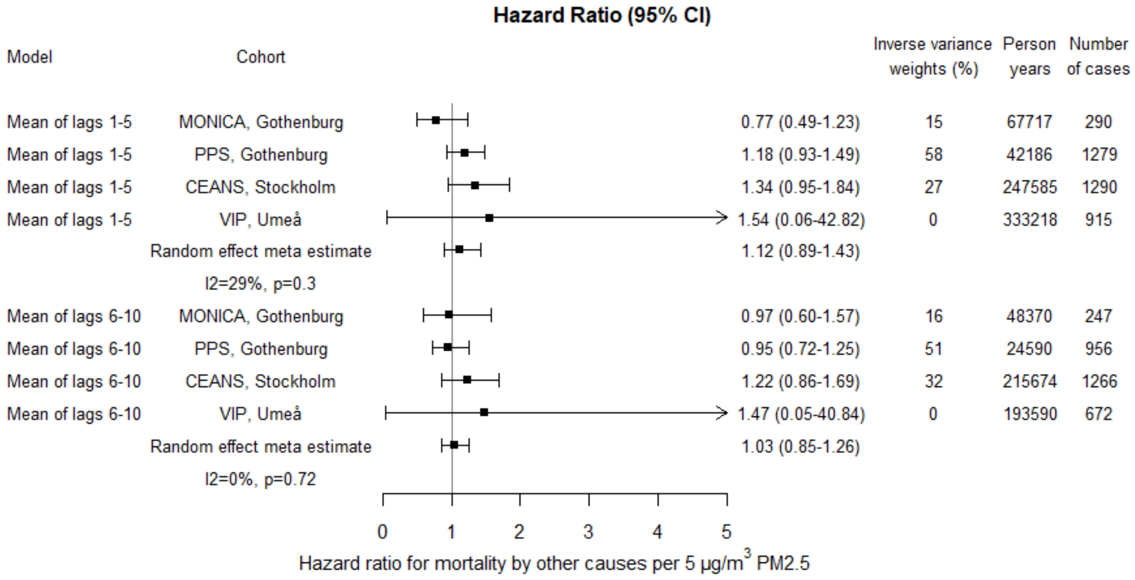

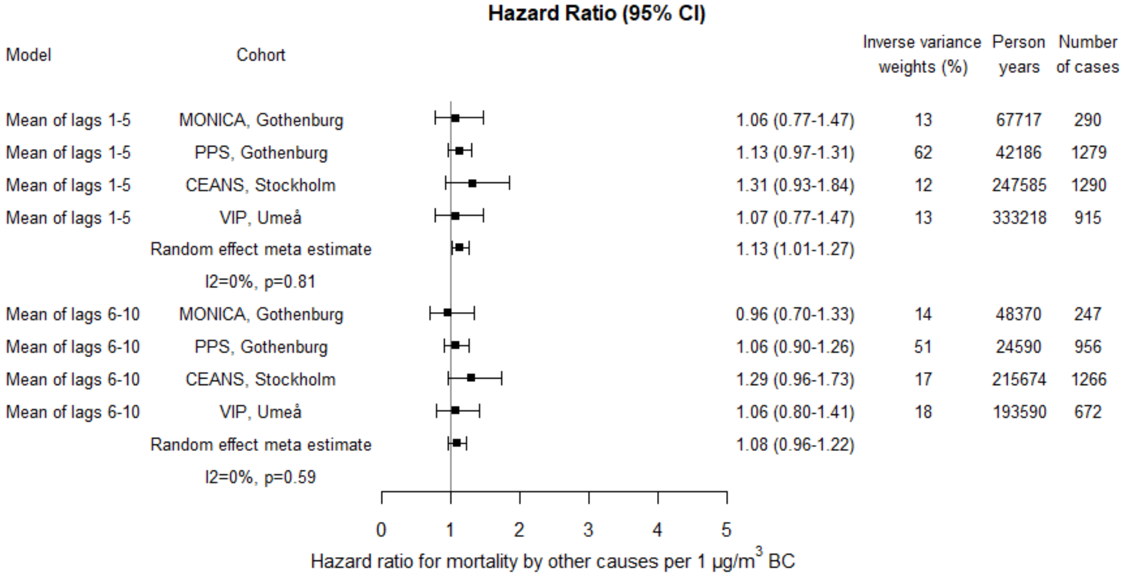

peer review only

Figure S6a-b. Adjusted hazard ratios of a) natural mortality and b) CVD mortality associated with PM10, PM2.5 and BC, within each cohort and by random effect meta-analyses estimates. Exposures were assessed by the residential concentration at the year of recruitment.

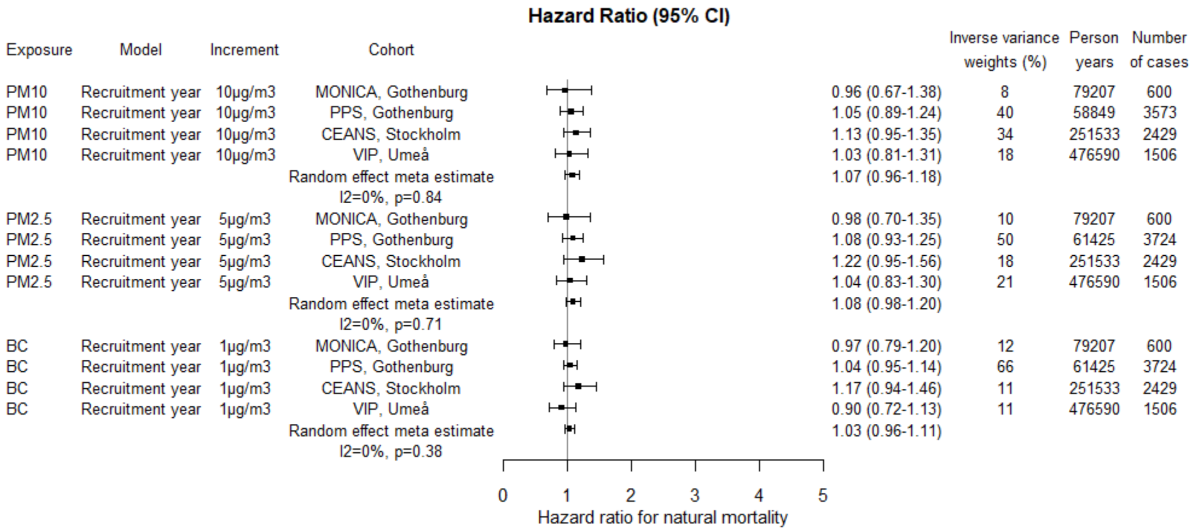

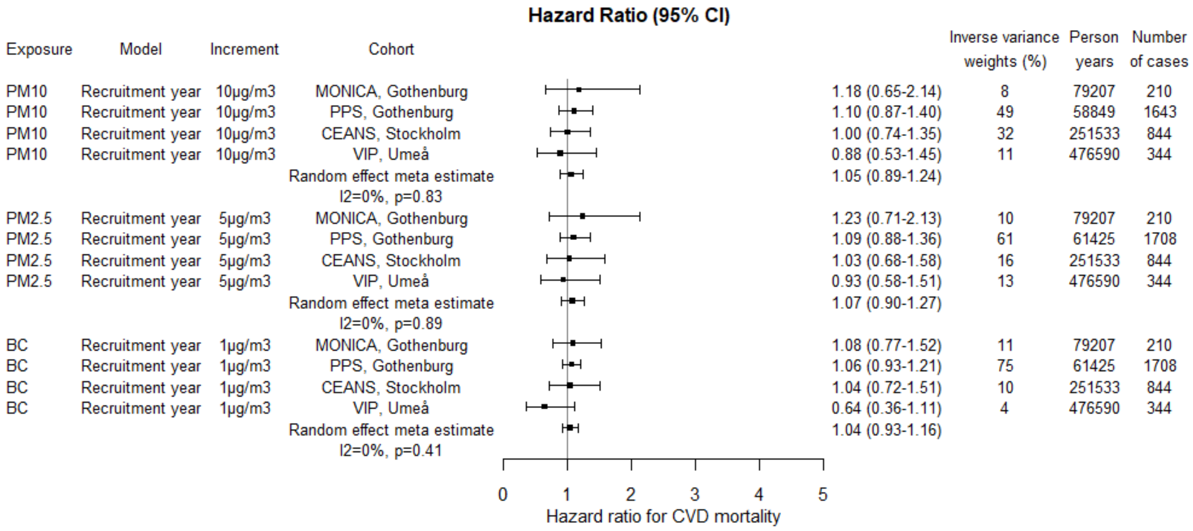

STROBE 2007 (v4) Statement—Checklist of items that should be included in reports of *cohort studies*

Section/Topic	Item #	Recommendation	Reported on page #
**Title and abstract**	1	(*a*) Indicate the study's design with a commonly used term in the title or the abstract	2
		(*b*) Provide in the abstract an informative and balanced summary of what was done and what was found	2
**Introduction**			
Background/rationale	2	Explain the scientific background and rationale for the investigation being reported	3
Objectives	3	State specific objectives, including any prespecified hypotheses	3-4
**Methods**			
Study design	4	Present key elements of study design early in the paper	4
Setting	5	Describe the setting, locations, and relevant dates, including periods of recruitment, exposure, follow-up, and data collection	4-6
Participants	6	(*a*) Give the eligibility criteria, and the sources and methods of selection of participants. Describe methods of follow-up	4-5
		(*b*) For matched studies, give matching criteria and number of exposed and unexposed	
Variables	7	Clearly define all outcomes, exposures, predictors, potential confounders, and effect modifiers. Give diagnostic criteria, if applicable	5-6
Data sources/ measurement	8*	For each variable of interest, give sources of data and details of methods of assessment (measurement). Describe comparability of assessment methods if there is more than one group	*5*
Bias	9	Describe any efforts to address potential sources of bias	10
Study size	10	Explain how the study size was arrived at	4-7
Quantitative variables	11	Explain how quantitative variables were handled in the analyses. If applicable, describe which groupings were chosen and why	5-6
Statistical methods	12	(*a*) Describe all statistical methods, including those used to control for confounding	6
		(*b*) Describe any methods used to examine subgroups and interactions	
		(*c*) Explain how missing data were addressed	6
		(*d*) If applicable, explain how loss to follow-up was addressed	6
		(*e*) Describe any sensitivity analyses	
**Results**			

For peer review only

Participants	13*	(a) Report numbers of individuals at each stage of study—eg numbers potentially eligible, examined for eligibility, confirmed eligible, included in the study, completing follow-up, and analysed	7
		(b) Give reasons for non-participation at each stage	
		(c) Consider use of a flow diagram	
Descriptive data	14*	(a) Give characteristics of study participants (eg demographic, clinical, social) and information on exposures and potential confounders	7
		(b) Indicate number of participants with missing data for each variable of interest	7
		(c) Summarise follow-up time (eg, average and total amount)	7
Outcome data	15*	Report numbers of outcome events or summary measures over time	7
Main results	16	(*a*) Give unadjusted estimates and, if applicable, confounder-adjusted estimates and their precision (eg, 95% confidence interval). Make clear which confounders were adjusted for and why they were included	7
		(*b*) Report category boundaries when continuous variables were categorized	
		(*c*) If relevant, consider translating estimates of relative risk into absolute risk for a meaningful time period	
Other analyses	17	Report other analyses done—eg analyses of subgroups and interactions, and sensitivity analyses	
**Discussion**			
Key results	18	Summarise key results with reference to study objectives	9
**Limitations**			
Interpretation	20	Give a cautious overall interpretation of results considering objectives, limitations, multiplicity of analyses, results from similar studies, and other relevant evidence	10
Generalisability	21	Discuss the generalisability (external validity) of the study results	10-11
**Other information**			
Funding	22	Give the source of funding and the role of the funders for the present study and, if applicable, for the original study on which the present article is based	12

*Give information separately for cases and controls in case-control studies and, if applicable, for exposed and unexposed groups in cohort and cross-sectional studies.

Note: An Explanation and Elaboration article discusses each checklist item and gives methodological background and published examples of transparent reporting. The STROBE checklist is best used in conjunction with this article (freely available on the Web sites of PLoS Medicine at http://www.plosmedicine.org/, Annals of Internal Medicine at http://www.annals.org/, and Epidemiology at http://www.epidem.com/). Information on the STROBE Initiative is available at www.strobe-statement.org.

For peer review only

BMJ Open

Long-term exposure to particulate air pollution and black carbon in relation to natural and cause specific mortality: a multi-cohort study in Sweden

Journal:	*BMJ Open*
Manuscript ID	bmjopen-2020-046040.R2
Article Type:	Original research
Date Submitted by the Author:	12-Mar-2021

Complete List of Authors:	Sommar, Johan; Umea University Department of Public Health and Clinical Medicine, Sustainable health<br>Andersson , Eva M.; Sahlgrenska University Hospital and University of Gothenburg, Occupational and Environmental Medicine<br>Andersson, Niklas; Institute of Environmental Medicine, Karolinska Institutet<br>Sallsten, Gerd; Sahlgrenska University Hospital and University of Gothenburg, Occupational and Environmental Medicine<br>Stockfelt, Leonard; Sahlgrenska University Hospital and University of Gothenburg, Occupational and Environmental Medicine<br>Ljungman, Petter; Karolinska Institutet<br>Segersson, David; SMHI<br>Eneroth, Kristina; SLB-analys, Environment and Health Administration<br>Gidhagen, Lars; SMHI<br>Molnar, Peter; Sahlgrenska University Hospital and University of Gothenburg, Occupational and Environmental Medicine<br>Wennberg, Patrik; Umeå University, Department of Public Health and Clinical Medicine, Family Medicine<br>Rosengren, Annika ; University of Gothenburg Sahlgrenska Academy, Institute of Medicine<br>Rizzuto, Debora; Karolinska Institutet , Aging Research Center, Department of Neurobiology, Health Care Sciences and Society; Stockholm University,<br>Leander, Karin; Karolinska Institutet, Institute of Environmental Medicine<br>Lager, Anton; Karolinska Institutet, Department of Public Health Sciences<br>Magnusson, Patrik; Karolinska Institutet, Dept. Medical Epidemiology and Biostatistics<br>Johansson, Christer; Department of Environmental Science and Analytical Chemistry<br>Barregard, Lars; Sahlgrenska University Hospital and University of Gothenburg, Occupational and Environmental Medicine<br>Bellander, Tom; Karolinska Institutet, Institute of Environmental Medicine; Stockholm County Council, Centre for Occupational and Environmental Medicine<br>Pershagen, Göran; Institute of Environmental Medicine, Karolinska Institutet<br>Forsberg, Bertil; Umeå University, Public Health and Clinical Medicine

**Primary Subject Heading**:	Epidemiology
Secondary Subject Heading:	Public health
Keywords:	EPIDEMIOLOGY, PUBLIC HEALTH, Cardiac Epidemiology < CARDIOLOGY

SCHOLARONE™
Manuscripts

I, the Submitting Author has the right to grant and does grant on behalf of all authors of the Work (as defined in the below author licence), an exclusive licence and/or a non-exclusive licence for contributions from authors who are: i) UK Crown employees; ii) where BMJ has agreed a CC-BY licence shall apply, and/or iii) in accordance with the terms applicable for US Federal Government officers or employees acting as part of their official duties; on a worldwide, perpetual, irrevocable, royalty-free basis to BMJ Publishing Group Ltd ("BMJ") its licensees and where the relevant Journal is co-owned by BMJ to the co-owners of the Journal, to publish the Work in this journal and any other BMJ products and to exploit all rights, as set out in our licence.

The Submitting Author accepts and understands that any supply made under these terms is made by BMJ to the Submitting Author unless you are acting as an employee on behalf of your employer or a postgraduate student of an affiliated institution which is paying any applicable article publishing charge ("APC") for Open Access articles. Where the Submitting Author wishes to make the Work available on an Open Access basis (and intends to pay the relevant APC), the terms of reuse of such Open Access shall be governed by a Creative Commons licence – details of these licences and which Creative Commons licence will apply to this Work are set out in our licence referred to above.

Other than as permitted in any relevant BMJ Author's Self Archiving Policies, I confirm this Work has not been accepted for publication elsewhere, is not being considered for publication elsewhere and does not duplicate material already published. I confirm all authors consent to publication of this Work and authorise the granting of this licence.

For peer review only

Long-term exposure to particulate air pollution and black carbon in relation to natural and cause specific mortality: a multi-cohort study in Sweden

Johan Nilsson Sommar[1*], Eva M. Andersson[2], Niklas Andersson[3], Gerd Sallsten[2], Leo Stockfelt[2], Petter L. S. Ljungman[3,4], David Segersson[5], Kristina Eneroth[6], Lars Gidhagen[5], Peter Molnar[2], Patrik Wennberg[7], Annika Rosengren[8], Debora Rizzuto[9,10], Karin Leander[11], Anton Lager[12,13], Patrik KE Magnusson[14], Christer Johansson[6,15], Lars Barregard[2], Tom Bellander[3,16], Göran Pershagen[3,16] and Bertil Forsberg[1]

[1] Section of Sustainable health, Department of Public Health and Clinical Medicine, Umeå University, Umeå, Sweden
[2] Occupational and Environmental Medicine, Department of Public Health and Community Medicine, Institute of medicine, Sahlgrenska Academy, University of Gothenburg & Sahlgrenska University Hospital, Sweden.
[3] Institute of Environmental Medicine, Karolinska Institutet, Stockholm, Sweden
[4] Department of Cardiology, Danderyd Hospital, Stockholm, Sweden
[5] Swedish Meteorological and Hydrological Institute, Norrköping, Sweden
[6] SLB-analys, Environment and Health Administration, Stockholm, Sweden
[7] Public Health and Clinical Medicine, Family Medicine, Umeå University, Umeå, Sweden
[8] Department of Molecular and Clinical Medicine, Institute of Medicine, University of Gothenburg, Sahlgrenska University Hospital, Gothenburg, Sweden
[9] Ageing Research Center, Department of Neurobiology, Care Sciences and Society, Karolinska Institutet and Stockholm University, Stockholm, Sweden
[10] Stockholm Gerontology Research Center, Stockholm, Sweden
[11] Institute of Environmental Medicine, Karolinska Institutet, Stockholm, Sweden
[12] Centre for Epidemiology and Community Medicine, Stockholm County Council, Stockholm, Sweden
[13] Department of Public Health Science, Karolinska Institutet, Stockholm, Sweden
[14] Department of Medical Epidemiology and Biostatistics, Karolinska Institutet, Stockholm, Sweden
[15] Department of Environmental Science, Stockholm University, Stockholm, Sweden
[16] Centre for Occupational and Environmental Medicine, Region Stockholm, Stockholm, Sweden

* Corresponding author:

Department of Public Health and Clinical Medicine, Section of Sustainable Health, Umeå University, Umeå, Sweden
Phone: +46 907853453
E-mail: johan.sommar@umu.se

For peer review only - http://bmjopen.bmj.com/site/about/guidelines.xhtml

Abstract

Objectives: To estimate concentration-response relationships for particulate matter (PM) and black carbon (BC) in relation to mortality in cohorts from three Swedish cities with comparatively low pollutant levels.

Setting: Cohorts from Gothenburg, Stockholm and Umeå, Sweden.

Design: High-resolution dispersion models were used to estimate annual mean concentrations of PM with aerodynamic diameter ≤10 µm (PM10) and ≤2.5 µm (PM2.5), and BC, at individual addresses during each year of follow up 1990-2011. Moving averages were calculated for the time windows 1-5 years (lag 1-5) and 6-10 years (lag 6-10) preceding the outcome. Cause-specific mortality data were obtained from the national cause of death registry. Cohort-specific hazard ratios were estimated using Cox regression models and then meta-analysed including a random effect of cohort.

Participants: During the study period, 7340 cases of natural mortality, 2755 cases of cardiovascular disease (CVD) mortality and 817 cases of respiratory and lung cancer mortality were observed among in total 68679 individuals and 689813 person-years of follow-up.

Results: Both PM10 (range: 6.3–41.9 µg/m^3) and BC (range: 0.2-6.8 µg/m^3) were associated with natural mortality showing 17% (95% CI 6-31%) and 9% (95% CI 0-18%) increased risks per 10 µg/m^3 and 1 µg/m^3 of lag1-5 exposure, respectively. For PM2.5 (range: 4.0–22.4 µg/m^3) the estimated increase was 13% per 5 µg/m^3, but less precise (95% CI -9-40%). Estimates for CVD mortality appeared higher for both PM10 and PM2.5. No association was observed with respiratory mortality.

Conclusion: The results support an effect of long-term air pollution on natural mortality and mortality in CVD with high relative risks also at low exposure levels. These findings are relevant for future decisions concerning air quality policies.

Key words: air pollution, particulate matter, black carbon, natural mortality, cardiovascular mortality

For peer review only

Strengths and limitations of this study

Few previous studies have followed up individuals by home address and time varying exposures as annual mean concentrations at actual place of residence

High-resolution dispersion models of particle concentrations were used to capture local contrasts in exposure

The study did however not include information on noise exposure and green space near the home address

This study showed associations with mortality within populations with comparatively low particle concentrations, generally below EU standards

As the first (multi)-cohort study using high resolution dispersion modelled particle concentrations at mean levels below WHO guidelines, this study found high relative risks of mortality

For peer review only

For peer review only - http://bmjopen.bmj.com/site/about/guidelines.xhtml

Introduction

Particulate matter ambient air pollution exposure has been associated with increased risk of premature mortality in several cohort studies [1 2]. According to the Global Burden of Diseases Study 2017 [3], assessing 84 risk factors for premature mortality in 195 countries, ambient particulate matter air pollution was ranked as the tenth most important risk factor for mortality and the most important environmental risk factor. Using risk functions restricted to cohort studies of outdoor air pollution, ambient particle matter pollution was estimated to cause 2.9 million (95% confidence interval (CI): 2.5-3.4) deaths in year 2017.

In a review of 11 cohort studies on long-term air pollution exposure and mortality, ambient concentrations of PM2.5 were associated with increased risk of all-cause mortality and particularly cardiovascular mortality. However, the heterogeneity between study relative risk estimates was large [2]. The large European Study of Cohorts for Air Pollution Effects (ESCAPE) project including 22 cohorts, observed an increased risk of overall mortality associated with ambient PM2.5 concentrations [4] whereas no association was found with cardiovascular mortality [5]. In a recent study within a large Danish cohort found increased risks for both natural cause and CVD mortality associated with PM2.5 exposure during the last 15 years [6]. Neither the Danish study nor the ESCAPE study were able to demonstrate associations with respiratory mortality [6 7].

Black carbon (BC), one component of PM, has been hypothesized to play a key role in the adverse health effects of air pollution exposure [8]. The review by Luben et al. identified three studies on the long-term effect of BC (or elemental carbon) and mortality in coronary heart disease [9] and ischemic heart disease [10 11]. However, the review did not find support for a stronger association with mortality for BC compared with PM2.5. Elemental carbon has been associated with all-cause mortality in another meta-analysis [2]. Within the ESCAPE project, PM2.5-absorbance was used as a proxy for BC. The estimated increased risk of natural mortality associated with PM2.5-absorbance did not reach statistical significance [4]. In Denmark (areas of Copenhagen and Aarhus), BC was, however, associated with both all-cause and cardiovascular mortality [6].

These previous studies varied in the method used for the exposure assessment of particle concentrations. Earlier studies assessed concentrations using centrally located monitoring stations, mostly representing the urban background [12]. Later studies use air pollution monitoring together with data on land use (land use regression, LUR) or detailed data on emissions and meteorological conditions (dispersion models). Most studies on long-term air pollution effects have assessed outdoor residential exposures at the year of inclusion into the cohort, a recent study on mortality however used annual mean concentrations on addresses during follow-up [6]. Studies using exposure assessments with a higher precision and in low-level exposure environments demonstrated higher relative risks of mortality in a recent meta-analysis [13]. Studies on low level air pollution are few, however. Adding to the knowledge from the long-term air pollution effects on mortality from the Canadian Community Health Survey-Mortality cohort [14-16] and a Medicare population in New England [17], this study is of importance for policy making and determination of limit values. Compared to these previous studies that used a 1x1 km^2 resolution for the assessment of air pollutant concentrations this study will make use of high-resolution 50x50 m^2 dispersion modelled particulate matter able to capture also local exposure contrasts.

Aim

This study aims to use high-resolution dispersion modelled annual mean residential PM10, PM2.5 and BC concentrations to estimate associations with natural and cause specific mortality in a

For peer review only - http://bmjopen.bmj.com/site/about/guidelines.xhtml

northern European environment characterized by annual averages largely compliant with EU air quality standards.

Methods and Materials

Study Cohorts

The study included four cohorts; two cohorts from Gothenburg, one from Stockholm (pooled from four cohorts) and one cohort from Umeå. Our study period ranged from January 1st 1990 to December 31st 2011 (December 31st 2013 for Umeå participants residing at the same address as in 2011). Residential address history was obtained for all cohort participants through linkage using personal identification numbers to mandatory records of residential addresses at Statistics Sweden or the Swedish Taxation Authority. These residential addresses were then geocoded by matching against the Swedish Mapping Cadastral and Land Registration Authority databases. When needed, addresses were manually checked and corrected for inconsistencies and assigned geographical coordinates.

From Gothenburg, two general population cohorts were included: The Primary Prevention Study (PPS) cohort and the GOT-MONICA cohort. The PPS cohort recruited a random third of all men in Gothenburg born between the years 1915 and 1925 who were examined in 1970-73 to study predictors of cardiovascular disease [18]. The GOT-MONICA cohort is one of the cohorts within the international MONICA project ("Multinational Monitoring of Trends and Determinants in Cardiovascular Diseases"), designed to study risk factors for cardiovascular diseases [19 20]. GOT-MONICA included residents aged 25-64 years. Recruitments were conducted in 1985, 1990 and 1995. For both cohorts, background data and information on cardiovascular risk factors were recorded by questionnaires. The recruited individuals were also examined by health care professionals.

Recruiting individuals from Stockholm County the Cardiovascular Effects of Air pollution and Noise Study (CEANS) cohort was included [21]. The cohort consists of four sub-cohorts. The Stockholm Diabetes Prevention Program [SDPP] was designed as a population-based prospective cohort, and includes 3128 men recruited between the years 1992 and 1994 and 4821 women recruited between the years 1996 and 1998. The program recruited individuals aged 35-56 years that had not previously been diagnosed with diabetes. About half of the recruited had a first or second degree relative with a history of diabetes. These were then matched with recruits of the same age and sex, who did not have a relative with a diabetes history. The second sub-cohort named SIXTY consists of a population-based random sample of 60-year old men and women (N=4232) living within Stockholm County between August 1997 and March 1999. The third cohort consists of the Screening Across the Lifespan Twin study [SALT], including twins born before year 1958. Only participants residing in Stockholm County at recruitment were included in the current analysis (N=7043). The fourth sub-cohort, the Swedish National Study on Aging and Care in Kungsholmen [SNAC-K], included a random sample of inhabitants aged at least 60 years and residing in a central area of Stockholm City (N=3363). Sub-cohort specific characteristics of included individuals in CEANS have previously been presented [22].

Including Umeå municipality, the Västerbotten intervention program (VIP) is a program where the population in the County is invited to a health examination the year they turn 40, 50 and 60 (and during some years also 30) years old [23]. The program was initiated in parts of the county in 1985 as a community and individual level program to reduce the morbidity and mortality from cardiovascular disease in Northern Sweden. The interview questions provide information about cardiovascular risk factors as well as social situation, education, diet and physical activity. So far more than 100 000 individuals have participated in the program.

For peer review only - http://bmjopen.bmj.com/site/about/guidelines.xhtml

The study was reviewed and approved by the Ethical Review Boards of Gothenburg (references T800-08 and T547- 13), Stockholm (reference 2018/2064-32), and Umeå (reference 2015/16-31Ö).

The public was not involved in the design, or conduct, or reporting, or dissemination plans of the research.

Patient and public involvement

No patient involved.

Mortality Outcomes

Cause-specific mortality was determined by linkage of national personal identification numbers to the Cause of Death Register at the Swedish National Board of Health and Welfare. We used the International Code of Diseases (ICD)9 001-779 or ICD-10 A00-R99 to define deaths by natural causes, ICD-9 400-440 or ICD-10 I10-I70 to define deaths in CVD, ICD9 162 or ICD10 C34 to define lung cancer deaths, and ICD9 460-519 or ICD10 J0-99 to define non-malignant respiratory mortality. In addition, deaths by other causes was studied (that is other than CVD, lung cancer, respiratory disease, and external causes).

Exposure Assessment

An in depth description of the dispersion model used for the exposure assessment has previously been published [24]. Both regional and local emission inventories for the years 1990, 2000 and 2011 in Gothenburg and Umeå, and years 1990, 1995, 2000, 2005 and 2011 in Stockholm, were used as input to Gaussian dispersion model simulations of annual mean concentrations of particulate matter (PM), i.e. PM10, PM2.5 and BC. To allow high resolution in vicinity of roads, a quadtree receptor grid was used, resulting in a resolution down to 35 m x 35 m. For inner-city streets with buildings on one or both sides, an additional concentration component was simulated with the Danish operational street pollution model (OSPM) [25].

Monitoring data were used in Stockholm to adjust the modelled concentrations to meteorological year-to-year variability. In Gothenburg and Umeå interpolation was used between the years and each year was also adjusted by a ventilation factor taking meteorological variability into account. Emission factors for traffic PM-exhaust for different vehicle types, speeds and driving conditions were calculated based on the Handbook on Emission Factors for Road Traffic version 3.1 [26], whereas emission factors for black carbon were based on TRANSPHORM [27]. The non-exhaust emissions of PM consist mainly of road wear particles, with a minor contribution from brake and tire wear [28-30]. For small-scale residential heating, emissions in Gothenburg and Stockholm were determined using household energy consumption data from Statistics Sweden with a resolution of 100 m x 100 m. This was based on proxy data including number of stoves or boilers in each municipality, living space of small houses per square km, population density per 100 m^2 and availability of district heating. In Umeå a detailed inventory of individual stoves and boilers was used with data from chimney sweepers and interviews about amount of wood burning [24]. Industrial and energy production facilities were included as point sources in the model. Emission from shipping were also included using a method described by Jalkanen et al. [31].

To model the long-range transport of PM10, PM2.5 and BC the difference between total concentrations measured at monitoring stations and modelled local particle concentrations at the same location was considered on an annual basis (Gothenburg and Umeå). In Stockholm annual concentrations from a regional background station was used to estimate the long-range transport. The long-range transport contributions were estimated differently for each city, but within each city

For peer review only - http://bmjopen.bmj.com/site/about/guidelines.xhtml

they had a purely temporal contrast. This was assumed since the spatial variation between regional background sites has been shown to be small [32]. These yearly contributions of background concentrations were then added to the estimated local concentrations. In a previous study, validations of total concentrations against measurements showed R2 values of 0.87, 0.65 and 0.93 for PM10, PM2.5, and BC, respectively [24].

The resulting concentrations were lastly added to each study participant's home addresses and annual exposures estimated accounting for any changes in address during the study period 1990-2011.

Confounders

Cohort-specific models were adjusted for potential confounding by including sex, calendar year, sub-cohort (in Stockholm), smoking status (current, former, never smoker), alcohol consumption (in Stockholm and Umeå; daily, weekly, seldom, never), physical activity (in Gothenburg and Umeå: sedentary, moderate, intermediate or vigorous; in Stockholm: once a month or less/>1 hour per week, about once a month/1 hour per week, 3 times a week or more/>2 hour per week), marital status (single, married or living with partner, no answer), socio-economic index by occupation (in Gothenburg (PPS) and Stockholm: blue collar, low and intermediate white collar and self-employed, high level white collar and self-employed professionals with academic degrees, no answer), education level (in Gothenburg (MONICA), Stockholm and Umeå: primary school or less, up to secondary school or equivalent, university degree or more, no answer), occupation status (in Stockholm and Umeå: gainfully employed, unemployed/not gainfully employed, retired, no answer). Area level socioeconomic status was estimated for each cohort member's residence from the mean neighborhood individual income in persons of working age by Small Areas for Market Statistics (SAMS) provided by Statistics Sweden for the calendar year 1994.

Statistical methods

Cox-proportional hazard models were used to estimate hazard ratios of mortality associated with PM and BC in cohort-specific analyses. Age was used as the underlying time variable for the baseline hazard. The regression model included adjustment for calendar year, baseline information on confounders as well as area level socio-economy. Since the Stockholm CEANS cohort consisted of four sub-cohorts that differed in age and time of recruitment, separate baseline hazards were used for the analyses within this cohort. We censured individuals at death by other causes, the end of the study period or time of permanent emigration from the study areas. Associations with PM and BC were assessed using two exposure windows; a moving average over 1-5 years preceding the event (lag1-5) and 6-10 years preceding the event (lag6-10). For inclusion, annual mean concentrations were required for at least 80% of the time window. These moving averages were used as time-varying exposures and therefore considers both between and within individual contrast during the study period. Besides these time windows, associations were also assessed with concentrations at the year of recruitment. Hazard ratios and 95% confidence intervals (CI) were expressed per 10, 5 and 1 $\mu g/m^3$ for PM10, PM2.5 and BC, respectively, as well as per interquartile ranges (IQRs) across the three study areas.

Meta-analyses of cohort-specific estimates were performed using random effects [33]. Heterogeneity between cohort estimates were assessed by the I2 statistic and statistically tested for deviations from homogeneity by a Chi-square test based on the Cochran's Q statistic.

Results

For peer review only - http://bmjopen.bmj.com/site/about/guidelines.xhtml

Participant characteristics

The total number of individuals included in our meta-analysis was 68,679, with the number of individuals included in each cohort presented in table 1. Total number of deaths by natural causes was 7344. Age at recruitment differed largely between cohorts with the highest median in the Gothenburg PPS cohort and lowest in VIP Umeå, 69 compared with 40 years. By design only males were recruited to the Gothenburg PPS cohort, whereas the other three cohorts had a slightly larger proportion of women compared with men. Baseline current smoking was highest in the Gothenburg PPS cohort and lowest in Stockholm CEANS and VIP Umeå, 39% compared with about 20%. Leisure time physical activity had a different categorization within the Stockholm CEANS cohort but it can be concluded that there was a larger proportion of individuals reporting a sedentary lifestyle at baseline in this cohort. The cohorts in Gothenburg had the largest proportion of physically active individuals, with higher frequency and intensity. Alcohol consumption was more frequent in Stockholm CEANS compared with VIP Umeå. University education was most common in Stockholm CEANS and VIP Umeå (above 30%) and less common in Gothenburg. A larger proportion of individuals were gainfully employed in the younger VIP Umeå than in Stockholm CEANS, 84% compared with 66%. Blue-collar occupation was more likely within the Gothenburg PPS cohort compared with Stockholm CEANS.

Particle concentrations

Particle concentrations of PM10, PM2.5 and BC all had the highest mean and IQR in the Gothenburg cohorts and the lowest in VIP Umeå (figure 1-3). In total, means of lag1-5 PM10, PM2.5 and BC were; 11.3, 6.9 and 0.6 μg/m^3, respectively. Lag1-5 concentrations were on average lower than lag6-10 concentrations due to a decreasing trend in concentrations during the study period. The Pearson correlation coefficient between lag 1-5 concentrations of PM10 and PM2.5 was 0.88, PM10 and BC 0.71, and between PM2.5 and BC 0.75.

Associations with mortality

Meta-estimates showed increased risks associated with PM10, PM2.5 and BC in relation to natural mortality; for lag1-5 17% (95% CI 6-31) increased risk per 10 μg/m^3 PM10, 13% (95% CI -9-40) increased risk per 5 μg/m^3 PM2.5 and 9% (95% CI 0-18) increased risk per 1 μg/m^3 BC (figure 4-6; crude estimates in supplementary material figure S1a-c). The statistical uncertainty was however considerable for the combined risk increase associated with PM2.5 and the confidence interval included 1. For PM10 and PM2.5 higher risk estimates were found for lag1-5 concentrations compared with lag6-10, whereas for BC, the risk estimates were the same. Increased risks were estimated for all cohorts except for Gothenburg MONICA, where the hazard ratio estimate for natural mortality was below 1.

The risk estimates for CVD mortality associated with PM10 and PM2.5 appeared higher compared with natural mortality; 19 (95% CI 1-40) and 23% (95% CI 3-48) increased risks for lag1-5, per 10 and 5 μg/m^3, respectively (figure 7-9; crude estimates in supplementary material figure S2a-b). The increased risks associated with BC seemed lower for CVD mortality than for natural mortality for lag1-5, but similar for lag6-10 (figure 9; crude estimates in supplementary material figure S2c).

The HRs per IQR for natural mortality were higher for PM2.5 and PM10 than for BC; 6, 5 and 2% increased risk, respectively. The same order per IQR was found for CVD mortality; 10, 5 and 1% for PM2.5, PM10 and BC, respectively.

No associations between PM and lung cancer or non-malignant respiratory mortality were observed (supplementary material figure S3-4a-c). Increased risk was, however, observed for mortality by

For peer review only - http://bmjopen.bmj.com/site/about/guidelines.xhtml

other causes (that is other than CVD, lung cancer, respiratory disease, and external causes) with the same magnitude as observed for natural and CVD mortality (supplementary material figure S5a-c).

For comparison with previous studies, associations were also assessed with residential PM concentrations for the recruitment year. These estimates were in general lower compared with those with lag1-5 and lag6-10 concentrations (supplementary material figure S6a-b). Estimated risk increases for natural mortality were 7 (95% CI -4-18), 8 (95% CI -2-20) and 3% (95% CI -4-11) in association with PM10, PM2.5 and BC, per 10, 5 and 1 µg/m^3, respectively.

The heterogeneity between cohort hazard ratio estimates in relation to precision was in general very low. Heterogeneity was, however, observed for associations with lag1-5 PM2.5 and lag6-10 BC in relation to natural mortality, although non-of these were statistically significant (figure 4-9).

Discussion

We observed in four Swedish cohorts located in Gothenburg, Stockholm and Umeå increased risks associated with long-term residential outdoor levels of PM10, PM2.5 and BC in relation to natural and CVD mortality. PM10 was most consistently associated with natural and CVD mortality, whereas larger uncertainties were found for the associations between PM2.5 and CVD mortality and BC and natural mortality. Risk estimates associated with PM10 and PM2.5 appeared larger for exposures during the last 5 years in comparison to 6-10 years prior. For BC the associated risks were the same for both exposure windows in relation to natural mortality but higher for the exposure window 6-10 years prior in relation to CVD mortality. Within a previous meta-analysis of these cohorts, an association was found between BC and stroke incidence [22]. No association was, however, found between PM10 or PM2.5 and incidence in ischemic heart disease.

The observed 17% increased risk per 10 µg/m^3 PM10 in relation to natural mortality is high compared with most studies (e.g. the 6% increased risk found by Hoek et al. 2013 [2]) but similar to the recent findings within a Danish cohort presenting 12% increased risk (95% CI 3-22%) associated with a 15-year moving average of residential ambient concentrations [6]. Their estimate for CVD mortality was 30% (95% CI 11-53%) which was higher than our meta-estimate of 19%. For PM2.5 our observed risk increase by 13% per 5 µg/m^3 for natural mortality is also in agreement with the Danish estimate of 13% (95 CI 5-21%). As for PM10 the risk increase for CVD mortality associated with PM2.5 was again lower in our study, 23% compared to 29% (95% CI 13-47%). The average PM10 and PM2.5 exposure levels were 2.2 and 2.6 times higher in the Danish cohort compared to our study (lag 1-10 time-weighted concentrations from the Danish cohort compared with lag 1-5 moving averages in our study).

Using less detailed assessments of outdoor residential exposures, a meta-analysis of 22 cohorts within the ESCAPE project reported a borderline significant 4% (95% CI 0-9%) increased risk of natural-cause mortality for a 10 µg/m^3 difference in annual PM10 at the year of recruitment [4]. No association with PM10 was, however, observed for deaths in ischemic heart disease or myocardial infarction, whereas a suggested association was found in relation to deaths in cerebrovascular disease; 22% (95% CI -9-63%) increased risk per 10 µg/m^3 [5]. The findings for mortality associated with PM2.5 have been heterogeneous, however, the meta-estimate among the ESCAPE cohorts showed a 7% (95% CI 2-13%) increased risk per 5 µg/m^3 of natural mortality and 21% (95% CI -13-69%) increased risk of death in cerebrovascular disease. As for PM10 no association was found in relation to death in ischemic heart disease or myocardial infarction. Within earlier studies; a review and meta-analysis by Hoek et al. (2013) found 6% (95% CI 4-8%) and 15% (95% CI 4-27) increased risks of all-cause mortality and cardiovascular mortality per 5 µg/m^3, respectively. A stronger association between PM and mortality with cardiovascular causes compared to all natural mortality has also

For peer review only - http://bmjopen.bmj.com/site/about/guidelines.xhtml

been reported by others [1], and is in agreement with our findings on CVD mortality; and most apparent in relation to exposures during the last 5 years.

In accordance with our meta-estimate for BC the Danish cohort reported a risk increase by 9% (95% CI 4-15%) per 1 µg/m3 for all-cause mortality [6]. An increased risk by 16% (95% CI 5-27%) was also observed for CVD mortality, higher than the 5% increased risk found in our study. In the review by Hoek et al. (2013) elemental carbon was found associated with a 6% (95% CI 5-7%) increased risk of all-cause mortality per 1 µg/m^3.

The lack of association between PM and non-malignant respiratory mortality in our study is in agreement with results from both the Danish study [6], the ESCAPE study [7] and a large cohort study in Rome [34]. Previous studies have, however, found an increased risk of lung cancer incidence associated with both PM10 and PM2.5 [35 36]. The statistical power for these outcomes was low in our meta-analyses due to the limited number of deaths in lung cancer and other respiratory causes.

The observed heterogeneity of PM associated risk for premature mortality between studies is possibly related to differences in particle composition, housing with differences in particle infiltration, population characteristics, and the minor differences in confounding adjustments. The air pollution dispersion model used for the exposure assessment in our study has a high spatial resolution, and for the follow up period it was also updated with new regional and local emission inventories as well as PM measurements to provide annual mean temporal PM concentrations. A higher precision in the exposure estimate have been found to produce higher relative health risk estimates [13]. The mortality risk associated with PM2.5 has recently been reported to be higher for local sources compared with distant sources, but with less precision in relative risk estimates, indicating the importance of high spatial resolution exposure data to successfully model differences in mortality [37]. The meta-analysis results in this study and the results presented by Hvidtfeldt et al. (2019) both generally provided higher hazard ratio estimates compared with previous mortality studies. Both studies also used moving averages based on annual mean concentrations at the place of residence. Besides exposure data with a high precision such a moving average over relevant time windows may be advantageous compared with residential concentrations at the year of recruitment or a time-weighted average exposure during the follow-up. These two studies also adjusted both for individual data on confounders and neighbourhood level data on socioeconomics.

Strengths and limitations

The strength of this study includes the state-of-the-art air pollution modelling used for exposure assessment. Several factors that affect the local dispersion of air pollution particles were considered including meteorological conditions, amount of traffic including vehicle types and speed, which affect exhaust and wear emissions, the width of the street, and the height of neighbouring building. This facilitates models with a high spatial resolution providing the ability to estimate an individual's exposure at the residential address. Temporal differences for each address were also accounted for by repeatedly updating the exposure model during the period of follow up. Our modelled concentrations have showed a good agreement when validated against measured concentrations by monitoring stations in city centres [24]. Due to uncertainties in data input to the dispersion model, and since modelled PM levels at the home address do not equal true personal exposure, exposure misclassifications at the individual level will nevertheless arise. True personal exposure would for instance include indoor particle concentrations at the place of residence, at the workplace, and during commuting. During the years of follow up there has been a decreasing trend in both total concentrations of PM and age specific mortality. It was therefore important to adjust for calendar year in the Cox models. Since this is an observational study it was also necessary to adjust for a set of

For peer review only - http://bmjopen.bmj.com/site/about/guidelines.xhtml

potential confounders, which were prespecified. Mortality outcomes, measures of exposures (PM10, PM2.5 and BC) and time windows (time dependent moving averages over lags 1-5 and 6-10, respectively) were also a priori selected. Besides natural mortality associations were assessed with CVD and three (more) specific causes of death. A limitation that needs to be considered for more exploratory outcomes is that confidence intervals were not constructed to maintain a 95% familywise error rate. Limitations also include the lack of information on noise exposure and green space near the home address which could be confounders or effect-modifiers of air pollution effects.

Another strength of this study is the large number of cohort participants and ability to adjust for a large set of confounders at both individual and area level, however information on confounders from questionnaires were only available at baseline. Even though we expect the cohort participants with age ranging from 40 years to have fairly stable lifestyles and habits, such baseline variables may change, for example due to disease. No clear heterogeneity in associations between cohorts was observed, however, with only four cohorts the test had low statistical power to reject the null hypothesis of homogeneity.

This study additionally contributes with hazard ratio estimates between PM and mortality in the lower range of exposure with PM2.5 ranging between 4.0 to 20.4 μg/m^3 and PM10 ranging between 6.3 to 41.9 μg/m^3. Even though this is lower than the current European Union standards of 25 μg/m^3 for PM2.5 and (with a few exceptions) lower than 40 μg/m^3 for PM10, the study found higher relative risks for premature mortality compared with most previous studies even though mean exposures in those studies have been higher.

Conclusions

This study of four cohorts with relatively low exposures showed increased risks of mortality in association with long-term exposure to PM10, PM2.5 and BC, and thus strongly support long-term air pollution associated effects. Since high risk increases were observed even at relatively low exposures, the findings are relevant for future decisions concerning air quality policies.

For peer review only - http://bmjopen.bmj.com/site/about/guidelines.xhtml

Tables

Table 1. Descriptive statistics of the study participants in each cohort

		Gothenburg, MONICA	Gothenburg, PPS	Stockholm, CEANS	Umeå, VIP
Participants (n)		4,500	5,850	22,314	42,580
Baseline data collection (calendar years)		1985,1990,1995	1970-74	1992-2004	1992-2014
Age at enrolment (years; median (range))		46 (25–66)	51 (47–56)	56 (35-104)	40 (40-50)
Women (%)		52	0	58	52
Smoking status (%)	Current smoker	29	39	22	19
	Former smoker	N/A	33	36	30
	Never smoker	65	27	40	49
	Missing data	5	0	1.8	1
Physical activity (%)	Once a month or less/<1h/week	N/A	N/A	61	N/A
	About once a month/~1h/week	N/A	N/A	26	N/A
	3 times a week or more/>2h/week	N/A	N/A	7.6	N/A
	Missing data	N/A	N/A	4.6	N/A
Leisure time physical activity (%)	Sedentary	18	24	N/A	36
	Moderate	62	59	N/A	42
	Intermediate and vigorous	18	17	N/A	22
	Missing data	2	1	N/A	2
Alcohol consumption (%)	Daily	N/A	N/A	6.7	2
	Weekly	N/A	N/A	55	16
	Seldom	N/A	N/A	31	42
	Never	N/A	N/A	5.6	1
	Missing data	N/A	N/A	1.8	39
Married/living with partner (%)	No	21	14	29	23
	Yes	47	86	70	77
	Missing data	32	<0.1	1.3	1
Education level (%)	Primary school or less	13	N/A	30	30
	Up to secondary school or equivalent	32[a]	N/A	36	30
	University degree & more	20	N/A	31	40
	Missing data	35	N/A	2.8	1

For peer review only

Occupation (%)	Gainfully employed	N/A	N/A	66	85
	Unemployed/not gainfully empolyed	N/A	N/A	5.8	6
	Retired	N/A	N/A	27	4
	Missing data	N/A	N/A	1.3	4
Socioeconomic index by occupation (%)	Blue collar	N/A	48	27	N/A
	Low-and intermediate white collar and self-employed	N/A	23	51	N/A
	High level white collar and self-employed professional with academic degrees	N/A	30	18	N/A
	Missing data	N/A	0	4.4	N/A
Mean income by SAMS 1994 (SEK)		154,780	148,602	N/A	130,000
Mean income by SAMS 2009 (SEK)		N/A	N/A	303,910	N/A

CEANS, Cardiovascular Effects of Air Pollution and Noise Study; MONICA, Multinational Monitoring of Trends and Determinants in Cardiovascular Diseases; N/A, not available; PPS, Primary Prevention Study; SAMS, Small Areas for Market Statistics; SEK, Swedish Krona; VIP, Västerbotten Intervention Programme.

a. Includes technical training

For peer review only

For peer review only - http://bmjopen.bmj.com/site/about/guidelines.xhtml

Legends to figures

Figure 1. Boxplot and density function of PM10 concentrations, in total and separate for each cohort. Tabulated values are quartile limits together with means and standard deviations.

Figure 2. Boxplot and density function of PM2.5 concentrations, in total and separate for each cohort. Tabulated values are quartile limits together with means and standard deviations.

Figure 3. Boxplot and density function of BC concentrations, in total and separate for each cohort. Tabulated values are quartile limits together with means and standard deviations.

Figure 4. Adjusted hazard ratios of natural mortality associated with PM10 within each cohort and by random effect meta-analyses estimates. Exposures were assessed by moving average residential concentrations within the exposure windows last 5 years and 6 to 10 years prior.

Figure 5. Adjusted hazard ratios of natural mortality associated with PM2.5 within each cohort and by random effect meta-analyses estimates. Exposures were assessed by moving average residential concentrations within the exposure windows last 5 years and 6 to 10 years prior.

Figure 6. Adjusted hazard ratios of natural mortality associated with BC within each cohort and by random effect meta-analyses estimates. Exposures were assessed by moving average residential concentrations within the exposure windows last 5 years and 6 to 10 years prior.

Figure 7. Adjusted hazard ratios of CVD mortality associated with PM10 within each cohort and by random effect meta-analyses estimates. Exposures were assessed by moving average residential concentrations within the exposure windows last 5 years and 6 to 10 years prior.

Figure 8. Adjusted hazard ratios of CVD mortality associated with PM2.5 within each cohort and by random effect meta-analyses estimates. Exposures were assessed by moving average residential concentrations within the exposure windows last 5 years and 6 to 10 years prior.

Figure 9. Adjusted hazard ratios of CVD mortality associated with BC within each cohort and by random effect meta-analyses estimates. Exposures were assessed by moving average residential concentrations within the exposure windows last 5 years and 6 to 10 years prior.

For peer review only

Author contributions

JNS performed the meta-analysis and wrote with support from BF the first draft manuscript. JNS, EA, NA and GS conducted the cohort analyses. DS, KE, LG, CJ were responsible for dispersion modelling of particles. PW, AR, DR, KL, AL, PM and PKM and were responsible for collection of cohort data. GP, BF, LB, TB, JNS, GS, LS, PL, EA and NA designed the epidemiological study. All authors contributed to the interpretation of the results, finalization of the manuscript, and approved the final manuscript.

Competing interests

All authors have completed the ICMJE uniform disclosure form at www.icmje.org/coi_disclosure.pdf and declare: no support from any organization other than those listed above for the submitted work; no financial relationships with any organizations that might have an interest in the submitted work in the previous three years; no other relationships or activities that could appear to have influenced the submitted work.

Funding

This study was funded by the Swedish Environmental Protection Agency as part of the Swedish Clean Air and Climate Research Program (grant NV-06576-13). BF and JNS was also supported by NordForsk grant no. 75007. The GOT-MONICA and PPS cohorts were supported by the Swedish Research Council, 2013-5187 (SIMSAM). The SDPP cohort was funded by the Stockholm County Council, the Swedish Research Council, the Swedish Diabetes Association, the Novo Nordisk Scandinavia, and GlaxoSmithKline. The 60YO cohort was funded by the Stockholm County Council and the Swedish Research Council. The Swedish Twin Registry is managed by Karolinska Institutet and receives funding through the Swedish Research Council under the grant no. 2017-00641. Data collection in SALT was supported by a grant from the National Institutes of Health (NIH), grant no. 1R01 AG08724. The VIP-Umeå cohort was funded by the Västerbotten County Council. PLL was supported by funding from the Swedish Research Council for Health, Working Life and Welfare (FORTE) 2015-00917 and Karolinska Institute's Strategic Research Area in Epidemiology (SFO-EPI). LB was financed by grants from the Swedish state under the agreement between the Swedish government and the county councils: the ALF-agreement (74580).

Data sharing statement

No additional data available

For peer review only

References

1. Brook RD, Rajagopalan S, Pope CA, 3rd, et al. Particulate matter air pollution and cardiovascular disease: An update to the scientific statement from the American Heart Association. *Circulation* 2010;121(21):2331-78. doi: 10.1161/CIR.0b013e3181dbece1
2. Hoek G, Krishnan RM, Beelen R, et al. Long-term air pollution exposure and cardio- respiratory mortality: a review. *Environ Health-Glob* 2013;12 doi: Artn 43 10.1186/1476-069x-12-43
3. Stanaway JD, Afshin A, Gakidou E, et al. Global, regional, and national comparative risk assessment of 84 behavioural, environmental and occupational, and metabolic risks or clusters of risks for 195 countries and territories, 1990–2017: a systematic analysis for the Global Burden of Disease Study 2017. *The Lancet* 2018;392(10159):1923-94. doi: 10.1016/S0140-6736(18)32225-6
4. Beelen R, Raaschou-Nielsen O, Stafoggia M, et al. Effects of long-term exposure to air pollution on natural-cause mortality: an analysis of 22 European cohorts within the multicentre ESCAPE project. *Lancet* 2014;383(9919):785-95. doi: 10.1016/S0140-6736(13)62158-3
5. Beelen R, Stafoggia M, Raaschou-Nielsen O, et al. Long-term exposure to air pollution and cardiovascular mortality: an analysis of 22 European cohorts. *Epidemiology* 2014;25(3):368-78. doi: 10.1097/EDE.0000000000000076
6. Hvidtfeldt UA, Sorensen M, Geels C, et al. Long-term residential exposure to PM2.5, PM10, black carbon, NO2, and ozone and mortality in a Danish cohort. *Environment international* 2019;123:265-72. doi: 10.1016/j.envint.2018.12.010
7. Dimakopoulou K, Samoli E, Beelen R, et al. Air Pollution and Nonmalignant Respiratory Mortality in 16 Cohorts within the ESCAPE Project. *American journal of respiratory and critical care medicine* 2014;189(6):684-96. doi: 10.1164/rccm.201310-1777OC
8. Luben TJ, Nichols JL, Dutton SJ, et al. A systematic review of cardiovascular emergency department visits, hospital admissions and mortality associated with ambient black carbon. *Environment international* 2017;107:154-62. doi: 10.1016/j.envint.2017.07.005
9. Gan WQ, Koehoorn M, Davies HW, et al. Long-term exposure to traffic-related air pollution and the risk of coronary heart disease hospitalization and mortality. *Environmental health perspectives* 2011;119(4):501-7. doi: 10.1289/ehp.1002511
10. Ostro B, Hu JL, Goldberg D, et al. Associations of Mortality with Long-Term Exposures to Fine and Ultrafine Particles, Species and Sources: Results from the California Teachers Study Cohort. *Environmental health perspectives* 2015;123(6):549-56. doi: 10.1289/ehp.1408565
11. Ostro B, Lipsett M, Reynolds P, et al. Long-Term Exposure to Constituents of Fine Particulate Air Pollution and Mortality: Results from the California Teachers Study. *Environmental health perspectives* 2010;118(3):363-69. doi: 10.1289/ehp.0901181
12. Baxter LK, Dionisio KL, Burke J, et al. Exposure prediction approaches used in air pollution epidemiology studies: Key findings and future recommendations. *J Expo Sci Env Epid* 2013;23(6):654-59. doi: 10.1038/jes.2013.62
13. Vodonos A, Awad YA, Schwartz J. The concentration-response between long-term PM2.5 exposure and mortality; A meta-regression approach. *Environmental research* 2018;166:677-89. doi: https://doi.org/10.1016/j.envres.2018.06.021
14. Christidis T, Erickson AC, Pappin AJ, et al. Low concentrations of fine particle air pollution and mortality in the Canadian Community Health Survey cohort. *Environmental health : a global access science source* 2019;18(1):84-84. doi: 10.1186/s12940-019-0518-y
15. Pinault LL, Weichenthal S, Crouse DL, et al. Associations between fine particulate matter and mortality in the 2001 Canadian Census Health and Environment Cohort. *Environmental research* 2017;159:406-15. doi: 10.1016/j.envres.2017.08.037 [published Online First: 2017/08/30]
16. Pinault L, Tjepkema M, Crouse DL, et al. Risk estimates of mortality attributed to low concentrations of ambient fine particulate matter in the Canadian community health survey

For peer review only - http://bmjopen.bmj.com/site/about/guidelines.xhtml

cohort. *Environmental health : a global access science source* 2016;15:18-18. doi: 10.1186/s12940-016-0111-6
17. Shi L, Zanobetti A, Kloog I, et al. Low-Concentration PM2.5 and Mortality: Estimating Acute and Chronic Effects in a Population-Based Study. *Environmental health perspectives* 2016;124(1):46-52. doi: 10.1289/ehp.1409111 [published Online First: 2015/06/03]
18. WILHELMSEN L, BERGLUND G, ELMFELDT D, et al. The multifactor primary prevention trial in Göteborg, Sweden. *European heart journal* 1986;7(4):279-88. doi: 10.1093/oxfordjournals.eurheartj.a062065
19. WILHELMSEN L, JOHANSSON S, ROSENGREN A, et al. Risk factors for cardiovascular disease during the period 1985–1995 in Göteborg, Sweden. The GOT-MONICA Project. *Journal of Internal Medicine* 1997;242(3):199-211. doi: 10.1046/j.1365-2796.1997.00163.x
20. WHO Monica Project Principal Investigators. The world health organization monica project (monitoring trends and determinants in cardiovascular disease): A major international collaboration. *Journal of Clinical Epidemiology* 1988;41(2):105-14. doi: https://doi.org/10.1016/0895-4356(88)90084-4
21. Korek MJ, Bellander TD, Lind T, et al. Traffic-related air pollution exposure and incidence of stroke in four cohorts from Stockholm. *Journal of exposure science & environmental epidemiology* 2015;25(5):517-23. doi: 10.1038/jes.2015.22
22. Ljungman PLS, Andersson N, Stockfelt L, et al. Long-Term Exposure to Particulate Air Pollution, Black Carbon, and Their Source Components in Relation to Ischemic Heart Disease and Stroke. *Environmental health perspectives* 2019;127(10):107012. doi: 10.1289/EHP4757
23. Norberg M, Wall S, Boman K, et al. The Västerbotten Intervention Programme: background, design and implications. *Global health action* 2010;3 doi: 10.3402/gha.v3i0.4643 [published Online First: 2010/03/27]
24. Segersson D, Eneroth K, Gidhagen L, et al. Health Impact of PM10, PM2.5 and Black Carbon Exposure Due to Different Source Sectors in Stockholm, Gothenburg and Umea, Sweden. *International Journal of Environmental Research and Public Health* 2017;14(7) doi: Artn 742 10.3390/Ijerph14070742
25. Berkowicz R. OSPM - A Parameterised Street Pollution Model. *Environmental monitoring and assessment* 2000;65(1):323-31. doi: 10.1023/A:1006448321977
26. Hausberger S, Rexeis M, Zallinger M. Emission Factors from the Model PHEM for the HBEFA Version 3., Institute for internal combustion engines and thermodynamics, Graz University of Technology. 2009
27. Ilias V, Leonidas N, Zissis S. Methodology for the Quantification of Road Transport PM-Emissions, Using Emission Factors or Profiles. Deliverable D1.1.2, Updated February 2013. TRANSPHORM, http://www.transphorm.eu/Portals/51/Documents/Deliverables/New%20Deliverables/D1.1.2_updated.pdf [accessed 26 October 2018]. 2013
28. Denby BR, Sundvor I, Johansson C, et al. A coupled road dust and surface moisture model to predict non-exhaust road traffic induced particle emissions (NORTRIP). Part 2: Surface moisture and salt impact modelling. *Atmos Environ* 2013;81:485-503. doi: https://doi.org/10.1016/j.atmosenv.2013.09.003
29. Denby BR, Sundvor I, Johansson C, et al. A coupled road dust and surface moisture model to predict non-exhaust road traffic induced particle emissions (NORTRIP). Part 1: Road dust loading and suspension modelling. *Atmos Environ* 2013;77:283-300. doi: https://doi.org/10.1016/j.atmosenv.2013.04.069
30. Omstedt G, Bringfelt B, Johansson C. A model for vehicle-induced non-tailpipe emissions of particles along Swedish roads. *Atmos Environ* 2005;39(33):6088-97. doi: https://doi.org/10.1016/j.atmosenv.2005.06.037
31. Jalkanen JP, Johansson L, Kukkonen J, et al. Extension of an assessment model of ship traffic exhaust emissions for particulate matter and carbon monoxide. *Atmos Chem Phys* 2012;12(5):2641-59. doi: 10.5194/acp-12-2641-2012

For peer review only - http://bmjopen.bmj.com/site/about/guidelines.xhtml

32. Forsberg B, Hansson HC, Johansson C, et al. Comparative health impact assessment of local and regional particulate air pollutants in Scandinavia. *Ambio* 2005;34(1):11-9. [published Online First: 2005/03/26]
33. Dersimonian R, Laird N. Metaanalysis in Clinical-Trials. *Control Clin Trials* 1986;7(3):177-88. doi: Doi 10.1016/0197-2456(86)90046-2
34. Cesaroni G, Badaloni C, Gariazzo C, et al. Long-term exposure to urban air pollution and mortality in a cohort of more than a million adults in Rome. *Environmental health perspectives* 2013;121(3):324-31. doi: 10.1289/ehp.1205862
35. Hamra GB, Guha N, Cohen A, et al. Outdoor Particulate Matter Exposure and Lung Cancer: A Systematic Review and Meta-Analysis. *Environmental health perspectives* 2014;122(9):906-11. doi: doi:10.1289/ehp/1408092
36. Raaschou-Nielsen O, Andersen ZJ, Beelen R, et al. Air pollution and lung cancer incidence in 17 European cohorts: prospective analyses from the European Study of Cohorts for Air Pollution Effects (ESCAPE). *The Lancet Oncology* 2013;14(9):813-22. doi: https://doi.org/10.1016/S1470-2045(13)70279-1
37. Lefler JS, Higbee JD, Burnett RT, et al. Air pollution and mortality in a large, representative U.S. cohort: multiple-pollutant analyses, and spatial and temporal decompositions. *Environmental health : a global access science source* 2019;18(1):101. doi: 10.1186/s12940-019-0544-9

For peer review only - http://bmjopen.bmj.com/site/about/guidelines.xhtml

Figure 1. Boxplot and density function of PM10 concentrations, in total and separate for each cohort. Tabulated values are quartile limits (Q1-Q3) and interquartile ranges (IQR:s) together with means and standard deviations.

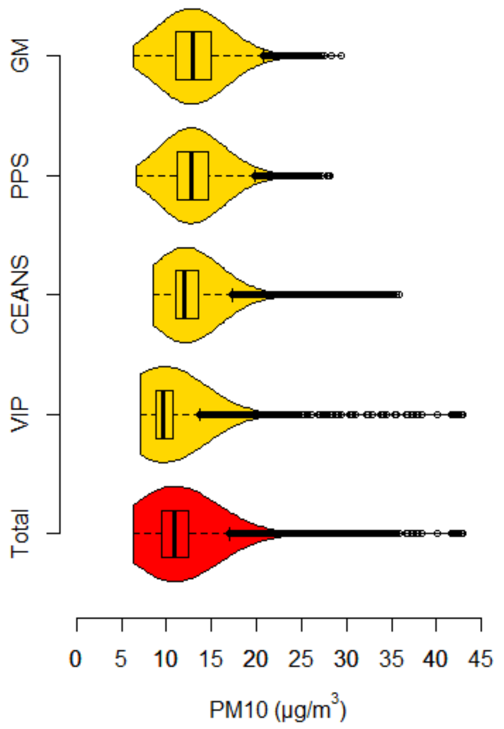

	Median (Q1-Q3)	IQR	Mean (sd)
GM	12.9 (11.1-14.9)	3.9	13.1 (2.8)
PPS	12.8 (11.3-14.7)	3.5	13.1 (2.6)
CEANS	12.0 (11.1-13.6)	2.5	12.5 (2.2)
VIP	9.6 (8.9-10.8)	1.9	9.9 (1.5)
Total	10.9 (9.5-12.5)	3.0	11.3 (2.4)

CEANS, Cardiovascular Effects of Air Pollution and Noise Study; MONICA, Multinational Monitoring of Trends and Determinants in Cardiovascular Diseases; PPS, Primary Prevention Study; VIP, Västerbotten Intervention Programme

ew only

Figure 2. Boxplot and density function of PM2.5 concentrations, in total and separate for each cohort. Tabulated values are quartile limits (Q1-Q3) and interquartile ranges (IQR:s) together with means and standard deviations.

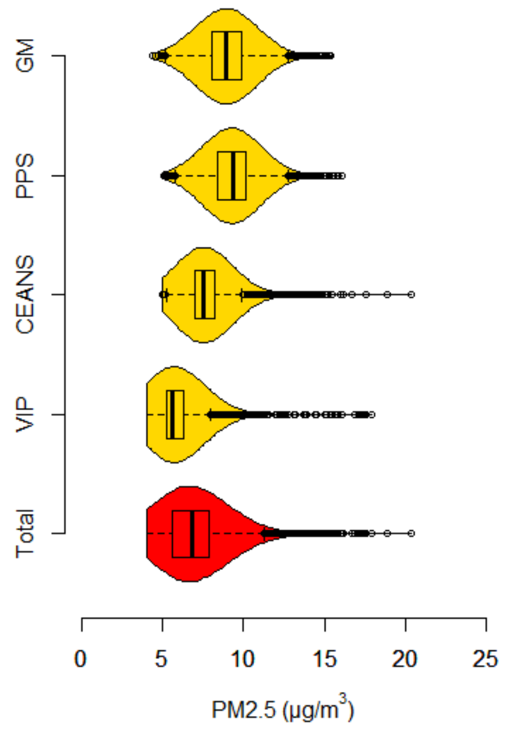

	Median (Q1-Q3)	IQR	Mean (sd)
GM	9.0 (8.0-9.9)	1.9	9.0 (1.4)
PPS	9.3 (8.4-10.2)	1.7	9.3 (1.3)
CEANS	7.5 (7.0-8.2)	1.2	7.6 (0.9)
VIP	5.7 (5.3-6.3)	1.1	5.8 (0.8)
Total	6.8 (5.6-7.9)	2.3	6.9 (1.5)

CEANS, Cardiovascular Effects of Air Pollution and Noise Study; MONICA, Multinational Monitoring of Trends and Determinants in Cardiovascular Diseases; PPS, Primary Prevention Study; VIP, Västerbotten Intervention Programme

ew only

Figure 3. Boxplot and density function of BC concentrations, in total and separate for each cohort. Tabulated values are quartile limits (Q1-Q3) and interquartile ranges (IQR:s) together with means and standard deviations.

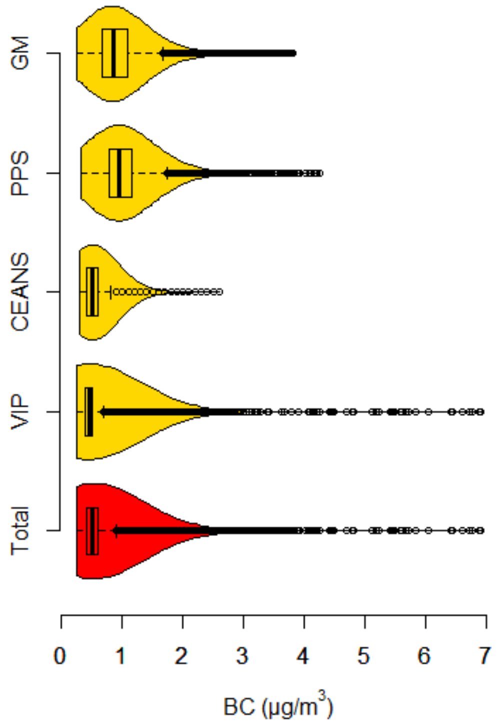

	Median (Q1-Q3)	IQR	Mean (sd)
GM	0.8 (0.7-1.1)	0.4	0.9 (0.4)
PPS	0.9 (0.8-1.2)	0.4	1.0 (0.4)
CEANS	0.5 (0.4-0.6)	0.2	0.5 (0.2)
VIP	0.4 (0.4-0.5)	0.1	0.5 (0.1)
Total	0.5 (0.4-0.6)	0.2	0.6 (0.3)

CEANS, Cardiovascular Effects of Air Pollution and Noise Study; MONICA, Multinational Monitoring of Trends and Determinants in Cardiovascular Diseases; PPS, Primary Prevention Study; VIP, Västerbotten Intervention Programme

ew only

Figure 4. Adjusted hazard ratios of natural mortality associated with PM10 within each cohort and by random effect meta-analyses estimates. Exposures were assessed by moving average residential concentrations within the exposure windows last 5 years and 6 to 10 years prior.

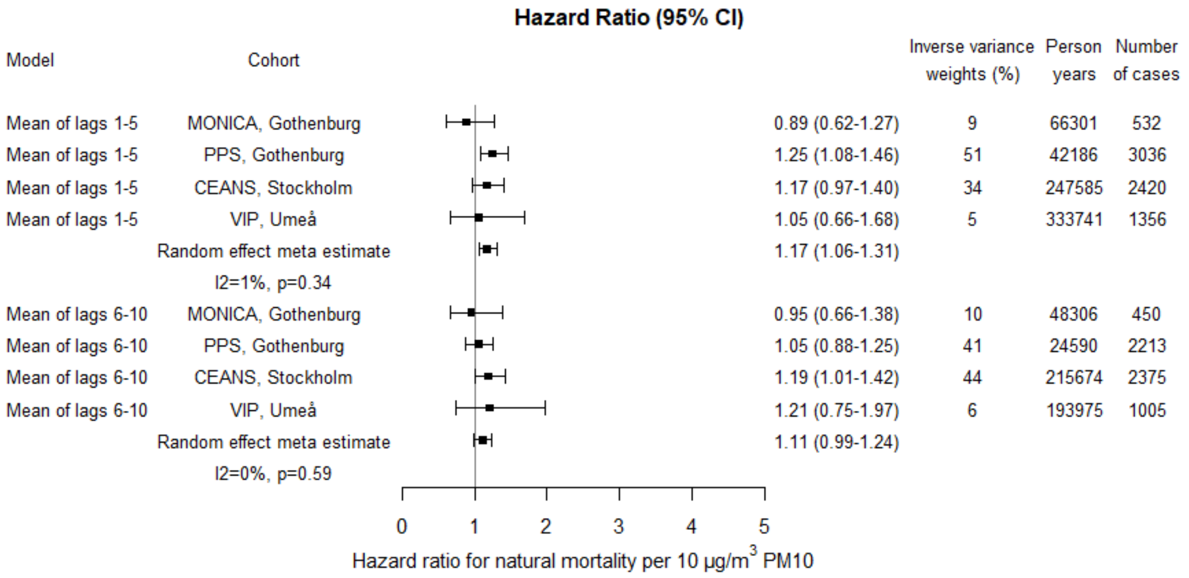

For peer review only - http://bmjopen.bmj.com/site/about/guidelines.xhtml

Figure 5. Adjusted hazard ratios of natural mortality associated with PM2.5 within each cohort and by random effect meta-analyses estimates. Exposures were assessed by moving average residential concentrations within the exposure windows last 5 years and 6 to 10 years prior.

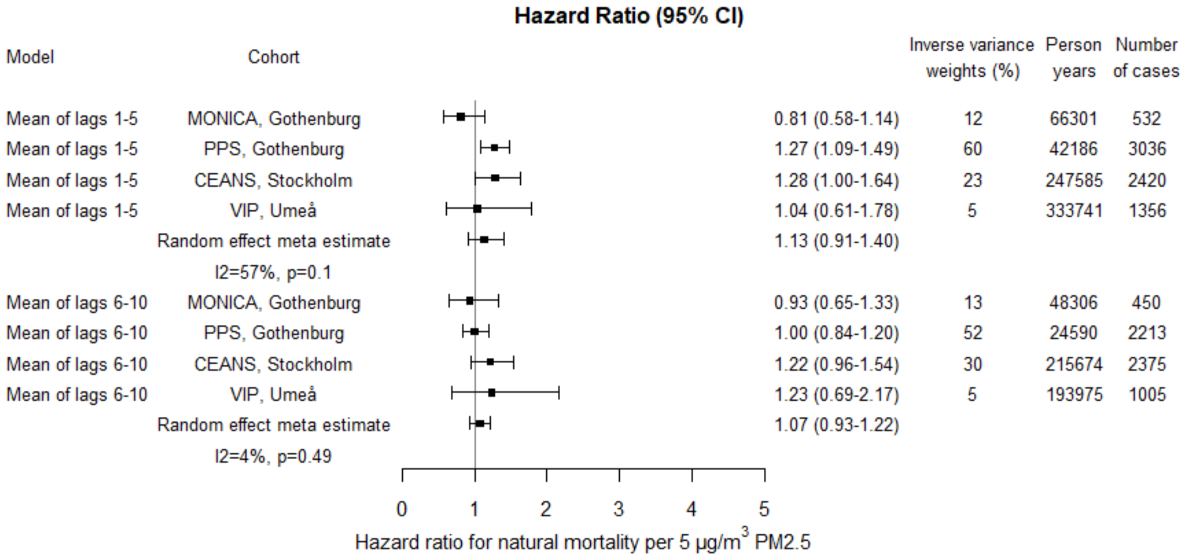

For peer review only

Figure 6. Adjusted hazard ratios of natural mortality associated with BC within each cohort and by random effect meta-analyses estimates. Exposures were assessed by moving average residential concentrations within the exposure windows last 5 years and 6 to 10 years prior.

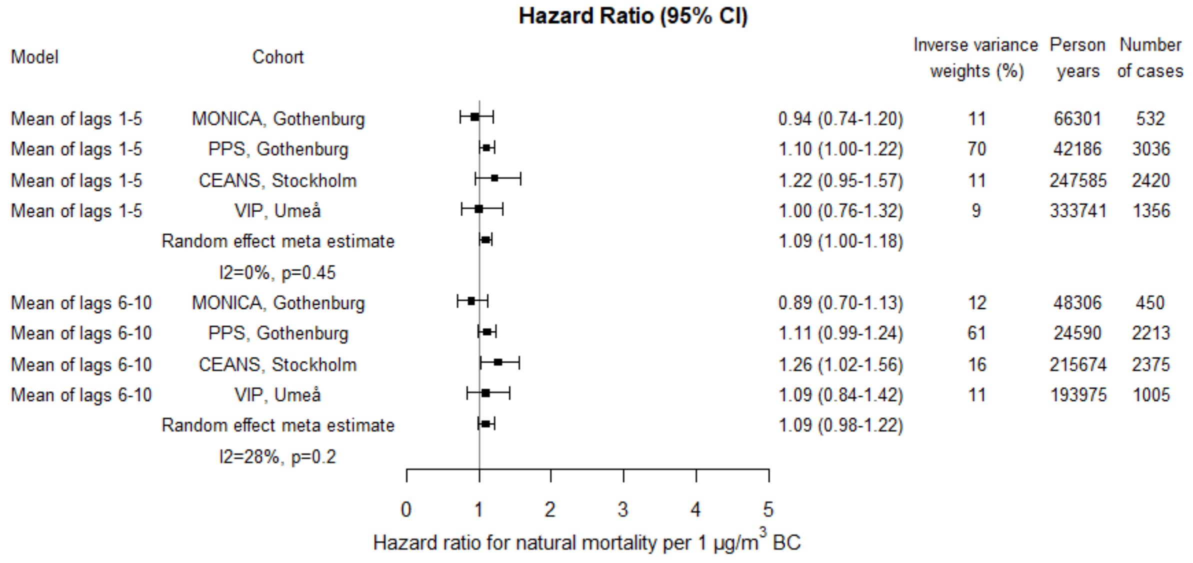

For peer review only

Figure 7. Adjusted hazard ratios of CVD mortality associated with PM10 within each cohort and by random effect meta-analyses estimates. Exposures were assessed by moving average residential concentrations within the exposure windows last 5 years and 6 to 10 years prior.

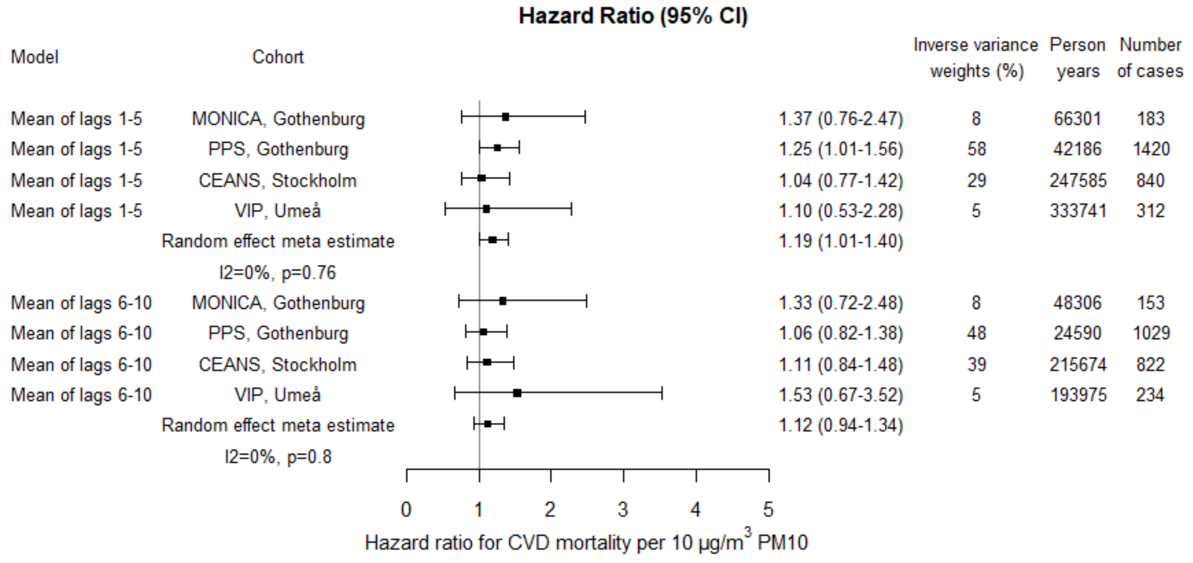

For peer review only

Figure 8. Adjusted hazard ratios of CVD mortality associated with PM2.5 within each cohort and by random effect meta-analyses estimates. Exposures were assessed by moving average residential concentrations within the exposure windows last 5 years and 6 to 10 years prior.

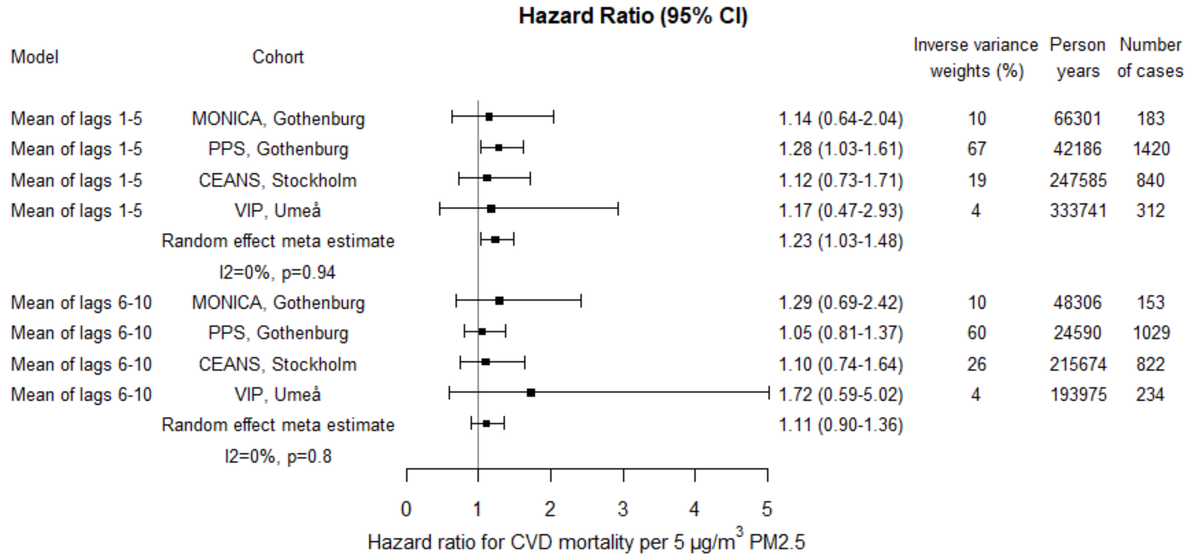

For peer review only

Figure 9. Adjusted hazard ratios of CVD mortality associated with BC within each cohort and by random effect meta-analyses estimates. Exposures were assessed by moving average residential concentrations within the exposure windows last 5 years and 6 to 10 years prior.

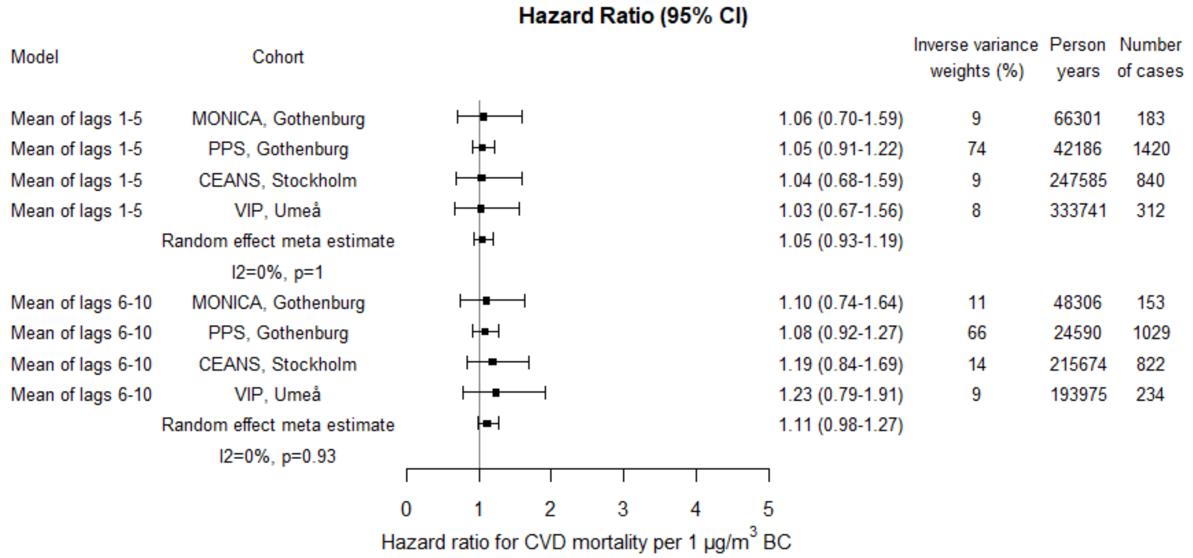

For peer review only

Figure S1a-c. Crude hazard ratios of natural mortality associated with a) PM10, b) PM2.5 and c) BC within each cohort and by random effect meta-analyses estimates. Exposures were assessed by moving average residential concentrations within the exposure windows last 5 years and 6 to 10 years prior.

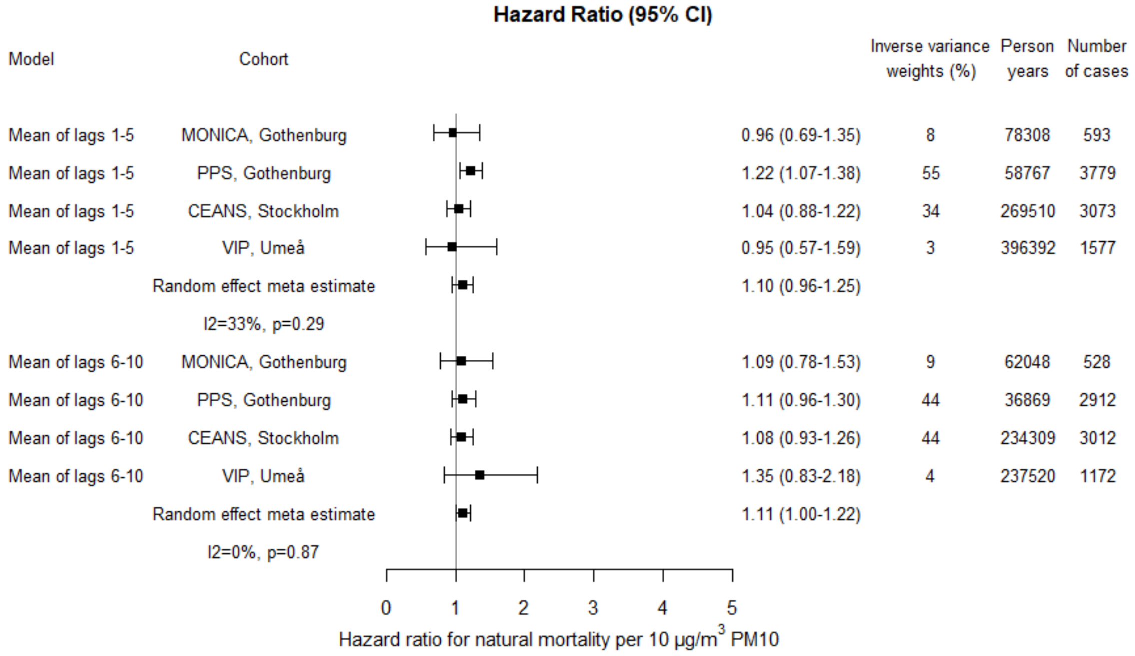

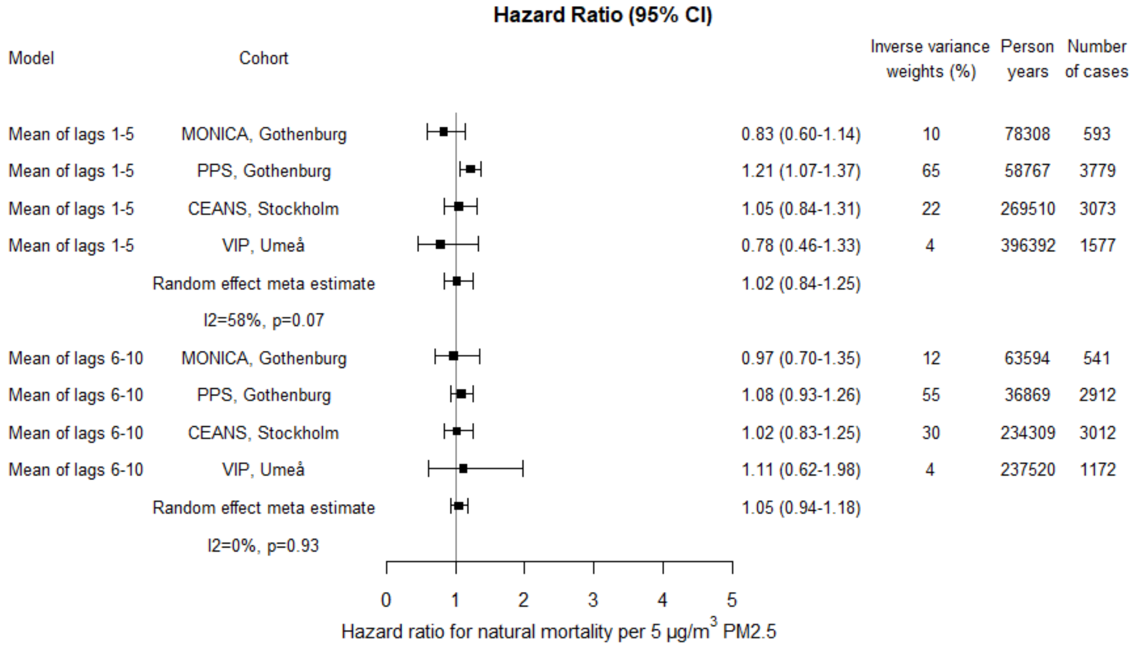

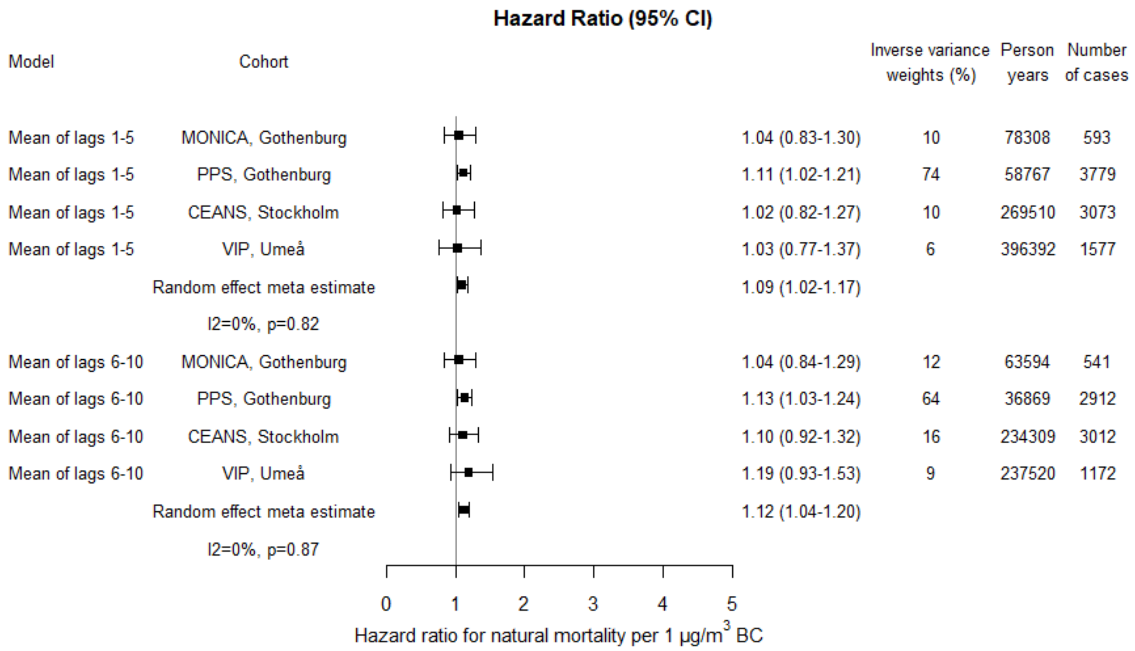
Hazard Ratio (95% CI)
Model
Cohort
Inverse variance weights (%)
Person years
Number of cases
Mean of lags 1-5 MONICA, Gothenburg 1.04 (0.83-1.30) 10 78308 593
Mean of lags 1-5 PPS, Gothenburg 1.11 (1.02-1.21) 74 58767 3779
Mean of lags 1-5 CEANS, Stockholm 1.02 (0.82-1.27) 10 269510 3073
Mean of lags 1-5 VIP, Umeå 1.03 (0.77-1.37) 6 396392 1577
Random effect meta estimate 1.09 (1.02-1.17)
I2=0%, p=0.82
Mean of lags 6-10 MONICA, Gothenburg 1.04 (0.84-1.29) 12 63594 541
Mean of lags 6-10 PPS, Gothenburg 1.13 (1.03-1.24) 64 36869 2912
Mean of lags 6-10 CEANS, Stockholm 1.10 (0.92-1.32) 16 234309 3012
Mean of lags 6-10 VIP, Umeå 1.19 (0.93-1.53) 9 237520 1172
Random effect meta estimate 1.12 (1.04-1.20)
I2=0%, p=0.87
0
1
2
3
4
5
Hazard ratio for natural mortality per 1 μg/m3 BC

Figure S2a-c. Crude hazard ratios of CVD mortality associated with a) PM10, b) PM2.5 and c) BC within each cohort and by random effect meta-analyses estimates. Exposures were assessed by moving average residential concentrations within the exposure windows last 5 years and 6 to 10 years prior.

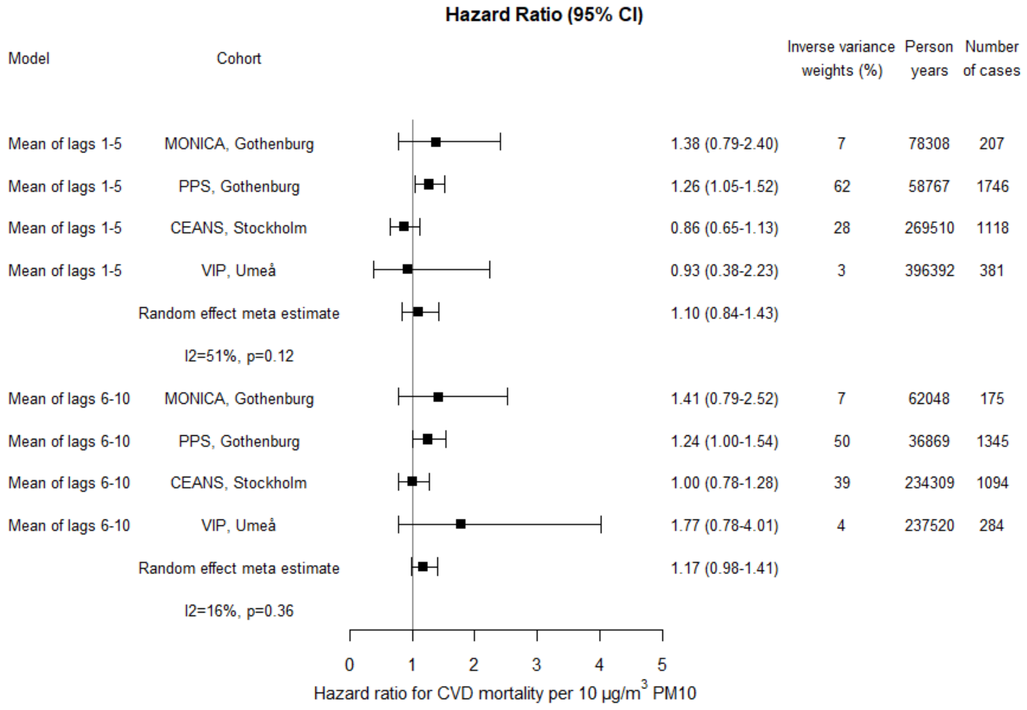

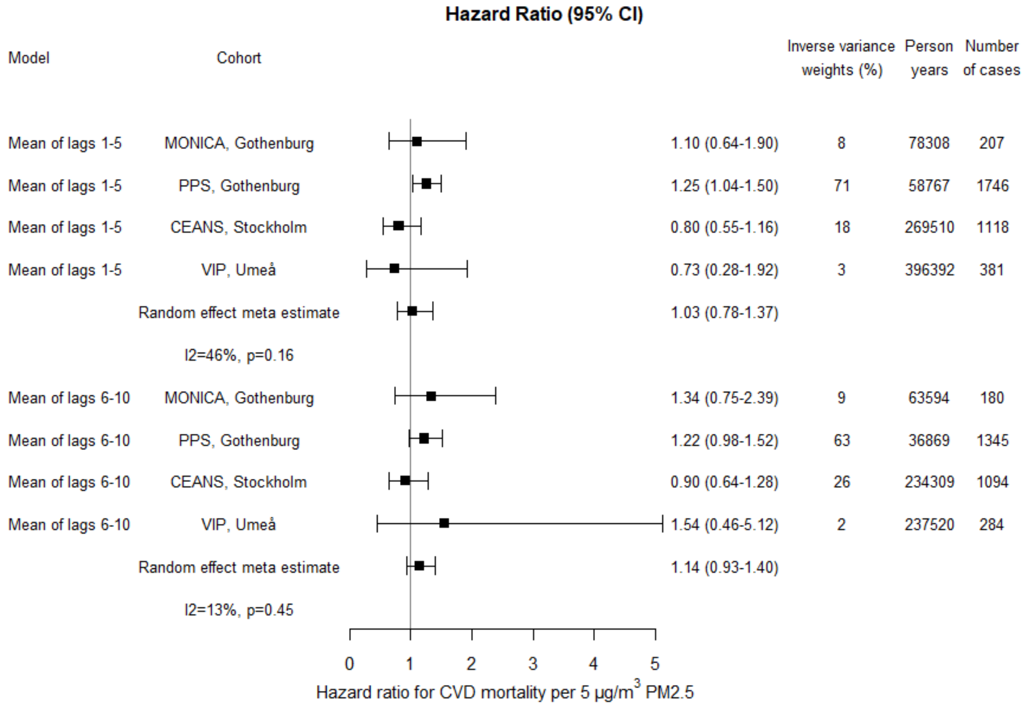

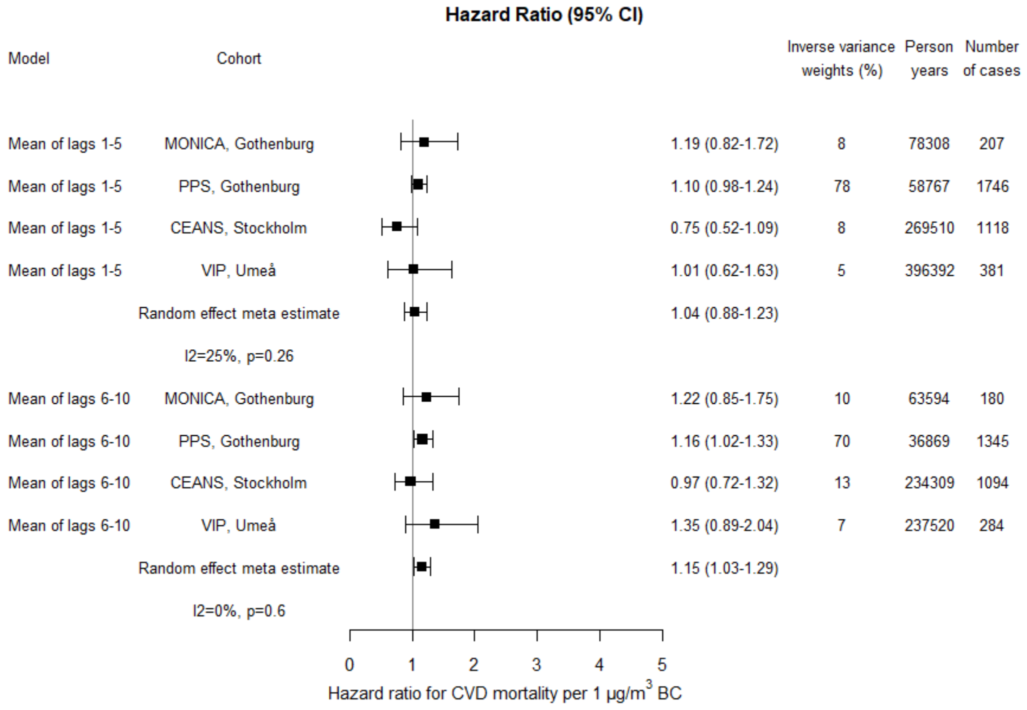
Hazard Ratio (95% CI)
Model
Cohort
Inverse variance weights (%)
Person years
Number of cases
Mean of lags 1-5 MONICA, Gothenburg 1.19 (0.82-1.72) 8 78308 207
Mean of lags 1-5 PPS, Gothenburg 1.10 (0.98-1.24) 78 58767 1746
Mean of lags 1-5 CEANS, Stockholm 0.75 (0.52-1.09) 8 269510 1118
Mean of lags 1-5 VIP, Umeå 1.01 (0.62-1.63) 5 396392 381
Random effect meta estimate 1.04 (0.88-1.23)
I2=25%, p=0.26
Mean of lags 6-10 MONICA, Gothenburg 1.22 (0.85-1.75) 10 63594 180
Mean of lags 6-10 PPS, Gothenburg 1.16 (1.02-1.33) 70 36869 1345
Mean of lags 6-10 CEANS, Stockholm 0.97 (0.72-1.32) 13 234309 1094
Mean of lags 6-10 VIP, Umeå 1.35 (0.89-2.04) 7 237520 284
Random effect meta estimate 1.15 (1.03-1.29)
I2=0%, p=0.6
0 1 2 3 4 5
Hazard ratio for CVD mortality per 1 μg/m3 BC

Figure S3a-c. Adjusted hazard ratios of lung cancer mortality associated with a) PM10, b) PM2.5 and c) BC within each cohort and by random effect meta-analyses estimates. Exposures were assessed by moving average residential concentrations within the exposure windows last 5 years and 6 to 10 years prior.

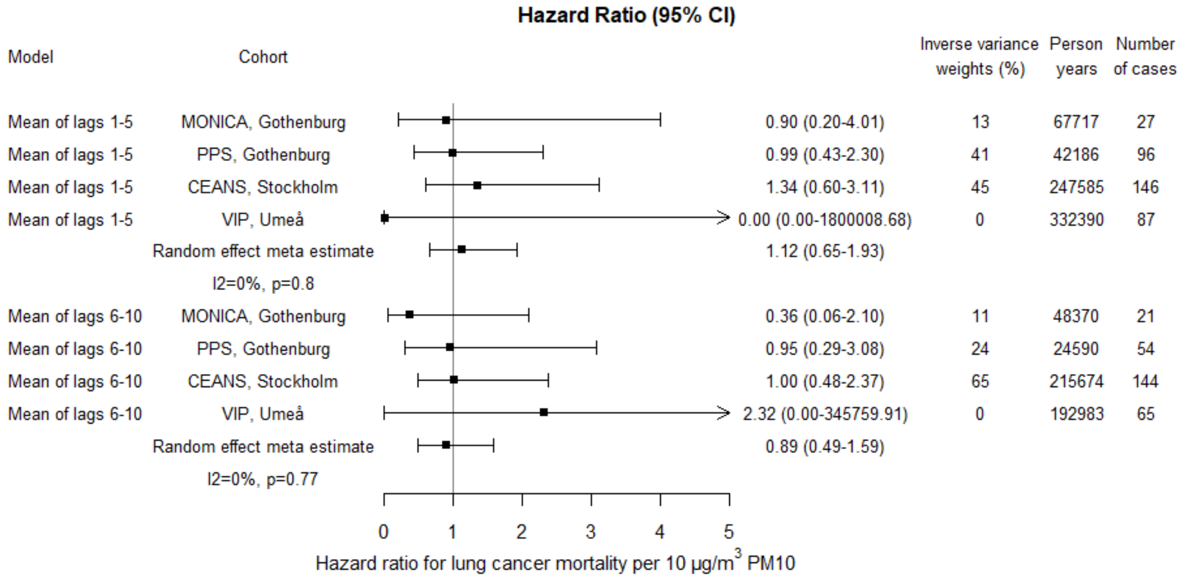

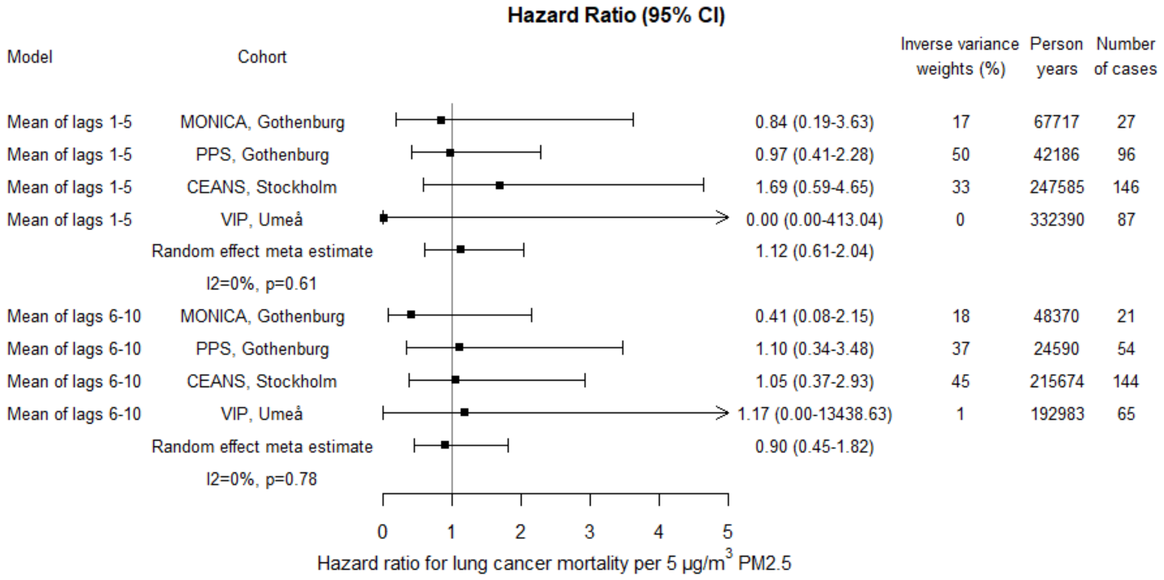

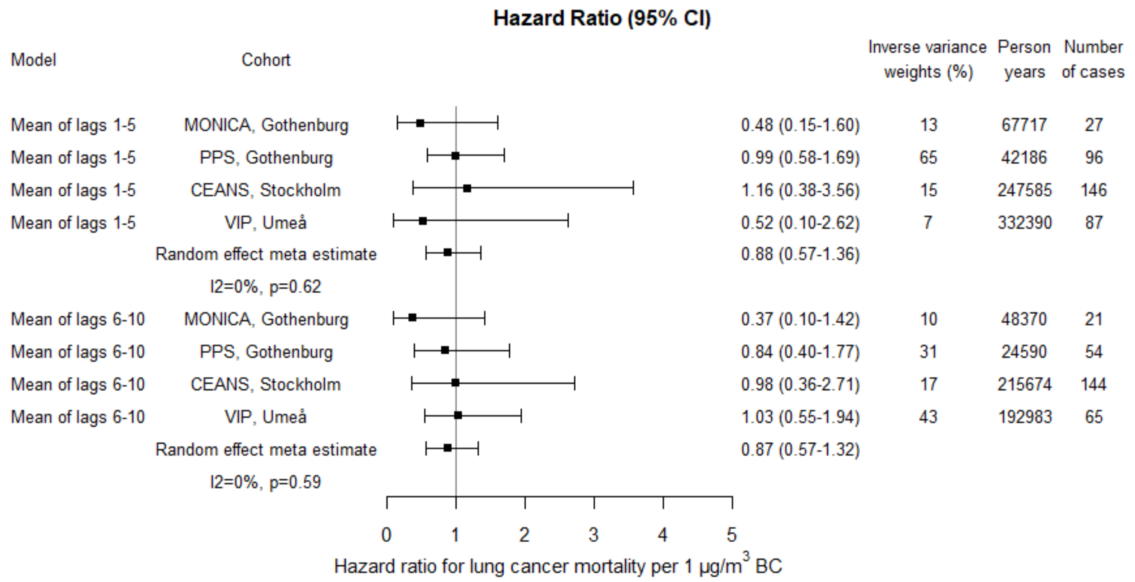

peer review only

Figure S4a-c. Adjusted hazard ratios of respiratory mortality associated with a) PM10, b) PM2.5 and c) BC within each cohort and by random effect meta-analyses estimates. Exposures were assessed by moving average residential concentrations within the exposure windows last 5 years and 6 to 10 years prior.

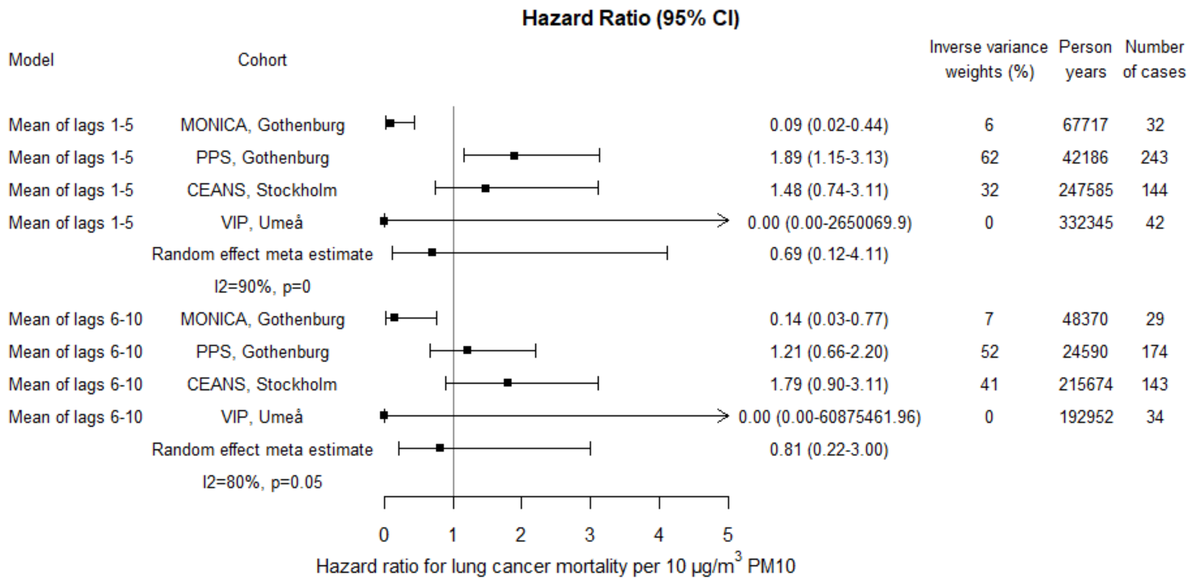

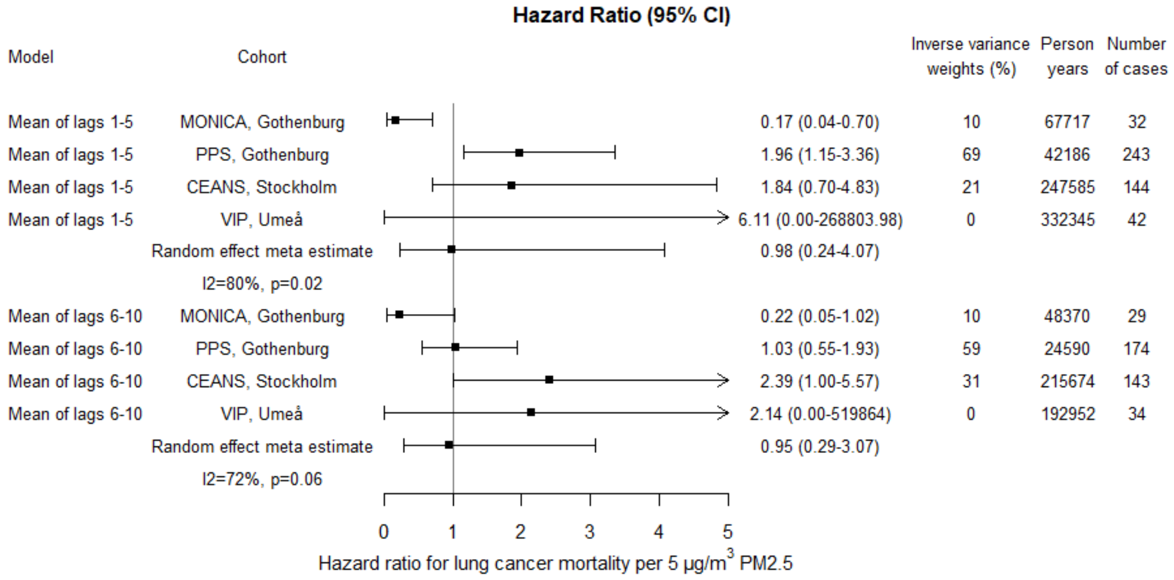

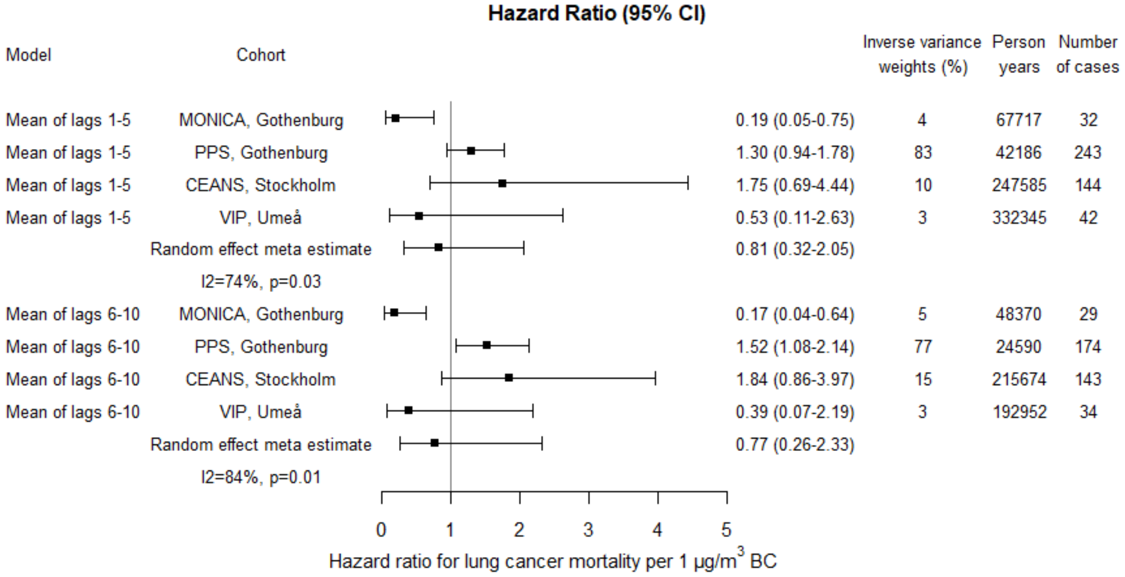

peer review only

Figure S5a-c. Adjusted hazard ratios of mortality by other casues (not CVD, respiratory, lung cancer or external causes) associated with a) PM10, b) PM2.5 and c) BC within each cohort and by random effect meta-analyses estimates. Exposures were assessed by moving average residential concentrations within the exposure windows last 5 years and 6 to 10 years prior.

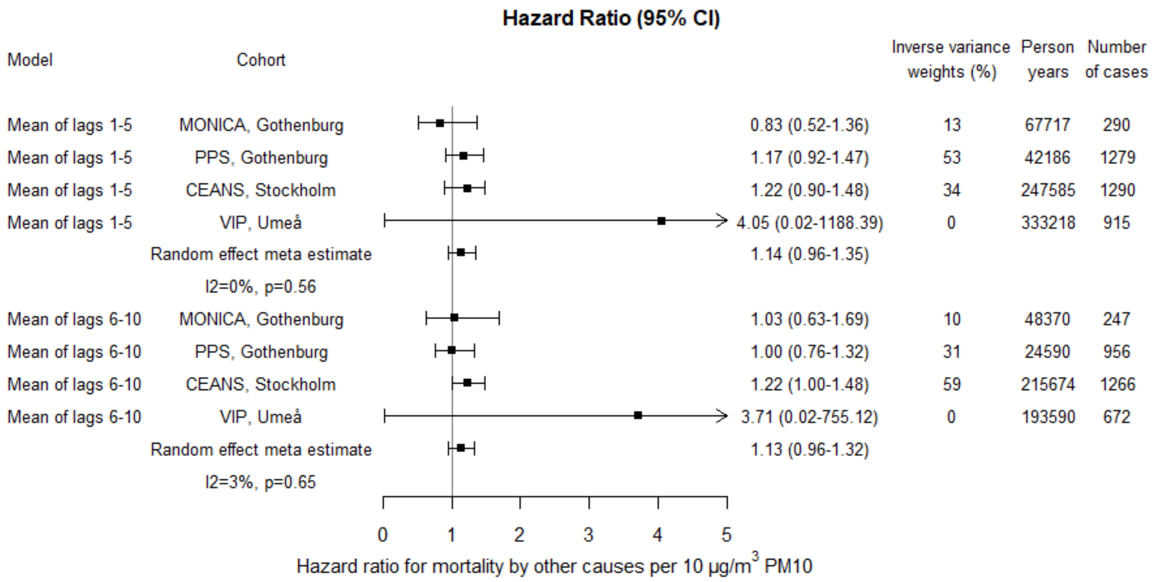

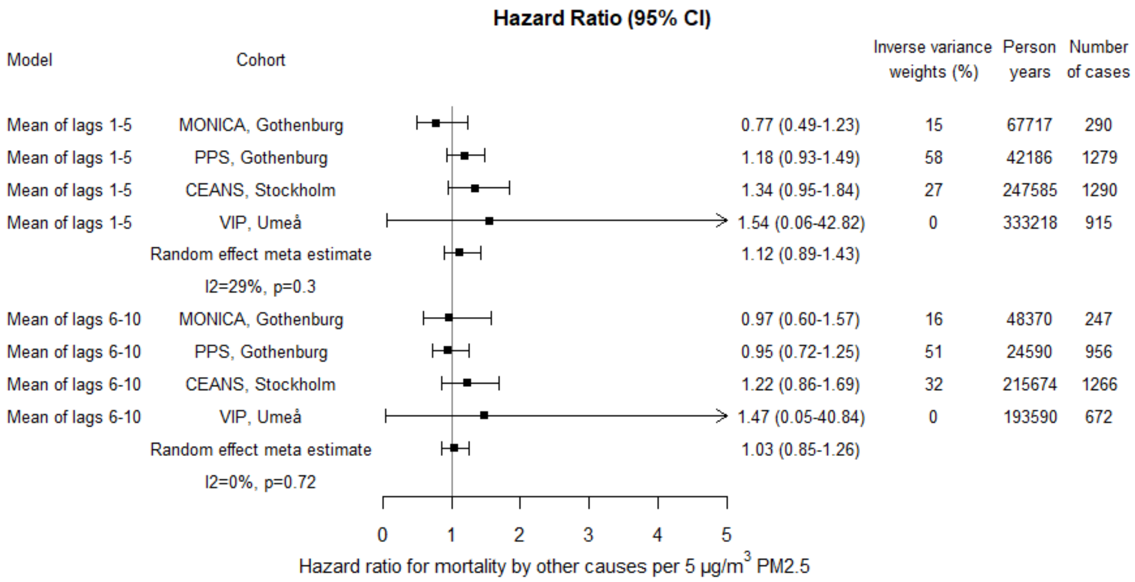

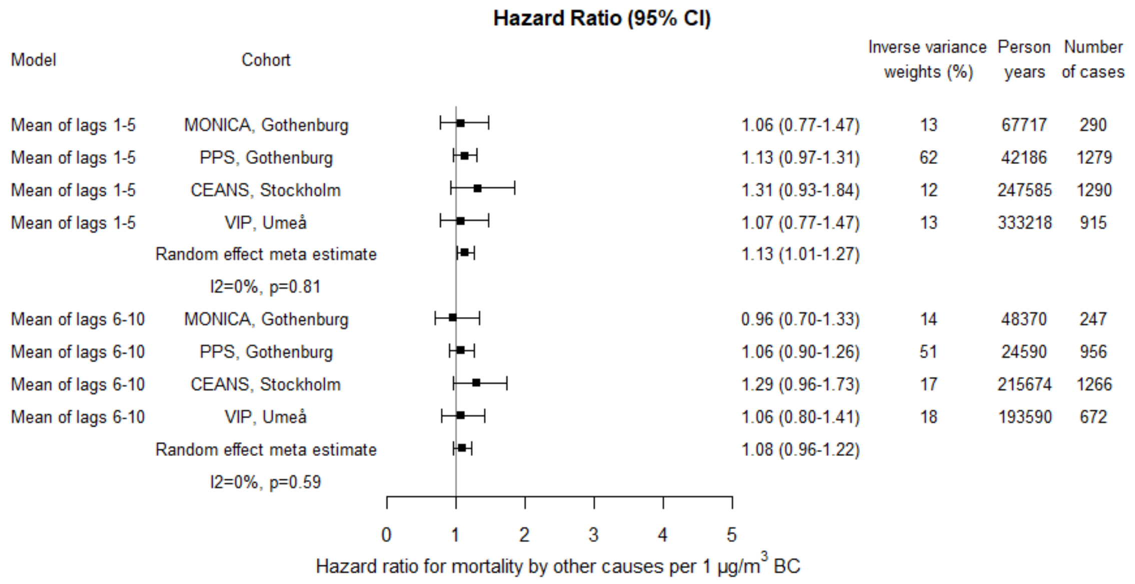

peer review only

Figure S6a-b. Adjusted hazard ratios of a) natural mortality and b) CVD mortality associated with PM10, PM2.5 and BC, within each cohort and by random effect meta-analyses estimates. Exposures were assessed by the residential concentration at the year of recruitment.

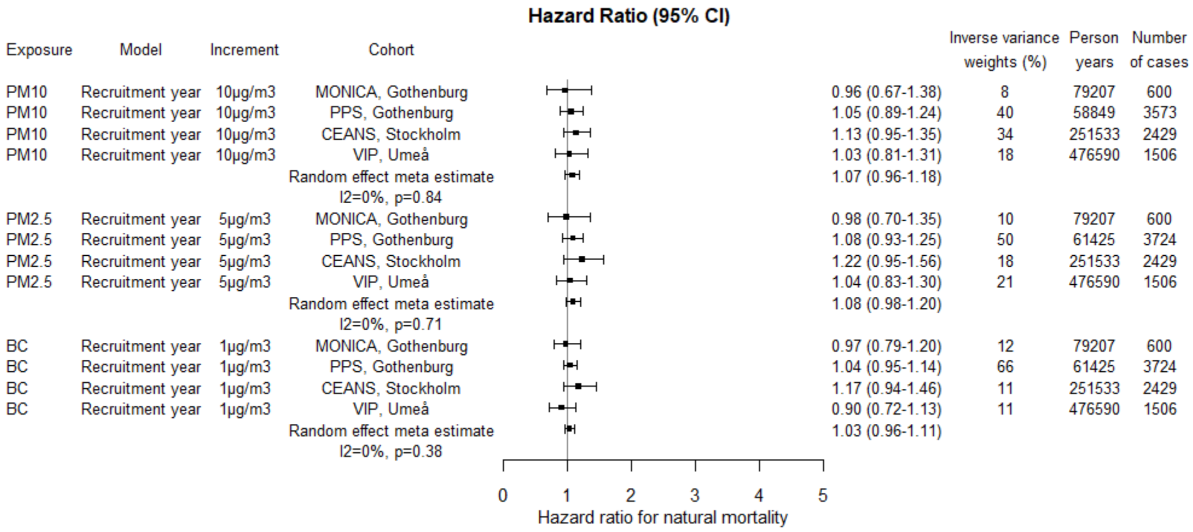

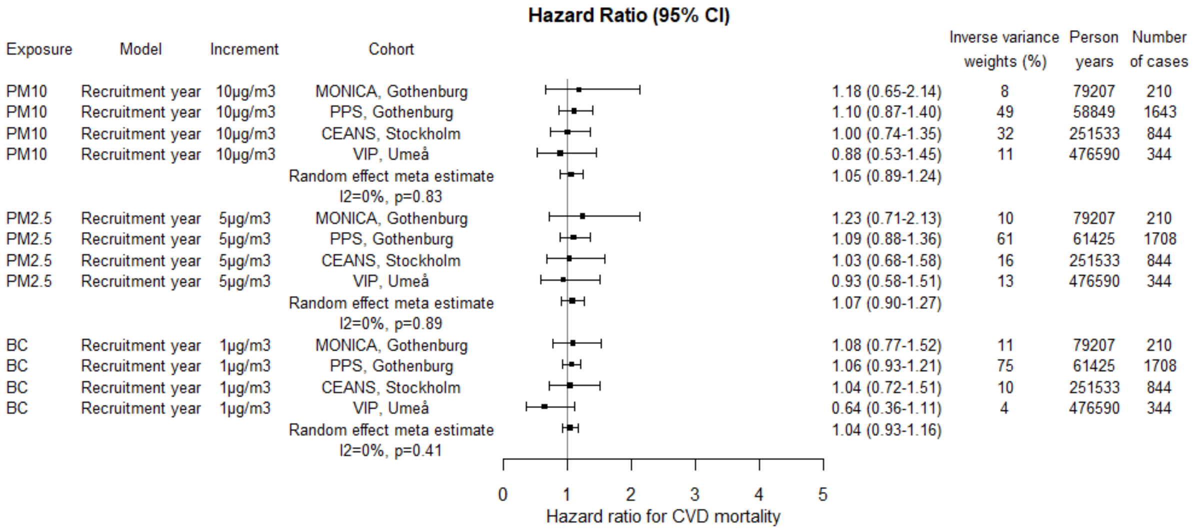

STROBE 2007 (v4) Statement—Checklist of items that should be included in reports of *cohort studies*

Section/Topic	Item #	Recommendation	Reported on page #
**Title and abstract**	1	(*a*) Indicate the study's design with a commonly used term in the title or the abstract	2
		(*b*) Provide in the abstract an informative and balanced summary of what was done and what was found	2
**Introduction**			
Background/rationale	2	Explain the scientific background and rationale for the investigation being reported	3
Objectives	3	State specific objectives, including any prespecified hypotheses	3-4
**Methods**			
Study design	4	Present key elements of study design early in the paper	4
Setting	5	Describe the setting, locations, and relevant dates, including periods of recruitment, exposure, follow-up, and data collection	4-6
Participants	6	(*a*) Give the eligibility criteria, and the sources and methods of selection of participants. Describe methods of follow-up	4-5
		(*b*) For matched studies, give matching criteria and number of exposed and unexposed	
Variables	7	Clearly define all outcomes, exposures, predictors, potential confounders, and effect modifiers. Give diagnostic criteria, if applicable	5-6
Data sources/ measurement	8*	For each variable of interest, give sources of data and details of methods of assessment (measurement). Describe comparability of assessment methods if there is more than one group	*5*
Bias	9	Describe any efforts to address potential sources of bias	10
Study size	10	Explain how the study size was arrived at	4-7
Quantitative variables	11	Explain how quantitative variables were handled in the analyses. If applicable, describe which groupings were chosen and why	5-6
Statistical methods	12	(*a*) Describe all statistical methods, including those used to control for confounding	6
		(*b*) Describe any methods used to examine subgroups and interactions	
		(*c*) Explain how missing data were addressed	6
		(*d*) If applicable, explain how loss to follow-up was addressed	6
		(*e*) Describe any sensitivity analyses	
**Results**			

For peer review only

Participants	13*	(a) Report numbers of individuals at each stage of study—eg numbers potentially eligible, examined for eligibility, confirmed eligible, included in the study, completing follow-up, and analysed	7
		(b) Give reasons for non-participation at each stage	
		(c) Consider use of a flow diagram	
Descriptive data	14*	(a) Give characteristics of study participants (eg demographic, clinical, social) and information on exposures and potential confounders	7
		(b) Indicate number of participants with missing data for each variable of interest	7
		(c) Summarise follow-up time (eg, average and total amount)	7
Outcome data	15*	Report numbers of outcome events or summary measures over time	7
Main results	16	(*a*) Give unadjusted estimates and, if applicable, confounder-adjusted estimates and their precision (eg, 95% confidence interval). Make clear which confounders were adjusted for and why they were included	7
		(*b*) Report category boundaries when continuous variables were categorized	
		(*c*) If relevant, consider translating estimates of relative risk into absolute risk for a meaningful time period	
Other analyses	17	Report other analyses done—eg analyses of subgroups and interactions, and sensitivity analyses	
**Discussion**			
Key results	18	Summarise key results with reference to study objectives	9
**Limitations**			
Interpretation	20	Give a cautious overall interpretation of results considering objectives, limitations, multiplicity of analyses, results from similar studies, and other relevant evidence	10
Generalisability	21	Discuss the generalisability (external validity) of the study results	10-11
**Other information**			
Funding	22	Give the source of funding and the role of the funders for the present study and, if applicable, for the original study on which the present article is based	12

*Give information separately for cases and controls in case-control studies and, if applicable, for exposed and unexposed groups in cohort and cross-sectional studies.

Note: An Explanation and Elaboration article discusses each checklist item and gives methodological background and published examples of transparent reporting. The STROBE checklist is best used in conjunction with this article (freely available on the Web sites of PLoS Medicine at http://www.plosmedicine.org/, Annals of Internal Medicine at http://www.annals.org/, and Epidemiology at http://www.epidem.com/). Information on the STROBE Initiative is available at www.strobe-statement.org.

For peer review only

BMJ Open

Long-term exposure to particulate air pollution and black carbon in relation to natural and cause specific mortality: a multi-cohort study in Sweden

Journal:	*BMJ Open*
Manuscript ID	bmjopen-2020-046040.R3
Article Type:	Original research
Date Submitted by the Author:	16-Jun-2021
Complete List of Authors:	Sommar, Johan; Umea University Department of Public Health and Clinical Medicine, Sustainable health<br>Andersson , Eva M.; Sahlgrenska University Hospital and University of Gothenburg, Occupational and Environmental Medicine<br>Andersson, Niklas; Institute of Environmental Medicine, Karolinska Institutet<br>Sallsten, Gerd; Sahlgrenska University Hospital and University of Gothenburg, Occupational and Environmental Medicine<br>Stockfelt, Leonard; Sahlgrenska University Hospital and University of Gothenburg, Occupational and Environmental Medicine<br>Ljungman, Petter; Karolinska Institutet<br>Segersson, David; SMHI<br>Eneroth, Kristina; SLB-analys, Environment and Health Administration<br>Gidhagen, Lars; SMHI<br>Molnar, Peter; Sahlgrenska University Hospital and University of Gothenburg, Occupational and Environmental Medicine<br>Wennberg, Patrik; Umeå University, Department of Public Health and Clinical Medicine, Family Medicine<br>Rosengren, Annika ; University of Gothenburg Sahlgrenska Academy, Institute of Medicine<br>Rizzuto, Debora; Karolinska Institutet , Aging Research Center, Department of Neurobiology, Health Care Sciences and Society; Stockholm University,<br>Leander, Karin; Karolinska Institutet, Institute of Environmental Medicine<br>Lager, Anton; Karolinska Institutet, Department of Public Health Sciences<br>Magnusson, Patrik; Karolinska Institutet, Dept. Medical Epidemiology and Biostatistics<br>Johansson, Christer; Department of Environmental Science and Analytical Chemistry<br>Barregard, Lars; Sahlgrenska University Hospital and University of Gothenburg, Occupational and Environmental Medicine<br>Bellander, Tom; Karolinska Institutet, Institute of Environmental Medicine; Stockholm County Council, Centre for Occupational and Environmental Medicine<br>Pershagen, Göran; Institute of Environmental Medicine, Karolinska Institutet<br>Forsberg, Bertil; Umeå University, Public Health and Clinical Medicine

**Primary Subject Heading**:	Epidemiology
Secondary Subject Heading:	Public health
Keywords:	EPIDEMIOLOGY, PUBLIC HEALTH, Cardiac Epidemiology < CARDIOLOGY

SCHOLARONE™
Manuscripts

I, the Submitting Author has the right to grant and does grant on behalf of all authors of the Work (as defined in the below author licence), an exclusive licence and/or a non-exclusive licence for contributions from authors who are: i) UK Crown employees; ii) where BMJ has agreed a CC-BY licence shall apply, and/or iii) in accordance with the terms applicable for US Federal Government officers or employees acting as part of their official duties; on a worldwide, perpetual, irrevocable, royalty-free basis to BMJ Publishing Group Ltd ("BMJ") its licensees and where the relevant Journal is co-owned by BMJ to the co-owners of the Journal, to publish the Work in this journal and any other BMJ products and to exploit all rights, as set out in our licence.

The Submitting Author accepts and understands that any supply made under these terms is made by BMJ to the Submitting Author unless you are acting as an employee on behalf of your employer or a postgraduate student of an affiliated institution which is paying any applicable article publishing charge ("APC") for Open Access articles. Where the Submitting Author wishes to make the Work available on an Open Access basis (and intends to pay the relevant APC), the terms of reuse of such Open Access shall be governed by a Creative Commons licence – details of these licences and which Creative Commons licence will apply to this Work are set out in our licence referred to above.

Other than as permitted in any relevant BMJ Author's Self Archiving Policies, I confirm this Work has not been accepted for publication elsewhere, is not being considered for publication elsewhere and does not duplicate material already published. I confirm all authors consent to publication of this Work and authorise the granting of this licence.

For peer review only

Long-term exposure to particulate air pollution and black carbon in relation to natural and cause specific mortality: a multi-cohort study in Sweden

Johan Nilsson Sommar[1*], Eva M. Andersson[2], Niklas Andersson[3], Gerd Sallsten[2], Leo Stockfelt[2], Petter L. S. Ljungman[3,4], David Segersson[5], Kristina Eneroth[6], Lars Gidhagen[5], Peter Molnar[2], Patrik Wennberg[7], Annika Rosengren[8], Debora Rizzuto[9,10], Karin Leander[11], Anton Lager[12,13], Patrik KE Magnusson[14], Christer Johansson[6,15], Lars Barregard[2], Tom Bellander[3,16], Göran Pershagen[3,16] and Bertil Forsberg[1]

[1] Section of Sustainable health, Department of Public Health and Clinical Medicine, Umeå University, Umeå, Sweden
[2] Occupational and Environmental Medicine, Department of Public Health and Community Medicine, Institute of medicine, Sahlgrenska Academy, University of Gothenburg & Sahlgrenska University Hospital, Sweden.
[3] Institute of Environmental Medicine, Karolinska Institutet, Stockholm, Sweden
[4] Department of Cardiology, Danderyd Hospital, Stockholm, Sweden
[5] Swedish Meteorological and Hydrological Institute, Norrköping, Sweden
[6] SLB-analys, Environment and Health Administration, Stockholm, Sweden
[7] Public Health and Clinical Medicine, Family Medicine, Umeå University, Umeå, Sweden
[8] Department of Molecular and Clinical Medicine, Institute of Medicine, University of Gothenburg, Sahlgrenska University Hospital, Gothenburg, Sweden
[9] Ageing Research Center, Department of Neurobiology, Care Sciences and Society, Karolinska Institutet and Stockholm University, Stockholm, Sweden
[10] Stockholm Gerontology Research Center, Stockholm, Sweden
[11] Institute of Environmental Medicine, Karolinska Institutet, Stockholm, Sweden
[12] Centre for Epidemiology and Community Medicine, Stockholm County Council, Stockholm, Sweden
[13] Department of Public Health Science, Karolinska Institutet, Stockholm, Sweden
[14] Department of Medical Epidemiology and Biostatistics, Karolinska Institutet, Stockholm, Sweden
[15] Department of Environmental Science, Stockholm University, Stockholm, Sweden
[16] Centre for Occupational and Environmental Medicine, Region Stockholm, Stockholm, Sweden

* Corresponding author:

Department of Public Health and Clinical Medicine, Section of Sustainable Health, Umeå University, Umeå, Sweden
Phone: +46 907853453
E-mail: johan.sommar@umu.se

For peer review only - http://bmjopen.bmj.com/site/about/guidelines.xhtml

Abstract

Objectives: To estimate concentration-response relationships for particulate matter (PM) and black carbon (BC) in relation to mortality in cohorts from three Swedish cities with comparatively low pollutant levels.

Setting: Cohorts from Gothenburg, Stockholm and Umeå, Sweden.

Design: High-resolution dispersion models were used to estimate annual mean concentrations of PM with aerodynamic diameter ≤10 µm (PM10) and ≤2.5 µm (PM2.5), and BC, at individual addresses during each year of follow up 1990-2011. Moving averages were calculated for the time windows 1-5 years (lag 1-5) and 6-10 years (lag 6-10) preceding the outcome. Cause-specific mortality data were obtained from the national cause of death registry. Cohort-specific hazard ratios were estimated using Cox regression models and then meta-analysed including a random effect of cohort.

Participants: During the study period, 7340 cases of natural mortality, 2755 cases of cardiovascular disease (CVD) mortality and 817 cases of respiratory and lung cancer mortality were observed among in total 68679 individuals and 689813 person-years of follow-up.

Results: Both PM10 (range: 6.3-41.9 µg/m^3) and BC (range: 0.2-6.8 µg/m^3) were associated with natural mortality showing 17% (95% CI 6-31%) and 9% (95% CI 0-18%) increased risks per 10 µg/m^3 and 1 µg/m^3 of lag1-5 exposure, respectively. For PM2.5 (range: 4.0-22.4 µg/m^3) the estimated increase was 13% per 5 µg/m^3, but less precise (95% CI -9-40%). Estimates for CVD mortality appeared higher for both PM10 and PM2.5. No association was observed with respiratory mortality.

Conclusion: The results support an effect of long-term air pollution on natural mortality and mortality in CVD with high relative risks also at low exposure levels. These findings are relevant for future decisions concerning air quality policies.

Key words: air pollution, particulate matter, black carbon, natural mortality, cardiovascular mortality

For peer review only

For peer review only - http://bmjopen.bmj.com/site/about/guidelines.xhtml

Strengths and limitations of this study

Few previous studies have followed up individuals by home address and time varying exposures as annual mean concentrations at actual place of residence

High-resolution dispersion models of particle concentrations were used to capture local contrasts in exposure

The study did however not include information on noise exposure and green space near the home address

This study showed associations with mortality within populations with comparatively low particle concentrations, generally below EU standards

As the first (multi)-cohort study using high resolution dispersion modelled particle concentrations at mean levels below WHO guidelines, this study found high relative risks of mortality

For peer review only - http://bmjopen.bmj.com/site/about/guidelines.xhtml

Introduction

Particulate matter ambient air pollution exposure has been associated with increased risk of premature mortality in several cohort studies [1 2]. According to the Global Burden of Diseases Study 2017 [3], assessing 84 risk factors for premature mortality in 195 countries, ambient particulate matter air pollution was ranked as the tenth most important risk factor for mortality and the most important environmental risk factor. Using risk functions restricted to cohort studies of outdoor air pollution, ambient particle matter pollution was estimated to cause 2.9 million (95% confidence interval (CI): 2.5-3.4) deaths in year 2017.

In a review of 11 cohort studies on long-term air pollution exposure and mortality, ambient concentrations of PM2.5 were associated with increased risk of all-cause mortality and particularly cardiovascular mortality. However, the heterogeneity between study relative risk estimates was large [2]. The large European Study of Cohorts for Air Pollution Effects (ESCAPE) project including 22 cohorts, observed an increased risk of overall mortality associated with ambient PM2.5 concentrations [4] whereas no association was found with cardiovascular mortality [5]. In a recent study within a large Danish cohort found increased risks for both natural cause and CVD mortality associated with PM2.5 exposure during the last 15 years [6]. Neither the Danish study nor the ESCAPE study were able to demonstrate associations with respiratory mortality [6 7].

Black carbon (BC), one component of PM, has been hypothesized to play a key role in the adverse health effects of air pollution exposure [8]. The review by Luben et al. identified three studies on the long-term effect of BC (or elemental carbon) and mortality in coronary heart disease [9] and ischemic heart disease [10 11]. However, the review did not find support for a stronger association with mortality for BC compared with PM2.5. Elemental carbon has been associated with all-cause mortality in another meta-analysis [2]. Within the ESCAPE project, PM2.5-absorbance was used as a proxy for BC. The estimated increased risk of natural mortality associated with PM2.5-absorbance did not reach statistical significance [4]. In Denmark (areas of Copenhagen and Aarhus), BC was, however, associated with both all-cause and cardiovascular mortality [6].

These previous studies varied in the method used for the exposure assessment of particle concentrations. Earlier studies assessed concentrations using centrally located monitoring stations, mostly representing the urban background [12]. Later studies use air pollution monitoring together with data on land use (land use regression, LUR) or detailed data on emissions and meteorological conditions (dispersion models). Most studies on long-term air pollution effects have assessed outdoor residential exposures at the year of inclusion into the cohort, a recent study on mortality however used annual mean concentrations on addresses during follow-up [6]. Studies using exposure assessments with a higher precision and in low-level exposure environments demonstrated higher relative risks of mortality in a recent meta-analysis [13]. Studies on low level air pollution are few, however. Adding to the knowledge from the long-term air pollution effects on mortality from the Canadian Community Health Survey-Mortality cohort [14-16] and a Medicare population in New England [17], this study is of importance for policy making and determination of limit values. Compared to these previous studies that used a 1x1 km^2 resolution for the assessment of air pollutant concentrations this study will make use of high-resolution 50x50 m^2 dispersion modelled particulate matter able to capture also local exposure contrasts.

Aim

This study aims to use high-resolution dispersion modelled annual mean residential PM10, PM2.5 and BC concentrations to estimate associations with natural and cause specific mortality in a

For peer review only - http://bmjopen.bmj.com/site/about/guidelines.xhtml

northern European environment characterized by annual averages largely compliant with EU air quality standards.

Methods and Materials

Study Cohorts

The study included four cohorts; two cohorts from Gothenburg, one from Stockholm (pooled from four cohorts) and one cohort from Umeå. Our study period ranged from January 1st 1990 to December 31st 2011 (December 31st 2013 for Umeå participants residing at the same address as in 2011). Residential address history was obtained for all cohort participants through linkage using personal identification numbers to mandatory records of residential addresses at Statistics Sweden or the Swedish Taxation Authority. These residential addresses were then geocoded by matching against the Swedish Mapping Cadastral and Land Registration Authority databases. When needed, addresses were manually checked and corrected for inconsistencies and assigned geographical coordinates.

From Gothenburg, two general population cohorts were included: The Primary Prevention Study (PPS) cohort and the GOT-MONICA cohort. The PPS cohort recruited a random third of all men in Gothenburg born between the years 1915 and 1925 who were examined in 1970-73 to study predictors of cardiovascular disease [18]. The GOT-MONICA cohort is one of the cohorts within the international MONICA project ("Multinational Monitoring of Trends and Determinants in Cardiovascular Diseases"), designed to study risk factors for cardiovascular diseases [19 20]. GOT-MONICA included residents aged 25-64 years. Recruitments were conducted in 1985, 1990 and 1995. For both cohorts, background data and information on cardiovascular risk factors were recorded by questionnaires. The recruited individuals were also examined by health care professionals.

Recruiting individuals from Stockholm County the Cardiovascular Effects of Air pollution and Noise Study (CEANS) cohort was included [21]. The cohort consists of four sub-cohorts. The Stockholm Diabetes Prevention Program [SDPP] was designed as a population-based prospective cohort, and includes 3128 men recruited between the years 1992 and 1994 and 4821 women recruited between the years 1996 and 1998. The program recruited individuals aged 35-56 years that had not previously been diagnosed with diabetes. About half of the recruited had a first or second degree relative with a history of diabetes. These were then matched with recruits of the same age and sex, who did not have a relative with a diabetes history. The second sub-cohort named SIXTY consists of a population-based random sample of 60-year old men and women (N=4232) living within Stockholm County between August 1997 and March 1999. The third cohort consists of the Screening Across the Lifespan Twin study [SALT], including twins born before year 1958. Only participants residing in Stockholm County at recruitment were included in the current analysis (N=7043). The fourth sub-cohort, the Swedish National Study on Aging and Care in Kungsholmen [SNAC-K], included a random sample of inhabitants aged at least 60 years and residing in a central area of Stockholm City (N=3363). Sub-cohort specific characteristics of included individuals in CEANS have previously been presented [22].

Including Umeå municipality, the Västerbotten intervention program (VIP) is a program where the population in the County is invited to a health examination the year they turn 40, 50 and 60 (and during some years also 30) years old [23]. The program was initiated in parts of the county in 1985 as a community and individual level program to reduce the morbidity and mortality from cardiovascular disease in Northern Sweden. The interview questions provide information about cardiovascular risk factors as well as social situation, education, diet and physical activity. So far more than 100 000 individuals have participated in the program.

For peer review only - http://bmjopen.bmj.com/site/about/guidelines.xhtml

The study was reviewed and approved by the Ethical Review Boards of Gothenburg (references T800-08 and T547- 13), Stockholm (reference 2018/2064-32), and Umeå (reference 2015/16-31Ö).

Patient and public involvement

No patient involved. The public was also not involved in the design, or conduct, or reporting, or dissemination plans of the research.

Mortality Outcomes

Cause-specific mortality was determined by linkage of national personal identification numbers to the Cause of Death Register at the Swedish National Board of Health and Welfare. We used the International Code of Diseases (ICD)9 001-779 or ICD-10 A00-R99 to define deaths by natural causes, ICD-9 400-440 or ICD-10 I10-I70 to define deaths in CVD, ICD9 162 or ICD10 C34 to define lung cancer deaths, and ICD9 460-519 or ICD10 J0-99 to define non-malignant respiratory mortality. In addition, deaths by other causes was studied (that is other than CVD, lung cancer, respiratory disease, and external causes).

Exposure Assessment

An in depth description of the dispersion model used for the exposure assessment has previously been published [24]. Both regional and local emission inventories for the years 1990, 2000 and 2011 in Gothenburg and Umeå, and years 1990, 1995, 2000, 2005 and 2011 in Stockholm, were used as input to Gaussian dispersion model simulations of annual mean concentrations of particulate matter (PM), i.e. PM10, PM2.5 and BC. To allow high resolution in vicinity of roads, a quadtree receptor grid was used, resulting in a resolution down to 35 m x 35 m. For inner-city streets with buildings on one or both sides, an additional concentration component was simulated with the Danish operational street pollution model (OSPM) [25].

Monitoring data were used in Stockholm to adjust the modelled concentrations to meteorological year-to-year variability. In Gothenburg and Umeå interpolation was used between the years and each year was also adjusted by a ventilation factor taking meteorological variability into account. Emission factors for traffic PM-exhaust for different vehicle types, speeds and driving conditions were calculated based on the Handbook on Emission Factors for Road Traffic version 3.1 [26], whereas emission factors for black carbon were based on TRANSPHORM [27]. The non-exhaust emissions of PM consist mainly of road wear particles, with a minor contribution from brake and tire wear [28-30]. For small-scale residential heating, emissions in Gothenburg and Stockholm were determined using household energy consumption data from Statistics Sweden with a resolution of 100 m x 100 m. This was based on proxy data including number of stoves or boilers in each municipality, living space of small houses per square km, population density per 100 m^2 and availability of district heating. In Umeå a detailed inventory of individual stoves and boilers was used with data from chimney sweepers and interviews about amount of wood burning [24]. Industrial and energy production facilities were included as point sources in the model. Emission from shipping were also included using a method described by Jalkanen et al. [31].

To model the long-range transport of PM10, PM2.5 and BC the difference between total concentrations measured at monitoring stations and modelled local particle concentrations at the same location was considered on an annual basis (Gothenburg and Umeå). In Stockholm annual concentrations from a regional background station was used to estimate the long-range transport. The long-range transport contributions were estimated differently for each city, but within each city they had a purely temporal contrast. This was assumed since the spatial variation between regional

For peer review only - http://bmjopen.bmj.com/site/about/guidelines.xhtml

background sites has been shown to be small [32]. These yearly contributions of background concentrations were then added to the estimated local concentrations. In a previous study, validations of total concentrations against measurements showed R2 values of 0.87, 0.65 and 0.93 for PM10, PM2.5, and BC, respectively [24].

The resulting concentrations were lastly added to each study participant's home addresses and annual exposures estimated accounting for any changes in address during the study period 1990-2011.

Confounders

Cohort-specific models were adjusted for potential confounding by including sex, calendar year, sub-cohort (in Stockholm), smoking status (current, former, never smoker), alcohol consumption (in Stockholm and Umeå; daily, weekly, seldom, never), physical activity (in Gothenburg and Umeå: sedentary, moderate, intermediate or vigorous; in Stockholm: once a month or less/>1 hour per week, about once a month/1 hour per week, 3 times a week or more/>2 hour per week), marital status (single, married or living with partner, no answer), socio-economic index by occupation (in Gothenburg (PPS) and Stockholm: blue collar, low and intermediate white collar and self-employed, high level white collar and self-employed professionals with academic degrees, no answer), education level (in Gothenburg (MONICA), Stockholm and Umeå: primary school or less, up to secondary school or equivalent, university degree or more, no answer), occupation status (in Stockholm and Umeå: gainfully employed, unemployed/not gainfully employed, retired, no answer). Area level socioeconomic status was estimated for each cohort member's residence from the mean neighborhood individual income in persons of working age by Small Areas for Market Statistics (SAMS) provided by Statistics Sweden for the calendar year 1994.

Statistical methods

Cox-proportional hazard models were used to estimate hazard ratios of mortality associated with PM and BC in cohort-specific analyses. Age was used as the underlying time variable for the baseline hazard. The regression model included adjustment for calendar year, baseline information on confounders as well as area level socio-economy. Since the Stockholm CEANS cohort consisted of four sub-cohorts that differed in age and time of recruitment, separate baseline hazards were used for the analyses within this cohort. We censured individuals at death by other causes, the end of the study period or time of permanent emigration from the study areas. Associations with PM and BC were assessed using two exposure windows; a moving average over 1-5 years preceding the event (lag1-5) and 6-10 years preceding the event (lag6-10). For inclusion, annual mean concentrations were required for at least 80% of the time window. These moving averages were used as time-varying exposures and therefore considers both between and within individual contrast during the study period. Besides these time windows, associations were also assessed with concentrations at the year of recruitment. Hazard ratios and 95% confidence intervals (CI) were expressed per 10, 5 and 1 μg/m^3 for PM10, PM2.5 and BC, respectively, as well as per interquartile ranges (IQRs) across the three study areas.

Meta-analyses of cohort-specific estimates were performed using random effects [33]. Heterogeneity between cohort estimates were assessed by the I2 statistic and statistically tested for deviations from homogeneity by a Chi-square test based on the Cochran's Q statistic.

Results

Participant characteristics

For peer review only - http://bmjopen.bmj.com/site/about/guidelines.xhtml

The total number of individuals included in our meta-analysis was 68,679, with the number of individuals included in each cohort presented in table 1. Total number of deaths by natural causes was 7344. Age at recruitment differed largely between cohorts with the highest median in the Gothenburg PPS cohort and lowest in VIP Umeå, 69 compared with 40 years. By design only males were recruited to the Gothenburg PPS cohort, whereas the other three cohorts had a slightly larger proportion of women compared with men. Baseline current smoking was highest in the Gothenburg PPS cohort and lowest in Stockholm CEANS and VIP Umeå, 39% compared with about 20%. Leisure time physical activity had a different categorization within the Stockholm CEANS cohort but it can be concluded that there was a larger proportion of individuals reporting a sedentary lifestyle at baseline in this cohort. The cohorts in Gothenburg had the largest proportion of physically active individuals, with higher frequency and intensity. Alcohol consumption was more frequent in Stockholm CEANS compared with VIP Umeå. University education was most common in Stockholm CEANS and VIP Umeå (above 30%) and less common in Gothenburg. A larger proportion of individuals were gainfully employed in the younger VIP Umeå than in Stockholm CEANS, 84% compared with 66%. Blue-collar occupation was more likely within the Gothenburg PPS cohort compared with Stockholm CEANS.

Particle concentrations

Particle concentrations of PM10, PM2.5 and BC all had the highest mean and IQR in the Gothenburg cohorts and the lowest in VIP Umeå (figure 1-3). In total, means of lag1-5 PM10, PM2.5 and BC were; 11.3, 6.9 and 0.6 µg/m^3, respectively. Lag1-5 concentrations were on average lower than lag6-10 concentrations due to a decreasing trend in concentrations during the study period. The Pearson correlation coefficient between lag 1-5 concentrations of PM10 and PM2.5 was 0.88, PM10 and BC 0.71, and between PM2.5 and BC 0.75.

Associations with mortality

Meta-estimates showed increased risks associated with PM10, PM2.5 and BC in relation to natural mortality; for lag1-5 17% (95% CI 6-31) increased risk per 10 µg/m^3 PM10, 13% (95% CI -9-40) increased risk per 5 µg/m^3 PM2.5 and 9% (95% CI 0-18) increased risk per 1 µg/m^3 BC (figure 4-6; crude estimates in supplementary material figure S1a-c). The statistical uncertainty was however considerable for the combined risk increase associated with PM2.5 and the confidence interval included 1. For PM10 and PM2.5 higher risk estimates were found for lag1-5 concentrations compared with lag6-10, whereas for BC, the risk estimates were the same. Increased risks were estimated for all cohorts except for Gothenburg MONICA, where the hazard ratio estimate for natural mortality was below 1.

The risk estimates for CVD mortality associated with PM10 and PM2.5 appeared higher compared with natural mortality; 19 (95% CI 1-40) and 23% (95% CI 3-48) increased risks for lag1-5, per 10 and 5 µg/m^3, respectively (figure 7-9; crude estimates in supplementary material figure S2a-b). The increased risks associated with BC seemed lower for CVD mortality than for natural mortality for lag1-5, but similar for lag6-10 (figure 9; crude estimates in supplementary material figure S2c).

The HRs per IQR for natural mortality were higher for PM2.5 and PM10 than for BC; 6, 5 and 2% increased risk, respectively. The same order per IQR was found for CVD mortality; 10, 5 and 1% for PM2.5, PM10 and BC, respectively.

No associations between PM and lung cancer or non-malignant respiratory mortality were observed (supplementary material figure S3-4a-c). Increased risk was, however, observed for mortality by other causes (that is other than CVD, lung cancer, respiratory disease, and external causes) with the same magnitude as observed for natural and CVD mortality (supplementary material figure S5a-c).

For peer review only - http://bmjopen.bmj.com/site/about/guidelines.xhtml

For comparison with previous studies, associations were also assessed with residential PM concentrations for the recruitment year. These estimates were in general lower compared with those with lag1-5 and lag6-10 concentrations (supplementary material figure S6a-b). Estimated risk increases for natural mortality were 7 (95% CI -4-18), 8 (95% CI -2-20) and 3% (95% CI -4-11) in association with PM10, PM2.5 and BC, per 10, 5 and 1 μg/m^3, respectively.

The heterogeneity between cohort hazard ratio estimates in relation to precision was in general very low. Heterogeneity was, however, observed for associations with lag1-5 PM2.5 and lag6-10 BC in relation to natural mortality, although non-of these were statistically significant (figure 4-9).

Discussion

We observed in four Swedish cohorts located in Gothenburg, Stockholm and Umeå increased risks associated with long-term residential outdoor levels of PM10, PM2.5 and BC in relation to natural and CVD mortality. PM10 was most consistently associated with natural and CVD mortality, whereas larger uncertainties were found for the associations between PM2.5 and CVD mortality and BC and natural mortality. Risk estimates associated with PM10 and PM2.5 appeared larger for exposures during the last 5 years in comparison to 6-10 years prior. For BC the associated risks were the same for both exposure windows in relation to natural mortality but higher for the exposure window 6-10 years prior in relation to CVD mortality. Within a previous meta-analysis of these cohorts, an association was found between BC and stroke incidence [22]. No association was, however, found between PM10 or PM2.5 and incidence in ischemic heart disease.

The observed 17% increased risk per 10 μg/m^3 PM10 in relation to natural mortality is high compared with most studies (e.g. the 6% increased risk found by Hoek et al. 2013 [2]) but similar to the recent findings within a Danish cohort presenting 12% increased risk (95% CI 3-22%) associated with a 15-year moving average of residential ambient concentrations [6]. Their estimate for CVD mortality was 30% (95% CI 11-53%) which was higher than our meta-estimate of 19%. For PM2.5 our observed risk increase by 13% per 5 μg/m^3 for natural mortality is also in agreement with the Danish estimate of 13% (95 CI 5-21%). As for PM10 the risk increase for CVD mortality associated with PM2.5 was again lower in our study, 23% compared to 29% (95% CI 13-47%). The average PM10 and PM2.5 exposure levels were 2.2 and 2.6 times higher in the Danish cohort compared to our study (lag 1-10 time-weighted concentrations from the Danish cohort compared with lag 1-5 moving averages in our study).

Using less detailed assessments of outdoor residential exposures, a meta-analysis of 22 cohorts within the ESCAPE project reported a borderline significant 4% (95% CI 0-9%) increased risk of natural-cause mortality for a 10 μg/m^3 difference in annual PM10 at the year of recruitment [4]. No association with PM10 was, however, observed for deaths in ischemic heart disease or myocardial infarction, whereas a suggested association was found in relation to deaths in cerebrovascular disease; 22% (95% CI -9-63%) increased risk per 10 μg/m^3 [5]. The findings for mortality associated with PM2.5 have been heterogeneous, however, the meta-estimate among the ESCAPE cohorts showed a 7% (95% CI 2-13%) increased risk per 5 μg/m^3 of natural mortality and 21% (95% CI -13-69%) increased risk of death in cerebrovascular disease. As for PM10 no association was found in relation to death in ischemic heart disease or myocardial infarction. Within earlier studies; a review and meta-analysis by Hoek et al. (2013) found 6% (95% CI 4-8%) and 15% (95% CI 4-27) increased risks of all-cause mortality and cardiovascular mortality per 5 μg/m^3, respectively. A stronger association between PM and mortality with cardiovascular causes compared to all natural mortality has also been reported by others [1], and is in agreement with our findings on CVD mortality; and most apparent in relation to exposures during the last 5 years.

For peer review only - http://bmjopen.bmj.com/site/about/guidelines.xhtml

In accordance with our meta-estimate for BC the Danish cohort reported a risk increase by 9% (95% CI 4-15%) per 1 µg/m3 for all-cause mortality [6]. An increased risk by 16% (95% CI 5-27%) was also observed for CVD mortality, higher than the 5% increased risk found in our study. In the review by Hoek et al. (2013) elemental carbon was found associated with a 6% (95% CI 5-7%) increased risk of all-cause mortality per 1 $\mu g/m^3$.

The lack of association between PM and non-malignant respiratory mortality in our study is in agreement with results from both the Danish study [6], the ESCAPE study [7] and a large cohort study in Rome [34]. Previous studies have, however, found an increased risk of lung cancer incidence associated with both PM10 and PM2.5 [35 36]. The statistical power for these outcomes was low in our meta-analyses due to the limited number of deaths in lung cancer and other respiratory causes.

The observed heterogeneity of PM associated risk for premature mortality between studies is possibly related to differences in particle composition, housing with differences in particle infiltration, population characteristics, and the minor differences in confounding adjustments. The air pollution dispersion model used for the exposure assessment in our study has a high spatial resolution, and for the follow up period it was also updated with new regional and local emission inventories as well as PM measurements to provide annual mean temporal PM concentrations. A higher precision in the exposure estimate have been found to produce higher relative health risk estimates [13]. The mortality risk associated with PM2.5 has recently been reported to be higher for local sources compared with distant sources, but with less precision in relative risk estimates, indicating the importance of high spatial resolution exposure data to successfully model differences in mortality [37]. The meta-analysis results in this study and the results presented by Hvidtfeldt et al. (2019) both generally provided higher hazard ratio estimates compared with previous mortality studies. Both studies also used moving averages based on annual mean concentrations at the place of residence. Besides exposure data with a high precision such a moving average over relevant time windows may be advantageous compared with residential concentrations at the year of recruitment or a time-weighted average exposure during the follow-up. These two studies also adjusted both for individual data on confounders and neighbourhood level data on socioeconomics.

Strengths and limitations

The strength of this study includes the state-of-the-art air pollution modelling used for exposure assessment. Several factors that affect the local dispersion of air pollution particles were considered including meteorological conditions, amount of traffic including vehicle types and speed, which affect exhaust and wear emissions, the width of the street, and the height of neighbouring building. This facilitates models with a high spatial resolution providing the ability to estimate an individual's exposure at the residential address. Temporal differences for each address were also accounted for by repeatedly updating the exposure model during the period of follow up. Our modelled concentrations have showed a good agreement when validated against measured concentrations by monitoring stations in city centres [24]. Due to uncertainties in data input to the dispersion model, and since modelled PM levels at the home address do not equal true personal exposure, exposure misclassifications at the individual level will nevertheless arise. True personal exposure would for instance include indoor particle concentrations at the place of residence, at the workplace, and during commuting. During the years of follow up there has been a decreasing trend in both total concentrations of PM and age specific mortality. It was therefore important to adjust for calendar year in the Cox models.

Air pollution gases were not part of our hypothesis and thus not modelled. Studies using nitrogen dioxide (NO2) and nitrogen oxides (NOx) as indicators of traffic related air pollution have also

For peer review only - http://bmjopen.bmj.com/site/about/guidelines.xhtml

reported associations with increased risk of premature mortality [2 38]. Even though there could be a nitrogen dioxide associated short-term effect on mortality independent of particle matter (e.g. Mills et al., 2016 [39]), the evidence for long-term effects when controlling for traffic related PM is weaker and exposure to particles is considered as the primary causal determinant of health effects [40].

As presented above the incidence in ischemic heart disease and stroke have previously been assessed in relation to particle concentrations on this material [22]. If the same hypothesis is repeatably tested on the same material then multiple testing would be an issue, affecting the confidence level of the uncertainty intervals presented for the estimated hazard ratios. On the other hand, results with improvements in exposure data and aggregation with similar cohorts are important to present. Parts of the Stockholm cohorts used for this study were also included in the ESCAPE project estimating the long-term effects on human health of exposure to air pollution in Europe. The period of follow-up differs between these studies, and the exposure modelling and assessment, which were in the ESCAPE-studies only performed for the year of inclusion and built on land use regression models. Also one of the cohorts from Gothenburg (PPM) has been used to study effects of air pollution but with other exposure measures [41] and outcomes [42].

Since this is an observational study it was also necessary to adjust for a set of potential confounders, which were prespecified. Mortality outcomes, measures of exposures (PM10, PM2.5 and BC) and time windows (time dependent moving averages over lags 1-5 and 6-10, respectively) were also a priori selected. Besides natural mortality associations were assessed with CVD and three (more) specific causes of death. A limitation that needs to be considered for more exploratory outcomes is that confidence intervals were not constructed to maintain a 95% familywise error rate. For the interpretation of the results this means that even though the study does not repeatably test the same hypothesis, for instance in subgroups of participants, it is for cause-specific mortality results necessary to consider that the evidence of air pollution effects need to be judged together with other studies. Specifically, for mortality by other causes (other than CVD, lung cancer, respiratory disease, and external causes), which has rarely been assessed in relation to air pollution, additional studies are needed. Limitations also include the lack of information on noise exposure and green space near the home address which could be confounders or effect-modifiers of air pollution effects.

Another strength of this study is the large number of cohort participants and ability to adjust for a large set of confounders at both individual and area level, however information on confounders from questionnaires were only available at baseline. Even though we expect the cohort participants with age ranging from 40 years to have fairly stable lifestyles and habits, such baseline variables may change, for example due to disease. No clear heterogeneity in associations between cohorts was observed, however, with only four cohorts the test had low statistical power to reject the null hypothesis of homogeneity.

This study additionally contributes with hazard ratio estimates between PM and mortality in the lower range of exposure with PM2.5 ranging between 4.0 to 20.4 $\mu g/m^3$ and PM10 ranging between 6.3 to 41.9 $\mu g/m^3$. Even though this is lower than the current European Union standards of 25 $\mu g/m^3$ for PM2.5 and (with a few exceptions) lower than 40 $\mu g/m^3$ for PM10, the study found higher relative risks for premature mortality compared with most previous studies even though mean exposures in those studies have been higher.

Conclusions

This study of four cohorts with relatively low exposures showed increased risks of mortality in association with long-term exposure to PM10, PM2.5 and BC, and thus strongly support long-term air

For peer review only - http://bmjopen.bmj.com/site/about/guidelines.xhtml

pollution associated effects. Since high risk increases were observed even at relatively low exposures, the findings are relevant for future decisions concerning air quality policies.

For peer review only

For peer review only - http://bmjopen.bmj.com/site/about/guidelines.xhtml

Tables

Table 1. Descriptive statistics of the study participants in each cohort

		Gothenburg, MONICA	Gothenburg, PPS	Stockholm, CEANS	Umeå, VIP
Participants (n)		4,500	5,850	22,314	42,580
Baseline data collection (calendar years)		1985,1990,1995	1970-74	1992-2004	1992-2014
Age at enrolment (years; median (range))		46 (25–66)	51 (47–56)	56 (35-104)	40 (40-50)
Women (%)		52	0	58	52
Smoking status (%)	Current smoker	29	39	22	19
	Former smoker	N/A	33	36	30
	Never smoker	65	27	40	49
	Missing data	5	0	1.8	1
Physical activity (%)	Once a month or less/<1h/week	N/A	N/A	61	N/A
	About once a month/~1h/week	N/A	N/A	26	N/A
	3 times a week or more/>2h/week	N/A	N/A	7.6	N/A
	Missing data	N/A	N/A	4.6	N/A
Leisure time physical activity (%)	Sedentary	18	24	N/A	36
	Moderate	62	59	N/A	42
	Intermediate and vigorous	18	17	N/A	22
	Missing data	2	1	N/A	2
Alcohol consumption (%)	Daily	N/A	N/A	6.7	2
	Weekly	N/A	N/A	55	16
	Seldom	N/A	N/A	31	42
	Never	N/A	N/A	5.6	1
	Missing data	N/A	N/A	1.8	39
Married/living with partner (%)	No	21	14	29	23
	Yes	47	86	70	77
	Missing data	32	<0.1	1.3	1
Education level (%)	Primary school or less	13	N/A	30	30
	Up to secondary school or equivalent	32[a]	N/A	36	30
	University degree & more	20	N/A	31	40
	Missing data	35	N/A	2.8	1

For peer review only

Occupation (%)	Gainfully employed	N/A	N/A	66	85
	Unemployed/not gainfully empolyed	N/A	N/A	5.8	6
	Retired	N/A	N/A	27	4
	Missing data	N/A	N/A	1.3	4
Socioeconomic index by occupation (%)	Blue collar	N/A	48	27	N/A
	Low-and intermediate white collar and self-employed	N/A	23	51	N/A
	High level white collar and self-employed professional with academic degrees	N/A	30	18	N/A
	Missing data	N/A	0	4.4	N/A
Mean income by SAMS 1994 (SEK)		154,780	148,602	N/A	130,000
Mean income by SAMS 2009 (SEK)		N/A	N/A	303,910	N/A

CEANS, Cardiovascular Effects of Air Pollution and Noise Study; MONICA, Multinational Monitoring of Trends and Determinants in Cardiovascular Diseases; N/A, not available; PPS, Primary Prevention Study; SAMS, Small Areas for Market Statistics; SEK, Swedish Krona; VIP, Västerbotten Intervention Programme.

a. Includes technical training

For peer review only

Legends to figures

Figure 1. Boxplot and density function of PM10 concentrations, in total and separate for each cohort. Tabulated values are quartile limits together with means and standard deviations.

Figure 2. Boxplot and density function of PM2.5 concentrations, in total and separate for each cohort. Tabulated values are quartile limits together with means and standard deviations.

Figure 3. Boxplot and density function of BC concentrations, in total and separate for each cohort. Tabulated values are quartile limits together with means and standard deviations.

Figure 4. Adjusted hazard ratios of natural mortality associated with PM10 within each cohort and by random effect meta-analyses estimates. Exposures were assessed by moving average residential concentrations within the exposure windows last 5 years and 6 to 10 years prior.

Figure 5. Adjusted hazard ratios of natural mortality associated with PM2.5 within each cohort and by random effect meta-analyses estimates. Exposures were assessed by moving average residential concentrations within the exposure windows last 5 years and 6 to 10 years prior.

Figure 6. Adjusted hazard ratios of natural mortality associated with BC within each cohort and by random effect meta-analyses estimates. Exposures were assessed by moving average residential concentrations within the exposure windows last 5 years and 6 to 10 years prior.

Figure 7. Adjusted hazard ratios of CVD mortality associated with PM10 within each cohort and by random effect meta-analyses estimates. Exposures were assessed by moving average residential concentrations within the exposure windows last 5 years and 6 to 10 years prior.

Figure 8. Adjusted hazard ratios of CVD mortality associated with PM2.5 within each cohort and by random effect meta-analyses estimates. Exposures were assessed by moving average residential concentrations within the exposure windows last 5 years and 6 to 10 years prior.

Figure 9. Adjusted hazard ratios of CVD mortality associated with BC within each cohort and by random effect meta-analyses estimates. Exposures were assessed by moving average residential concentrations within the exposure windows last 5 years and 6 to 10 years prior.

For peer review only

For peer review only - http://bmjopen.bmj.com/site/about/guidelines.xhtml

Author contributions

JNS performed the meta-analysis and wrote with support from BF the first draft manuscript. JNS, EA, NA and GS conducted the cohort analyses. DS, KE, LG, CJ were responsible for dispersion modelling of particles. PW, AR, DR, KL, AL, PM and PKM and were responsible for collection of cohort data. GP, BF, LB, TB, JNS, GS, LS, PL, EA and NA designed the epidemiological study. All authors contributed to the interpretation of the results, finalization of the manuscript, and approved the final manuscript.

Competing interests

All authors have completed the ICMJE uniform disclosure form at www.icmje.org/coi_disclosure.pdf and declare: no support from any organization other than those listed above for the submitted work; no financial relationships with any organizations that might have an interest in the submitted work in the previous three years; no other relationships or activities that could appear to have influenced the submitted work.

Funding

This study was funded by the Swedish Environmental Protection Agency as part of the Swedish Clean Air and Climate Research Program (grant NV-06576-13). BF and JNS was also supported by NordForsk grant no. 75007. The GOT-MONICA and PPS cohorts were supported by the Swedish Research Council, 2013-5187 (SIMSAM). The SDPP cohort was funded by the Stockholm County Council, the Swedish Research Council, the Swedish Diabetes Association, the Novo Nordisk Scandinavia, and GlaxoSmithKline. The 60YO cohort was funded by the Stockholm County Council and the Swedish Research Council. The Swedish Twin Registry is managed by Karolinska Institutet and receives funding through the Swedish Research Council under the grant no. 2017-00641. Data collection in SALT was supported by a grant from the National Institutes of Health (NIH), grant no. 1R01 AG08724. The VIP-Umeå cohort was funded by the Västerbotten County Council. PLL was supported by funding from the Swedish Research Council for Health, Working Life and Welfare (FORTE) 2015-00917 and Karolinska Institute's Strategic Research Area in Epidemiology (SFO-EPI). LB was financed by grants from the Swedish state under the agreement between the Swedish government and the county councils: the ALF-agreement (74580).

Data sharing statement

No additional data available.
Cohort data were made available for this project only and cannot be shared according to agreements with cohort owners and ethical approvals. Details regarding cohorts and variable definitions can be obtained from the corresponding author.

For peer review only - http://bmjopen.bmj.com/site/about/guidelines.xhtml

References

1. Brook RD, Rajagopalan S, Pope CA, 3rd, et al. Particulate matter air pollution and cardiovascular disease: An update to the scientific statement from the American Heart Association. *Circulation* 2010;121(21):2331-78. doi: 10.1161/CIR.0b013e3181dbece1
2. Hoek G, Krishnan RM, Beelen R, et al. Long-term air pollution exposure and cardio- respiratory mortality: a review. *Environ Health-Glob* 2013;12 doi: Artn 43 10.1186/1476-069x-12-43
3. Stanaway JD, Afshin A, Gakidou E, et al. Global, regional, and national comparative risk assessment of 84 behavioural, environmental and occupational, and metabolic risks or clusters of risks for 195 countries and territories, 1990–2017: a systematic analysis for the Global Burden of Disease Study 2017. *The Lancet* 2018;392(10159):1923-94. doi: 10.1016/S0140-6736(18)32225-6
4. Beelen R, Raaschou-Nielsen O, Stafoggia M, et al. Effects of long-term exposure to air pollution on natural-cause mortality: an analysis of 22 European cohorts within the multicentre ESCAPE project. *Lancet* 2014;383(9919):785-95. doi: 10.1016/S0140-6736(13)62158-3
5. Beelen R, Stafoggia M, Raaschou-Nielsen O, et al. Long-term exposure to air pollution and cardiovascular mortality: an analysis of 22 European cohorts. *Epidemiology* 2014;25(3):368-78. doi: 10.1097/EDE.0000000000000076
6. Hvidtfeldt UA, Sorensen M, Geels C, et al. Long-term residential exposure to PM2.5, PM10, black carbon, NO2, and ozone and mortality in a Danish cohort. *Environment international* 2019;123:265-72. doi: 10.1016/j.envint.2018.12.010
7. Dimakopoulou K, Samoli E, Beelen R, et al. Air Pollution and Nonmalignant Respiratory Mortality in 16 Cohorts within the ESCAPE Project. *American journal of respiratory and critical care medicine* 2014;189(6):684-96. doi: 10.1164/rccm.201310-1777OC
8. Luben TJ, Nichols JL, Dutton SJ, et al. A systematic review of cardiovascular emergency department visits, hospital admissions and mortality associated with ambient black carbon. *Environment international* 2017;107:154-62. doi: 10.1016/j.envint.2017.07.005
9. Gan WQ, Koehoorn M, Davies HW, et al. Long-term exposure to traffic-related air pollution and the risk of coronary heart disease hospitalization and mortality. *Environmental health perspectives* 2011;119(4):501-7. doi: 10.1289/ehp.1002511
10. Ostro B, Hu JL, Goldberg D, et al. Associations of Mortality with Long-Term Exposures to Fine and Ultrafine Particles, Species and Sources: Results from the California Teachers Study Cohort. *Environmental health perspectives* 2015;123(6):549-56. doi: 10.1289/ehp.1408565
11. Ostro B, Lipsett M, Reynolds P, et al. Long-Term Exposure to Constituents of Fine Particulate Air Pollution and Mortality: Results from the California Teachers Study. *Environmental health perspectives* 2010;118(3):363-69. doi: 10.1289/ehp.0901181
12. Baxter LK, Dionisio KL, Burke J, et al. Exposure prediction approaches used in air pollution epidemiology studies: Key findings and future recommendations. *J Expo Sci Env Epid* 2013;23(6):654-59. doi: 10.1038/jes.2013.62
13. Vodonos A, Awad YA, Schwartz J. The concentration-response between long-term PM2.5 exposure and mortality; A meta-regression approach. *Environmental research* 2018;166:677-89. doi: https://doi.org/10.1016/j.envres.2018.06.021
14. Christidis T, Erickson AC, Pappin AJ, et al. Low concentrations of fine particle air pollution and mortality in the Canadian Community Health Survey cohort. *Environmental health : a global access science source* 2019;18(1):84-84. doi: 10.1186/s12940-019-0518-y
15. Pinault LL, Weichenthal S, Crouse DL, et al. Associations between fine particulate matter and mortality in the 2001 Canadian Census Health and Environment Cohort. *Environmental research* 2017;159:406-15. doi: 10.1016/j.envres.2017.08.037 [published Online First: 2017/08/30]
16. Pinault L, Tjepkema M, Crouse DL, et al. Risk estimates of mortality attributed to low concentrations of ambient fine particulate matter in the Canadian community health survey

For peer review only - http://bmjopen.bmj.com/site/about/guidelines.xhtml

cohort. *Environmental health : a global access science source* 2016;15:18-18. doi: 10.1186/s12940-016-0111-6

17. Shi L, Zanobetti A, Kloog I, et al. Low-Concentration PM2.5 and Mortality: Estimating Acute and Chronic Effects in a Population-Based Study. *Environmental health perspectives* 2016;124(1):46-52. doi: 10.1289/ehp.1409111 [published Online First: 2015/06/03]
18. WILHELMSEN L, BERGLUND G, ELMFELDT D, et al. The multifactor primary prevention trial in Göteborg, Sweden. *European heart journal* 1986;7(4):279-88. doi: 10.1093/oxfordjournals.eurheartj.a062065
19. WILHELMSEN L, JOHANSSON S, ROSENGREN A, et al. Risk factors for cardiovascular disease during the period 1985–1995 in Göteborg, Sweden. The GOT-MONICA Project. *Journal of Internal Medicine* 1997;242(3):199-211. doi: 10.1046/j.1365-2796.1997.00163.x
20. Investigators WMPP. The world health organization monica project (monitoring trends and determinants in cardiovascular disease): A major international collaboration. *Journal of Clinical Epidemiology* 1988;41(2):105-14. doi: https://doi.org/10.1016/0895-4356(88)90084-4
21. Korek MJ, Bellander TD, Lind T, et al. Traffic-related air pollution exposure and incidence of stroke in four cohorts from Stockholm. *Journal of exposure science & environmental epidemiology* 2015;25(5):517-23. doi: 10.1038/jes.2015.22
22. Ljungman PLS, Andersson N, Stockfelt L, et al. Long-Term Exposure to Particulate Air Pollution, Black Carbon, and Their Source Components in Relation to Ischemic Heart Disease and Stroke. *Environmental health perspectives* 2019;127(10):107012. doi: 10.1289/EHP4757
23. Norberg M, Wall S, Boman K, et al. The Västerbotten Intervention Programme: background, design and implications. *Global health action* 2010;3 doi: 10.3402/gha.v3i0.4643 [published Online First: 2010/03/27]
24. Segersson D, Eneroth K, Gidhagen L, et al. Health Impact of PM10, PM2.5 and Black Carbon Exposure Due to Different Source Sectors in Stockholm, Gothenburg and Umea, Sweden. *International Journal of Environmental Research and Public Health* 2017;14(7) doi: Artn 742 10.3390/Ijerph14070742
25. Berkowicz R. OSPM - A Parameterised Street Pollution Model. *Environmental monitoring and assessment* 2000;65(1):323-31. doi: 10.1023/A:1006448321977
26. Hausberger S, Rexeis M, Zallinger M. Emission Factors from the Model PHEM for the HBEFA Version 3., Institute for internal combustion engines and thermodynamics, Graz University of Technology. 2009
27. Ilias V, Leonidas N, Zissis S. Methodology for the Quantification of Road Transport PM-Emissions, Using Emission Factors or Profiles. Deliverable D1.1.2, Updated February 2013. TRANSPHORM, http://www.transphorm.eu/Portals/51/Documents/Deliverables/New%20Deliverables/D1.1.2_updated.pdf [accessed 26 October 2018]. 2013
28. Denby BR, Sundvor I, Johansson C, et al. A coupled road dust and surface moisture model to predict non-exhaust road traffic induced particle emissions (NORTRIP). Part 2: Surface moisture and salt impact modelling. *Atmos Environ* 2013;81:485-503. doi: https://doi.org/10.1016/j.atmosenv.2013.09.003
29. Denby BR, Sundvor I, Johansson C, et al. A coupled road dust and surface moisture model to predict non-exhaust road traffic induced particle emissions (NORTRIP). Part 1: Road dust loading and suspension modelling. *Atmos Environ* 2013;77:283-300. doi: https://doi.org/10.1016/j.atmosenv.2013.04.069
30. Omstedt G, Bringfelt B, Johansson C. A model for vehicle-induced non-tailpipe emissions of particles along Swedish roads. *Atmos Environ* 2005;39(33):6088-97. doi: https://doi.org/10.1016/j.atmosenv.2005.06.037
31. Jalkanen JP, Johansson L, Kukkonen J, et al. Extension of an assessment model of ship traffic exhaust emissions for particulate matter and carbon monoxide. *Atmos Chem Phys* 2012;12(5):2641-59. doi: 10.5194/acp-12-2641-2012

For peer review only - http://bmjopen.bmj.com/site/about/guidelines.xhtml

32. Forsberg B, Hansson HC, Johansson C, et al. Comparative health impact assessment of local and regional particulate air pollutants in Scandinavia. *Ambio* 2005;34(1):11-9. [published Online First: 2005/03/26]
33. Dersimonian R, Laird N. Metaanalysis in Clinical-Trials. *Control Clin Trials* 1986;7(3):177-88. doi: Doi 10.1016/0197-2456(86)90046-2
34. Cesaroni G, Badaloni C, Gariazzo C, et al. Long-term exposure to urban air pollution and mortality in a cohort of more than a million adults in Rome. *Environmental health perspectives* 2013;121(3):324-31. doi: 10.1289/ehp.1205862
35. Hamra GB, Guha N, Cohen A, et al. Outdoor Particulate Matter Exposure and Lung Cancer: A Systematic Review and Meta-Analysis. *Environmental health perspectives* 2014;122(9):906-11. doi: doi:10.1289/ehp/1408092
36. Raaschou-Nielsen O, Andersen ZJ, Beelen R, et al. Air pollution and lung cancer incidence in 17 European cohorts: prospective analyses from the European Study of Cohorts for Air Pollution Effects (ESCAPE). *The Lancet Oncology* 2013;14(9):813-22. doi: https://doi.org/10.1016/S1470-2045(13)70279-1
37. Lefler JS, Higbee JD, Burnett RT, et al. Air pollution and mortality in a large, representative U.S. cohort: multiple-pollutant analyses, and spatial and temporal decompositions. *Environmental health : a global access science source* 2019;18(1):101. doi: 10.1186/s12940-019-0544-9
38. Atkinson RW, Butland BK, Anderson HR, et al. Long-term Concentrations of Nitrogen Dioxide and Mortality: A Meta-analysis of Cohort Studies. *Epidemiology* 2018;29(4):460-72. doi: 10.1097/ede.0000000000000847 [published Online First: 2018/05/11]
39. Mills IC, Atkinson RW, Anderson HR, et al. Distinguishing the associations between daily mortality and hospital admissions and nitrogen dioxide from those of particulate matter: a systematic review and meta-analysis. *BMJ Open* 2016;6(7):e010751. doi: 10.1136/bmjopen-2015-010751
40. WHO. Review of evidence on health aspects of air pollution - REVIHAAP Project: Technical Report. Copenhagen2013.
41. Stockfelt L, Andersson EM, Molnár P, et al. Long term effects of residential NOx exposure on total and cause-specific mortality and incidence of myocardial infarction in a Swedish cohort. *Environmental research* 2015;142:197-206. doi: https://doi.org/10.1016/j.envres.2015.06.045
42. Andersson EM, Ogren M, Molnar P, et al. Road traffic noise, air pollution and cardiovascular events in a Swedish cohort. *Environmental research* 2020;185:109446. doi: 10.1016/j.envres.2020.109446 [published Online First: 2020/04/12]

For peer review only - http://bmjopen.bmj.com/site/about/guidelines.xhtml

Figure 1. Boxplot and density function of PM10 concentrations, in total and separate for each cohort. Tabulated values are quartile limits (Q1-Q3) and interquartile ranges (IQR:s) together with means and standard deviations.

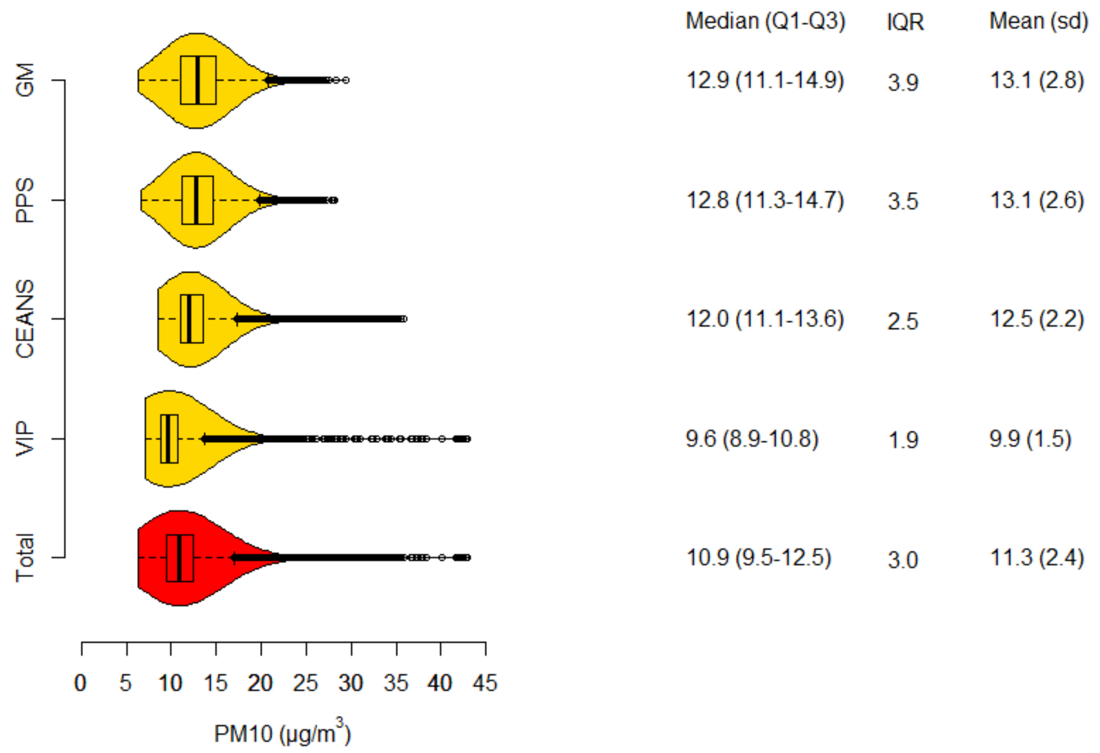

CEANS, Cardiovascular Effects of Air Pollution and Noise Study; MONICA, Multinational Monitoring of Trends and Determinants in Cardiovascular Diseases; PPS, Primary Prevention Study; VIP, Västerbotten Intervention Programme

ew only

Figure 2. Boxplot and density function of PM2.5 concentrations, in total and separate for each cohort. Tabulated values are quartile limits (Q1-Q3) and interquartile ranges (IQR:s) together with means and standard deviations.

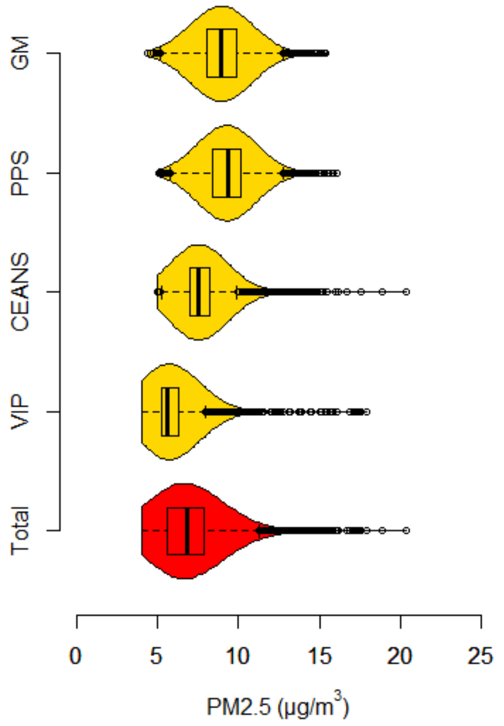

	Median (Q1-Q3)	IQR	Mean (sd)
GM	9.0 (8.0-9.9)	1.9	9.0 (1.4)
PPS	9.3 (8.4-10.2)	1.7	9.3 (1.3)
CEANS	7.5 (7.0-8.2)	1.2	7.6 (0.9)
VIP	5.7 (5.3-6.3)	1.1	5.8 (0.8)
Total	6.8 (5.6-7.9)	2.3	6.9 (1.5)

CEANS, Cardiovascular Effects of Air Pollution and Noise Study; MONICA, Multinational Monitoring of Trends and Determinants in Cardiovascular Diseases; PPS, Primary Prevention Study; VIP, Västerbotten Intervention Programme

ew only

Figure 3. Boxplot and density function of BC concentrations, in total and separate for each cohort. Tabulated values are quartile limits (Q1-Q3) and interquartile ranges (IQR:s) together with means and standard deviations.

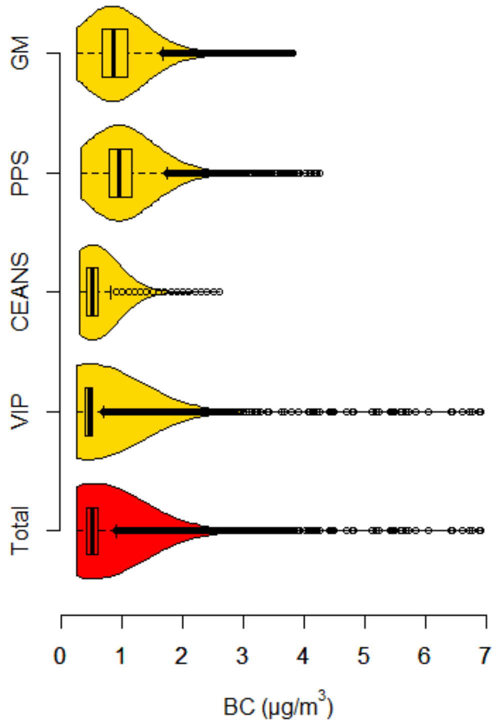

	Median (Q1-Q3)	IQR	Mean (sd)
GM	0.8 (0.7-1.1)	0.4	0.9 (0.4)
PPS	0.9 (0.8-1.2)	0.4	1.0 (0.4)
CEANS	0.5 (0.4-0.6)	0.2	0.5 (0.2)
VIP	0.4 (0.4-0.5)	0.1	0.5 (0.1)
Total	0.5 (0.4-0.6)	0.2	0.6 (0.3)

CEANS, Cardiovascular Effects of Air Pollution and Noise Study; MONICA, Multinational Monitoring of Trends and Determinants in Cardiovascular Diseases; PPS, Primary Prevention Study; VIP, Västerbotten Intervention Programme

ew only

Figure 4. Adjusted hazard ratios of natural mortality associated with PM10 within each cohort and by random effect meta-analyses estimates. Exposures were assessed by moving average residential concentrations within the exposure windows last 5 years and 6 to 10 years prior.

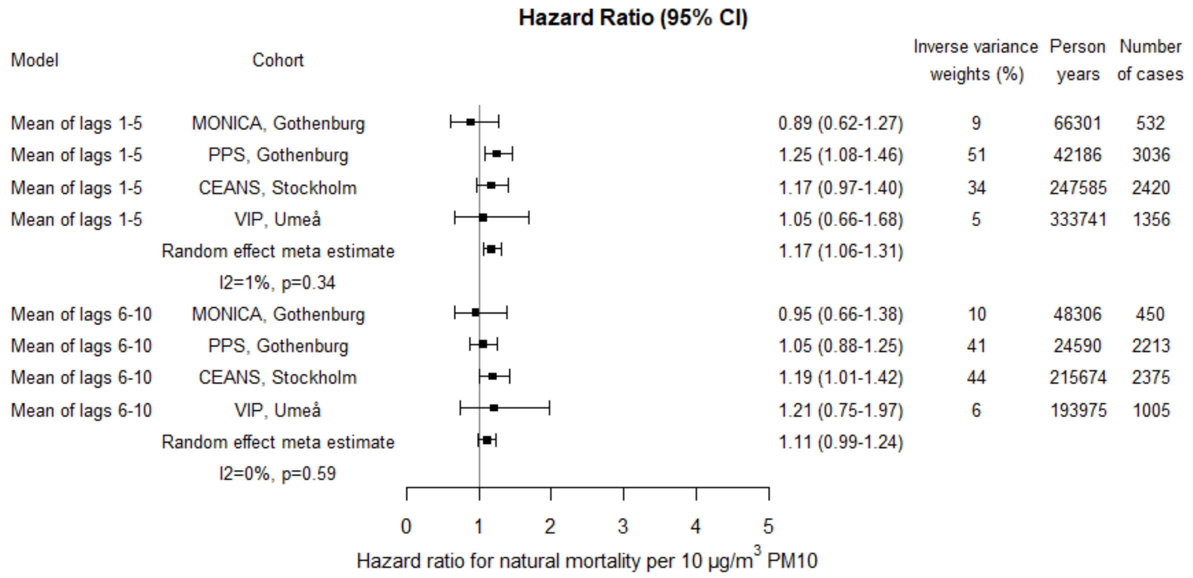

For peer review only

Figure 5. Adjusted hazard ratios of natural mortality associated with PM2.5 within each cohort and by random effect meta-analyses estimates. Exposures were assessed by moving average residential concentrations within the exposure windows last 5 years and 6 to 10 years prior.

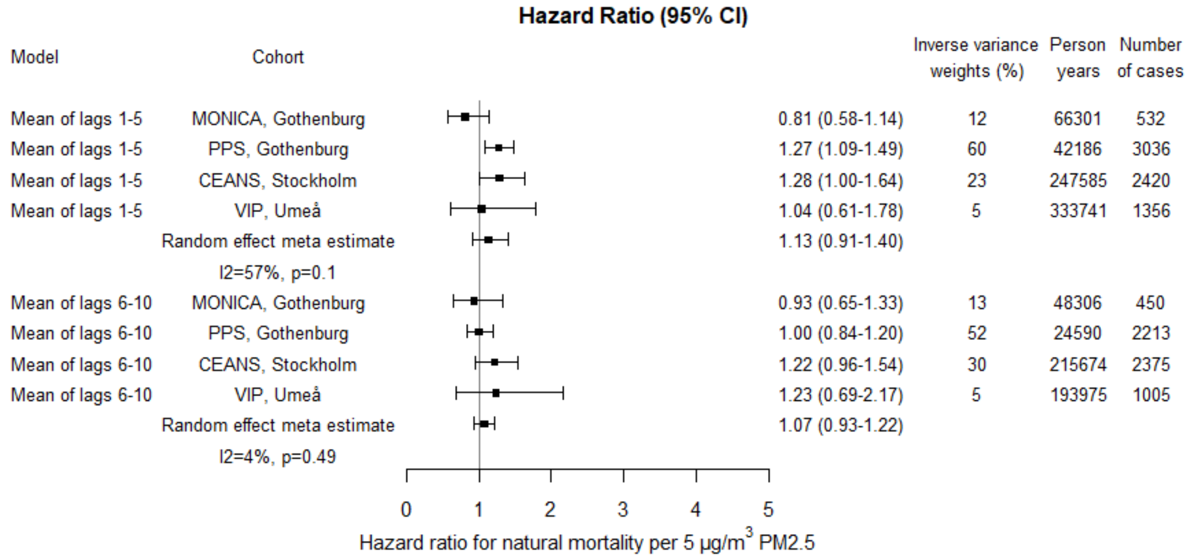

For peer review only

Figure 6. Adjusted hazard ratios of natural mortality associated with BC within each cohort and by random effect meta-analyses estimates. Exposures were assessed by moving average residential concentrations within the exposure windows last 5 years and 6 to 10 years prior.

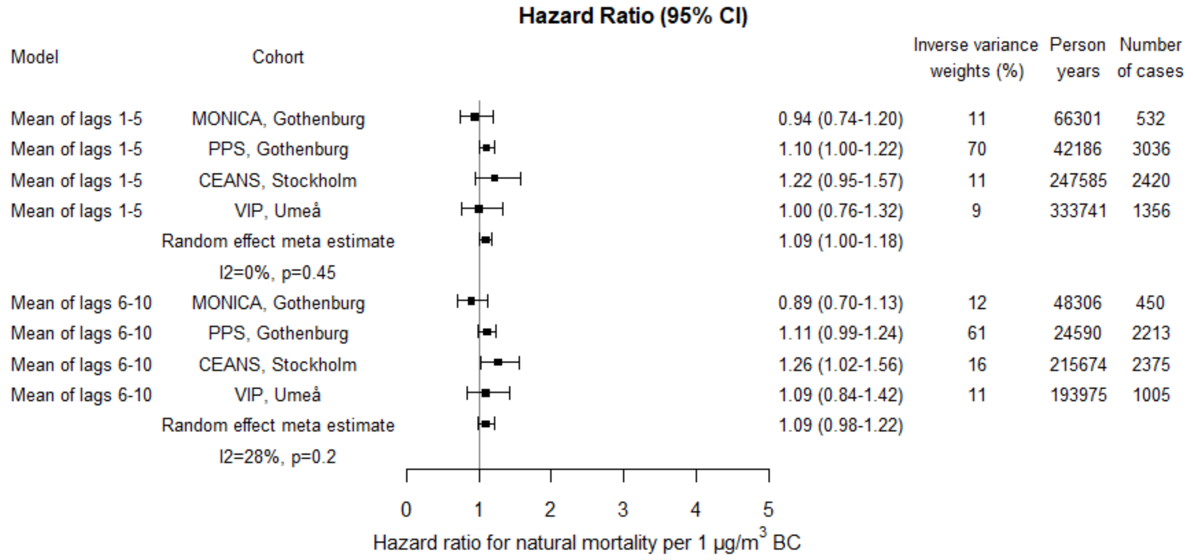

For peer review only

Figure 7. Adjusted hazard ratios of CVD mortality associated with PM10 within each cohort and by random effect meta-analyses estimates. Exposures were assessed by moving average residential concentrations within the exposure windows last 5 years and 6 to 10 years prior.

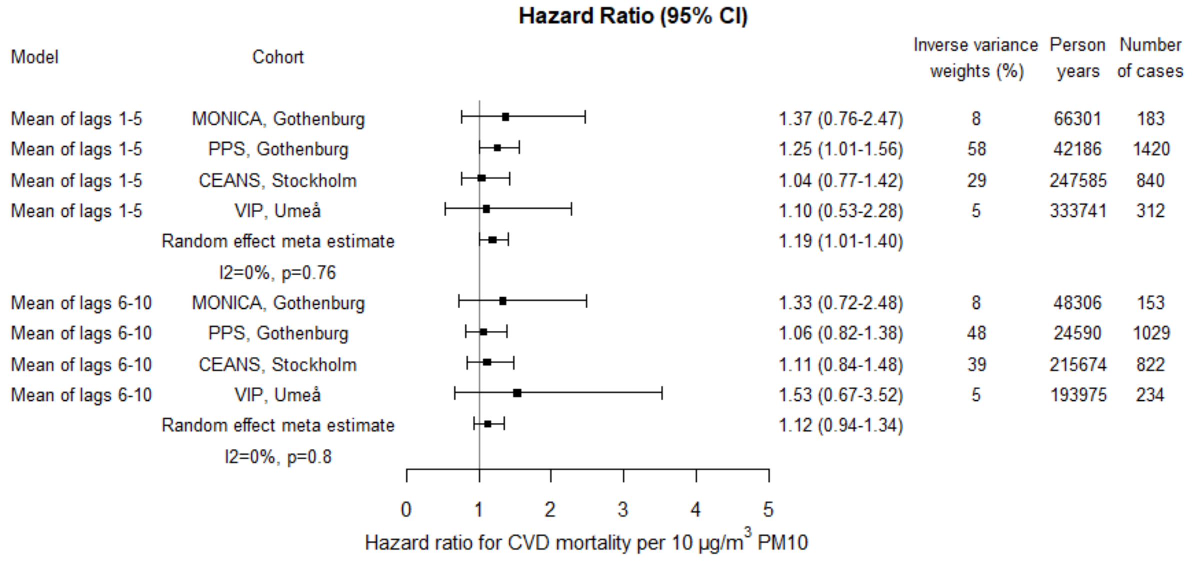

For peer review only

Figure 8. Adjusted hazard ratios of CVD mortality associated with PM2.5 within each cohort and by random effect meta-analyses estimates. Exposures were assessed by moving average residential concentrations within the exposure windows last 5 years and 6 to 10 years prior.

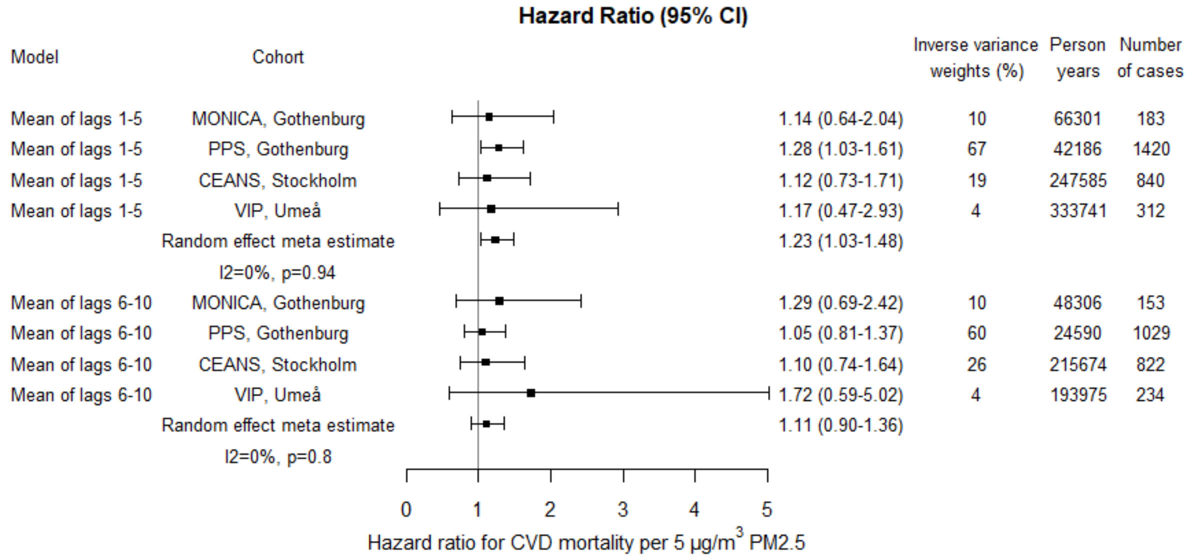

For peer review only

Figure 9. Adjusted hazard ratios of CVD mortality associated with BC within each cohort and by random effect meta-analyses estimates. Exposures were assessed by moving average residential concentrations within the exposure windows last 5 years and 6 to 10 years prior.

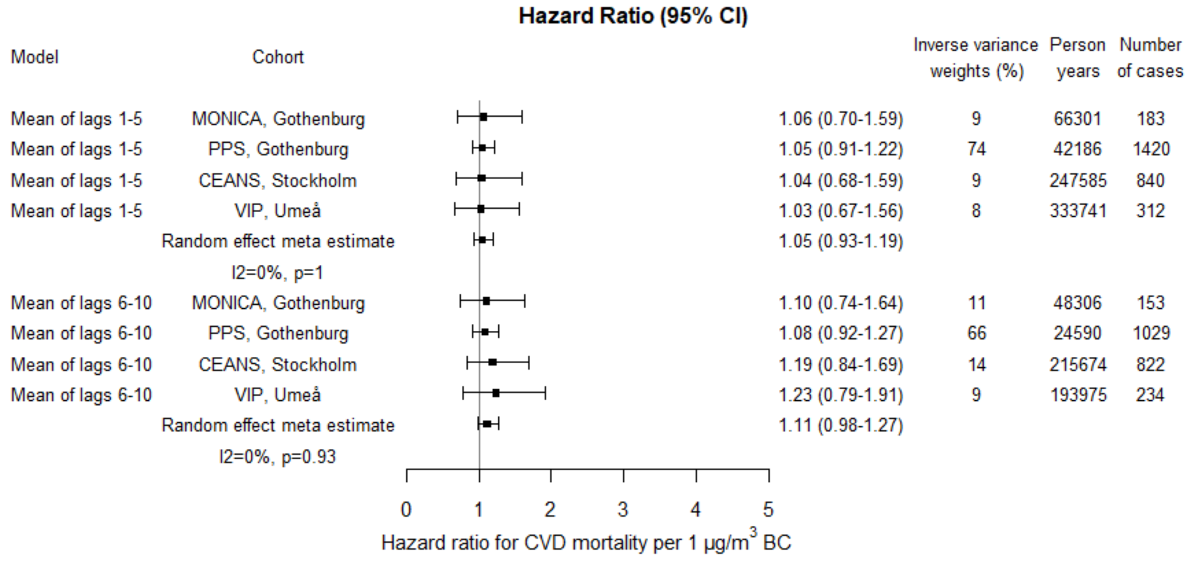

Figure S1a-c. Crude hazard ratios of natural mortality associated with a) PM10, b) PM2.5 and c) BC within each cohort and by random effect meta-analyses estimates. Exposures were assessed by moving average residential concentrations within the exposure windows last 5 years and 6 to 10 years prior.

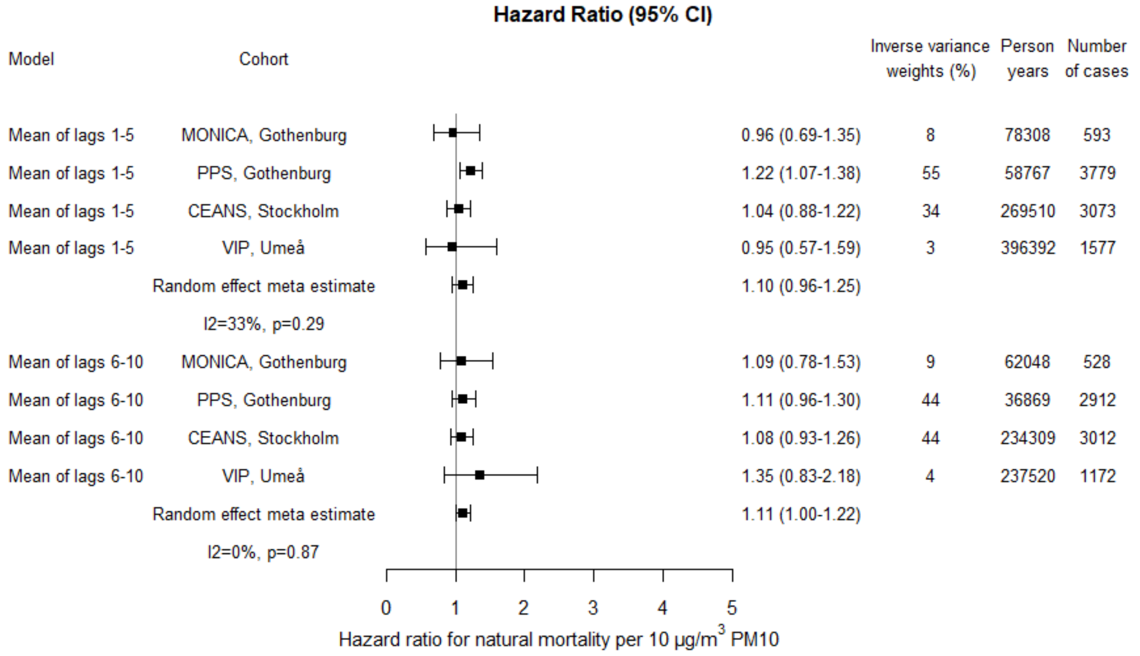

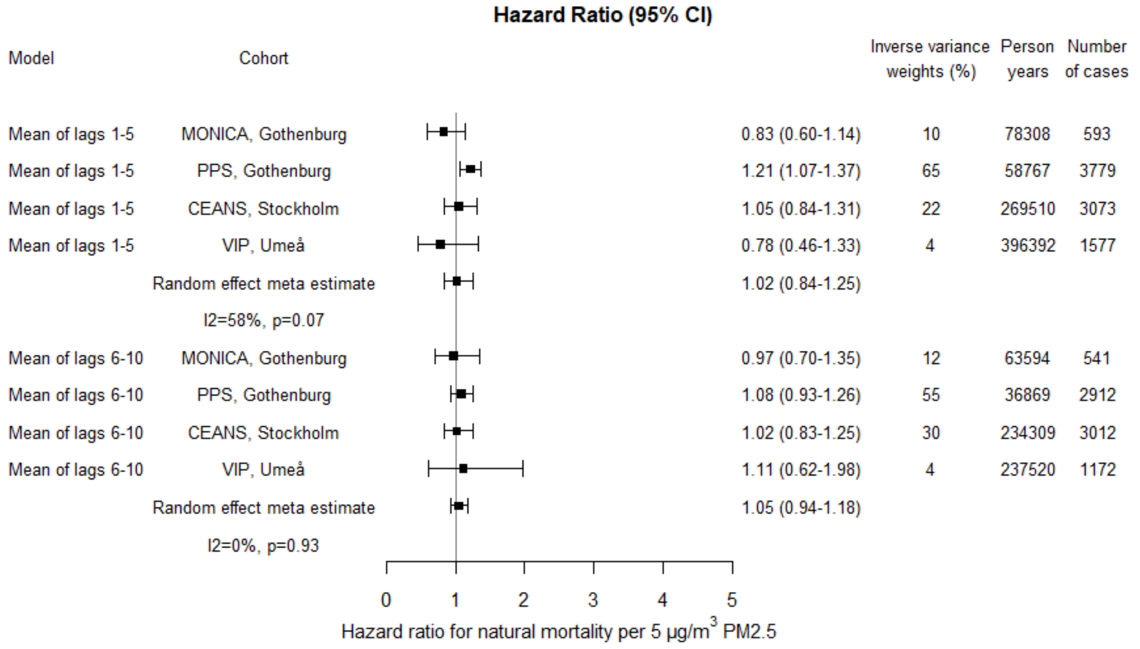

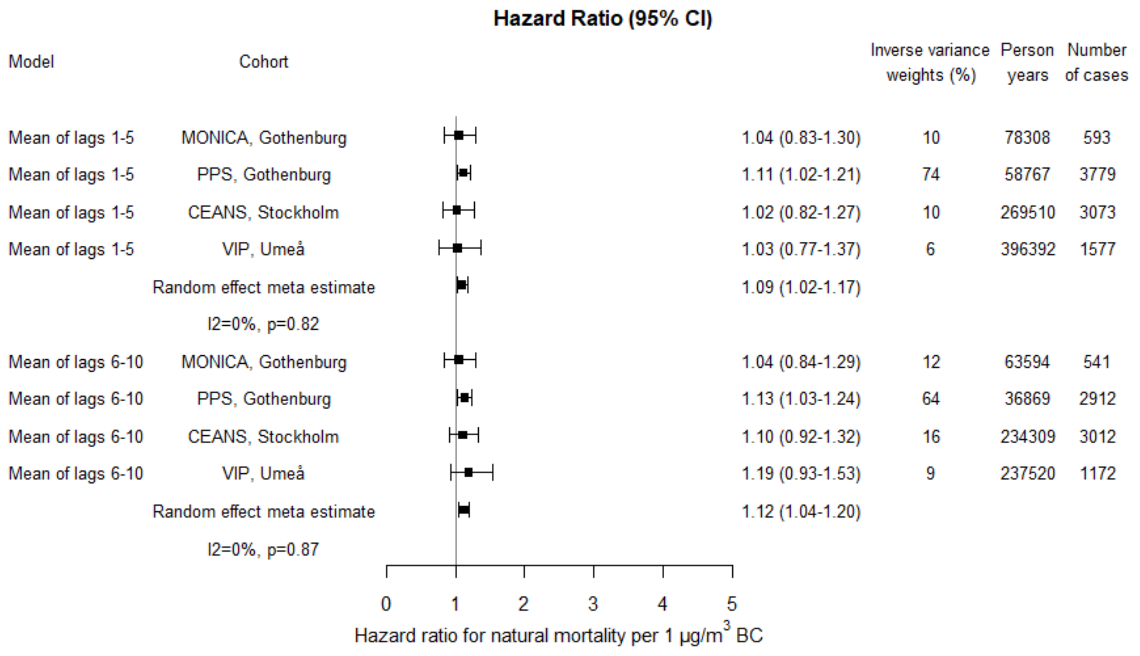

For peer review only

Figure S2a-c. Crude hazard ratios of CVD mortality associated with a) PM10, b) PM2.5 and c) BC within each cohort and by random effect meta-analyses estimates. Exposures were assessed by moving average residential concentrations within the exposure windows last 5 years and 6 to 10 years prior.

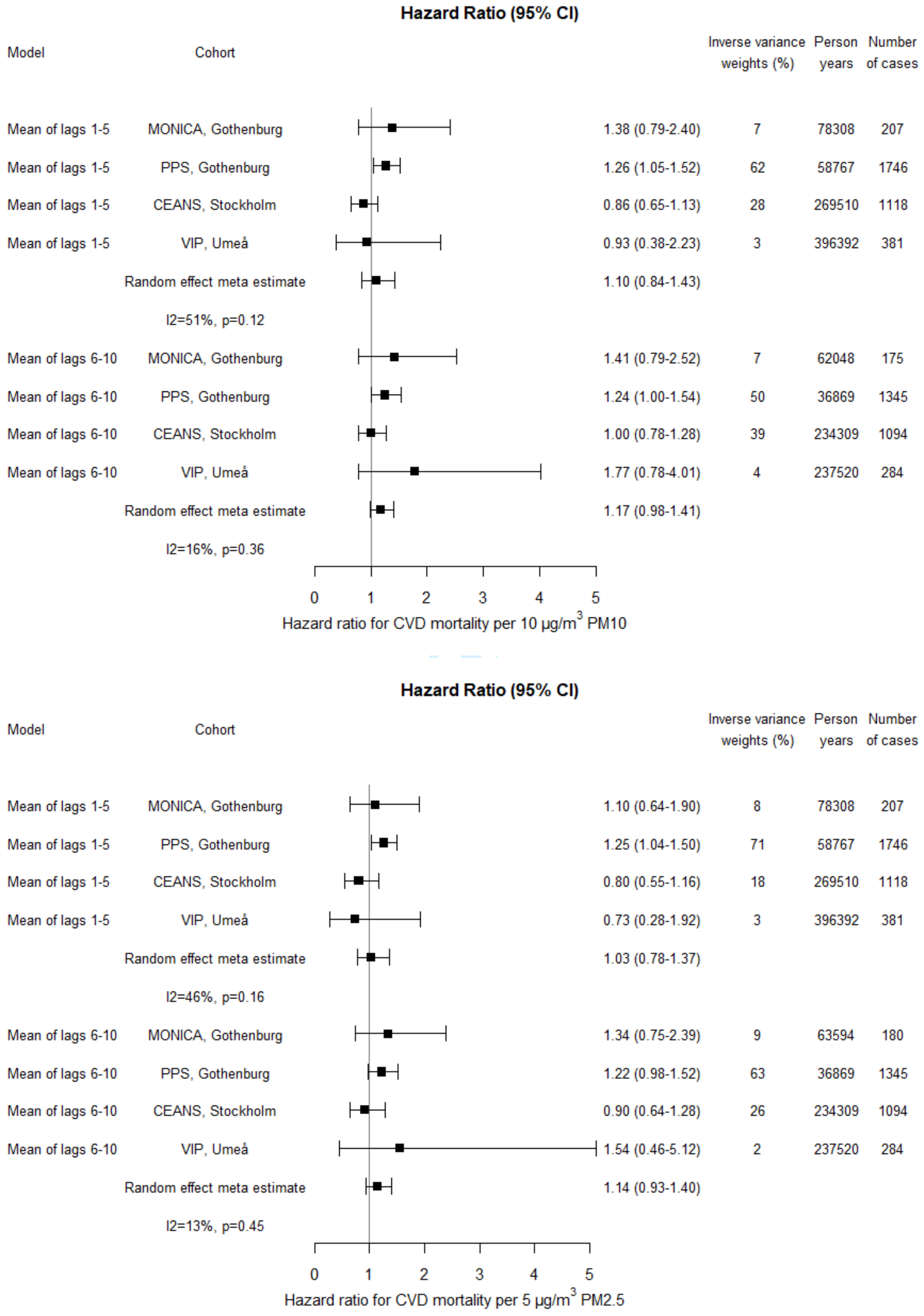

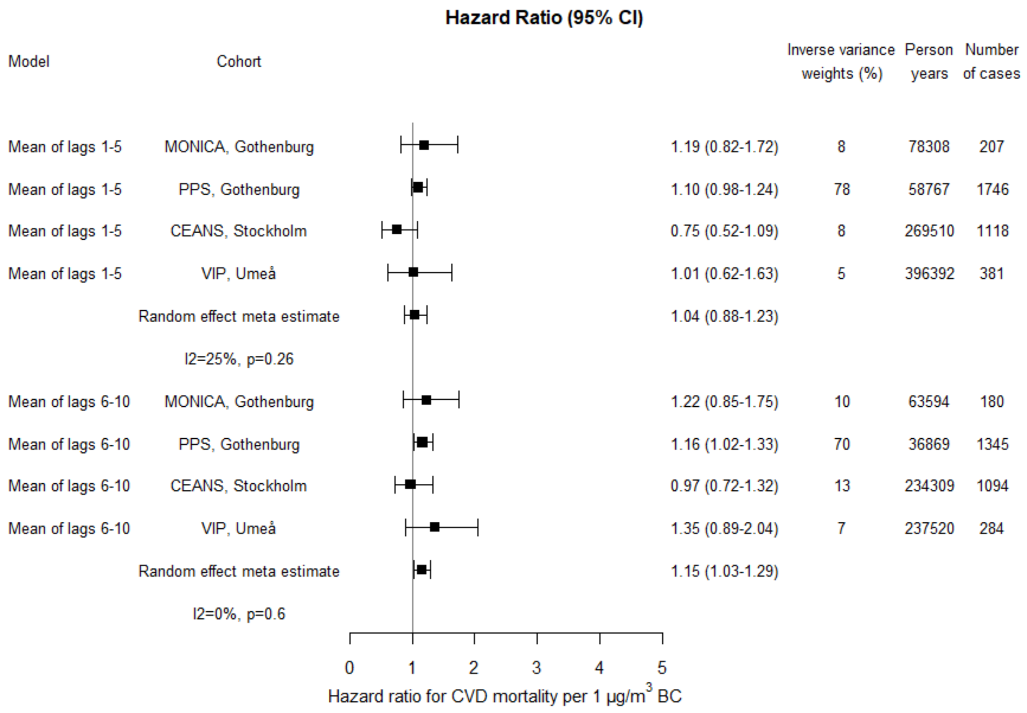

For peer review only

Figure S3a-c. Adjusted hazard ratios of lung cancer mortality associated with a) PM10, b) PM2.5 and c) BC within each cohort and by random effect meta-analyses estimates. Exposures were assessed by moving average residential concentrations within the exposure windows last 5 years and 6 to 10 years prior.

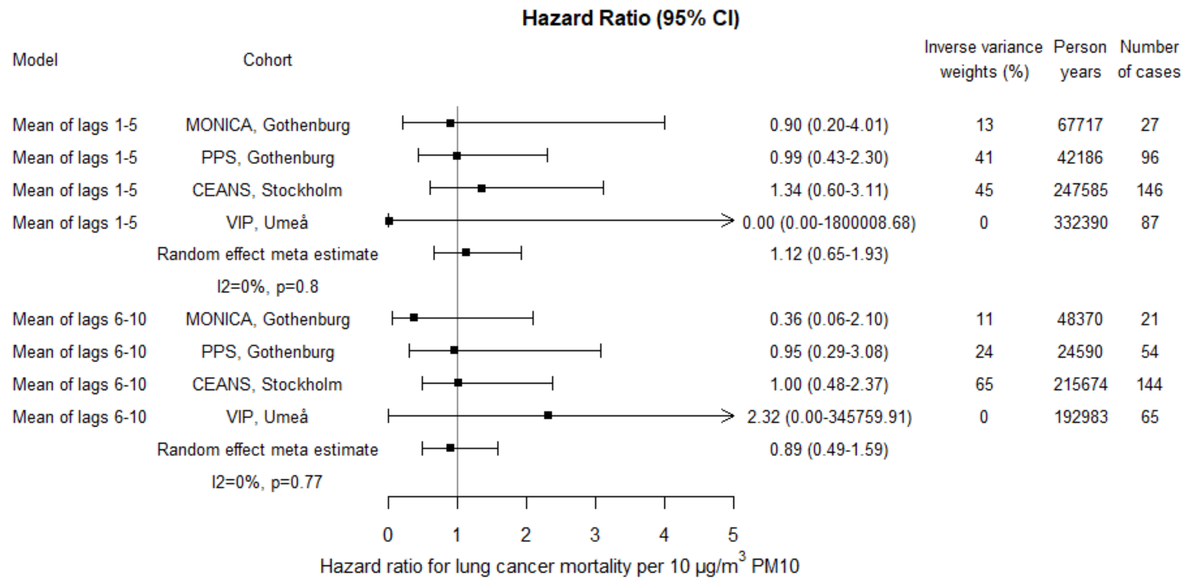

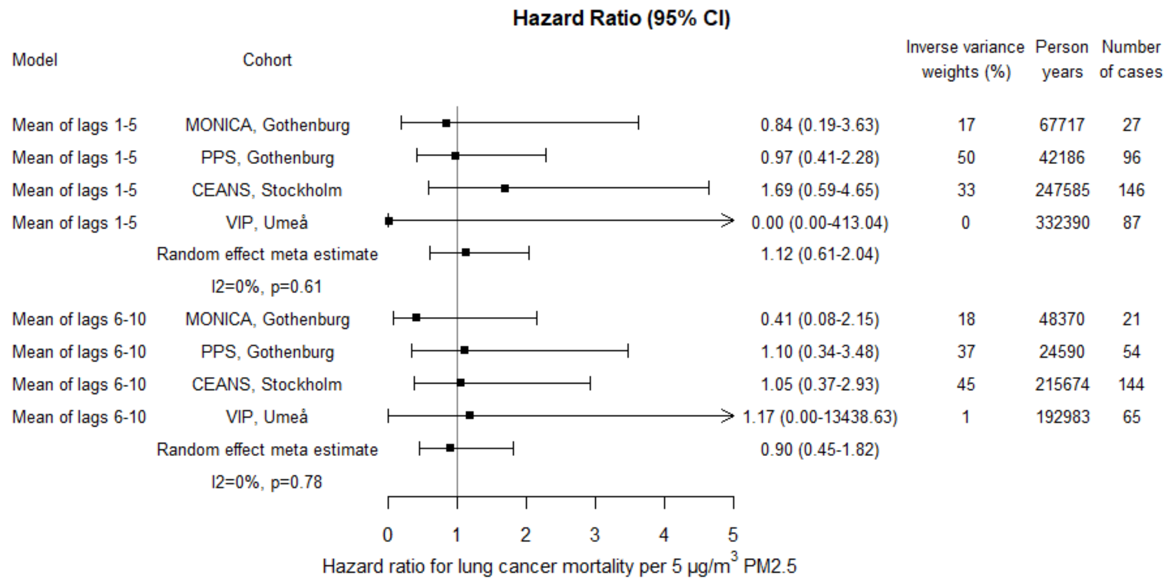

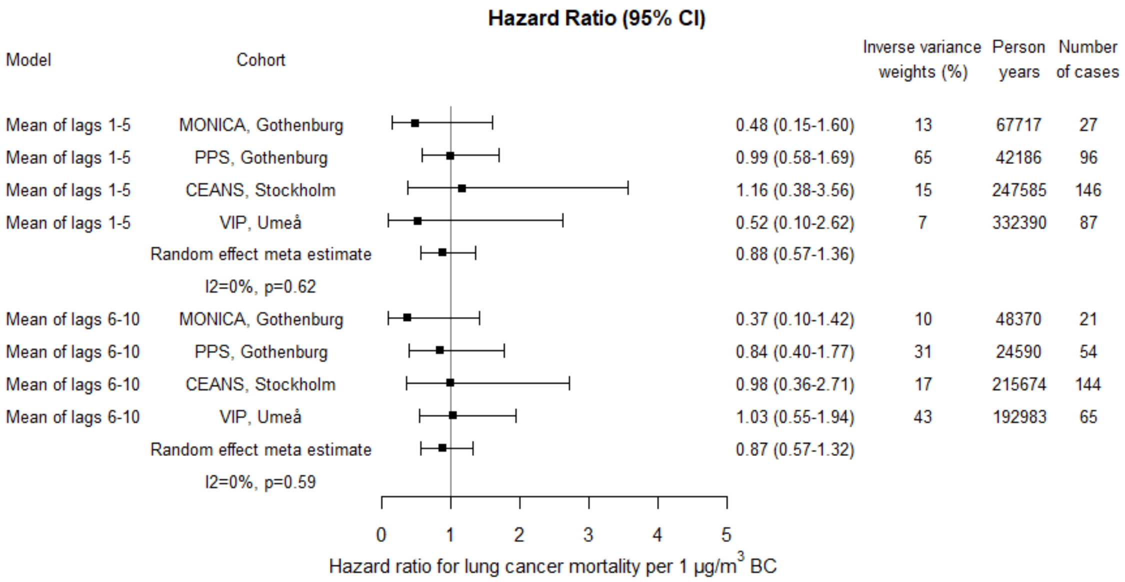

peer review only

Figure S4a-c. Adjusted hazard ratios of respiratory mortality associated with a) PM10, b) PM2.5 and c) BC within each cohort and by random effect meta-analyses estimates. Exposures were assessed by moving average residential concentrations within the exposure windows last 5 years and 6 to 10 years prior.

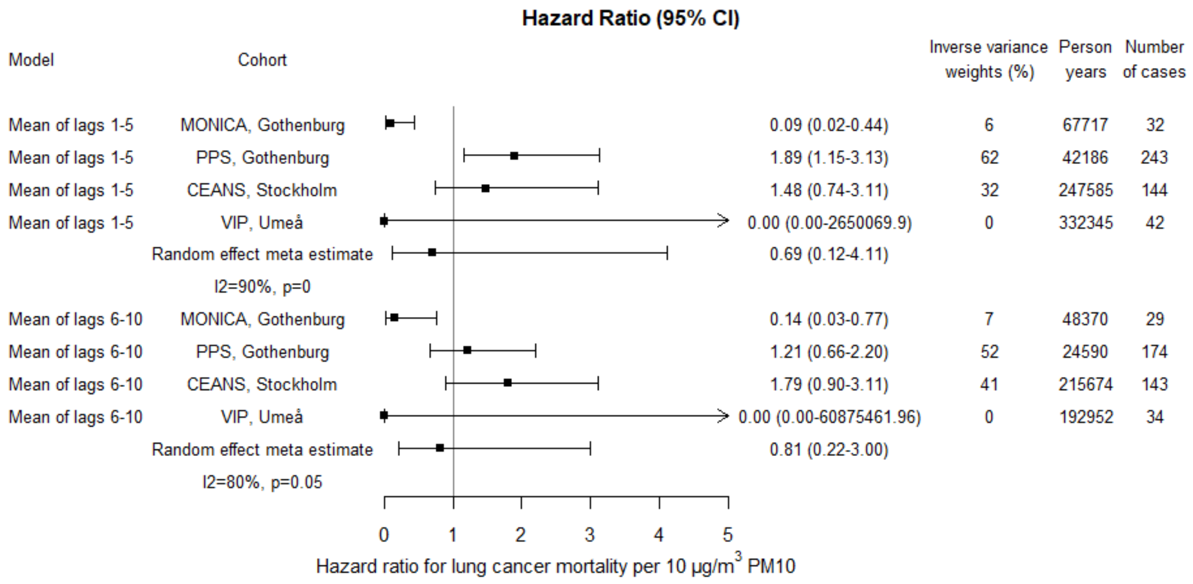

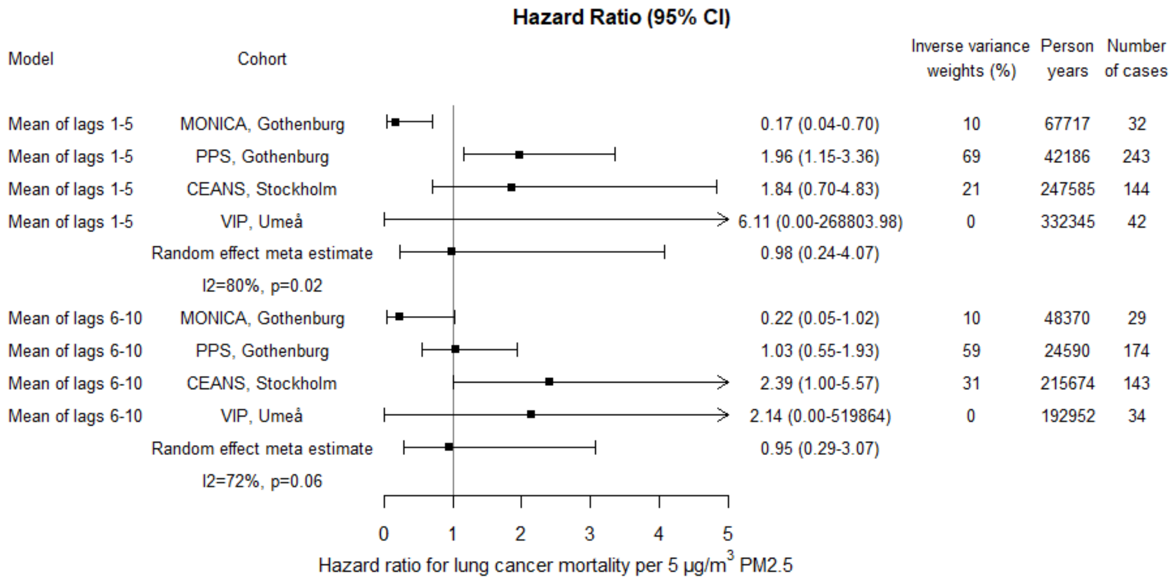

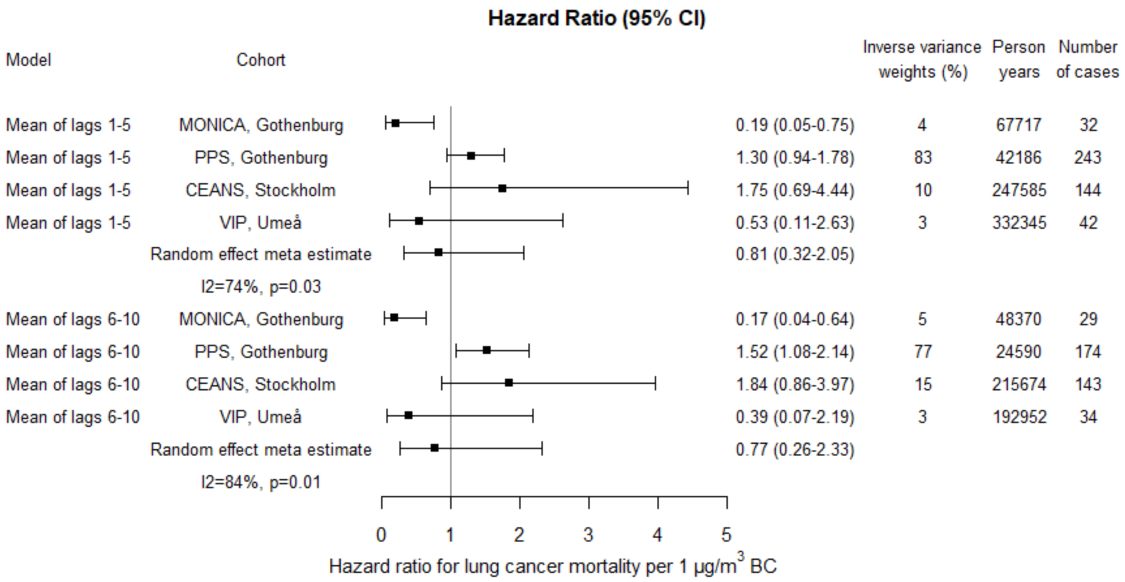

peer review only

Figure S5a-c. Adjusted hazard ratios of mortality by other casues (not CVD, respiratory, lung cancer or external causes) associated with a) PM10, b) PM2.5 and c) BC within each cohort and by random effect meta-analyses estimates. Exposures were assessed by moving average residential concentrations within the exposure windows last 5 years and 6 to 10 years prior.

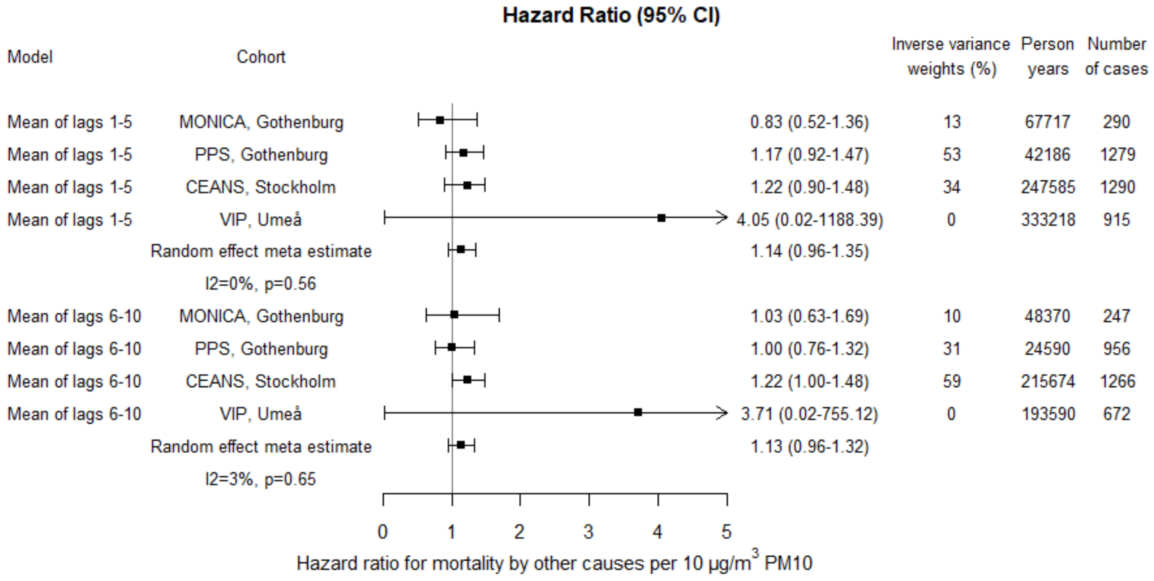

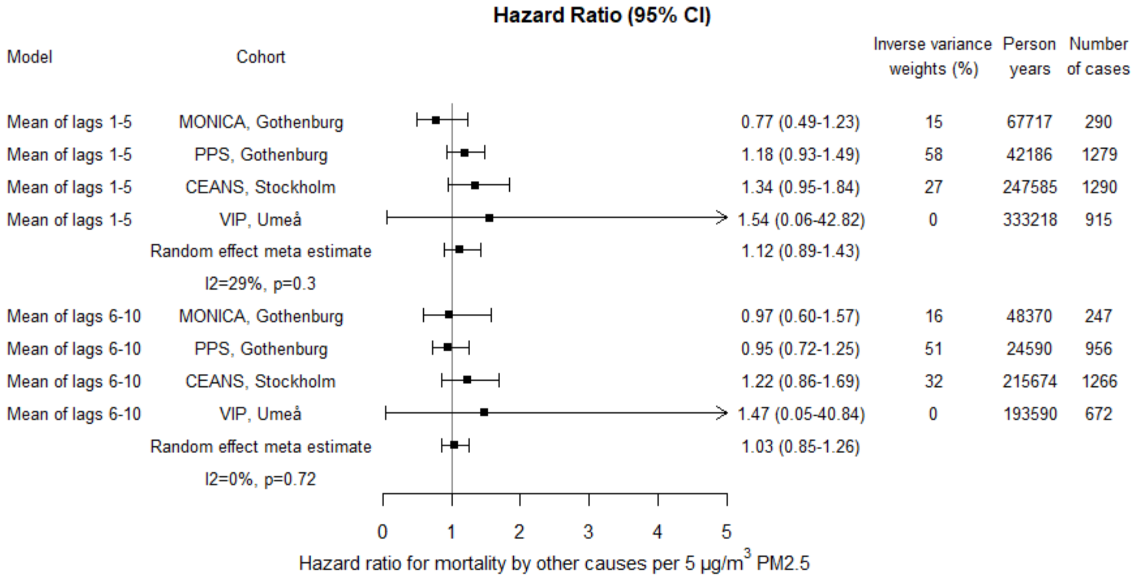

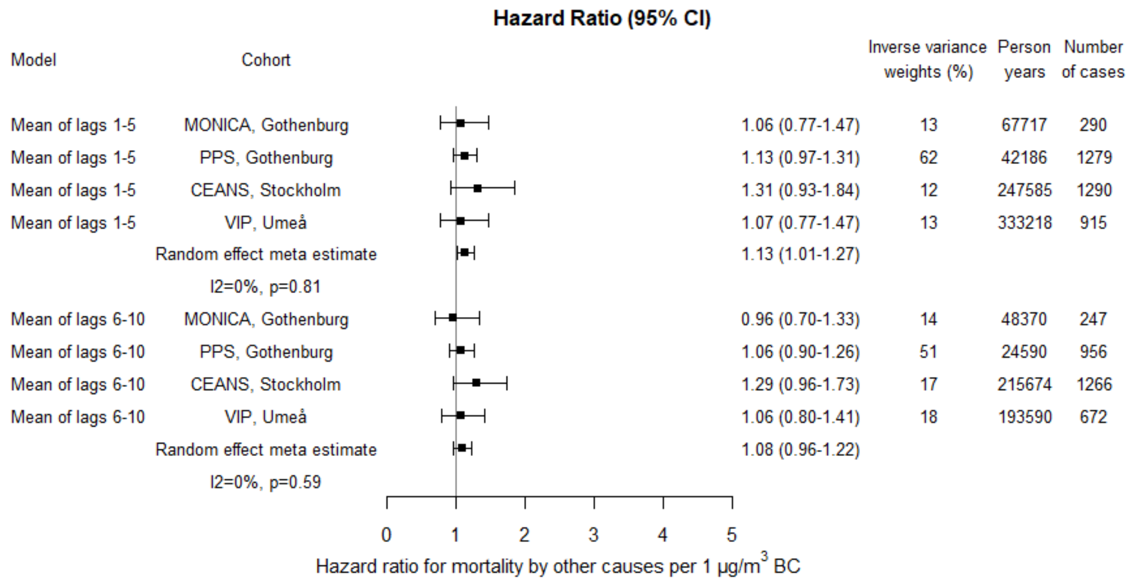

peer review only

Figure S6a-b. Adjusted hazard ratios of a) natural mortality and b) CVD mortality associated with PM10, PM2.5 and BC, within each cohort and by random effect meta-analyses estimates. Exposures were assessed by the residential concentration at the year of recruitment.

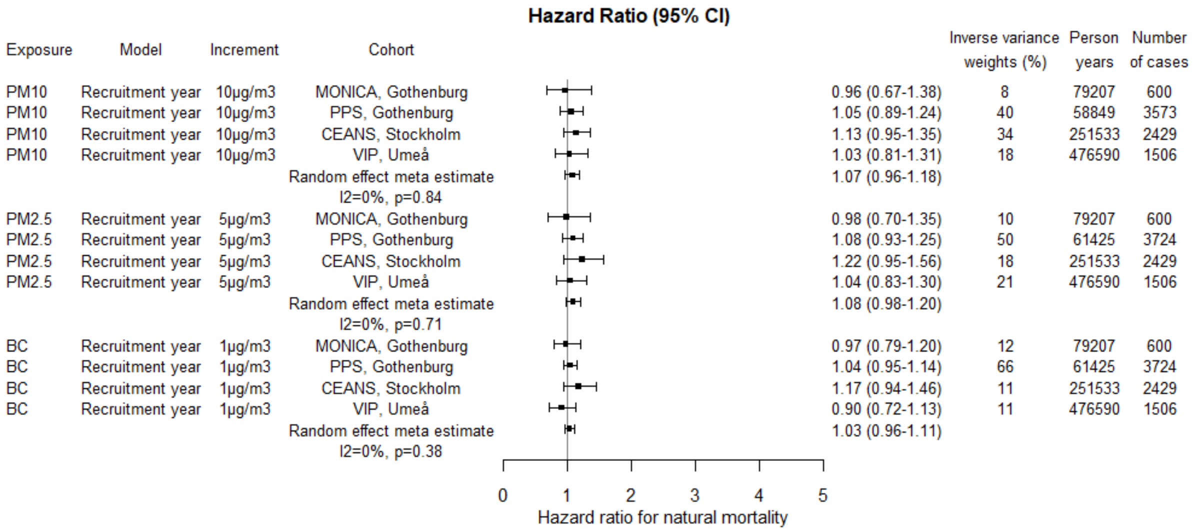

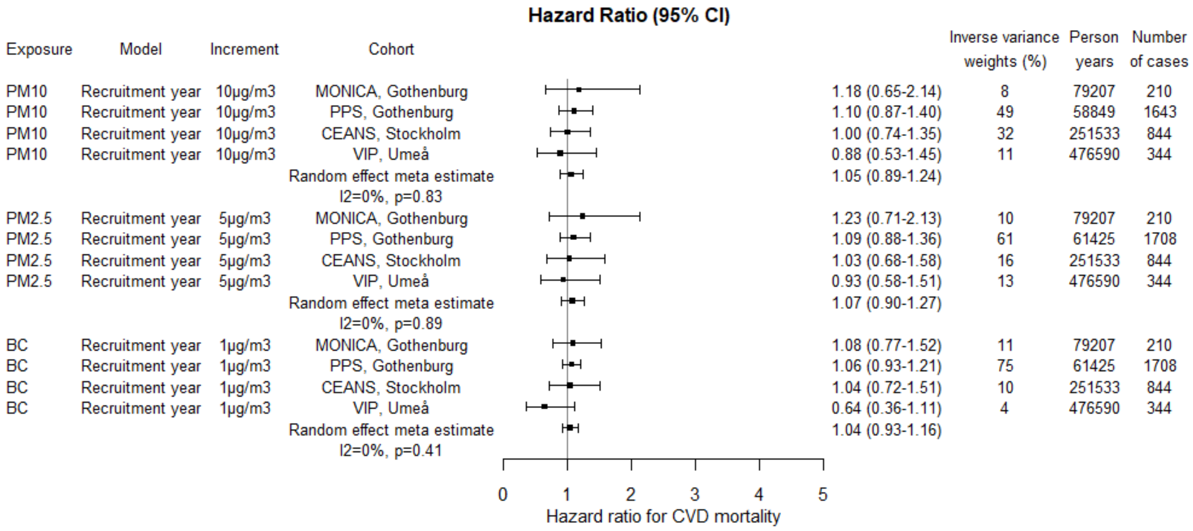

STROBE 2007 (v4) Statement—Checklist of items that should be included in reports of *cohort studies*

Section/Topic	Item #	Recommendation	Reported on page #
**Title and abstract**	1	(*a*) Indicate the study's design with a commonly used term in the title or the abstract	2
		(*b*) Provide in the abstract an informative and balanced summary of what was done and what was found	2
**Introduction**			
Background/rationale	2	Explain the scientific background and rationale for the investigation being reported	3
Objectives	3	State specific objectives, including any prespecified hypotheses	3-4
**Methods**			
Study design	4	Present key elements of study design early in the paper	4
Setting	5	Describe the setting, locations, and relevant dates, including periods of recruitment, exposure, follow-up, and data collection	4-6
Participants	6	(*a*) Give the eligibility criteria, and the sources and methods of selection of participants. Describe methods of follow-up	4-5
		(*b*) For matched studies, give matching criteria and number of exposed and unexposed	
Variables	7	Clearly define all outcomes, exposures, predictors, potential confounders, and effect modifiers. Give diagnostic criteria, if applicable	5-6
Data sources/ measurement	8*	For each variable of interest, give sources of data and details of methods of assessment (measurement). Describe comparability of assessment methods if there is more than one group	*5*
Bias	9	Describe any efforts to address potential sources of bias	10
Study size	10	Explain how the study size was arrived at	4-7
Quantitative variables	11	Explain how quantitative variables were handled in the analyses. If applicable, describe which groupings were chosen and why	5-6
Statistical methods	12	(*a*) Describe all statistical methods, including those used to control for confounding	6
		(*b*) Describe any methods used to examine subgroups and interactions	
		(*c*) Explain how missing data were addressed	6
		(*d*) If applicable, explain how loss to follow-up was addressed	6
		(*e*) Describe any sensitivity analyses	
**Results**			

For peer review only

Participants	13*	(a) Report numbers of individuals at each stage of study—eg numbers potentially eligible, examined for eligibility, confirmed eligible, included in the study, completing follow-up, and analysed	7
		(b) Give reasons for non-participation at each stage	
		(c) Consider use of a flow diagram	
Descriptive data	14*	(a) Give characteristics of study participants (eg demographic, clinical, social) and information on exposures and potential confounders	7
		(b) Indicate number of participants with missing data for each variable of interest	7
		(c) Summarise follow-up time (eg, average and total amount)	7
Outcome data	15*	Report numbers of outcome events or summary measures over time	7
Main results	16	(*a*) Give unadjusted estimates and, if applicable, confounder-adjusted estimates and their precision (eg, 95% confidence interval). Make clear which confounders were adjusted for and why they were included	7
		(*b*) Report category boundaries when continuous variables were categorized	
		(*c*) If relevant, consider translating estimates of relative risk into absolute risk for a meaningful time period	
Other analyses	17	Report other analyses done—eg analyses of subgroups and interactions, and sensitivity analyses	
**Discussion**			
Key results	18	Summarise key results with reference to study objectives	9
**Limitations**			
Interpretation	20	Give a cautious overall interpretation of results considering objectives, limitations, multiplicity of analyses, results from similar studies, and other relevant evidence	10
Generalisability	21	Discuss the generalisability (external validity) of the study results	10-11
**Other information**			
Funding	22	Give the source of funding and the role of the funders for the present study and, if applicable, for the original study on which the present article is based	12

*Give information separately for cases and controls in case-control studies and, if applicable, for exposed and unexposed groups in cohort and cross-sectional studies.

Note: An Explanation and Elaboration article discusses each checklist item and gives methodological background and published examples of transparent reporting. The STROBE checklist is best used in conjunction with this article (freely available on the Web sites of PLoS Medicine at http://www.plosmedicine.org/, Annals of Internal Medicine at http://www.annals.org/, and Epidemiology at http://www.epidem.com/). Information on the STROBE Initiative is available at www.strobe-statement.org.

For peer review only
